# *Plasmodium* pseudo-Tyrosine Kinase-like binds PP1 and SERA5 and is exported to host erythrocytes

**DOI:** 10.1038/s41598-019-44542-3

**Published:** 2019-05-31

**Authors:** Bénédicte Gnangnon, Aline Fréville, Katia Cailliau, Catherine Leroy, Caroline De Witte, David Tulasne, Alain Martoriarti, Vincent Jung, Ida Chiara Guerrera, Sabrina Marion, Jamal Khalife, Christine Pierrot

**Affiliations:** 1 Center for Infection and Immunity of Lille, Inserm U1019-CNRS UMR 8204, Université de Lille, Institut Pasteur de Lille, 1 Rue du Professeur Calmette, F-59000 Lille, France; 2 Université de Lille, CNRS, INRA, UMR 8576-UGSF - Unité de Glycobiologie Structurale et Fonctionnelle, F-59000 Lille, France; 3 University of Lille, CNRS, Institut Pasteur de Lille, UMR 8161 - M3T - Mechanisms of Tumorigenesis and Target Therapies, F-59000 Lille, France; 4 Proteomics platform 3P5-Necker, Université Paris Descartes - Structure Fédérative de Recherche Necker, INSERM US24/CNRS UMS3633, 14 Rue Maria Helena Vieira Da Silva, Paris, 75014 France

**Keywords:** Phosphorylation, Parasite biology

## Abstract

Pseudokinases play key roles in many biological processes but they are poorly understood compared to active kinases. Eight putative pseudokinases have been predicted in *Plasmodium* species. We selected the unique pseudokinase belonging to tyrosine kinase like (TKL) family for detailed structural and functional analysis in *P*. *falciparum* and *P*. *berghei*. The primary structure of *Pf*pTKL lacks residues critical for kinase activity, supporting its annotation as a pseudokinase. The recombinant pTKL pseudokinase domain was able to bind ATP, but lacked catalytic activity as predicted. The sterile alpha motif (SAM) and RVxF motifs of *Pf*pTKL were found to interact with the *P*. *falciparum* proteins serine repeat antigen 5 (SERA5) and protein phosphatase type 1 (PP1) respectively, suggesting that pTKL has a scaffolding role. Furthermore, we found that PP1c activity in a heterologous model was modulated in an RVxF-dependent manner. During the trophozoite stages, *Pb*pTKL was exported to infected erythrocytes where it formed complexes with proteins involved in cytoskeletal organization or host cell maturation and homeostasis. Finally, genetic analysis demonstrated that viable strains obtained by genomic deletion or knocking down *PbpTKL* did not affect the course of parasite intra-erythrocytic development or gametocyte emergence, indicating functional redundancy during these parasite stages.

## Introduction

Malaria remains one of the most life-threatening parasitic diseases in the world, with 219 million cases leading to an estimated 435,000 deaths worldwide in 2017^1^. Among the *Plasmodium* species that infect humans, *P*. *falciparum* is the most virulent and is responsible for more than 90% of deaths, mostly in African children less than 5 years old. Thanks to preventative measures such as bed nets and treatments mainly involving artemisinin-based combination therapy (ACT), the global incidence of malaria fell by 38% between 2010 and 2016^2,3^. However, the rapid spread of ACT-resistant *Plasmodium* strains in South-East Asia is alarming, and innovative antimalarial strategies are therefore needed^4–6^.

The life cycle of *Plasmodium* species is complex because the parasite infects two hosts and progresses through various stages of development that are replicative, invasive or involved in sexual reproduction^7,8^. To sustain development and respond to environmental changes, the parasite uses post-translational modifications (PTMs) for the dynamic modulation of protein function, in many cases phosphorylation^9^. This reversible modification is catalyzed by protein kinases and the phosphate groups are removed by protein phosphatases. Phosphoproteomics approaches have revealed that kinases and phosphatases play key roles in cell growth, division, motility, invasion and communication with host cells during the *Plasmodium* life cycle^10–13^. The essential role of these enzymes has also been demonstrated using phosphatase and kinase inhibitors^14–16^, most of which are deleterious to the parasite^17^. Reverse genetics has helped to decipher the role of several of these enzymes at each developmental stage^12,18,19^. These studies, as well as the more detailed characterization of specific enzymes, have improved our understanding of *Plasmodium* signal transduction pathways^20^.

Although phosphorylated tyrosine residues can be detected in the late *Plasmodium* development stages^13^, the parasite kinome is mainly composed of serine/threonine (S/T) kinases^21^. Hanks-type S/T kinases are membrane-bound or cytoplasmic enzymes that share common features^22^, including a 12-subdomain signature in the catalytic domain required for kinase activity^23,24^. Active kinases take part in regulatory networks that allow the integration of signals by controlling protein structure and activity. Enzymes that lack one of the catalytic residues required for kinase activity are classified as pseudokinases^25^. They constitute 10% of the mammalian kinome and fulfil a plethora of biological roles, depending on their subdomain composition and their ability to bind ATP and/or divalent ions^26,27^. They can introduce PTMs, either as active kinases if they have evolved compensatory mechanisms to overcome the lack of one or more key subdomains (e.g., CASK^28^, Haspin^29^, WNK1^30^, and other examples that have been reviewed^31,32^) or as AMPylation catalyzers^33^, demonstrating the functional versatility of kinase domains. Some pseudokinases also play a role in cell signaling. They can notably function as protein–protein interaction platforms or allosteric regulators of various enzymes, including kinases^34^, or of protein trafficking and subcellular localization^35^. Their importance in metazoan cell homeostasis is highlighted by their role in diseases, including cancer^36^.

In apicomplexan parasites, most pseudokinases are poorly characterized, but the highly polymorphic ROP5 family in *Toxoplasma gondii* has been studied in detail^37^. *Tg*ROP5 binds ATP but lacks kinase activity *in vitro*, probably due to the absence of a catalytic aspartate residue in subdomain VI. However, the conservation of this non-canonical motif is necessary for parasitic virulence^37^. *Tg*ROP5 forms a complex with the kinases *Tg*ROP17 and *Tg*ROP18, causing the synergistic neutralization of host immunity-related GTPases (IRGs), favoring infection by protecting the parasite from cell-mediated clearance^38,39^. Reverse genetics has been used to confirm that *Tg*ROP5 is required for parasite virulence in genetically divergent strains^40^, revealing that parasite pseudoenzymes can play essential roles at the host–pathogen interface.

The *Plasmodium* kinome was initially predicted to include eight pseudokinases^41^, five of which are probably essential for the intra-erythrocytic development of the parasite^19^. Here, we report the characterization of the unique *Plasmodium* pseudo-tyrosine kinase-like (pTKL) protein, previously named TKL5^42^. We evaluated its potential for kinase activity, characterized its interactions to establish its molecular function, and investigated its biological role using different knockout and knock-down approaches.

## Results

### *Pf*pTKL transcript sequencing and protein annotation

To confirm *PfpTKL* transcription, we performed overlapping PCR on reverse transcribed *Pf*3D7 total RNA using primer pairs derived from the predicted PF3D7_1106800 sequence (Supplementary Fig. S1 and Table S1). We analyzed 6–12 sequences amplified from two independent reverse transcribed RNA preparations for each of the eight transcript fragments (length 600–1200 bp). The deduced amino acid sequence (1483 residues) was compared to the PlasmoDB^43^ predicted sequence. Both sets of data were identical with the exception of minor substitutions that did not affect the overall annotation (Supplementary Fig. S1).

**Figure 1:**
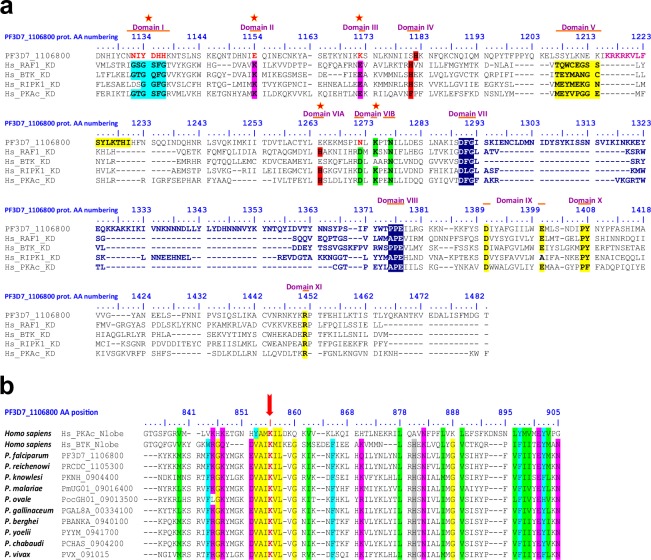
*In silico* annotation of PF3D7_1106800 (*Pf*pTKL) catalytic domains. (**a**) *Pf*pTKL has a pseudo-kinase domain. Sequence alignment between human PKAc (used as the kinase domain reference), RAF1 (used as a TKL reference), RIPK1 (used as a pseudo-TKL reference), BTK (used as a tyrosine kinase reference) and *Pf*pTKL kinase domains. The kinase subdomains (I to XI on the sequence alignment) were delineated according to Hanks & Hunter^23^ and are shaded. Non canonical S/T kinase subdomain amino acids are shown in red and marked with a red star. The activation loop residues are shown in dark blue. (**b**) *Pf*pTKL possesses a Bruton’s tyrosine kinase (BTK) N-terminal lobe-like domain upstream of its pseudo-kinase domain. Protein sequence alignment of BTK-like domains of *Pf*pTKL and its *Plasmodium* homologs with the N-terminal lobe sequences of human BTK and PKAc. The alignment was created with MAFFT^89^ and conserved residues were shaded using BioEdit v7.2.5^116^. The ATP-binding lysine conserved across all the aligned sequences is indicated with a red arrow.

**Figure 2:**
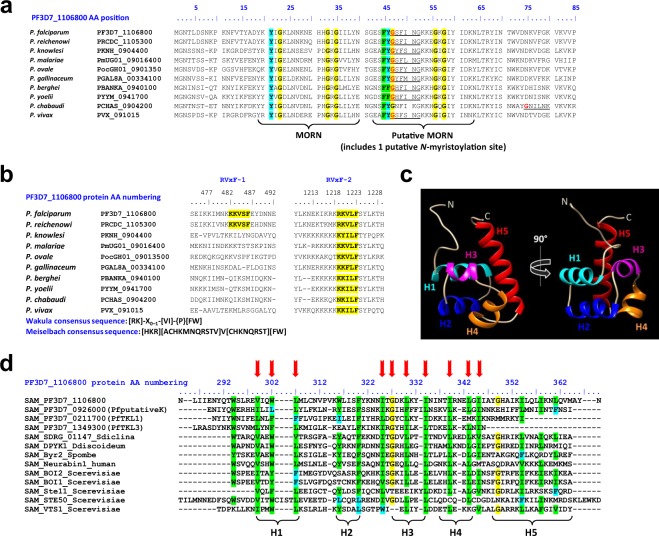
*In silico* annotation of *Pf*pTKL interaction motifs and domains. (**a**) *Plasmodium Pf*pTKL homologs share common N-terminal features beside the MORN motif(s). The alignment was created with MAFFT^89^ and residues 100% conserved over all of the sequences were shaded using BioEdit v7.2.5^116^. The putative N-myristoylation site is shown in red. (**b**) *Plasmodium Pf*pTKL homologs possess one or two RVxF motifs. The alignment was created with MAFFT^89^. RVxF motif consensus sequences used *in silico* to detect RVxF1 and RVxF2 are displayed below the alignment^86,87^. (**c**) The *Pf*pTKL SAM undergoes classic folding. Tertiary structure *Pf*pTKL SAM predicted with Phyre2^81^ (75 residues (97%) modeled at >90% accuracy). Structure annotation and shading were achieved with Chimera v1.10.1^117^. (**d**) *Plasmodium* SAMs showing classic composition compared with SAM references. Sequence alignment created with MAFFT^89^ between the four *P*. *falciparum* SAMs and reference SAMs (Sdiclina = *Saprolegnia diclina*, Scerevisiae = *Saccharomyces cerevisiae*, Spombe = *Schizosaccharomyces pombe*, Ddiscoideum = *Dictyostelium discoideum*). Conserved residues were shaded using BioEdit v7.2.5^116^. Arrows indicate residues conserved in SAMs. Below the alignment are delineated the five α-helices (H1-H5) of the SAM core^118^.

**Figure 3:**
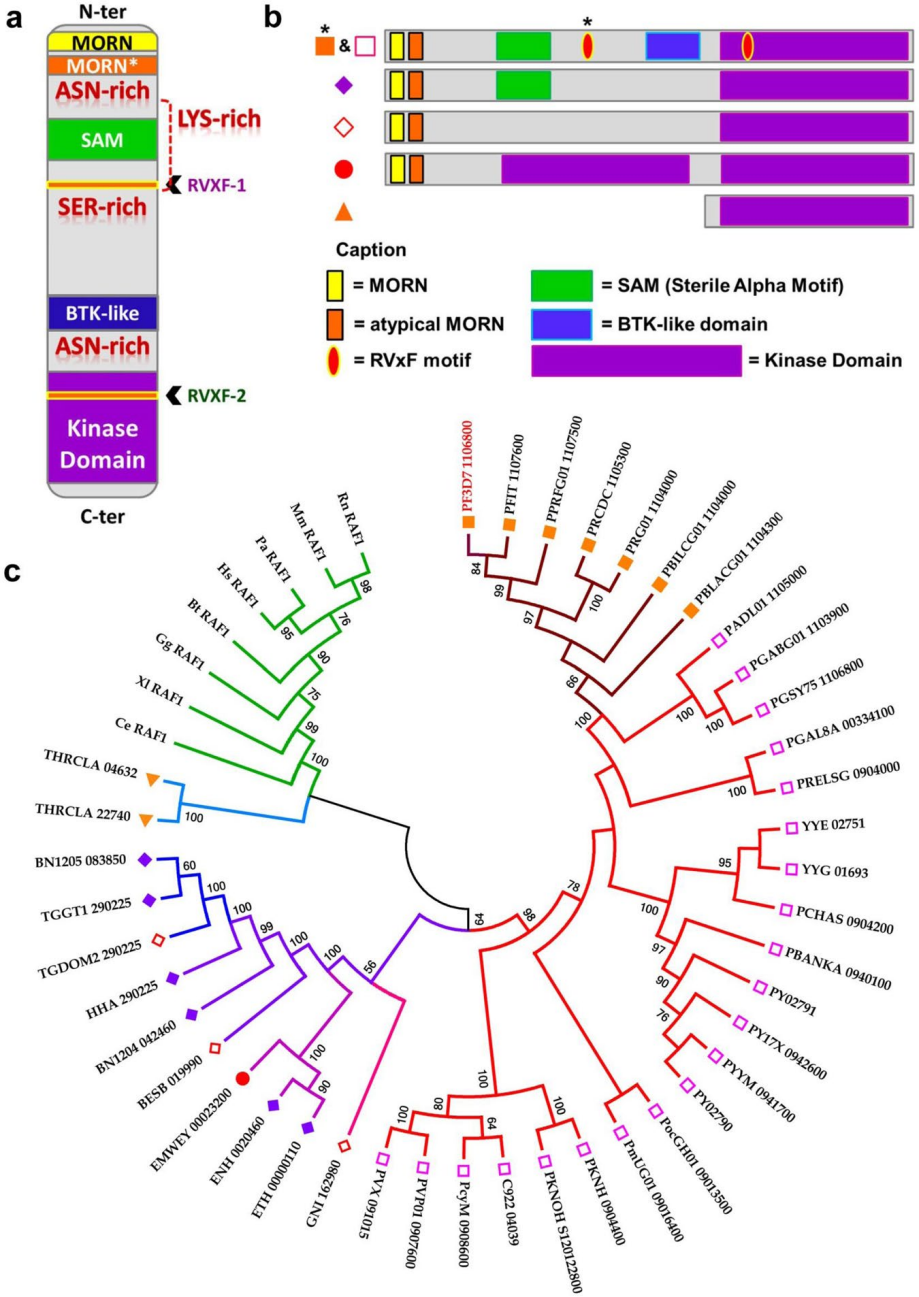
Molecular structure and phylogenetic analysis of *Pf*pTKL and its homologs in the *Plasmodium* genus. (**a**) *Pf*pTKL molecular structure. All protein domains/motifs (MORN, SAM, RVxF, BTK-like, kinase) identified *in silico* are shown, as well as amino acid repeat regions (Asn = asparagine, Lys = lysine and Ser = serine, delineated using ScanProsite^80^). (**b**) Comparison of *Pf*pTKL and its homologs. Sequence alignment was created in MAFFT^89^ and was used to assess conserved domains. Symbols on the left are linked to the proteins included in the phylogenetic analysis displayed in (**c**). (**c**) Phylogenetic analysis of *Pf*pTKL and its homologs. Maximum likelihood phylogenetic analysis of *Pf*pTKL homologs from: Alveolata: PF3D7 = *Plasmodium falciparum* 3D7, PFIT = *P*. *falciparum* IT, PPRFG01 = *P*. *praefalciparum* G01, PRCDC = *P*. *reichenowi* CDC, PRG01 = *P*. *reichenowi* G01, PBILCG01 = *P*. *billcollinsi* G01, PBLACG01 = *P*. *blacklocki* G01, PADL01 = *P*. *adleri* G01, PGAB01 = *P*. *gaboni* G01, PGSY75 = *P*. *gaboni* SY75, PYYM = *P*. *yoelii yoelii* YM, PY = *P*. *yoelii* 17XNL, PBANKA = *P*. *berghei* ANKA, PCHAS = *P*. *chabaudi*, YYE = *P*. *vinckei vinckei* VINCKEI, YYG = *P*. *vinckei petteri* CR, PGAL8A = *P*. *gallinaceum* 8A, PRELSG = *P*. *relictum* SGS1-like, PocGH01 = *P*. *ovale curtisi* GH01, PmUG01 = *P*. *malariae* UG01, PKNOH = *P*. *knowlesi* Malayan PK1, PKNH = *P*. *knowlesi* H, C922 = *P*. *inui* San Antonio 1, PcyM = *P*. *cynomolgi* M, PVP01 = *P*. *vivax* 01, PVX = *P*. *vivax* Sal 01, GNI = *Gregarina niphandrodes*, ETH = *Eimeria tenella*, ENH = *Eimeria necatrix*, EMWEY = *Eimeria maxima*, BESB = *Besnoitia besnoiti*, BN1204 = *Neospora caninum*, HHA = *Hammondia hammondi*, TGDOM2 = *Toxoplasma gondii* DOM2, TGGT1 = *T*. *gondii* GT1, BN1205 = *T*. *gondii* VEG; Stramenopile: THRCLA = *Thraustotheca clavata*. Eight RAF1 protein sequences from Opisthokonta: Mammals (Rn = *Rattus norvegicus*, Mm = *Mus musculus*, Pa = *Pongo abelii*, Hs = *Homo sapiens*, Bt = *Bos taurus*), Aves (Gg = *Gallus gallus*), Amphibia (Xl = *Xenopus laevis*), Chromaderea (Ce = *Caenorhabditis elegans*) were included in the analysis as an outgroup.

More detailed analysis revealed the presence of multiple domains (Figs 1 and 2; summarized in Table 1 and showed in Fig. 3a). From the N-terminus to the C-terminus, we identified two membrane occupation recognition nexus (MORN) motifs (Fig. 2a), one classical sterile alpha motif (SAM) predicted to bind proteins (Fig. 2c,d; Supplementary Fig. S5), one Bruton’s tyrosine kinase (BTK)-like N-terminal lobe, designated as BTK-like domain (Fig. 1b; Supplementary Fig. S3) and one kinase domain (KD; Fig. 1a). We found that the KD lacks key residues required for ATP stabilization and phosphoryl transfer, confirming that *Pf*pTKL is a pseudokinase (Fig. 1a). Two putative RVxF motifs, described as protein phosphatase 1 (PP1) catalytic subunit PP1c binding motifs^44^, were also identified (Fig. 2b). RVxF1 was localized to the C-terminal side of the SAM whereas RVxF2 was localized within the KD. Low-complexity regions enriched in asparagine, lysine or serine were identified between the predicted folded domains.

**Table 1:** *Pf*pTKL domain/motif annotation results.

Domain/motif name	*Pf*pTKL region (AA)	Data from the literature	*Pf*pTKL annotation data (this study)
Kinase domain (KD)	1080–1483 (Fig. 1a see Supplementary Table S2 for details)	Kinase domains are composed of 12 subdomains^23^, six of which are critical for kinase activity^25^. *Pf*pTKL lacks some canonical KD subdomains^41^. *Pb*pTKL is a pseudokinase^19^.	*Pf*pTKL KD (Fig. 1a): —belongs to the tyrosine kinase-like (TKL) family^42^ (Supplementary Fig. S1). —lacks the following subdomains: -I (glycine triad) -II (ATP-binding lysine) although there might be an alignment offset because the *Pf*pTKL KD N-terminal lobe is longer than in reference kinases -III (αC helix glutamate can interact with subdomain II lysine) —includes some specificities: -VIB aspartate (ATP γ-phosphate transfer catalyzer) is mutated to asparagine -a ~50-residue insertion in its activation loop (shown in red in Fig. 1a).
Bruton’s tyrosine kinase-like (BTK-like) domain	830–905 (Fig. 1b)	BTK is named after Dr Ogden Carr Bruton, who discovered X-linked agammaglobulinemia (XLA) in boys in 1952. This cytoplasmic tyrosine kinase (mostly in B lymphocytes) needs to bind lipids via its PH-TH module to adopt an active conformation^104^. BTK is a promising target in tumors^105^.	BTK-like domain: —was predicted by most of the prediction tools we used. —alignment with *Hs*PKA and *Hs*BTK N-terminal lobe sequences shows the conservation of canonical KD subdomains II (this lysine is shaded in red in Fig. 1b), IV (highlighted in gray) and V. —tertiary structure prediction superimposes well with *Hs*BTK N-terminal lobe (Supplementary Fig. S3).
Membrane occupation recognition nexus (MORN) domain	MORN1: 19–40 MORN2: 43–63 (Fig. 2a)	First identified in Junctophilins^106^, MORN motifs have been shown to bind: -lipids and regulate kinase activity in plant PIPKs^107,108^ -the cytoskeleton as in *Tg*MORN1^109^ -membranes and is required for the localization of retinophilin in the *Drosophila melanogaster* rhabdomere^110^	There is no signal peptide at the N-terminus of *Pf*pTKL. MORN1 follows the MORN motif signature: a 14–16 residue core domain starting with Y/FxG and ending with GxG (Supplementary Fig. S4). MORN2 does not follow a canonical MORN signature but contains a putative N-myristoylation site that is conserved across all *Plasmodium* species (Fig. 2a).
RVxF motifs	RVxF1: 482–486 RVxF2: 1219–1223 (Fig. 2b)	RVxF motifs: - so-called because of their binding signature^111^ - are the most frequent binding motifs used by PP1 partners to interact with PP1c^112^ - can be predicted with consensus sequences (Fig. 2b)	RVxF1 follows the Meiselbach consensus sequence^87^. It is located in a low-complexity region, which may increase its interaction potential with PP1c^50^. It is conserved among *Pf*pTKL homologs in *P*. *praefalciparum*, *P*. *reichenowi*, *P*. *billcollinsi* and *P*. *blacklocki* (Fig. 3c). RVXF2 follows the Wakula consensus sequence^86^. It is located in a basic charged region, which could reinforce its binding potential^75^. It is conserved across all *Pf*pTKL homologs in the genus *Plasmodium* (Figs 2b and 3c).
Sterile alpha motif (SAM)	299–364 (Fig. 2d)	SAMs belong to the alpha protein family (289 folds – SCOP database^113^). They typically fold into 4–5 orthogonal α helices. They are involved in interactions with protein or RNA^71,72^. In the *Plasmodium* proteome: - there are four SAMs (*Pf*pTKL, *Pf*TKL1, *Pf*TKL3, PF3D7_0926000)^66^ - there are two SAM-like domains involved in interactions with RNA (prototypical HTH motifs) in *Pf*g27^114,115^.	The *Pf*pTKL SAM domain is predicted to fold classically (Fig. 2c). The *Pf*pTKL SAM core domain includes all SAM canonical residues (Fig. 2d). The *Pf*pTKL SAM domain is close to SAMs involved in protein–protein interactions (Supplementary Fig. S5). This phylogenetic analysis includes *Plasmodium* SAM and SAM-like sequences as well as reference SAM and SAM-like sequences across the tree of life.

### Phylogenetic classification of *Pf*pTKL

We constructed a phylogenetic tree comprising *PfpTKL* homologs and a RAF1 outgroup. The tree with the highest log likelihood is presented in Fig. 3c. The topology of the tree reflects the general classification of eukaryotes^45^, with the apicomplexa clustering together in one clade (Alveolata), as well as the Stramenopile *Thraustotheca clavata* and the Metazoa (RAF1 outgroup). Within the alveolates, *Toxoplasma*, *Eimeria*, *Neospora*, *Hammondia* and *Besnoitia* (Eucoccidia) cluster in one clade, as well as *Plasmodium* (Haemosporida). *Gregarina niphandrodes* forms a monophyletic clade within the alveolates. This classification reflects the molecular structure of all of the proteins displayed in Fig. 3b. Stramenopile proteins contain only one KD. All alveolate proteins contain two MORN motifs at their N-terminus (one atypical) and at least one KD at their extreme C-terminus. Eucoccidia proteins occasionally contain a SAM domain in addition to MORN motifs and a KD. *Eimeria maxima* stands out with two KDs, but nevertheless clusters with other *Eimeria* proteins. The *Plasmodium* clade members are the most complex in terms of molecular composition. In addition to the domains listed for Eucoccidia, they also contain at least one RVxF motif in their KD and a BTK-like domain upstream of the C-terminal KD. Interestingly, the *P*. *falciparum*, *P*. *praefalciparum*, *P*. *reichenowi*, *P*. *billcollinsi* and *P*. *blacklocki* sequences clustered together as previously described^46^ and stand out among all *Plasmodium* sequences given the presence of a second RVxF motif located downstream of the SAM domain. Importantly, this analysis revealed that *pTKL* is specific to certain apicomplexan species.

### The *Pf*pTKL KD binds ATP but is catalytically inactive

**Figure 4:**
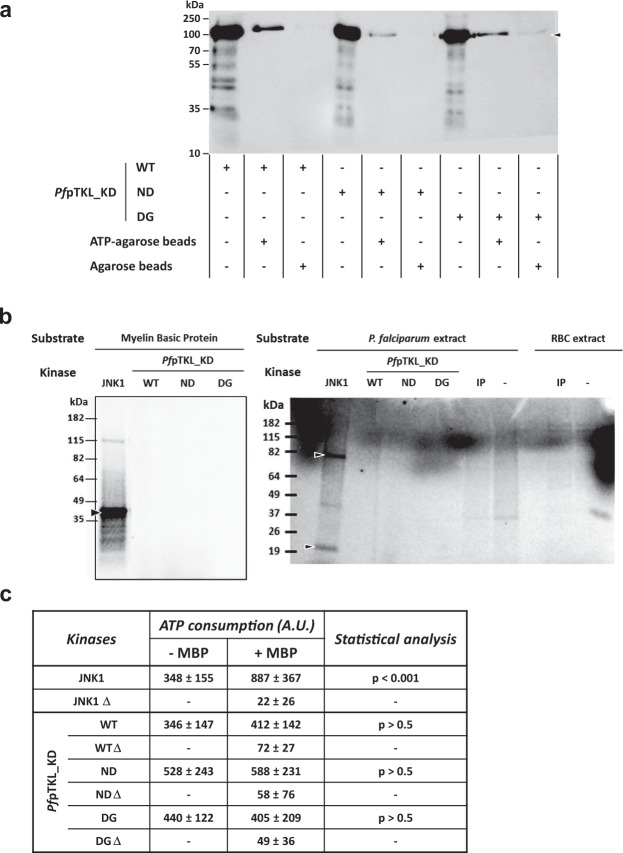
*Pf*pTKL kinase domain (pTKL_KD_WT) and its mutants (pTKL_KD_ND and pTKL_KD_DG) bind ATP but do not display any kinase activity *in vitro*. (**a**) *Plasmodium* pTKL kinase domain binds ATP. Wild-type and mutated recombinant pTKL kinase domains were incubated with ATP-agarose or agarose beads. Eluates were separated by SDS-PAGE and His_6_-tagged proteins bound to the beads were detected by western blot. (**b**) *Plasmodium* pTKL kinase domain does not catalyze kinase reactions. Kinase reactions performed with γ^33^P-ATP were stopped by the addition of loading buffer and separated by SDS-PAGE. Gels are displayed according to the substrate: myelin basic protein (left panel), *P*. *falciparum* and erythrocyte extracts (right panel). WT = wild-type recombinant kinase domain, ND = N^1272^D mutant and DG = ^1290^DFG^1292^ to ^1290^GFE^1292^ mutant. IP = immunoprecipitated *Pb*pTKL-AID-HA. JNK1 was used as an active kinase control. (**c**) *Plasmodium* pTKL kinase domain binds ATP but does not display any kinase activity. WT and mutant recombinant pTKL kinase domains were distributed in plates in the presence or absence of myelin basic protein used here as a substrate. JNK1 was used as an active kinase control and all kinases were also heated (∆) to generate negative controls. After incubation at 37 °C for 2 h, the quantity of free ATP remaining in wells was evaluated by luminometry. The ATP consumption displayed in the table corresponds to the difference between the luminometry value obtained without kinase and the luminometry value obtained with the maximum amount of kinase (80 ng/well for JNK1, and 8 µg/well for *Pf*pTKL) (mean ± SD; three biological replicates and three technical replicates per data point – raw data are available in Supplementary Table S4).

The *Pf*pTKL KD was produced as a recombinant protein named pTKL_KD_WT (Supplementary Fig. S6) and its ability to bind ATP was evaluated using a pull-down assay based on ATP-agarose beads (Fig. 4a). To determine whether the ATP-binding activity of *Pf*pTKL KD is cation dependent, the amino acid triplet ^1290^DFG^1292^ in subdomain VII was mutated to ^1290^GFE^1292^ (pTKL_KD_DG) because this motif is required for cation binding to stabilize ATP and favor phosphate group transfer. The pTKL_KD_DG mutant retained its ability to bind ATP (Fig. 4a) indicating that *Pf*pTKL belongs to the class 2 (nucleotide-only binding) pseudokinases^26^.

Next, we tested the catalytic activity of pTKL_KD_WT and pTKL_KD_DG using γ^33^P-ATP and three different substrates: myelin basic protein (MBP), *P*. *falciparum* extracts and erythrocyte extracts (Fig. 4b). The positive control JNK1 displayed kinase activity against both MBP and the *P*. *falciparum* extracts, confirming the assay was working correctly, but neither pTKL_KD_WT nor pTKL_KD_DG were active against any substrate (Fig. 4b). To determine the reason for inactivity, we mutated N^1272^ back to a canonical phosphate group-transferring subdomain VIB aspartate (pTKL_KD_ND), but this mutant remained inactive despite retaining the ability to bind ATP (Fig. 4a,b).

Finally, we conducted a luminescence assay in the presence of MBP, which confirmed the ability of JNK1 to hydrolyze ATP whereas pTKL_KD_WT, pTKL_KD_ND and pTKL_KD_DG lacked such activity (Fig. 4c, Supplementary Table S4).

Collectively, these experiments showed that the *Pf*pTKL pseudo-KD was able to bind ATP but did not display any kinase activity.

### *Pf*pTKL RVxF1 and RVxF2 bind *Pf*PP1c *in vitro* but only RVxF2 is functional in *Xenopus laevis* oocytes

**Figure 5:**
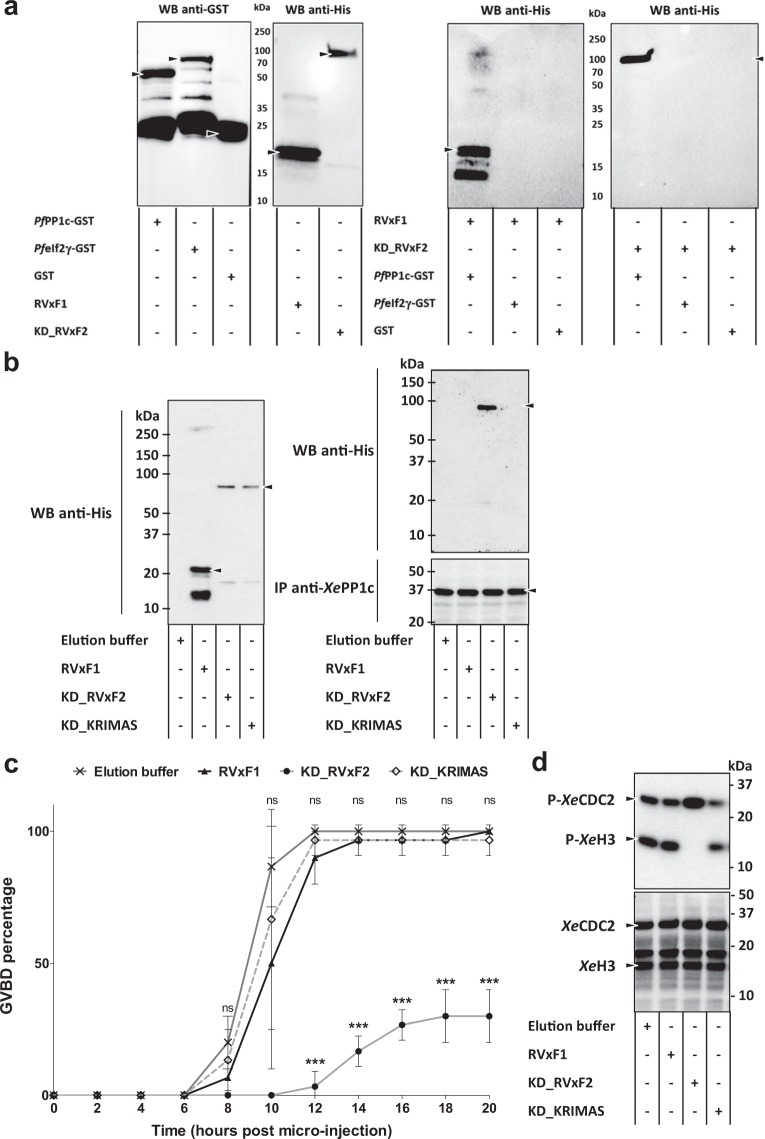
Both *Pf*pTKL RVxF1 and RVxF2 motifs bind *Pf*PP1c *in vitro* but only RVxF2 exerts a functional role in *Xenopus laevis* oocytes. (**a**) RVxF1 and RVxF2 both directly bind PfPP1c *in vitro*. *Pf*PP1c-GST, *Pf*eIf2γ-GST or GST (the last two = negative control) beads were incubated with His_6_-pTKL_RVxF1 (RVxF1) or pTKL_KD_WT (KD_RVxF2). Following washes, beads were eluted in Laemmli buffer. Proteins were separated by SDS-PAGE and His_6_-tagged proteins bound to beads were identified by western blot (WB, right panel – both blot first lanes show the specific and direct interaction of RVxF1 and KD_RVxF2 with *Pf*PP1c-GST). All inputs were analyzed by western blot as well (left panel). Uncropped blots are available in the Supplementary Information. (**b**) Only RVxF2 binds *Xe*PP1c *in vivo*. RVxF1, KD_RVxF2 and KD_KRIMAS (containing RVxF2 inactivated by mutation) were micro-injected into *X*. *laevis* oocytes. To check for the presence of RVxF-containing proteins, oocytes were lysed and His_6_-tagged proteins were separated by SDS-PAGE and detected by western blot (left panel). Oocytes micro-injected with RVxF1, KD_RVxF2 or KD_KRIMAS and incubated with progesterone were lysed 15 h after micro-injection and anti-*Xe*PP1c immunoprecipitation assays (IP) were performed. After washes, proteins bound to beads were detected by western blot (right panel). (**c**,**d**) RVxF2 inhibits progesterone-induced germinal vesicle breakdown (GVBD) in *X*. *laevis* oocytes. (**c**) Chart showing GVBD percentage over time in progesterone-incubated *X*. *laevis* oocytes micro-injected either with proteins or with control buffers (mean ± SD; three biological replicates and two technical replicates with 10 oocytes per data point). Oocyte images (external view and hemisections) are available for each sample in the Supplementary Information. ***, p < 0.001. (**d**) GVBD of oocytes micro-injected with RVxF-containing proteins was assayed by analyzing *Xe*CDC2 dephosphorylation and *Xe*H3 phosphorylation by western blot. Only KD_RVxF2 (third lane) was able to block progesterone-induced *Xe*H3 phosphorylation. Lower panel, western blot analysis of total *Xe*CDC2 and *Xe*H3. Uncropped blots are available in the Supplementary Information.

The ability of the *Pf*pTKL RVxF1 and RVxF2 motifs to bind the *Pf*PP1 catalytic subunit (*Pf*PP1c) was determined using glutathione *S*-transferase (GST) pull-down assays. A portion of *Pf*pTKL containing RVxF1 (residues 371–531) was produced as the recombinant protein His_6_-pTKL_RVxF1 (Supplementary Fig. S6) to test the first motif, and pTKL_KD_WT (containing RVxF2, as described above) was used to test the second one. *Pf*eIf2γ-GST and GST were used as negative controls^47^. Both His_6_-pTKL_RVxF1 and pTKL_KD_WT were only pulled down by *Pf*PP1c-GST beads (Fig. 5a, right panel, lanes 1 and 4).

To investigate the *Pf*pTKL–PP1c interaction in more detail, we used *Xenopus laevis* oocytes as a model system given that *X*. *laevis* and *P*. *falciparum* PP1c share 85% identity at the amino acid level. *X*. *laevis* oocytes arrested at G2/M meiotic prophase I can be used to study signaling pathways involved in cell cycle progression because the induction of meiosis with progesterone triggers germinal vesicle breakdown (GVBD). GVBD is easily detected by the rise of the nucleus to the oocyte surface (visible as a white maturation spot in the animal hemisphere), *Xe*CDC2 dephosphorylation (necessary for the activation of downstream pathways) and histone H3 (*Xe*H3) phosphorylation, an indicator of chromosome condensation^48^. Previously, we showed how *X*. *laevis* oocytes could be used to investigate the function of *P*. *falciparum* PP1c regulators: *Xe*PP1c contributes to inhibit GVBD, so the micro-injection of oocytes with a PP1c inhibitor triggers GVBD whereas the introduction of an activator prevents progesterone-induced GVBD^49^.

Accordingly, we injected *X*. *laevis* oocytes with recombinant His_6_-pTKL_RVxF1 and pTKL_KD_WT followed by co-immunoprecipitation assays to determine their *Xe*PP1c-binding activity. Only pTKL_KD_WT was able to bind *Xe*PP1c (Fig. 5b). Interestingly, pTKL_KD_WT did not trigger GVBD in untreated arrested oocytes, showing that it may not be a direct *Xe*PP1c inhibitor (Supplementary Fig. S9a), but it was able to block GVBD in the presence of progesterone in 60–70% of the oocytes. The micro-injection of His_6_-pTKL_RVxF1 had no effect, similar to the protein elution buffer used as a negative control (Fig. 5c). This led us to the conclusion that pTKL_KD_WT is probably a PP1c activator, which was supported by the relatively high phosphorylation of *Xe*CDC2 and the absence of phosphorylation of *Xe*H3 (Fig. 5d). To examine the contribution of RVxF2 in more detail, we produced the recombinant protein pTKL_KD_KRIMAS in which the RVxF2 motif ^1216^KRKVLF^1221^ was mutated to ^1216^KRIMAS^1221^ to reduce its ability to bind PP1c^50^ (Supplementary Fig. S6). We found that pTKL_KD_KRIMAS was unable to bind *Xe*PP1c and its micro-injection did not affect progesterone-induced GVBD (Fig. 5b–d).

These experiments showed that *Pf*pTKL was able to bind and modulate PP1c via the RVxF2 motif, whereas the RVxF1 motif-containing region bound PP1c *in vitro* but did not modulate its activity.

### The *Pf*pTKL SAM domain interacts with *Pf*SERA5

**Figure 6:**
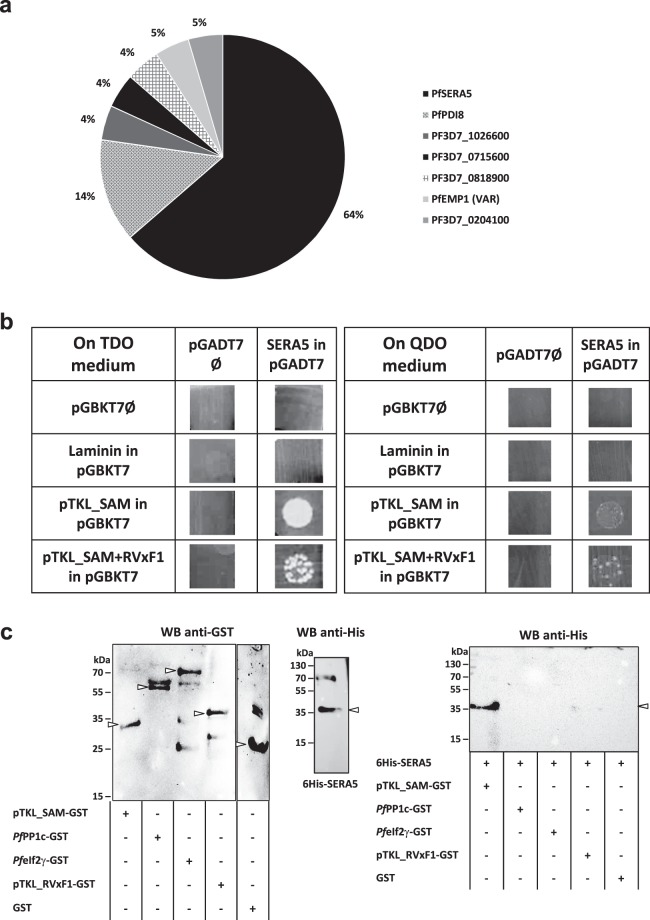
*Pf*pTKL SAM domain interacts specifically with *Pf*SERA5. (**a**) *Pf*SERA5 is the main binding partner of the *Pf*pTKL SAM. *P*. *falciparum* proteins interacting with the *Pf*pTKL SAM + RVxF1 region were identified by yeast two-hybrid (Y2H) screening. The percentage occurrence of each interacting protein is plotted for clones in frame with GAL4AD. All the hits returned by the screening are available in Supplementary Table S5. (**b**) *Pf*SERA5 interacts strongly and specifically with the *Pf*pTKL SAM in yeast. The chart shows the results of the mating run between Y2Hgold cells transformed with pGADT7 (either empty (Ø) or cloned with the longest *Pf*SERA5 cDNA sequenced during Y2H screening) and Y187 cells transformed with pGBKT7 (empty (Ø), encoding laminin as a negative interaction control or SAM + RVxF-1 or SAM only). Diploids were either streaked on TDO/A medium (left panel) or QDO/A medium (right panel). (**c**) *Pf*SERA5 interacts directly with the *Pf*pTKL SAM *in vitro*. His_6_-tagged *Pf*SERA5 (His_6_-SERA5) was incubated with pTKL_SAM-GST, pTKL_RVxF1-GST, *Pf*eIf2γ-GST, *Pf*PP1c-GST or GST (the last three = negative controls). After washes, proteins were eluted in Laemmli buffer, separated by SDS-PAGE and detected by western blot (WB, right panel). Inputs were also detected by western blot (left and middle panels). His_6_-tagged *Pf*SERA5 binds directly to pTKL_SAM-GST (first lane on right). Uncropped blots are available in the Supplementary Information.

*In silico* analysis of the *Pf*pTKL SAM domain showed that it might be involved in protein–protein interactions (Fig. 2e). To identify potential interaction partners, we screened a *P*. *falciparum* cDNA library using a yeast two-hybrid (Y2H) assay with the 838–1594 bp portion of *Pf*pTKL cDNA as the bait, corresponding to the SAM and RVxF1. We found that 32 clones of the 6000 screened were able to grow on stringent media, indicating strong interactions with the *Pf*pTKL SAM + RVxF1 region. These represented 12 *P*. *falciparum* proteins (Supplementary Table S5). Interestingly, *Pf*SERA5 (PF3D7_0207600) was detected in 64% of the in-frame screening hits (Fig. 6a). *Pf*SERA5 is the most abundant pseudo-protease in the parasitophorous vacuole^51^. To confirm this interaction, a Y2Hgold strain transformed with pGADT7 (either empty or encoding the longest *Pf*SERA5 fragment identified during the Y2H screen) was mated to a Y187 strain transformed with pGBKT7 (empty or encoding the interaction negative control laminin, SAM + RVXF1 or SAM alone). The only diploids that could grow on stringent media were those expressing both *Pf*SERA5 and at least the *Pf*pTKL SAM domain, thus excluding RVxF1 as a requirement for *Pf*SERA5 binding (Fig. 6b). These data confirm the strong and specific interaction between *Pf*SERA5 and the SAM domain of *Pf*pTKL.

The GST pull-down assay described above was repeated using *Pf*SERA5-His_6_ as the bait for *Pf*pTKL_SAM-GST and *Pf*pTKL_RVxF1-GST beads, with unrelated GST-tagged proteins as negative controls (Supplementary Fig. S6). *Pf*SERA5-His_6_ alone remained bound to *Pf*pTKL_SAM-GST beads, confirming the binding specificity between the pseudo-protease and the *Pf*pTKL SAM domain (Fig. 6c, right panel, lane 1).

### *Pb*pTKL is exported in infected host erythrocytes

**Figure 7:**
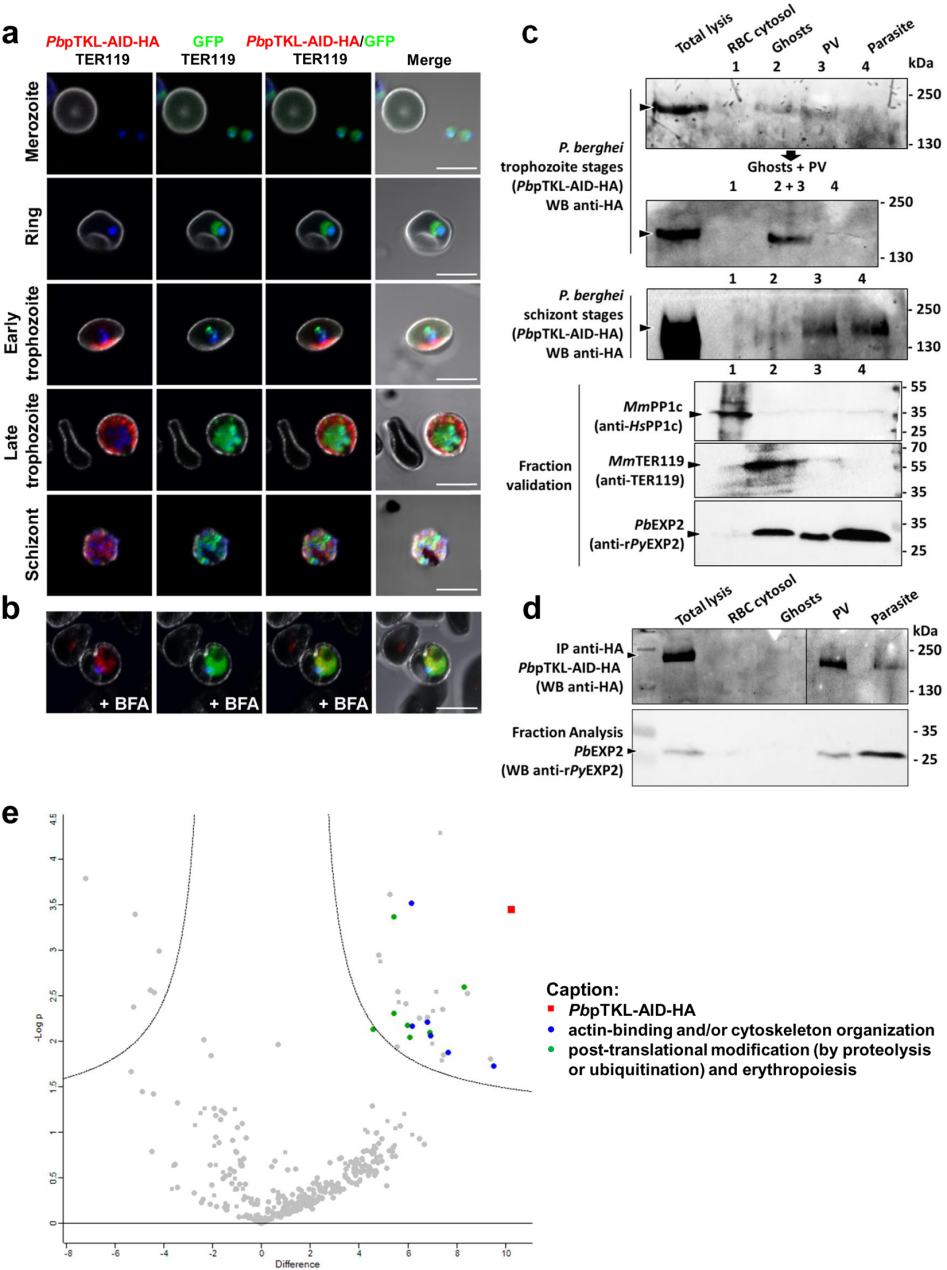
*Pb*pTKL is exported in the erythrocyte during the trophozoite stages and binds host erythrocyte proteins. (**a**,**b**) *Pb*pTKL is actively exported from the parasite between the trophozoite and schizont stages. (**a**) Confocal microscopy images showing the localization of the *Pb*pTKL-AID-HA protein (red) in infected erythrocytes at different stages (top to bottom: merozoite, ring, early trophozoite, late trophozoite and schizonts). Parasite nuclei are stained with Hoechst (blue). The erythrocyte membrane is labeled after detection of TER119 (white). Bar = 5 µm. (**b**) Confocal microscopy images showing the localization of *Pb*pTKL-AID-HA in brefeldin A (+BFA) treated trophozoites. *Pb*pTKL-AID-HA (red, BFA aggregates) is no longer exported but retained within the parasite cytosol (GFP signal). Bar = 5 µm. (**c**) *Pb*pTKL associates with ghosts during the trophozoite stages based on sequential lysis results (Supplementary Fig. S8). Fractions were verified by western blot (WB): anti-*Hs*PP1c recognizes mouse PP1c in the erythrocyte cytosol, anti-TER119 recognizes mouse erythrocyte ghosts, and anti-r*Py*EXP2 recognizes *Pb*EXP2 in the parasite cytosol, parasitophorous vacuole and vesicles in the host erythrocyte cytosol^119^. Anti-HA immunoprecipitation assays were performed on each fraction and *Pb*pTKL-AID-HA was detected by anti-HA western blot. Uncropped blots are provided in the Supplementary Information. (**d**) *Pb*pTKL is actively exported to the host erythrocyte cytosol. Sequential lysis on *P*. *berghei* trophozoites treated with BFA (verified by fraction analysis using anti-r*Py*EXP2, as opposed to fraction analysis without treatment shown in (**c**). Anti-HA IP assays were performed on each fraction and *Pb*pTKL-AID-HA was detected by anti-HA western blot. Uncropped blots can be found in the Supplementary Information. (**e**) Exported *Pb*pTKL binds erythrocyte proteins. Volcano plot showing proteins identified by mass spectrometry after anti-HA immunoprecipitation assays on *Pb*pTKL-AID-HA versus wild-type pG230 *P*. *berghei*-infected erythrocytes (*y*-axis shows negative log p-value, *x*-axis shows fold-change between wild-type and *Pb*pTKL-AID-HA samples). Squares = *P*. *berghei* proteins; circles = mouse proteins. Proteins absent from wild-type samples and detected in at least two of three *Pb*pTKL-AID-HA ghost samples are shown on the upper right panel of the chart (see caption). Three biological replicates were analyzed per sample type (raw and interactome mouse protein analysis data are available in Supplementary Table S6).

Following unsuccessful attempts to knock-in *pTKL* in *P*. *falciparum*, we generated a *P*. *berghei* line expressing an auxin-induced degradation–hemagglutinin (AID-HA)-tagged *Pb*pTKL to study the subcellular localization of pTKL throughout the asexual life stages (Supplementary Fig. S7a). *Pf*pTKL and *Pb*pTKL share a global 36% identity and a protein domain conservation of 50–70% (Supporting Information). The only difference between the two orthologs is the absence of RVxF1 in *P*. *berghei*, but as described above this sequence does not appear to be required for PP1c modulation (Fig. 5). Immunofluorescence assays using synchronized intra-erythrocytic stages of the *Pb*pTKL-AID-HA line revealed that *Pb*pTKL was exported within the infected erythrocytes and was not detected in the parasite from the early to late trophozoite stages (Fig. 7a). Small amounts of *Pb*pTKL were secreted during the early trophozoite stages, but from the late trophozoite to schizont stages the protein filled the entire erythrocyte cytosol, and probably also the parasitophorous vacuole (Fig. 7a). The active export of *Pb*pTKL within the infected erythrocytes was further confirmed by treating *Pb*pTKL-AID-HA trophozoites with brefeldin A (BFA), described as an inhibitor of protein transport^52^. In BFA-treated trophozoites, *Pb*pTKL was solely detected within the parasite cytosol as expected (Fig. 7b,d). Given that pTKL lacks a PEXEL motif and a predicted signal peptide, this also indicates that *Pb*pTKL is a PEXEL-negative protein (Table 1).

To confirm these observations, the sequential lysis of infected erythrocytes was performed on trophozoites and schizonts, followed by anti-HA immunoprecipitation (Supplementary Fig. S8). During the trophozoite stages, *Pb*pTKL-AID-HA was detected in the parasitophorous vacuole fraction and was also associated with erythrocyte ghosts. However, it was not detected in the infected erythrocyte cytosolic fraction, suggesting that the protein may be associated only with the erythrocyte membrane and cytoskeleton. During the schizont stages, *Pb*pTKL-AID-HA was detected in both the parasite and the parasitophorous vacuole fractions, but was no longer associated with erythrocyte ghosts (Fig. 7c).

### *Pb*pTKL interactome in trophozoite-infected erythrocytes

To determine whether the exported *Pb*pTKL interacts with erythrocyte proteins, we screened for interaction partners in trophozoite-infected mouse erythrocytes using anti-HA immunoprecipitation. We screened the total lysates of erythrocytes infected with a wild-type parental *P*. *berghei* line and ghost lysates of erythrocytes infected with the *P*. *berghei* line expressing *Pb*pTKL-AID-HA. Proteins that remained bound to the anti-HA beads were analyzed by mass spectrometry, and those absent in the wild-type samples but present in at least two ghost samples from three experiments were selected. The resulting list of 23 mouse proteins (Fig. 7e; Supplementary Table S6) was assessed for cellular functions using the Uniprot database^53^, GO annotation and interactome data available in the STRING database^54^. Six of the proteins were found to be involved in the organization or dynamic modulation of the actin cytoskeleton, in agreement with the localization data presented above (Fig. 7a,c). Three further proteins were found to be involved in the intracellular trafficking of ions and vesicles. Another seven proteins were members of the proteasome complex or involved in the regulation of protein ubiquitination, such as ISG15^55^. The list also included two ribosomal proteins involved in translation and two proteins involved in the physiological response to oxidative stress. The last three proteins were not considered relevant due to the subcellular localization data available on Uniprot^53^ (Supplementary Table S6). Attempts to find *Pb*pTKL interaction partners in schizonts did not lead to any exploitable data given the low expression level of *Pb*pTKL during these stages (*Pb*pTKL-AID-HA was detected in only one of three replicates).

### *Pb*pTKL is not required to complete the intra-erythrocytic cycle or gametocyte emergence

**Figure 8:**
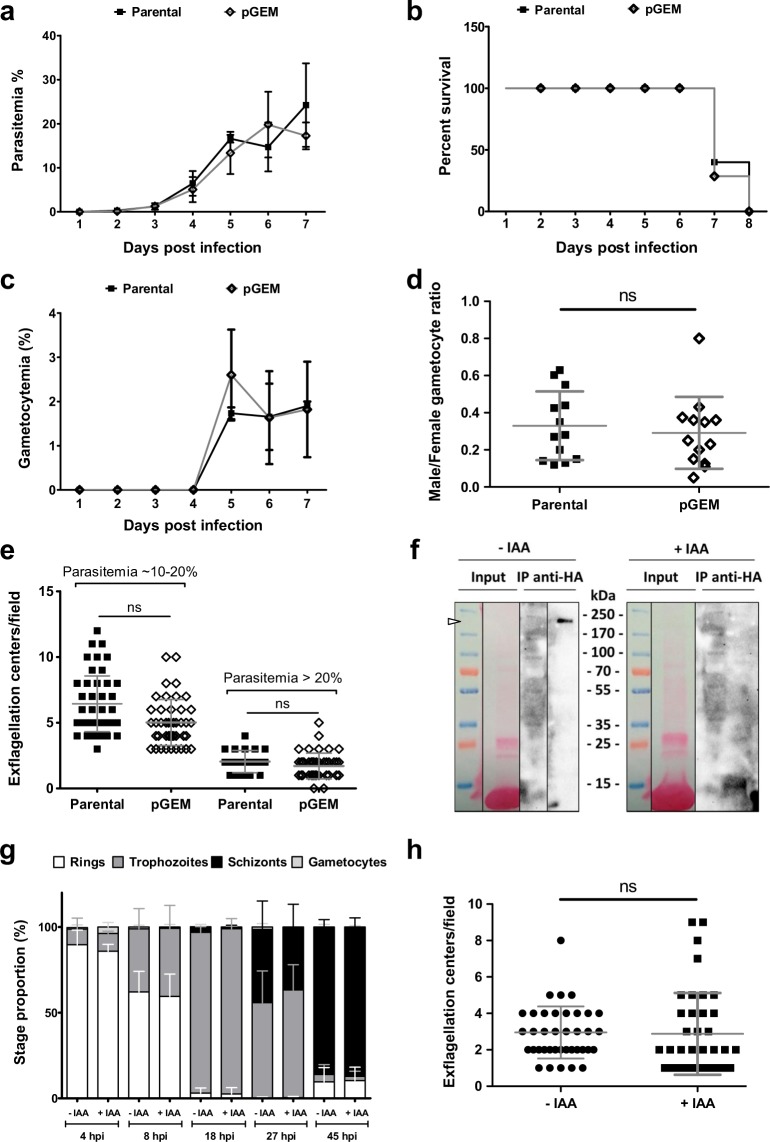
*PbpTKL* is dispensable for intra-erythrocytic cycle completion, gametocyte emergence and male gametocyte exflagellation. (**a**–**e**) Data collected using mice infected either with the wild-type *Pb*GFP strain (parental) or the PbGEM-342364-transfected strain (pGEM) – 10^6^ parasites/mouse, seven mice per group. (**a**) Knocking out *PbpTKL* does not impact parasitemia. Parasitemia follow-up in mice infected either with the parental or pGEM *P*. *berghei* parasites (mean ± SD). (**b**) Knocking out *PbpTKL* does not improve mouse survival. Percent survival of mice infected with either parental or pGEM *P*. *berghei* parasites. (**c**) Knocking out *PbpTKL* does not affect gametocyte emergence. Gametocytemia follow-up in mice infected with either with the parental or pGEM *P*. *berghei* parasites (mean ± SEM). (**d**) Knocking out *PbpTKL* does not affect male/female gametocyte ratio. Male/female ratios were evaluated in mice infected either with the parental or pGEM *P*. *berghei* parasites (data points and means ± SD are shown). (**e**) Knocking out *PbpTKL* does not affect male gametocyte exflagellation. Exflagellation center numbers were assayed for parental and pGEM parasites at a parasitemia of 10–20% and > 20% (data points and means ± SD are shown). (**f**–**h**) Data collected using cloned pG230 *P*. *berghei* parasites expressing *Pb*pTKL-AID-HA, after incubation with or without the auxin 3-idoleacetic acid (IAA). (**f**) *Pb*pTKL-AID-HA is degraded *in vitro* in the presence of IAA. *Pb*pTKL-AID-HA parasites were incubated either with (+IAA) or without IAA (−IAA). After total lysis of incubated parasites, immunoprecipitation assays (IP) were performed using anti-HA agarose beads. *Pb*pTKL-AID-HA was detected by western blot (WB) using anti-HA antibodies. Uncropped blots are available in the Supplementary Information. (**g**) Inducible knockdown of *PbpTKL* does not prevent completion of the intra-erythrocytic cycle. *In vitro* follow-up of *Pb*pTKL-AID-HA parasite stages after synchronized infection in mice (five per group) – parasites were cultured in the presence (+IAA) or absence (−IAA) of IAA, hpi = hours post-intravenous injection of Nycodenz-enriched schizonts (mean ± SD). (**h**) Inducible knockdown of *PbpTKL* does not affect male gametocyte exflagellation. Exflagellation centers were assayed for *Pb*pTKL-AID-HA parasites after incubation with (+IAA) or without IAA (−IAA) (mean ± SD). ns, no significant difference.

*PbpTKL* was recently found to be dispensable for the completion of intra-erythrocytic development^56^. This result was thus in conflict with the systematic functional analysis of the *P*. *berghei* kinome^19^. Both studies were based on similar approaches (i.e., gene disruption by double homologous recombination), but differed mainly in the length of the cloned homology regions, which favor recombination during PlasmoGEM screening^56^. We therefore attempted to generate a *pTKL-*knockout *P*. *berghei* line (pGEM) by introducing the readily available PlasmoGEM pTKL construct (PbGEM-342364) into the *Pb*GFP ANKA strain (Supplementary Fig. S7b). After selection, pyrimethamine-resistant parasites were cloned and analyzed by genomic PCR to confirm the correct recombination events (Supplementary Fig. S7b). However, both the 5′ end of the *PbpTKL* transcript (amplified using primers F_5′_/GW2, including the MORN domains) and its 3′ end (amplified using primers F28/R29, potentially including at least the KD) were detected by RT-PCR (Supplementary Fig. S7b). Furthermore, primers GW1/GT did not detect the *dhfr* resistance cassette, thus excluding the expression of a chimeric transcript comprising the *PbpTKL* 5′ region, resistance cassette and *PbpTKL* 3′ region. Phenotypic analysis of *PbpTKL* knockout parasites showed identical parasitemia and survival rates compared to the wild-type parental strain (Fig. 8a,b). Furthermore, there was no difference between the lines in terms of gametocyte production, male/female gametocyte ratio and male gametocyte activation (exflagellation) (Fig. 8c–e). Because we could not exclude the possibility that the lack of phenotype reflected the partial expression of *PbpTKL*, we used the AID-HA knock-in approach^57^ in a TIR1-expressing *P*. *berghei* ANKA pG230 strain. Clones were confirmed by diagnostic PCR (Supplementary Fig. S7a), and the degradation of *Pb*pTKL-AID-HA following the incubation of parasites *in vitro* with auxin was confirmed by western blot (Fig. 8f). The intra-erythrocytic cycle was completed and male gametocyte exflagellation occurred similarly in the parasite cultures in the presence or absence of auxin (Fig. 8h).

Together, these data show that *PbpTKL* is probably not required to complete the intra-erythrocytic cycle or to support gametocyte production and male gametocyte activation.

## Discussion

The *Plasmodium* kinome is mainly composed of S/T kinases with key roles in parasite development^20^. Although the multiple roles of pseudokinases are well known in higher eukaryotic cells^28–36^, little is known about apicomplexan pseudokinases. We therefore characterized pTKL, a pseudokinase specific to certain apicomplexan parasites, including *Plasmodium* species. Our results demonstrated that in *P*. *berghei*, *Pb*pTKL is expressed mostly during the trophozoite stages and the protein is exported to the host cell where it associates with ghosts, i.e. the erythrocyte membrane and cytoskeleton^58^. During the schizont stages, *Pb*pTKL is retained within the parasite and parasitophorous vacuole. Because *Pb*pTKL is PEXEL-negative and lacks a signal peptide as well as a transmembrane domain, its export mechanism remains unclear. We propose that pTKL crosses the parasite plasma membrane using its hydrophobic MORN motifs. Either both of them or mostly MORN2 which contains a putative N-myristoylation site could also allow the protein to associate with erythrocytic ghosts. This is further supported by the fact that MORN motifs bind to membranes and cytoskeleton (Table 1). Importantly, our interactome analysis revealed that *Pb*pTKL interacts with actin cytoskeleton-binding proteins (Fig. 7e). *Pb*pTKL may therefore be involved in the remodeling of the erythrocyte cytoskeleton or membrane, as shown for other unessential exported proteins such as *Pf*FIKK7.1 and *Pf*FIKK12^59^, *Pb*SMAC^60^ and *Pb*RESA^61^.

When exported to the host erythrocyte cytosol, *Pb*pTKL was found to reside at the crosstalk of two main protein networks. The first is composed of three proteins involved in cellular trafficking, and the second regulates protein expression and degradation (the seven members of this network include ribosomal proteins, regulators of ubiquitination and proteasome components). These proteins are classically found in erythrocyte ghost samples^58^. They are involved in the last steps of erythropoiesis, during which hemoglobin synthesis increases while other proteins, including ribosomal proteins, are degraded. This process ensures the simplification of the proteome as cells mature from reticulocytes to erythrocytes, in which 98% of all protein molecules are globin^62,63^. Two proteins involved in oxidative stress regulation were also detected in the *Pb*pTKL ghost interactome. Such proteins play a key role in maintaining erythrocytes by protecting them from the continuous oxidative stress they experience due to their oxygen transport role^64^. *Pb*pTKL might thus favor the implementation of host–pathogen communication pathways as already described for many classical code-breaking pseudo-kinases and truncated kinases^65^.

From a biochemical point of view, the *Pf*pTKL catalytic site can bind ATP in a cation-independent manner, confirming that it belongs to the class 2 pseudokinases^26^. However, the *Pf*pTKL KD was catalytically inactive under our experimental conditions, probably due to the lack of canonical subdomains I-III and VI which usually play key roles in ATP stabilization and γ-phosphate group transfer^25^. Notably, the native *Pb*pTKL-AID-HA protein also lacked catalytic activity (Fig. 4b). The role of the BTK-like domain and potentially of the SAM domain in terms of regulation of KD activity (as shown for *Pf* TKL3^66^) remains unclear. However, in addition to interaction data collected from ghost samples, the ability of the pTKL KD to bind ATP could turn the protein into an enzyme cofactor within the erythrocyte cytosol. Furthermore, ATP is an important messenger for erythrocytes^67^: it is released during hypoxia, oxidative stress or infection by *Plasmodium* species and may therefore mediate communication between infected cells and uninfected bystander erythrocytes^68^. The parasite may export an ATP-binding protein and anchor this to the host cell cytoskeleton as a strategy to modulate ATP release during its replication.

In parallel, we showed that *Pf*pTKL interacts with two parasite proteins *in vitro* and in heterologous models. The RVxF motifs of *Pf*pTKL can bind *Pf*PP1c, the major phosphatase of this parasite^69,70^. Interaction studies in the *X*. *laevis* oocyte model showed that only RVxF2 binds *Xe*PP1c *in vitro* and also influences GVBD. This suggests that RVxF1 may interact transiently with PP1c *in vivo* to bring pTKL and PP1c closer, probably only during the schizont stages where both pTKL and PP1c are localized in the parasite. RVxF2, which is conserved in all pTKL homologs across the *Plasmodium* genus, appears to bind PP1c more strongly than RVxF1 and also allows the allosteric modulation of phosphatase activity.

Using a Y2H assay, we found that the *Pf*pTKL SAM domain binds strongly and specifically to *Pf*SERA5, an abundant parasitophorous vacuole pseudo-protease that is expressed during the *P*. *falciparum* schizont stages and is involved in the egress of merozoites^51^. *Pf*SERA5 does not appear to be conserved in *P*. *berghei*, so the *Pb*pTKL SAM domain may interact with another member of the SERA family in this species. Although SAM domains feature simple and uniform primary, secondary and tertiary structures, they can bind to different proteins and nucleic acids^71,72^. The *Pb*pTKL SAM domain may therefore interact with other proteins when it is exported to the host cell cytosol. Although we could not confirm an interaction between *Pb*pTKL and *Pb*PP1c or a *Pb*SERA family member in schizonts due to the low *Pb*pTKL expression level, we found a strong correlation between the interactions identified *in vitro* and the localization of the protein and its partners in the same compartments during the schizont stages.

Having tested the catalytic and scaffolding roles of pTKL, we next investigated whether the protein is required during the *P*. *berghei* blood stages. The phenotypic analysis of PbGEM-342364-transfected *P*. *berghei* and the use of the AID knockdown system led us to the conclusion that *PbpTKL* is probably not required for the completion of the intra-erythrocytic cycle or for gametocyte emergence, in agreement with a recent saturation mutagenesis study in *P*. *falciparum*^73^. Thirteen piggyBac transposons could be inserted within the *PfpTKL* locus and surrounding intergenic regions without affecting *P*. *falciparum* intra-erythrocytic development, suggesting the protein is dispensable for blood stage parasites. However, we cannot exclude the possibility that the *Pb*pTKL MORN motifs or KD may continue to be expressed in the knockout line. Although we also used the *in vitro* AID system, which is useful to determine whether proteins are essential for invasion, cell cycle completion or gametocyte activation^57^, it may not be suitable for the analysis of proteins that are rapidly exported to the parasitophorous vacuole or the host cell after their synthesis, even though we observed that *Pb*pTKL degradation was induced in trophozoites in the presence of auxin.

In conclusion, we have shown that the *Plasmodium* pseudo-kinase pTKL can bind ATP and acts as a protein scaffold at the host–pathogen interface. Although probably unnecessary during the blood stages, it remains to be demonstrated whether this protein is required during the mosquito and/or liver stages of parasite development. Indeed, a recent single-cell transcriptomics study showed that *Pb*pTKL is strongly transcribed during the *Plasmodium* liver stages (Malaria Cell Atlas^74^).

## Materials and Methods

### Materials

Commercial plasmids were sourced from Invitrogen (pCR2.1-TOPO), Novagen (pETDuet-1), GE Life Sciences (pGEX-6P3), Qiagen (pMALpIII), and Clontech (pGBKT7 and pGADT7), whereas pG362 and PbGEM-342364 were kindly provided by Dr N. Philip (The University of Edinburgh, Edinburgh, UK) and the Plasmogem-Wellcome Sanger Institute (Cambridge, UK), respectively. The *P*. *falciparum* cDNA bank used for Y2H library preparation was described in an earlier study^75^.

Primary antibodies were sourced from Invitrogen (anti-His_6_), Thermo Fisher Scientific (anti-GST), StemCell (TER119) and Roche (rat anti-HA and anti-GFP). Anti-*Hs*PP1c (clone E9) and anti-*Xe*PP1c were provided by Santa Cruz Biotechnology. Rabbit anti-HA, anti-Cdc2, anti-phospho-Cdc2 (Tyr15) and anti-phospho-histone H3 (Ser10) polyclonal antibodies were provided by Cell Signaling Technology. Anti-histone H3 was provided by Novus Biological. Secondary highly-cross-adsorbed antibodies were obtained from Invitrogen. Anti-r*Py*EXP2 was a kind gift from Dr J. Burns (Drexel University College of Medicine, Philadelphia, USA).

Equinatoxin II (EQT) was a kind gift from Dr G. Anderluh (National Institute of Chemistry, Ljubljana, Slovenia)^76^.

### Transcript sequencing and sequence confirmation

*Pf*3D7 total RNA was extracted from saponin-lysed parasites using TRIzol (Thermo Fisher Scientific) and reverse transcribed into cDNA using the SuperScript III First-Strand Synthesis Super Mix kit (Invitrogen) following the manufacturer’s instructions. The cDNA was used to amplify and clone overlapping regions of the PF3D7_1106800 (*Pf*pTKL) transcript into vector pCR2.1-TOPO using primers F1–R12 (Supplementary Fig. S1 and Table S1). Following the transformation of TOP10 bacteria and clone sequencing, 6–12 sequences per region were compiled into one consensus covering the whole *Pf*pTKL transcript using SeqMan for comparison with PlasmoDB data^43^.

### Protein annotation

The *Pf*pTKL protein sequence was analyzed using SMART^77^, CATH^78^, Interpro^79^ and ScanProsite^80^. The tertiary structure was determined using the prediction tools Phyre2 Server^81^, M4T^82^ and RAPTOR^83^ (all well ranked by CAMEO^84,85^). RVxF motifs were identified based on two consensus sequences^86,87^ (Fig. 2b) and the PlasmoDB Protein Motif Pattern tool. Low-complexity regions and putative post-translational modifications were predicted using ScanProsite^80^. Signal peptides were predicted using SignalHmmS^88^.

### Phylogenetic analysis

We identified 48 *Pf*pTKL (PF3D7_1106800) homologs via back-and-forth PSI-BLAST analysis, first using the *Pf*pTKL sequence as a query, and then using PSI-BLAST results as queries against the *Pf*3D7 proteome. Candidates were confirmed as PF3D7_1106800 homologs when pTKL was the highest scoring hit (except for the RAF1 outgroup). Verified sequences were then retrieved from PlasmoDB^43^ or Uniprot^53^ for non-*Plasmodium* proteins before inclusion in the phylogenetic analysis. The 128 SAM amino acid sequences were retrieved from Uniprot^53^.

For each phylogenetic analysis, sequences were aligned using MAFFT^89^ (Supplementary Information) and evolutionary history was inferred using the maximum likelihood method based on the JTT matrix model in Molecular Evolutionary Genetics Analysis v6 (MEGA6)^90,91^. All positions with less than 80% site coverage were eliminated, leaving 598 positions in the final *Pf*pTKL dataset and 64 in the final SAM dataset. The initial tree for the heuristic search was obtained by applying the neighbor-joining method to a matrix of pairwise distances estimated using a JTT model. For *Pf*pTKL, a discrete gamma distribution was used to model evolutionary rate differences among sites. Evolutionary history was represented by the bootstrap consensus tree inferred from 1000 replicates^92^. The tree was visualized using MEGA6 and edited in Inkscape v0.92.

### Recombinant protein production and purification

GST, *Pf*PP1c-GST, *Pf*PP1c-His_6_ and *Pf*eIf2γ-GST were produced as previously described^47,93^. *Pf*pTKL_KD_WT was synthesized (Eurofins) as a re-codonized synthetic gene (synthetic gene 2 in Supplementary Information) and transferred to pMALPIII to stabilize the recombinant protein via an N-terminal fusion to the maltose binding protein^94,95^ with a C-terminal His_6_ tag. The resulting construct was introduced into the *Escherichia coli* strain JM109. The *Pf*pTKL_KD_WT construct was mutated by PCR targeted mutagenesis as previously described^96^. The N^1272^D mutant (pTKL_KD_ND) was prepared using primers F22/R23, the ^1290^GFE^1292^ mutant (pTKL_KD_DG) was prepared using primers F24/R25, and the ^1218^KRIMAS^1222^ mutant (pTKL_KD_KRIMAS) was prepared using primers F26/R27 (Supplementary Table S1).

The pTKL_RVxF1 recombinant proteins with His_6_ or GST tags were prepared by cloning *Pf*pTKL (cDNA 1114–1594 bp) in vectors pETDuet-1 and pGEX-6P3 using primers F13/R14 (Supplementary Table S1) and introducing them into *E*. *coli* strains C41 and AD494, respectively, to produce the proteins His_6_-pTKL_RVxF1 and GST-pTKL_RVxF1. The SAM domain (cDNA 838–1149 bp) was re-codonized by GenScript (synthetic gene 1 in Supplementary Information), transferred to vector pGEX-6P3 and introduced into *E*. *coli* strain AD494 (recombinant protein pTKL_SAM). The His_6_-tagged SERA5 was obtained by cloning *Pf*SERA5 (PF3D7_0207600) cDNA (432–1089 bp) directly from Y2H screening clone 3457 (Supplementary Table S5) into pETDuet-1 using primers F20/R21 (Supplementary Table S1). The resulting construct was introduced into *E*. *coli* strain BL21 (recombinant protein His_6_-*Pf*SERA5). All recombinant protein constructs were verified by sequencing (Supplementary Information).

The expression of recombinant proteins was induced at 16 °C overnight shaking at 200 rpm in the presence of 0.5 mM IPTG (and 1 mM MgSO_4_ as a kosmotrope for the pTKL_KD proteins^97^). Cells were harvested in ice-cold sonication buffer (20 mM Tris pH 7.5 or 8, 300 mM NaCl, 20 mM imidazole, 0.2 U/mL DNase, Roche cOmplete EDTA-free anti-protease cocktail, 0.02 mg/mL lysozyme, 1% Triton X-100, and 1 mM MgSO_4_ for the pTKL_KD proteins). His_6_ and GST tagged proteins were purified on Ni-NTA beads (Jena Bioscience) and glutathione beads (Sigma-Aldrich) respectively, according to the manufacturer’s instructions (Supplementary Fig. S6).

Protein concentrations were measured using the Pierce BCA Protein Assay Kit (Thermo Fisher Scientific).

### Kinase activity assay

Recombinant pTKL_KD_WT, pTKL_KD_ND and pTKL_KD_DG were purified on beads as described above. Beads were suspended in purification washing buffer (20 mM Tris pH 7.5, 300 mM NaCl, 20 mM imidazole, 1 mM MgSO_4_) and the concentration was determined using the Pierce BCA Protein Assay Kit. 400–800 µg of protein were used per test. Endogenous *Pb*pTKL-AID-HA was immunoprecipitated on HA-agarose beads as described below. Beads were washed and equilibrated in assay washing buffer (20 mM Tris pH 7.5, 1 mM DTT, 200 µM activated sodium orthovanadate, Roche cOmplete EDTA-free anti-protease cocktail). JNK1 (Sigma-Aldrich) was used as a positive control (1 µg per test). We mixed 20 µL of kinase (either beads or free protein) with 5 µg of substrate, 10 µL of 10 μCi ATP [γ-^33^P] (PerkinElmer Life Sciences) and ATP, final concentration 50 µM, in 40 µL of wash buffer. After incubation for 1 h at 37 °C, kinase reactions were stopped by adding Laemmli buffer. Samples were analyzed by SDS-PAGE. The resulting gel was fixed with 10% acetic acid and 10% methanol before drying. The incorporation of [γ-^33^P] was visualized and quantified using a PharosFX Plus Molecular Imager.

### ATP affinity assay

Recombinant kinases were dialyzed against PBS containing 1 mM DTT and 1 mM EDTA overnight at 4 °C and then assayed for ATP affinity using the ATP Affinity Test Kit (Jena Bioscience) according to the manufacturer’s protocol.

### ATP hydrolysis assay

ATP hydrolysis activity was evaluated using the Kinase Glo Luminescent Kinase assay kit (Promega). Beads prepared as described above were distributed in increasing quantities in white 96-well plates in washing buffer (20 mM Tris pH 7.5, 1 mM DTT, 200 µM activated sodium orthovanadate, Roche cOmplete EDTA-free anti-protease cocktail) to 20 µL per well. ATP buffer (washing buffer containing 3.3 µM ATP, 16.7 µg/mL MBP and 3.3 mM MgCl_2_) was then added to a final volume of 50 µL per well. JNK1 was used as a kinase positive control. After incubation at 37 °C for 2 h shaking at 54 rpm in the dark, Glo reagent equilibrated to room temperature was added (50 µL per well) and the plate was incubated shaking at 100 rpm for 10 min in the dark at room temperature before measuring the luminescence on a TECAN Spark 10 M luminometer (see Supplementary Table S4 for raw data).

### GST pull-down assay (RVxF interaction with *Pf*PP1c-GST)

GST pull-down assays were prepared as previously described^47^. Briefly, 2 μg of recombinant His_6_-pTKL_RVxF1, pTKL_KD_WT or pTKL_KD_KRIMAS was incubated with *Pf*PP1c-GST, *Pf*eIF2γ-GST (negative interaction control) or GST bound to glutathione-Sepharose beads, and 25 µg of bovine serum albumin (BSA) in binding buffer (20 mM Tris-HCl pH 7.4, 500 mM NaCl, 20 mM HEPES, 0.2 mM EDTA, 0.1% Triton X-100, 1 mM DTT, Roche cOmplete EDTA-free anti-protease cocktail, 1 mM MnCl_2_, 50 µM ZnCl_2_) for 2 h at 4 °C on a rotating wheel. After three washes, proteins were analyzed by western blot using an anti-His_6_ antibody (diluted 1:1000) or an anti-GST antibody (diluted 1:2000).

### Ethics statement for animal experimentation

Experiments were carried out in accordance with the principles of the European Community Council recommendations (86/609/EEC) for animal experimentations. All animal procedures were approved and supervised by the local Ethics Animal Committee (CEEA-75 Comité d’Ethique en Expérimentation Animale, Nord - Pas de Calais, France). The ethical approval number for protocols and procedures on mice (male CD1 (22–24 g) from Charles River Laboratories) is 00527.04. Experiments conducted on *X*. *laevis* (University of Rennes, UMS 3387) were approved likewise (CEEA 07/2010).

### *X. laevis* oocyte assays

Oocytes were micro-injected 1 h before incubation in 10 µM progesterone as previously described^96^ (although with recombinant proteins rather than synthetic mRNA). GVBD was assessed by checking for the appearance of a white spot at the surface of activated oocytes and by verifying the absence of any nucleus inside activated oocytes (hemisections of oocytes were prepared). *Xe*PP1c was immunoprecipitated 2 or 15 h after micro-injection^98^.

### Yeast two-hybrid assay

A *PfpTKL* cDNA segment including the SAM domain and RVxF-1 motif (residues 255–371) was transferred to vector pGBKT7 using primers F15/R16 (Supplementary Table S1) and introduced into Y187 competent yeast cells (Clontech) according to the manufacturer’s instructions. The resulting strain was used as a bait to screen protein interactions by mating with Y2Hgold cells transformed with the *P*. *falciparum* cDNA library in vector pGADT7^75^. Mating produces diploids that are viable on double dropout/aureobasidine (DDO/A) medium. We streaked 6000 diploids that grew on DDO/A post-mating on triple dropout/A (TDO/A) plates and then on quadruple dropout/A (QDO/A) plates, as increasing stringency allowed for interaction evaluation^75^. Only 32 diploids were able to develop on QDO/A medium (Supplementary Table S5). Plasmid DNA recovered from 31 of the 32 diploids was introduced into *E*. *coli* strain DH5α and checked for the presence of an insert by digestion with SfiI. Finally, the insert sequences were used as BLAST queries against PlasmoDB^43^ to identify putative *Pf*pTKL SAM interactors. For further Y2H assays, a Y2Hgold strain expressing the SAM alone was generated by mutating construct SAM + RVxF1_pGBKT7 using primers F17/R18.

### GST pull-down assay (SAM-GST interaction with *Pf*SERA5–6His)

The pTKL_SAM-GST, *P*fPP1-GST, *Pf*eIF2γ-GST, pTKL_RVxF1-GST or GST proteins bound to glutathione-Sepharose beads were blocked with 25 μg BSA in binding buffer (20 mM Tris-HCl pH 8.0, 500 mM NaCl, 1 mM MnCl_2_, 50 μM ZnCl_2_) for 1 h at 4 °C on a rotating wheel. We incubated 1 μg of recombinant *Pf*SERA5-His_6_ protein with blocked beads for 30 min, and bound proteins were detected as described above.

### Purification of stage-specific *P. berghei* parasites

Parasite stages were isolated as previously described^99^. Briefly, for schizonts, intra-erythrocytic parasites from mice 3 days post-infection were cultured in RPMI 1640 medium containing 25 mM HEPES, 0.4% Albumax, 0.2 mM hypoxanthine and 20 µg/mL gentamycin at 37 °C with gentle shaking (54 rpm) for 20 h. Infected erythrocytes were separated from non-infected cells on a 60% Nycodenz column (27.6% w/v Nycodenz in 5 mM Tris-HCl pH 7.20, 3 mM KCl, 0.3 mM EDTA).

### Generation of transgenic *P. berghei* parasites

*PbpTKL* was disrupted by double crossover homologous recombination following the introduction of the plasmoGEM vector PbGEM-342364 (Wellcome Sanger Institute^56^) into *Pb*GFP ANKA parasites (kind gift from O. Silvie, Université Pierre et Marie Curie, Paris, France) by transfection. The PlasmoGEM construct was linearized using NotI before transfection.

C-terminal AID-HA-tagged *Pb*pTKL strains were generated by single homologous recombination. A 1126-bp region of *Pb*pTKL starting 3664 bp downstream from the start codon and lacking a stop codon was prepared using primers F28/R29 and F31/R32 (Supplementary Table S1) and inserted upstream of the AID-HA sequence in pG362 before the transfection of *P*. *berghei* ANKA strain pG230 (both the plasmid and parasite strain were kind gifts from N. Philip (The University of Edinburgh, Edinburgh, UK). The targeting sequence was linearized using BsaBI before transfection.

Transfections were carried out as previously described^100^. Briefly, Nycodenz-enriched schizonts were electroporated with 5–10 µg of linearized DNA and immediately intravenously injected into naive mice. Transfectants were selected using pyrimethamine because the PbGEM-342364 and pG362 vectors both contain a *dhfr/ts* expression cassette (Supplementary Fig. S7). From day 1 post-infection, 10 mg/L pyrimethamine (Sigma-Aldrich) was supplied in the drinking water. Drug selection was carried out by repeated passages in mice. Transgenic parasites were cloned by limiting dilution.

### Genotypic analysis of mutants

C-terminal AID-HA-tagged *Pb*pTKL parasites were identified by diagnostic PCR using primers F30/Ext and GU533/R_3′_ (Supplementary Fig. S7a). Immunoprecipitation followed by western blot analysis confirmed the expression of a correctly-sized *Pb*pTKL-AID-HA protein. Knockout parasites were identified by diagnostic PCR as shown in Supplementary Fig. S7b. Primers GT/GW1 were used to confirm integration and primers F_5′_/R_5′_ were used to confirm the deletion of *PbpTKL*. Finally, RT-PCR was used to determine the *PbpTKL* transcription status of the mutants (Supplementary Table S1 and Fig. S7).

### Lysis of infected erythrocytes

*P*. *berghei*-infected erythrocytes directly isolated from infected mouse blood or overnight-matured schizonts, treated or not with 5 µg/mL BFA (Sigma-Aldrich) for 6–20 h, were Nycodenz-enriched. Two lysis protocols were used.

For total lysis, a 500-µL dry erythrocyte pellet was lysed in 5 mL ice-cold total lysis buffer (50 mM Tris pH 7.5, 100 mM NaCl, 1 mM DTT, 1% Triton X-100, 0.5 mM EDTA, 0.2 U/mL DNase, Roche cOmplete EDTA-free anti-protease cocktail) for 5 min. The resulting lysate was fractionated by ultracentrifugation (41,657 *g*, 4 °C, 15 min) and the supernatant was used for immunoprecipitation.

For sequential lysis, a 500-µL dry pellet was lysed in 3 mL of EQT buffer (20 mM Tris pH 7.4, 130 mM NaCl, 1 mM MgSO_4_, 6 µg/mL EQT) on wheel for 15 min at room temperature. After centrifugation, the supernatant was fractionated by ultracentrifugation as above to recover ghosts. The EQT lysate ultra-supernatant (corresponding to the erythrocyte cytosol) was kept on ice for further analysis. The ghosts were lysed in ice-cold Triton buffer (20 mM Tris pH 8.0, 50 mM NaCl, 9% Triton X-100, 5 mM EDTA, Roche cOmplete EDTA-free anti-protease cocktail). The pellet, composed of parasites embedded in their parasitophorous vacuole, was further lysed in 3 mL of 0.07% saponin (on ice for 10 min). After centrifugation, the supernatant (the parasitophorous vacuole content) was kept for further analysis while the parasite pellet was lysed in 2 mL of ice-cold Triton buffer. Saponin, parasite and ghost lysates were diluted in total lysis buffer to 5 mL and fractionated by ultracentrifugation as above prior to immunoprecipitation (Supplementary Fig. S8).

### Immunoprecipitation assay

Lysates prepared as described above were incubated for 1 h rotating on wheel at 4 °C with 20–30 µL HA-agarose beads (Invitrogen). The beads were washed three times in ice-cold washing buffer (20 mM Tris pH 7.5, 150 mM NaCl, 0.5% Triton X-100) and resuspended in Laemmli sample buffer for western blot analysis and/or mass spectrometry.

### Mass spectrometry

*Pb*pTKL-AID-HA was immunoprecipitated from ghosts during the trophozoite stages and from parasite + parasitophorous vacuole fractions during the schizont stages. Total lysis of the *P*. *berghei* pG230 strain trophozoites was followed by anti-HA immunoprecipitation to generate wild-type samples. The presence of *Pb*pTKL-AID-HA in the ghost and schizont samples but not the wild-type samples was confirmed by western blot. Three biological replicates of each sample were analyzed by mass spectrometry. The detailed NanoLC-MS/MS protein identification and quantification protocol is provided in the Supplementary Information. Raw mass spectrometry data have been deposited in the ProteomeXchange Consortium via the PRIDE partner repository^101^ with the dataset identifier PXD011986.

The MS data files were processed using MaxQuant v1.5.8.3 and searched using the Andromeda search engine against the Mus musculus database from Swissprot 07/2017 and Plasmodium berghei ANKA from PlasmoDB (v37). The PbpTKL-AID-HA chimeric protein sequence was also integrated. Parent mass and fragment ions were searched with initial mass deviations of 4.5 and 20 ppm, respectively. The minimum peptide length was set to seven amino acids and strict specificity for trypsin cleavage was required, allowing up to two missed cleavage sites. Carbamidomethylation (Cys) was set as a fixed modification, whereas oxidation (Met) and N-terminal acetylation were set as variable modifications. The false discovery rates (FDRs) at the protein and peptide level were set to 1%. Scores were calculated in MaxQuant as previously described^102^. The reverse and common contaminant hits were removed from the MaxQuant output. Proteins were quantified according to the MaxQuant label-free quantitation (LFQ) algorithm^102,103^. Matches between runs were not allowed. Wild-type and *Pb*pTKL-AID-HA IP biological replicates were analyzed using Perseus v1.6.2.3. The LFQ data were log2 transformed and proteins identified in at least two of three *Pb*pTKL-AID-HA replicates but never in the wild-type samples were considered “significant”. The data were represented on a volcano plot following the imputation of missing values by creating a Gaussian distribution of random numbers with a standard deviation of 30% relative to the standard deviation of the measured values and a 4 (wild-type) or 1.8 (*Pb*pTKL-AID-HA) standard deviation downshift of the mean.

### Phenotypic analysis of transgenic *P. berghei* parasites

Mice were infected by the intraperitoneal administration of either cryopreserved clones or infected erythrocytes from mice carrying either the wild-type or mutant parasites. Parasitemia and gametocytemia were monitored every day up to 7–8 days using Giemsa-stained blood smears. To follow the intra-erythrocytic cycle, infection was synchronized by intravenously injecting Nycodenz-enriched schizonts into mice^99^. Blood was collected by cardiac puncture after 3–4 h (typically 0.5–1.2% parasitemia was observed) and cultured at 37 °C shaking at 54 rpm for 48 h. Parasite intra-erythrocytic development was followed using Giemsa-stained smears at precise time points and compared to wild-type cultures. Exflagellation assays were performed 3–4 days after synchronized infection in mice treated with phenylhydrazine 2 days before infection (4 mg/mouse). Following the assessment of gametocytemia, 10 µL of infected blood was added to 50 µL of ookinete medium (RPMI1640 containing 25 mM HEPES and 10% fetal calf serum, pH 8)^99^ at 21 °C. Exflagellation centers were counted 15 min post-activation by light microscopy (at least 10 fields).

### Inducible knockdown of *P. berghei* pTKL

The degradation of *Pb*pTKL-AID-HA in the presence of 3-indoleacetic acid (IAA, Sigma-Aldrich) was first analyzed by western blot. Nycodenz-enriched infected erythrocytes, with or without exposure to 0.5 µM IAA for 30 min at 37 °C, were lysed (total lysis protocol) and *Pb*pTKL-AID-HA was immunoprecipitated. After confirming protein degradation in the presence of IAA, the intra-erythrocytic cycle was studied *in vitro* as described above, except that rings were cultured with or without 0.5 µM IAA^57^.

### Immunofluorescence assays and microscopy

*P*. *berghei* parasites were synchronized as described above. Infected erythrocytes were collected at each development stage and fixed (4% paraformaldehyde and 0.075% glutaraldehyde in PBS) for 10 min on ice. Following incubation on poly-L-lysine-coated coverslips overnight at 4 °C, infected erythrocytes were blocked and permeabilized (1% BSA and 0.5% Triton X-100 in PBS) for 30 min at room temperature. Coverslips were incubated with rabbit anti-HA antibodies (diluted 1:150 in 1% BSA in PBS) and TER119 (diluted 1:500 as above) for 1 h at 37 °C. Coverslips were then incubated with Hoechst 33342 diluted 1:500 (Sigma-Aldrich) and secondary highly-cross-adsorbed anti-rabbit-AF594 and anti-rat-AF647 (diluted 1:1000 as above) for 30 min at 37 °C. The samples were mounted in Mowiol 4–88 (Sigma-Aldrich). Images were obtained at room temperature with a 63 × Plan Apochromat (1.4 NA) oil objective on the LSM880 confocal microscope (Zeiss) and processed using Zen Software.

### Statistical analysis

The Mann-Whitney U test for non-parametric data was used for statistical comparisons of ATP consumption (*in vitro* Kinase Glo assay), percentages of GVBD observed in *Xenopus* oocytes and phenotypic analyses performed with *P*. *berghei* parental and pGEM lines. p < 0.001 was considered significant.

## Supplementary information

Supplementary Information

Table S6

## Data Availability

The authors confirm that the data supporting the findings of this study are available within the article and its supplementary materials. Raw mass spectrometry data are openly available on PRIDE repository (ProteomeXchange Consortium, PXD011986). Data that support the findings of this study are available from the corresponding author CP upon reasonable request.
