# Supplementary material for: Plasmodium pseudo-Tyrosine Kinase-like binds PP1 and SERA5 and is exported to host erythrocytes
Source: Sci Rep. 2019 May 31;9:8120. doi: 10.1038/s41598-019-44542-3 (PMC6544628; doi:10.1038/s41598-019-44542-3)
Supplement: Supplementary file 1 — Supplementary Information [file 41598_2019_44542_MOESM1_ESM.pdf]

Supplementary information

Plasmodium pseudo-Tyrosine Kinase-like binds PP1 and SERA5 and is exported to host erythrocytes

Bénédicte Gnangnon[1], Aline Fréville[1], Katia Cailliau[2], Catherine Leroy[3], Caroline De Witte[1], David Tulasne[3], Alain Martoriarti[2], Vincent Jung[4], Ida Chiara Guerrera[4], Sabrina Marion[1], Jamal Khalife[1], Christine Pierrot[1]*

[1] Center for Infection and Immunity of Lille, Inserm U1019-CNRS UMR 8204, Université de Lille, Institut Pasteur de Lille, 1 Rue du Professeur Calmette, F-59000 Lille, France

[2] Université de Lille, CNRS, INRA, UMR 8576-UGSF - Unité de Glycobiologie Structurale et Fonctionnelle, F-59000 Lille, France

[3] University of Lille, CNRS, Institut Pasteur de Lille, UMR 8161 - M3T - Mechanisms of Tumorigenesis and Target Therapies, F-59000 Lille, France.

[4] Proteomics platform 3P5-Necker, Université Paris Descartes - Structure Fédérative de Recherche Necker, INSERM US24/CNRS UMS3633, 14 Rue Maria Helena Vieira Da Silva, Paris 75014, France.

Table of Contents

Supplementary Table S1. Primer table. 2
Supplementary Figure S1. PF3D7_1106800 transcript sequencing data. 4
Supplementary Table S2. PF3D7_1106800 kinase domain in silico prediction. 5
Supplementary Figure S2. PF3D7_1106800 kinase domain phylogenies. 6
Supplementary Figure S3. The *Pf*pTKL BTK-like domain folds like the BTK N-terminal lobe. 7
Supplementary Figure S4. *P. falciparum* MORN motif alignment 8
Supplementary Figure S5. *Pf*pTKL SAM domain phylogenetics 9
Supplementary Table S3. *Pf*pTKL regions produced as recombinant proteins. 10
Supplementary Figure S6. Recombinant protein purification. 10
Supplementary Table S4. Raw data – Kinase Glo assays 11
Supplementary Table S5. Y2H screening results. 12
Supplementary Figure S7 - Reverse genetics construct and genotyping data. 13
Supplementary Figure S8 – Infected erythrocyte sequential lysis protocol. 15
Supplementary Figure S9. *Xenopus laevis* oocyte experiments. 16
Uncropped blots 17
NanoLC/MS protein identification and quantification (mass spectrometry) 21
Supporting Information 1 – Synthetic gene 1 – pTKL_SAM-GST 22
Supporting Information 2 – Synthetic gene 2 – pTKL_KD_WT 22
Supporting Information 3 – MAFFT Alignment of *Pf*pTKL orthologs BTK-like domains 23
Supporting Information 4 - MAFFT Alignment Kinase Domains (phylogenetics analysis) 24
Supporting Information 5 – MAFFT Alignment – *Pf*pTKL orthologs and homologs 42
Supporting Information 6 – MAFFT Alignment SAM and SAM-like domains 62
Supporting Information 7 – EMBOSS Needle alignment *Pf*pTKL vs *Pb*pTKL 65
Supporting Information 8 – Sequencing data – pTKL_RVxF1 68
Supporting Information 9 – Sequencing data pTKL_SAM&RVxF1 71
Supporting Information 10 – Sequencing data pTKL_KD_WT and mutants 74
Supporting Information 11 – Sequencing data *Pf*SERA5 recombinant protein 79
References 84

Supplementary Table S1. Primer table.

Primer	5' to 3' sequence	Use
F1	ATGGGCAACACCCTTGATAG	Amplification of PF3D7_1106800 0-1100 bp transcript portion
R2	TCTACAAGTTATGGCATATAAT	
F3	ATTGCTTATGGACATGCAATCA	Amplification of PF3D7_1106800 1040-1600 bp transcript portion
R4	AAAATATGCTATCACTACATATACC	
F5	GGTATATGTAGTGATAGCATATTTT	Amplification of PF3D7_1106800 1600-2280 bp transcript portion
R6	TCATGTATATATTGTTGCAATACT	
F7	ATAGTATATCTTCAACATGTTCTTC	Amplification of PF3D7_1106800 2150-2760 bp transcript portion
R8	ATGTAGATATGAAAAGAGACACTTA	
F9	ATGGGAGTATCTATTCATTATCC	Amplification of PF3D7_1106800 2700-3300 bp transcript portion
R10	TCGTATGCTCCACATAATACAT	
F11	CAAGTACACTTTCTGATCATAG	Amplification of PF3D7_1106800 3180-4500 bp transcript portion
R12	TTATGTTCCATCCATAAATGAAAT	
F13	GCCAGGATCCGAATTCTTCAATAACTTAATACAAATAGAAG	Cloning of cDNA encoding PF3D7_1106800$_{371\text{-}531}$ protein in pETduET to express *Pf*pTKL_RVxF1
R14	AAGCATTATGCGGCCGCTATGCTATCACTACATATACC	
F15	CATGGAGGCCGAATTCGACAAAATGAATATATCCCTTCAT	Cloning of cDNA encoding PF3D7_1106800$_{\mathbf{255\text{-}531}}$ protein (SAM+RVxF1) in pGBKT7 to express the Y2H bait in Y2Hg
R16	GCAGGTCGACGGATCCTATGCTATCACTACATATACC	
F17	ATGGCATATAATAAATAATTCAATAACTTAATA	Targeted mutagenesis primers used to insert a STOP codon in SAM+RVxF1_pGBKT7 construct
R18	TATTAAGTTATTGAATTATTTATTATATGCCAT	
F20	ACCACAGCCAGGATCCGGATACAGAAGATACTAATATCGAA	Cloning of cDNA encoding protein *Pf*SERA5$_{144\text{-}363}$ in pETduET-1
R21	CGCCGAGCTCGAATTCTACTAAAAGAGCACATTGAAAGCA	
F22	GGAGAAAATGAGCCCCATTGATCTG	Targeted mutagenesis primers used in N1272D mutation (to generate pTKL_KD_ND mutant)
R23	CAGATCAATGGGGCTCATTTTCTCC	
F24	CGAATCTCTGAATGCCAAAATTAGCGGTTTCGAAATCAGC	Targeted mutagenesis primers used in ^{1290}DFG^{1292}-^{1290}GFE^{1292} mutation (to generate pTKL_KD_DG mutant)
R25	GCTGATTTCGAAACCGCTAATTTTGGCATTCAGAGATTCG	
F26	GATCAAACGGAAACGCATAATGGCGTCTTCCTACCTGA	Targeted mutagenesis primers used to mutate RVxF2 motif (^{1216}KRKVLF^{1221} into ^{1216}KRIMAS^{1221})
R27	TCAGGTAGGAAGACGCCATTATGCGTTTCCGTTTGATC	

F28	ACCGCGGTGGCGGCCGCCGGATTTTGGAATTAAAGAAATAG	Cloning of the last 1126 bp of PBANKA_0940100 gene (STOP codon excluded) from *P. berghei* gDNA in pG362
R29	TCATTCTAGTCTCGAGTCCATCCATAAAAGACATTAAAGC	
F30	GCGTATCTAACTATTCCACTTTA	Primers used to genotype the *Pb*pTKL-AID-HA cloned parasites (Supplementary Fig. S5)
GU533	GATTAAGTTGGGTAACGCC	
Ext	CCGTCTCTGAGAACCCTCT	
$R_{3'}$	GGCGGCCGCTCTAGATTCATGATTAACAGTGTTAATTAAACA	
F31	GTAAGGCTAAATTGGATATCAATCAAATAT	Silent targeted mutagenesis primers used to add a BsaBI restriction site within pG362 *Pb*pTKL insert
R32	ATATTTGATTGATATCCAATTTAGCCTTAC	
GT	TGCCAAACTGCTATTGATGT	Primers used to genotype the *P. berghei* parasites transfected with PbGEM-342364 from Plasmogem (Supplementary Fig. S5)
GW1	CATACTAGCCATTTTATGTG	
GW2	CTTTGGTGACAGATACTAC	
$F_{5'}$	CGTGCCCGATTATGCCGGCATGGGAAATATAAATTCTATTCAAAC	
$R_{5'}$	CCAAAATTTTAACGCTAGCCGCTTAAAGCCCATTTCATAGG	

Supplementary Figure S1. PF3D7_1106800 transcript sequencing data.

a

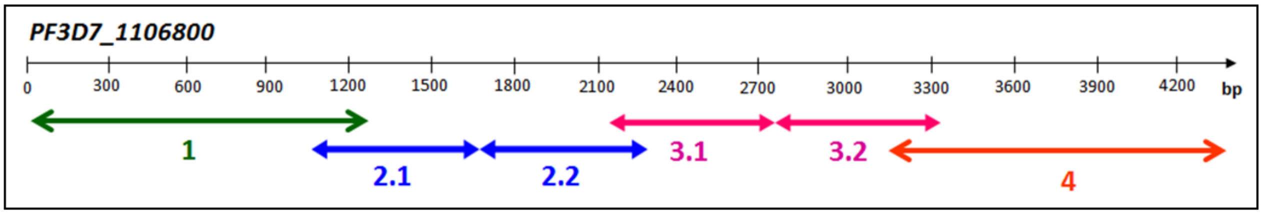

b

PlasmoDB vs our data / Gene region N°	At the gDNA level	At the cDNA	At the protein level
1 (0-1100 bp)	N/A	T531A	K177N
2.1 (1040-1600 bp)	N/A	G1218T<br>A1220T	K406N<br>K407I
2.2 (1600-2280 bp)	One 24 nucleotide gap	Various 24 (major), 96 or 138 nucleotide gaps	Various 8 (major), 32 or 46 amino acid gaps
3.1 (2150-2760 bp)	N/A	-	-
3.2 (2700-3300 bp)	N/A	-	-
4 (3180-4500 bp)	One 30 nucleotide gap	30 nucleotide gap	10 amino acid gap

c

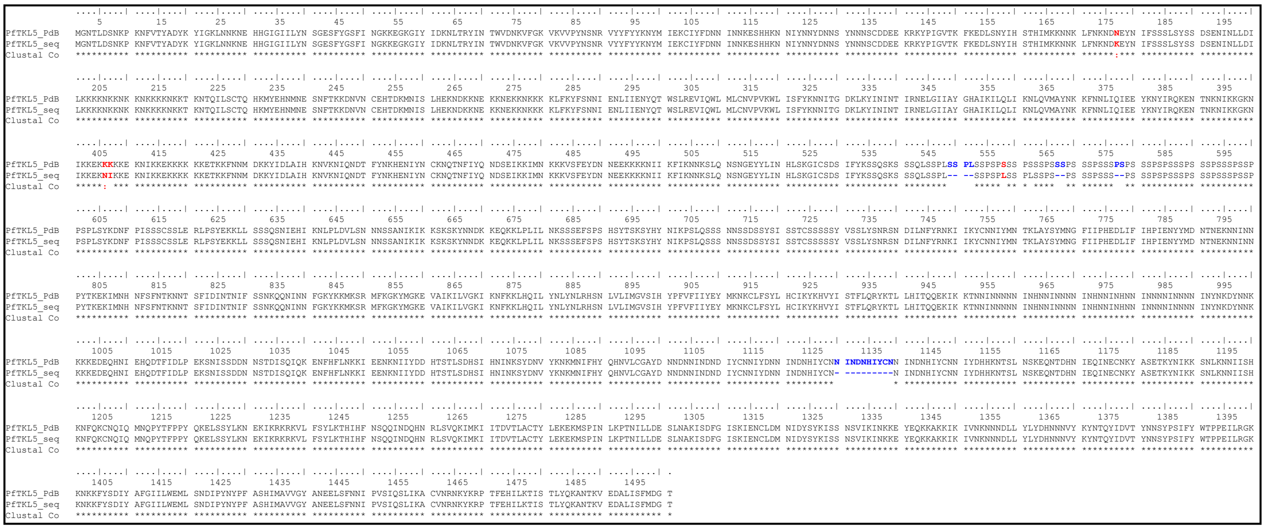

(a) Scheme of the overlapping regions in the PF3D7_1106800 transcript which were amplified by RT-PCR using total Pf3D7 RNA as the template before sequencing.

(b) Comparison of data collected by overlapping RT-PCR using total Pf3D7 RNA (or total Pf3D7 genomic DNA) and data from PlasmoDB. Two independent RT-PCRs were performed and 6–12 clones were sequenced for each transcript region.

(c) Pairwise sequence alignment of the PF3D7_1106800 PlasmoDB sequence (PfTKL5_PdB) and the translated sequenced transcript (PfTKL5_seq') using TCoffee. The mutations and gaps highlighted in Fig. 2 are shaded respectively in red and blue.

Supplementary Table S2. PF3D7_1106800 kinase domain in silico prediction.

Prediction Software	Kinase domain delineation	Prediction Confidence
SMART	1150–1463	9.51 $x10^{-8}$
Interpro	1080–1465	-
CATH	1237–1464	1.2 $x10^{-29}$
ScanProsite	1080–1465	score = 18.238
Phyre2	1144–1483	Template = IKK1<br>Prediction confidence = 100%
RAPTOR-X	1136–1483	Template = TYK2 from the JAK family ;<br>Prediction p-value = 4.56 $x10^{-11}$

Summary of primary (SMART[1], Interpro[2], CATH[3] and ScanProsite[4]) **and tertiary** (Phyre2[5] RAPTORX[6]) **structure delineations of the PF3D7_1106800 kinase domain**: Compiling these results leads to a final delineation for the kinase domain: residues 1080–1483.

Supplementary Figure S2. PF3D7_1106800 kinase domain phylogenies.

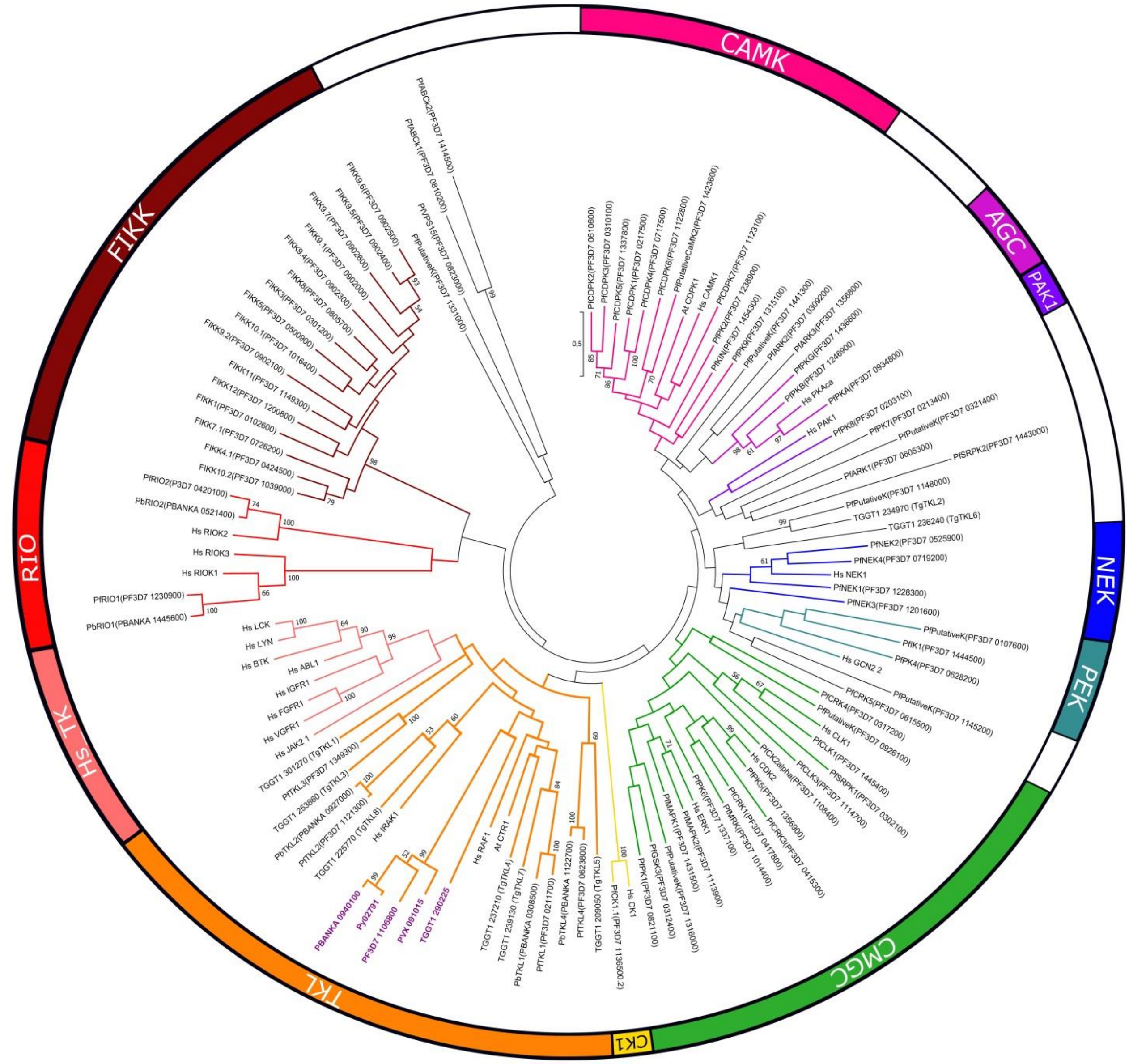

PF3D7_1106800 is a tyrosine kinase-like protein. The figure displays the classification of *Plasmodium falciparum* kinases with various reference kinases from one each of the categories Alveolata, Opisthokonta (Metazoa), and Archeaplastida (Plantae). Species names can be found from the sequence IDs: Alveolata: Pf= *Plasmodium falciparum*, Pb= *P. berghei*, PVX= *P. vivax*, Py=*P. yoelii*, TGGT1= *Toxoplasma gondii* GT1; Metazoa: Hs= *Homo sapiens*; Plantae: At= *Arabidopsis thaliana*. The 117 kinase domain amino acid sequences included in this classification were retrieved from Uniprot[7] and aligned using MAFFT[8]. The phylogenetic analysis was performed with MEGA6[9-11]. Less than 30% alignment gaps, missing data, and ambiguous bases were allowed at any position (there were a total of 229 positions in the final dataset). The tree is drawn to scale, with branch lengths measured in the number of substitutions per site. Graphical presentation and editing were achieved using MEGA6[9-11] and Inkscape 0.92.

Supplementary Figure S3. The *Pf*pTKL BTK-like domain folds like the BTK N-terminal lobe.

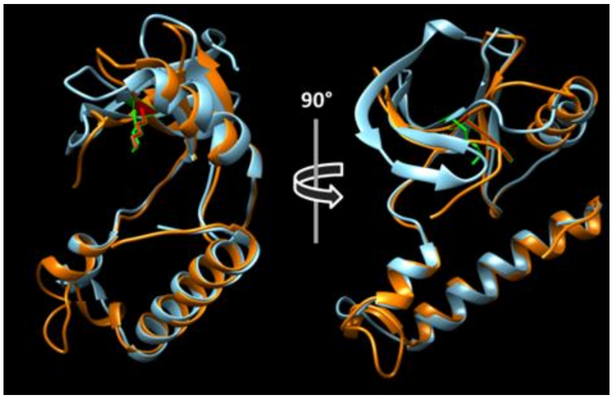

Tertiary structure comparison between *Pf*pTKL BTK-like domain shaded in orange (tertiary structure was predicted with Phyre 2[5], 120 residues (98%) modeled at >90% accuracy) and BTK N-terminal lobe shaded in gray (PDB ID: 4RFZ, residues 396–519). The ATP-binding lysine of BTK is shaded in green and the one conserved in the *Pf*pTKL BTK-like domain is shaded in red. The shading and structure comparison were achieved using the Color and MatchMaker tools of Chimera v1.10.1[12] respectively.

Supplementary Figure S4. *P. falciparum* MORN motif alignment

a

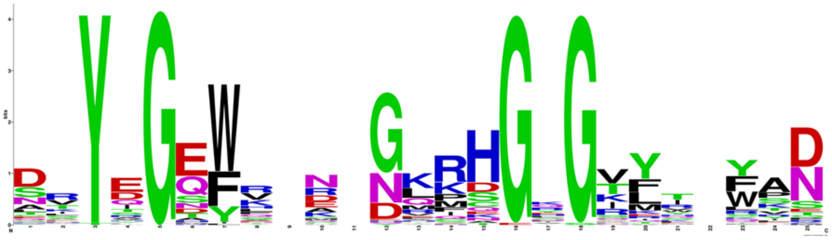

b

```
                            ....|....| ....|....| ....|
                                 5         15        25
PF3D7_0211700_1             YIGRI-ENGQ KNGWGLQINN N----
PF3D7_0211700_2             YIGTY-KKGK KHGKGILINY N----
PF3D7_0514700_1             YKGSW-KNNK RHGYGKIKLY N----
PF3D7_0514700_2             YVGFW-KKNK KWGYGKMTWI Q----
PF3D7_0514700_3             YVGQW-KNDK EEGYGKFFWF K----
PF3D7_0514700_4             YEGYW-KKGK RFGYGIFNYY N----
PF3D7_0514700_5             YIGIW-KNNK KHGYGYLINV N----
PF3D7_1031200_1             YNGNI-KDGL FHGFGILIYS Q----
PF3D7_1031200_2             YEGDF-VYGK REGRGKFTYA D----
PF3D7_1031200_3             YEGEW-VDDK IHGKGIANFV S----
PF3D7_1031200_4             YEGEW-ENGK INGFGMLCYN N----
PF3D7_1031200_5             YEGEW-LDGK MHGRGTYTYE D----
PF3D7_1031200_6             YIGEW-KNDK RHGKGCVKYK G----
PF3D7_1031200_7             YEGDW-VDGK MQGRGTYFFA D----
PF3D7_1031200_8             YEGDW-VDGK MEGKGVYKYL N----
PF3D7_1031200_9             YEGEW-INDM KNGYGTLAYV N----
PF3D7_1031200_10            YEGYW-KNDK VHGKGTLTYS K----
PF3D7_1031200_11            YIGEW-KYAK KCGEGELIYA S----
PF3D7_1031200_12            FKGQW-KNDK ANGYGILLYN N----
PF3D7_1031200_13            YEGEW-LDDH RHGMGTFTCK E----
PF3D7_1031200_14            YSGHF-QFNR KHGKGTLTFV N----
PF3D7_0926000_1             YIGTS-RNKK RNGFGYYLYV N----
PF3D7_0926000_2             YQGQW-NDNT KNGYGTLYNQ K----
PF3D7_0926000_3             YSGEW-LNNI PHGFGCNYKD G----
PF3D7_0926000_4             YMGCY-KYGF MNGVGILRKK R----
PBANKA_0940100_1            YAGDLNVDNL PHGRGLMLYE NGN--
PBANKA_0940100_2            F-GHF-INGK KHGKGIYIDK N----
PF3D7_1106800_1*            YIGKLNNKNE HHGIGIILYN SGE--
PF3D7_1106800_2*            FYGSF-INGK KEGKGIYIDK NLTRY
PF3D7_1426400_1             YEGEF-KNEK FHGEGMLIFK D----
PF3D7_1426400_2             YKGKW-ENGK FVD-GKYYFS DGLQ-
PF3D7_1349300_1             YIGET-INNI RNGWGMLIYN DRVI-
PF3D7_1349300_2             FEGEY---KM NNAIGYFIKY NEYST
```

(a) MORN domain sequence pattern. This sequence logo was prepared using MORN domain family sequences from SMART[1]. The logo was made with WebLogo[13]

(b) Alignment of various MORN domains belonging to different *Plasmodium falciparum* 3D7 proteins (MORN1 = PF3D7_1031200, MORN repeat proteins = PF3D7_0514700, PF3D7_1426400, tyrosine kinase-like proteins: *Pf*TKL1 = PF3D7_0211700, *Pf*TKL3 = PF3D7_1349300). Proteins are identified with their PlasmoDB[14] identifier and each MORN domain position in the protein (from N-terminus to C-terminus) is numbered from 1 to 14. MORN domains were predicted using SMART[1] and aligned using MAFFT[8]. A 80% threshold shading was applied to the alignment using BioEdit v7.2.5. This alignment shows that most *Plasmodium falciparum* MORN domains follow the sequence signature shown in panel (a).

Supplementary Figure S5. *Pf*pTKL SAM domain phylogenetics

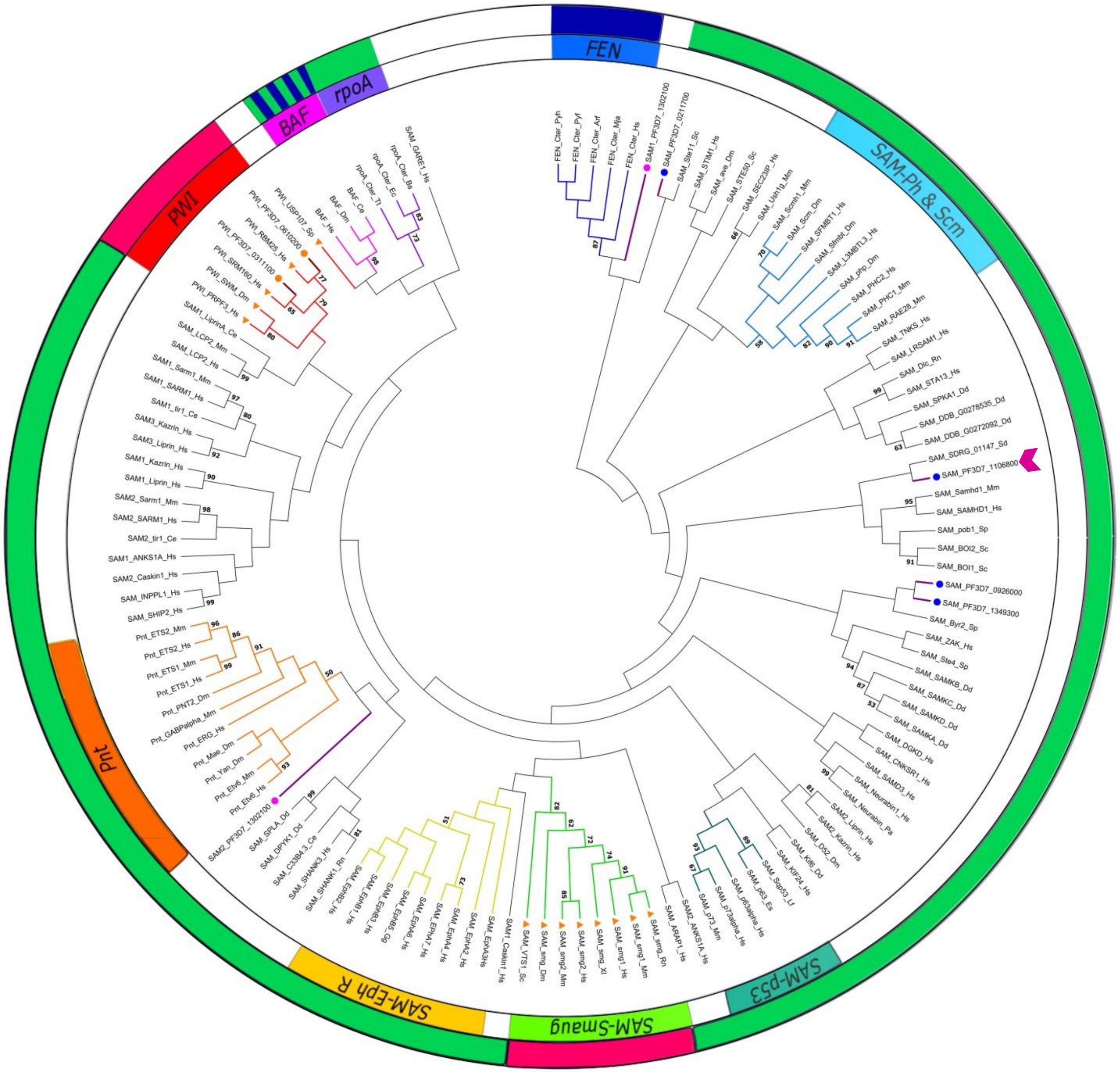

Caption :

- = interaction with DNA
- = interaction with RNA
- = interaction with proteins

*Pf*pTKL SAM clusters with SAMs involved in protein–protein interactions. The figure shows a classification of SAMs and SAM-like sequences across the tree of life. Species names can be found from sequence IDs as follows: Archae: Arf = *Archeoglobus fulgidus*, Mja = *Methanocaldococcus jannaschii*, Pyh= *Pyrococcus horikoshii*, Pyf = *P. furiosus*; Bacteria: Bs= *Bacillus subtilis*, Ec= *Escherichia coli*, Tt= *Thermus thermophilus*; Amoebozoa: Dd= *Dictyostelium discoideum*, Metazoa: Ce= *Caenorhabditis elegans*, Dm= *Drosophila melanogaster*, Es= *Euprymna scolopus* (Hawaii bobtail squid), Hs= *Homo sapiens*, Lf= *Loligo forbesi* (Northern European squid), Mm= *Mus musculus*, Pa= *Papio anubis*, Rn= *Rattus norvegicus*, Xl= *Xenopus laevis*; Fungi: Sc= *Saccharomyces cerevisiae*, Sp= *Schizosaccharomyces pombe*; Stramenopile: Sd= *Saprolegnia diclina*; Alveolata: *Plasmodium falciparum* 3D7 proteins are identified with their PlasmoDB ID: PF3D7_xxxxxxx.

Supplementary Table S3. *Pf*pTKL regions produced as recombinant proteins.

Recombinant protein name	*Pf*TKL protein region (AA)	Tags	Remarks
pTKL_RVxF1	371-531	His_6 (3') or GST (5')	-
pTKL_SAM	279-383	GST (5')	Synthetic gene 1
pTKL_KD_WT	1076-1435	MaBP (5') and His_6 (3')	Synthetic gene 2
pTKL_KD_ND	1076-1435	MaBP (5') and His_6 (3')	Obtained by targeted mutagenesis of the pTKL_KD_WT gene
pTKL_KD_DG	1076-1435	MaBP (5') and His_6 (3')	
pTKL_KD_KRIMAS	1076-1435	MaBP (5') and His_6 (3')	

Chart showing the characteristics of the recombinant proteins named in the main text. Synthetic gene sequences are available in annex Supporting Information.

His_6 = 6-histidine tag, GST = glutathione *S*-transferase, MaBP = maltose binding protein.

Supplementary Figure S6. Recombinant protein purification.

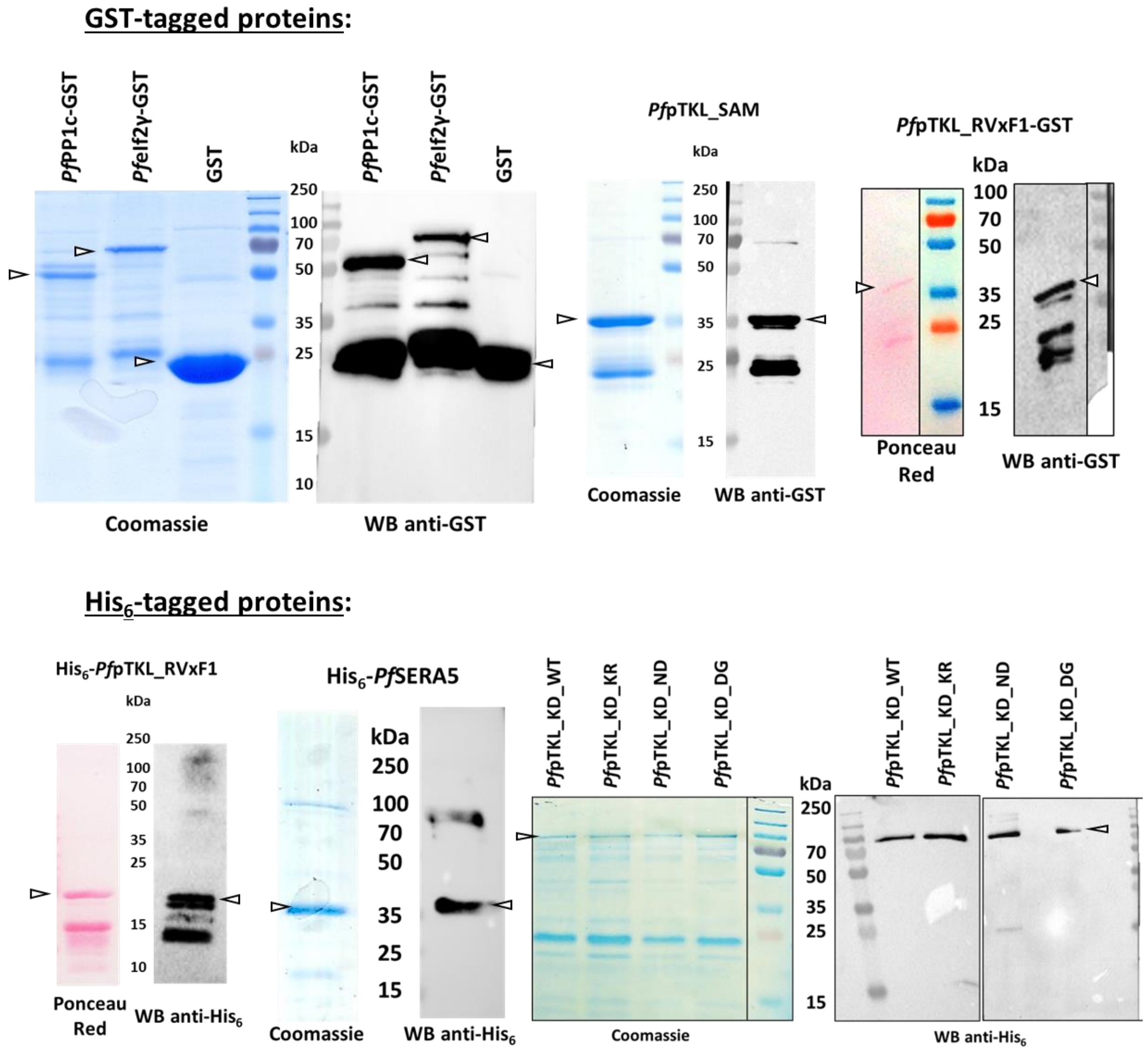

SDS-PAGE followed by western blot analysis was performed to attest for recombinant protein purity. Protein names are displayed above the gels and blots. Gel and western blot types are indicated below.

Supplementary Table S4. Raw data (divided by 1000) – Kinase Glo assays

	pTKL_KD_WT - MBP			pTKL_KD_WT + MBP			pTKL_KD_WTΔ + MBP		
Kinase (μg/well)	0	4	8	0	4	8	0	4	8
Experiment 1	-	670	569	856	702	613	800	770	810
	832	665	649	840	698	561	857	817	743
	722	664	566	835	707	556	857	825	794
Experiment 2	1349	1161	900	1284	902	849	1066	1086	1006
	1334	1157	931	1299	824	652	1024	1208	1183
	1317	1103	1007	1221	860	645	1162	1179	1209
Experiment 3	1227	1021	763	1013	842	656	-	-	-
	1207	991	749	1106	728	562	-	-	-
	1197	1002	712	938	765	587	-	-	-
	**pTKL_KD_ND - MBP**			**pTKL_KD_ND + MBP**			**pTKL_KD_NDΔ + MBP**		
Kinase (μg/well)	0	4	8	0	4	8	0	4	8
Experiment 1	802	672	467	850	629	416	779	782	799
	789	611	511	810	649	452	778	761	816
	787	499	495	734	735	423	771	795	773
Experiment 2	1486	976	675	1280	675	448	1323	1101	1098
	1515	1025	705	1329	699	406	1304	1230	1144
	1462	979	726	1150	605	416	1229	1186	1178
Experiment 3	1403	1160	865	1295	1125	721	1222	1314	1209
	1406	1135	895	1308	1076	766	1176	1215	1178
	1383	1209	941	1259	1075	670	971	1228	1235
	**pTKL_KD_DG - MBP**			**pTKL_KD_DG + MBP**			**pTKL_KD_DGΔ + MBP**		
Kinase (μg/well)	0	4	8	0	4	8	0	4	8
Experiment 1	799	648	488	823	682	541	756	773	760
	792	628	499	812	665	-	784	788	-
	786	751	491	825	672	772	782	754	622
Experiment 2	1423	1100	883	1182	983	755	1279	1238	1288
	1405	1189	921	1283	922	710	1209	1193	1206
	1386	1115	926	1249	954	699	1159	932	1184
Experiment 3	1374	1124	809	1414	1089	587	1247	1212	1210
	1370	1099	874	1177	995	680	1252	1131	1155
	1366	1126	850	1048	1018	762	1204	1167	1178
	**JNK1 - MBP**			**JNK1 + MBP**			**JNK1Δ + MBP**		
Kinase (ng/well)	0	40	80	0	40	80	0	40	80
Experiment 1	861	753	690	843	349	216	792	703	732
	859	755	675	842	339	211	794	876	850
	866	756	663	837	351	-	811	871	850
Experiment 2	1410	1156	835	1327	311	71	1132	1210	1196
	1397	1166	919	1169	382	67	1151	1118	1075
	1365	1152	880	1175	329	93	1090	1071	1141

Supplementary Table S5. Y2H screening results.

Clone number	Growth on TDO/A	Growth on QDO/A	Growth in liquid SD-leucine	DH5α transformation	Sequencing result (grey = out of frame with GAL4-AD)	Protein name	*Pf*SERA5 region (AA)
**12**	+	+	+	+	PF3D7_0827900	**PDI8**	
**156**	+	+	+	+	PF3D7_0207600	**SERA5**	**200-334**
**157**	+	+	+	+	PF3D7_0207600	**SERA5**	**191-323**
**183**	+	+	+	+	PF3D7_0207600	**SERA5**	**185-317**
**199**	+	+	+	+	PF3D7_0827900	**PDI8**	
**249**	+	+	+	-	/		
**605**	+	+	+	+	PF3D7_0207600	**SERA5**	**188-253**
**610**	+	+	+	+	PF3D7_0827900	**PDI8**	
**614**	+	+	+	+	PF3D7_0715600		
**680**	+	+	+	+	PF3D7_0827900	**PDI8**	
**699**	+	-	/	/	/		
**748**	+	+	+	+	PF3D7_1026600		
**988**	+	+	+	+	PF3D7_0207600	**SERA5**	**191-257**
**1236**	+	+	+	+	PF3D7_0207600	**SERA5**	**201-336**
**1237**	+	+	+	+	PF3D7_0207600	**SERA5**	**201-336**
**1537**	+	+	+	+	PF3D7_0312800		
**1546**	+	+	+	+	PF3D7_1028700		
**2775**	+	+	+	+	PF3D7_0207600	**SERA5**	**200-332**
**3070**	+	+	+	+	CHR13		
**3087**	+	+	+	+	PF3D7_1140900		
**3292**	/	+	+	+	PF3D7_0818900		
**3303**	/	+	+	+	PF3D7_0207600	**SERA5**	**191-257**
**3457**	/	+	+	+	PF3D7_0207600	**SERA5**	**145-276**
**3597**	/	+	+	+	PF3D7_0827900	**PDI8**	
**3821**	/	+	+	+	PfEMP1 (VAR)		
**4112**	/	+	+	+	PF3D7_1337500		
**4117**	/	+	+	+	PF3D7_0207600	**SERA5**	**199-265**
**4119**	/	+	+	+	PF3D7_1337500		
**4333**	/	+	+	+	PF3D7_0207600	**SERA5**	**189-255**
**4497**	/	+	+	+	PF3D7_0207600	**SERA5**	**216-365**
**4921**	/	+	+	+	PF3D7_0207600	**SERA5**	**191-257**
**5068**	/	+	+	+	PF3D7_0204100		
**5517**	/	+	+	+	PF3D7_0827900	**PDI8**	

This chart shows the results of a Y2H screening assay in which Y187 cells expressing *Pf*pTKL_SAM&RVxF1 were mated with Y2Hgold cells transformed with a library of *P. falciparum* cDNAs. We streaked 6000 diploids on TDO/A and (or directly on) QDO/A media, 32 of which survived selection (clone identifiers are shown in column 1). They were cultured in liquid SD-leucine to remove the bait pGBKT7 construct (which includes an auxotrophy cassette for tryptophan) – column 4. Plasmid DNA recovered from diploids was propagated in *E. coli* DH5α (column 5) and analyzed by sequencing (column 6). The two main interaction partner names (SERA5, PDI8) are shown in column 7. Regions of *Pf*SERA5 identified by sequencing are shown in column 8 (probable region involved in interaction with SAM = 216–253).

Supplementary Figure S7 - Reverse genetics construct and genotyping data.

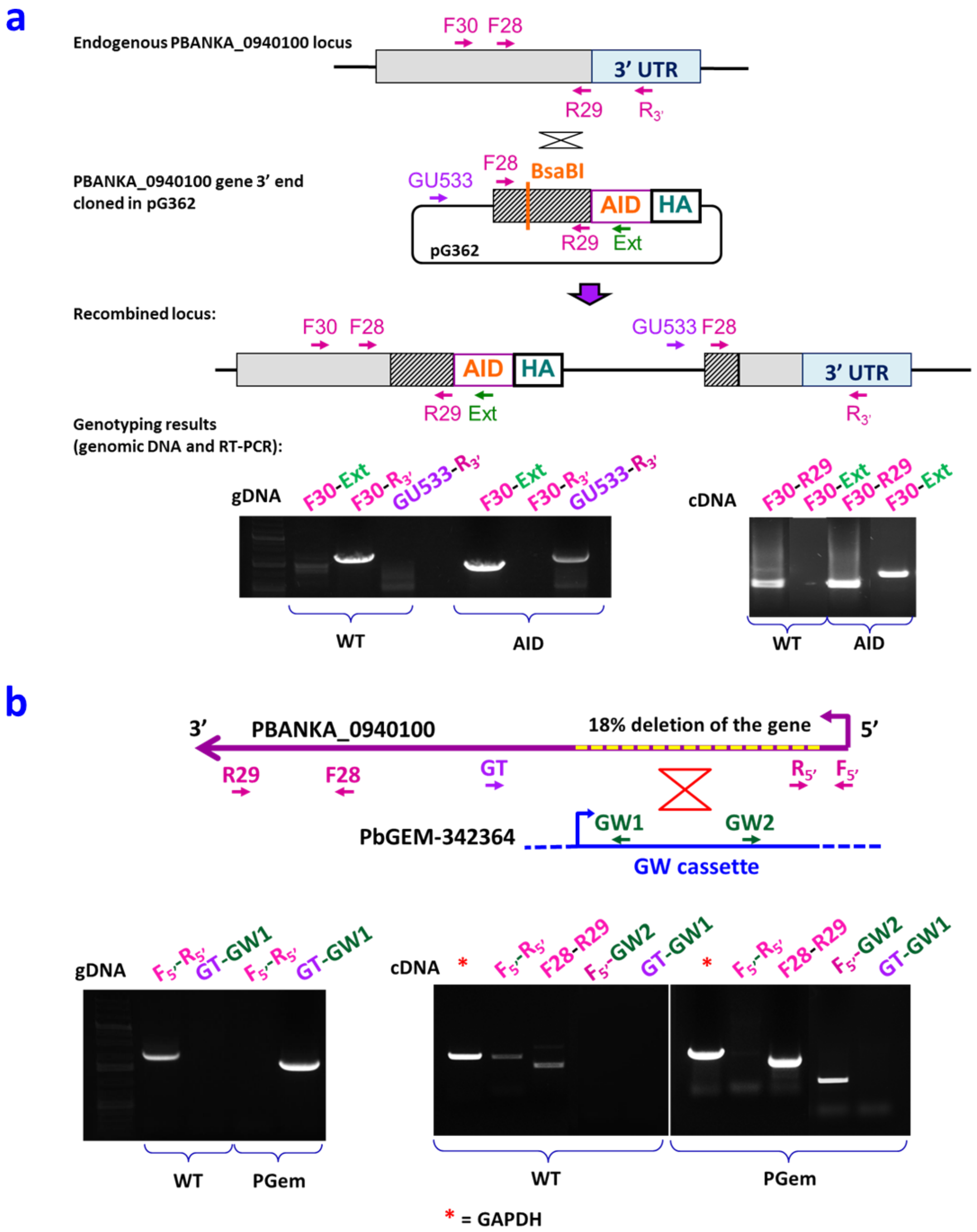

(a) Auxin-induced degradation (AID) construct and genotyping data. An AID-HA knock-in of *PbpTKL* segment amplified using primers F28/R29 (an overlapping PCR was performed with primers F31/R32 to insert the BsaBI restriction site) was cloned in the pG362 plasmid[15]. The construct was linearized with BsaBI before the transfection of pG230 *P. berghei* parasites. After parasite cloning by limiting dilution, endogenous locus modification was assessed by genomic PCR using primers F30/R3' (unmodified endogenous locus), F30-Ext (presence of the AID sequence downstream of *PbpTKL* endogenous locus) and GU533/R3' (full recombination of *PbpTKL*pG362 construct between *PbpTKL* locus and *PbpTKL* 3'UTR).

PbpTKL transcript modification (i.e. including the AID-HA-encoding region) was assessed by RT-PCR. F30/R29 primers were used to verify cDNA preparation. F30-Ext primers were used to detect the *PbpTKL-AID-HA* transcript. **(b) PlasmoGEM construct (PbGEM-342364) and genotyping data.** *PbpTKL* locus modification using the PbGEM-342364 construct (obtained from the Wellcome Sanger Institute)[16]. The plasmid was linearized with NotI before the transfection of *Pb*GFP parasites, which were cloned by limiting dilution. Endogenous locus modification was assessed by genomic PCR using primers F5′/R5′ (unmodified endogenous locus) and GT/GW1 (recombination of PbGEM-342364 construct at *PbpTKL* endogenous locus). *PbpTKL* transcript expression was assessed by RT-PCR (cDNA) in parental (WT) and transfected (PGem) *P. berghei* lines. The cDNA preparations were verified by checking for the presence of the *GAPDH* transcript (red star). The F5′/R5′ primers were used to detect the 5′ end of the *PbpTKL* transcript and the F28/R29 primers were used to detect the 3′ end. The F5′/GW2 and GT/GW1 primers were used to assess the presence of the *dhfr*-containing GW cassette at the cDNA level.

Supplementary Figure S8 – Infected erythrocyte sequential lysis protocol.

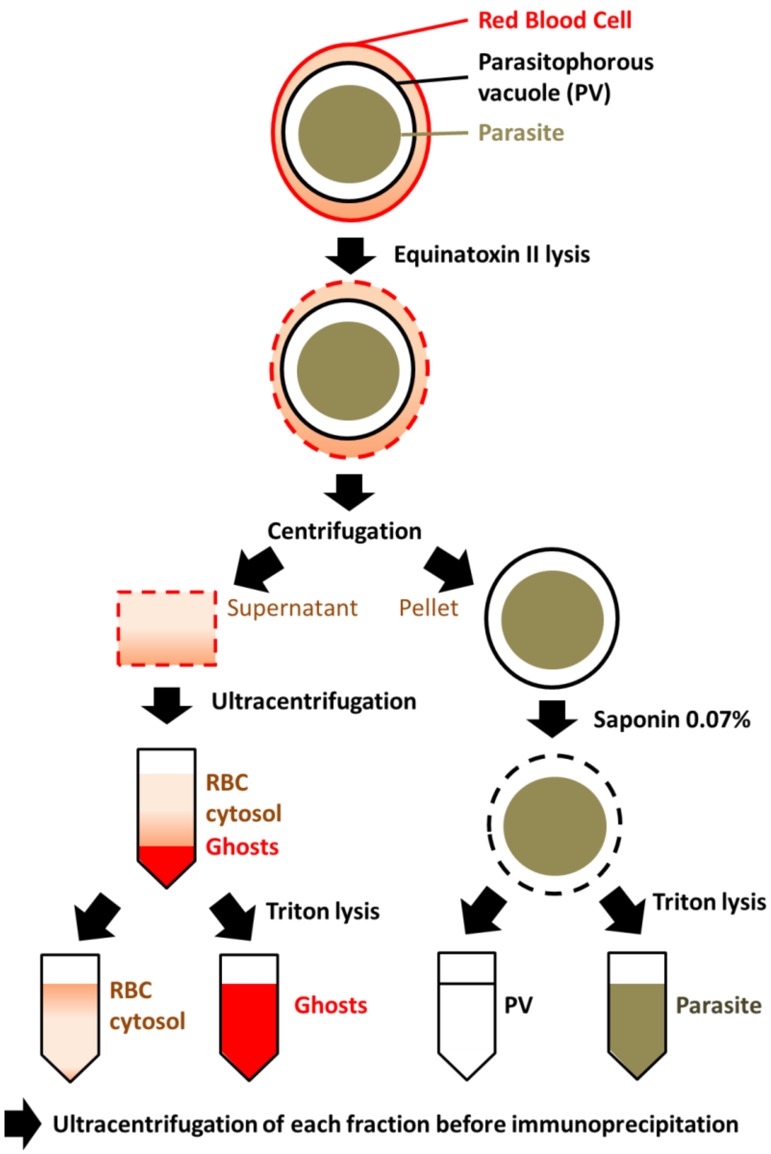

For sequential lysis, a 500-µL dry pellet of infected erythrocytes was lysed in 3 mL of EQT buffer (20 mM Tris pH 7.4, 130 mM NaCl, 1 mM $MgSO_4$, 1/250 Equinatoxin II[17]) on a wheel for 15 min at room temperature. After centrifugation, the supernatant was fractionated by ultracentrifugation (41,657 *g*, 4°C, 15 min) to recover ghosts. The ghosts were lysed in ice-cold Triton buffer (20 mM Tris pH 8.0, 50 mM NaCl, 9% Triton X-100, 5 mM EDTA, Roche cOmplete EDTA-free anti-protease cocktail). The pellet, composed of parasites embedded in their parasitophorous vacuole, was further lysed in 3 mL of 0.07% saponin (on ice for 10 min). After centrifugation as above, the supernatant (the parasitophorous vacuole content) was kept for further analysis while the parasite pellet was lysed in 2 mL of ice-cold Triton buffer. The EQT lysate ultra-supernatant (corresponding to the erythrocyte cytosol) was kept on ice for further analysis. Saponin, parasite and ghost lysates were diluted in total lysis buffer to 5 mL and fractionated by ultracentrifugation as above prior to immunoprecipitation.

Supplementary Figure S9. *Xenopus laevis* oocyte experiments.

a

	GVBD percentage after 15 hours		
**Micro-injection**	**Experiment 1**	**Experiment 2**	**Experiment 3**
**Elution buffer**	10	0	0
**RVxF1**	0	0	0
**KD_RVxF2**	20	20	0
**KD_KRIMAS**	0	0	0
**Progesterone**	100	100	100

b

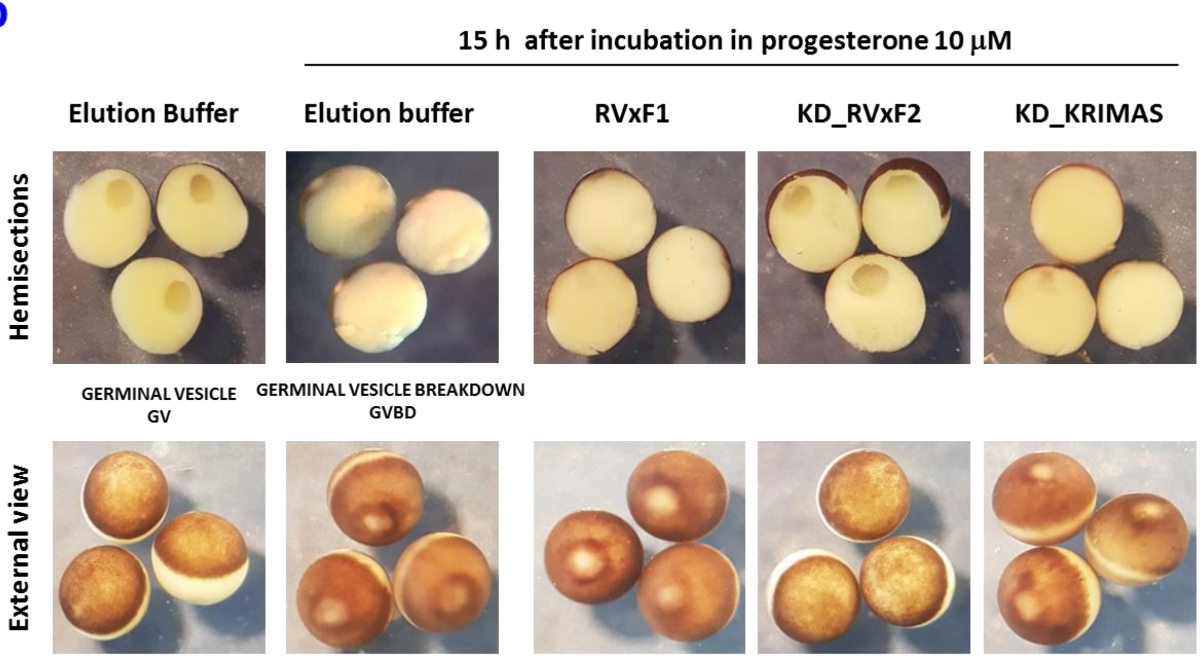

(a) Neither RVxF1 nor KD_RVxF2 micro-injection triggers GVBD. RVxF1, KD_RVxF2 and KD_KRIMAS recombinant proteins were micro-injected into arrested *X. laevis* oocytes (protein elution buffer was used as a negative control). GVBD percentage was assessed 15 h later by looking for the appearance of a white maturation spot. This experiment shows that none of the micro-injected proteins can trigger GVBD. **(b) RVxF2 inhibits progesterone-induced GVBD.** RVxF1, KD_RVxF2 and KD_KRIMAS recombinant proteins were micro-injected into arrested *X. laevis* oocytes. They were treated with progesterone (PG) 1 h later and GVBD was assessed by the rise of the oocyte nucleus to the surface of the oocyte (i.e. the absence of any nucleus inside the oocyte – hemisection lane – and the appearance of a white maturation spot at the surface of the oocyte – outside view lane). This figure supports the chart shown in Figure 5c.

Uncropped blots

Uncropped versions of western blots shown in Figure 5

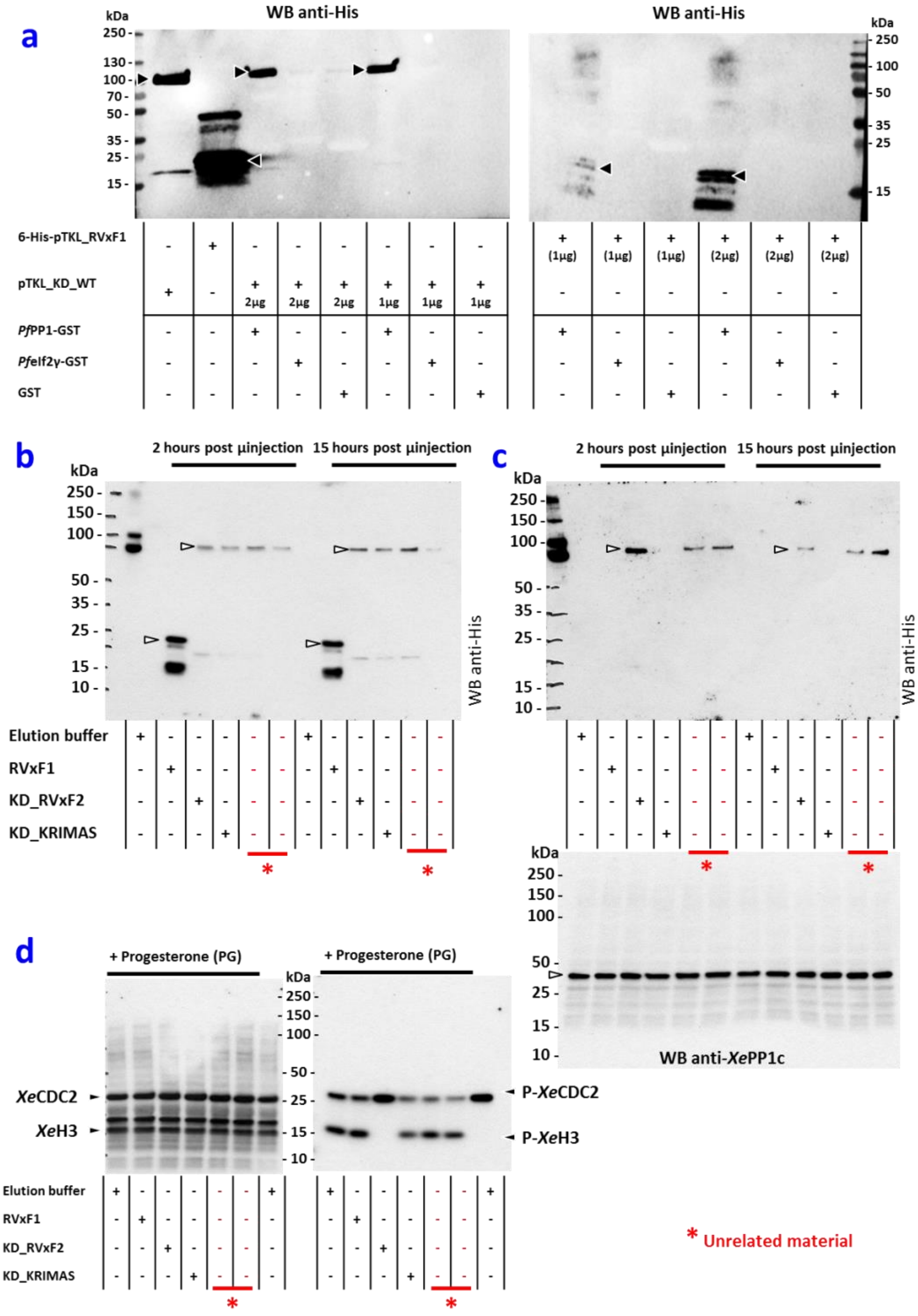

(a) RVxF1 and RVxF2 both directly bind *Pf*PP1c *in vitro* (Fig. 5a)

(b-c) Only RVxF2 binds *Xe*PP1c (Fig. 5b)

(d) Only RVxF2 exerts a functional role in *Xenopus laevis* oocytes (Fig. 5d)

Uncropped versions of western blots shown in Figure 6c

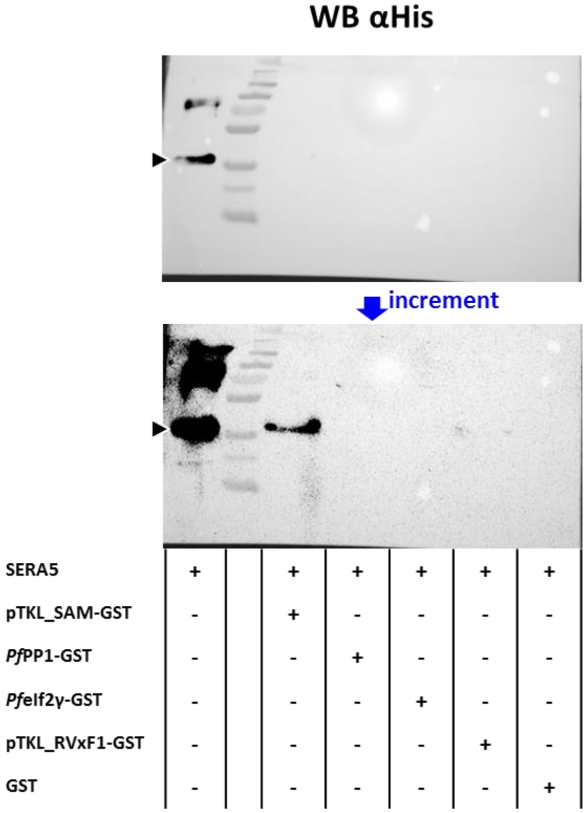

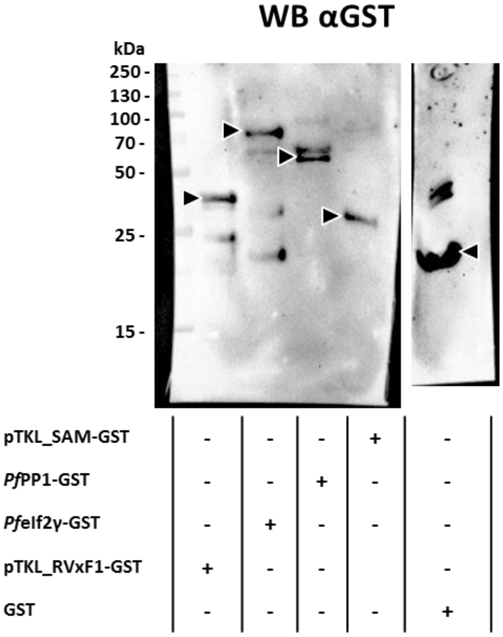

Uncropped versions of western blots shown in Figures 7c-d

a. Fraction Analysis

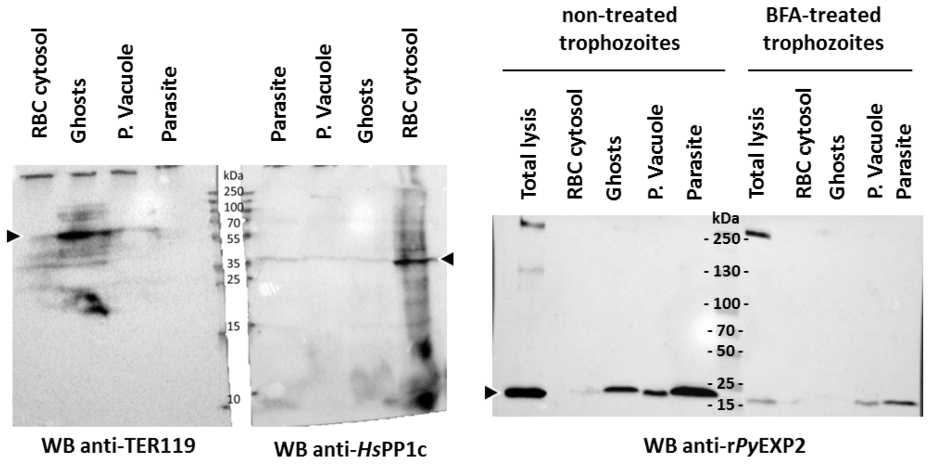

b

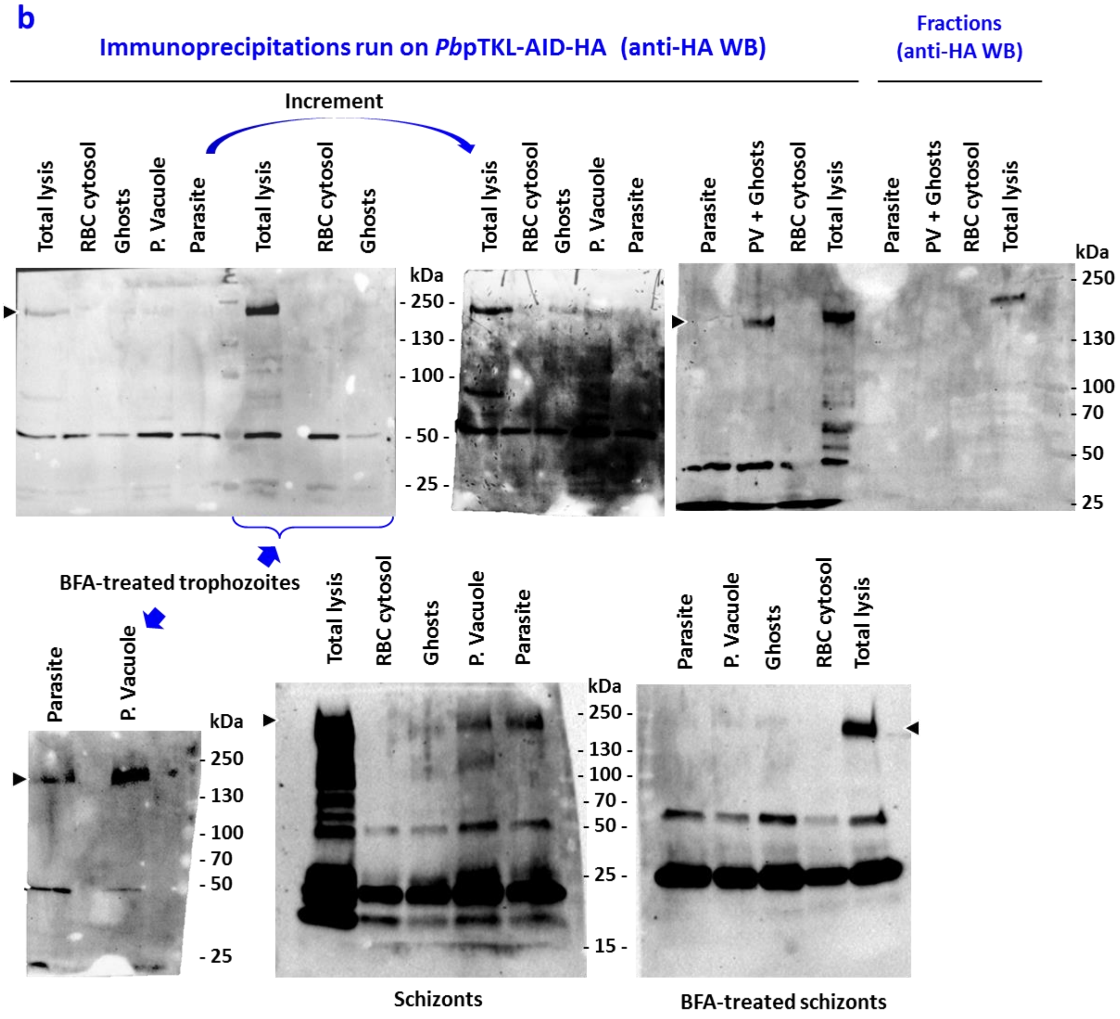

(a) Infected erythrocyte sequential lysis fraction analysis and validation.

(b-c) *Pb*pTKL is actively exported from the parasite between the trophozoite and schizont stages.

Uncropped versions of western blot shown in Figure 8

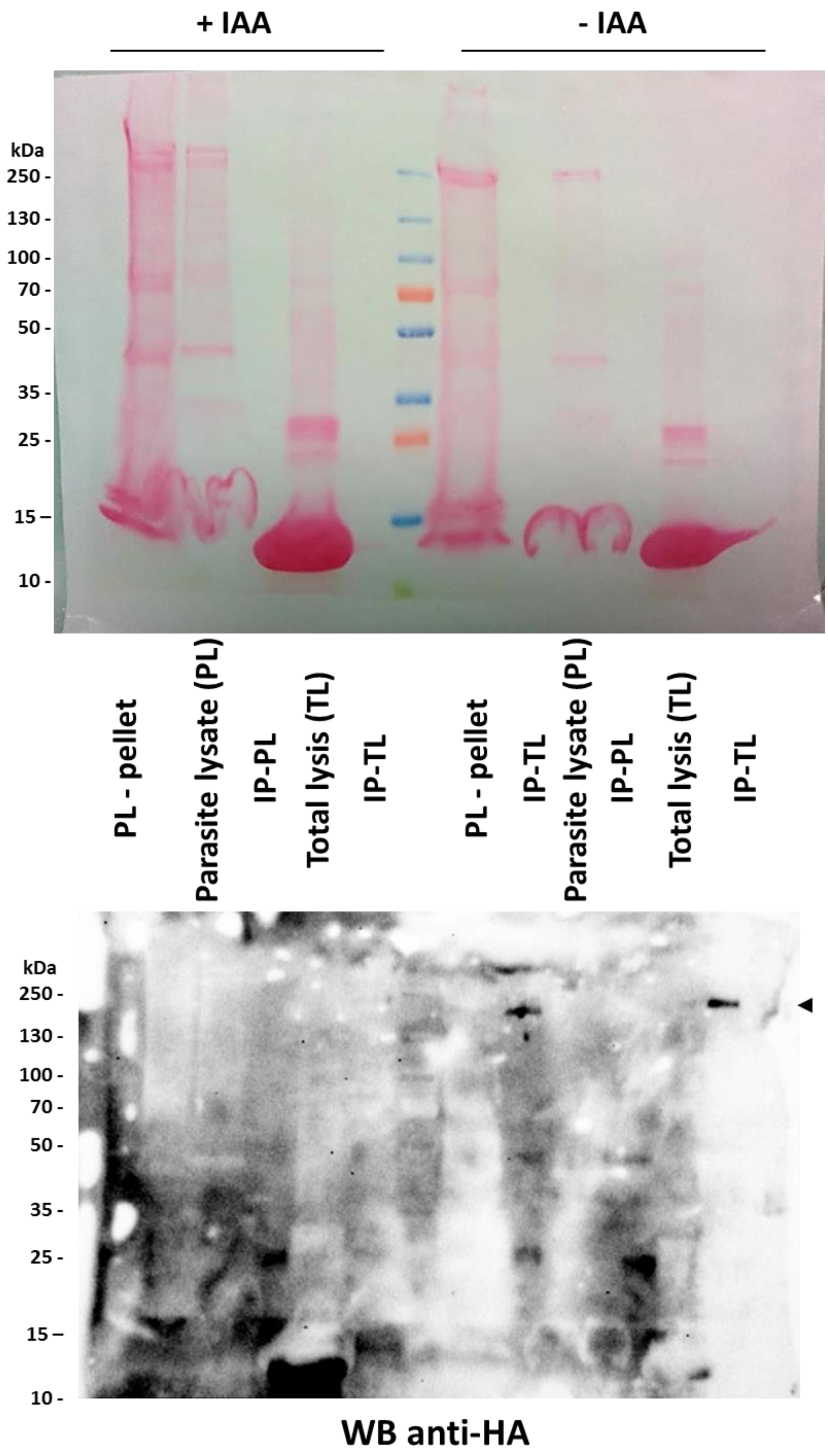

NanoLC/MS protein identification and quantification (mass spectrometry)

S-Trap:

S-Trap micro-spin column (Protifi, Huntington, USA) digestion was performed on immunoprecipitation eluates according to the manufacturer’s protocol. Briefly, 5% SDS was added to the samples. Proteins were alkylated by the addition of iodoacetamide to a final concentration of 50 mM. Aqueous phosphoric acid was added to a final concentration of 1.2%. A colloidal protein particulate was formed by adding six sample volumes of S-Trap binding buffer (90% aqueous methanol, 100 mM Tetraethylammonium bromide (TEAB), pH 7.1). The mixtures were transferred to the S-Trap 1.7-mL columns and centrifuged at 4000 *g* for 30 s. The columns were washed five times with 150 µL S-Trap binding buffer and centrifuged at 4000 *g* for 30 s with 180 degrees rotation of the columns between washes. Samples were digested with 2 µg trypsin (Promega) at 37°C overnight. Peptides were eluted with 40 µL of 50 mM TEAB followed by 40 µL of 0.2% aqueous formic acid and 35 µL 50% acetonitrile containing 0.2% formic acid before vacuum drying.

NanoLC-MS/MS protein identification and quantification:

Samples were resuspended in 35 µL of 0.1% TFA in HPLC-grade water. For each run, 5 µL was injected into a nanoRSLC-Q Exactive PLUS RSLC Ultimate 3000 column (Thermo Fisher Scientific). Peptides were loaded onto a µ-precolumn (Acclaim PepMap 100 C18, cartridge, 300 µm i.d.×5 mm, 5 µm) (Thermo Fisher Scientific, and were separated on a 50 cm reversed-phase column (0.075 mm ID, Acclaim PepMap 100, C18, 2 µm) (Thermo Fisher Scientific). The solvents were (A) 0.1% formic acid in water, and (B) 80% acetonitrile, 0.08% formic acid. Peptides were eluted from the column with the following gradient 5% to 40% B (120 min), 40% to 80% (1 min). The gradient was then maintained at 80% for 5 min before returning to 5% over 20 min for re-equilibration prior to the next injection. Two blanks were run between each series to prevent sample carryover. Peptides eluting from the column were analyzed by data-dependent MS/MS, using the top-10 acquisition method. Peptides were fragmented using higher-energy collisional dissociation (HCD). Briefly, the instrument settings were as follows: resolution was set to 70,000 for MS scans and 17,500 for the data-dependent MS/MS scans in order to increase speed. The MS AGC target was set to 3.106 counts with maximum injection time set to 60 ms, whereas the MS/MS AGC target was set to 1.105 with maximum injection time set to 60 ms. The MS scan range was from 400 to 2000 *m/z*. Dynamic exclusion was set to 30 s duration.

Supporting Information 1 – Synthetic gene 1 – pTKL_SAM-GST

Gene Length: 342 bp,Vector name: pUC57, Cloning site: EcoRV-HindIII ,

Gene sequence:

GATATCGGATCCGAATTCAAAAAACTGTTCAAATACTTTAGCAATAACATCGAAAACCTGATTATCGAAAACTACCA
AACCTGGAGCCTGCGCGAAGTGATTCAATGGCTGATGCTGTGCAACGTGCCGGTTAAGTGGCTGATCAGCTTCTAC
AAAAACAACATTACCGGTGACAAGCTGAAATATATCAACATTAACACCATCCGTAACGAGCTGGGTATCATTGCGT
ACGGCCACGCGATCAAGATTCTGCAGCTGATTAAAAACCTGCAAGTGATGGCGTATAATAAGAAGTTTAACAACCT
GATCCAGATTGAGGAGTACAAGAATCTCGAGAAGCTT

Protein sequence:

DIGSEFKKLFKYFSNNIENLIIENYQTWSLREVIQWLMLCNVPVKWLISFYKNNITGDKLKYININTIRNELGIIAYGHAIKIL
QLIKNLQVMAYNKKFNNLIQIEEYKNLEKL

Supporting Information 2 – Synthetic gene 2 – pTKL_KD_WT

Gene Length: 1116 bp,Vector name: pEX-A258, Cloning site: EcoRI-BamHI ,

GAATTCGACAATCACATTTACTGCAATAACATTTACGATCACCACAAAAACACGTCATTGAACTCGAAAGAACAAAA
TACGGACCATAACATCGAACAGATTAATGAGTGTAACAAATATGCCAGTGAAACCAAATACAACATTAAGAAATCG
AACCTGAAGAATAACATTATCTCGCATAAGAACTTCCAGAAATGCAATCAGATTCAGATGAACCAACCCTACACGTT
TCCACCATATCAGAAAGAACTGAGCTCTTATCTTAAAAACGAAAAGATCAAACGGAAACGCAAAGTGCTGTTT
TCCTACCTGAAAACCCATATTCACTTCAACAGTCAGCAGATTAACGATCAACACAATCGCTTATCTGTGCAGAAAAT
TATGAAAATCATTACTGATGTCACCCTTGCTTGCACATATCTGGAAAAGGAGAAAATGAGCCCCATTAATCTGAAAC
CTACCAATATCCTGTTGGACGAATCTCTGAATGCCAAAATTAGCGATTTCGGTATCAGCAAAATTGAGAATTGTCTG
GATATGAACATTGACTATTCATACAAAATTAGCTCCAACTCAGTCATCAAAATTAATAAAAAAGAATATGAGCAAAA
GAAAGCGAAGAAAATCAAAATTGTGAATAAAAACAATAATGACTTACTGTACCTGTACGATCACAATAACAACGTC
TATAAGTACAATACCCAATATATCGACGTAACCTATAACAACAGTTATCCGAGCATCTTCTATTGGACTCCTCCGGA
AATCTTACGTGGCAAAAAAAACAAGAAATTTTACAGCGATATTTACGCATTTGGGATCATCCTCTGGGAAATGCTG
AGTAATGATATTCCGTACAACTATCCGTTTGCATCCCATATTATGGCCGTTGTAGGCTATGCGAATGAAGAACTGTC
ATTCAACAACATTCCGGTTTCCATTCAGTCGTTGATCAAAGCGTGTGTTAATCGCAACAAATATAAGCGTCCGACGT
TTGAGCATATTCTGAAAACGATCTCTACACTCTATCAGAAAGCGAATACCAAAGTGGAAGATGCTCTTATCAGCTTT
ATGGATGGTACTCTCGAGCATCATCACCATCATCATTAAGGATCC

Protein Sequence:

EFDNHIYCNNIYDHHKNTSLNSKEQNTDHNIEQINECNKYASETKYNIKKSNLKNNIISHKNFQKCNQIQMNQPYTFPPY
QKELSSYLKNEKIKRKRKVLFSYLKTHIHFNSQQINDQHNRLSVQKIMKIITDVTLACTYLEKEKMSPINLKPTNILLDESLNA
KISDFGISKIENCLDMNIDYSYKISSNSVIKINKKEYEQKKAKKIKIVNKNNNDLLYLYDHNNNVYKYNTQYIDVTYNNSYPSI
FYWTPPEILRGKKNKKFYSDIYAFGIILWEMLSNDIPYNYPFASHIMAVVGYANEELSFNNIPVSIQSLIKACVNRNKYKRP
TFEHILKTISTLYQKANTKVEDALISFMDGTLEHHHHHH

Supporting Information 3 – MAFFT Alignment of *Pf*pTKL orthologs BTK-like domains

```
PF3D7_1106800 AA numbering   841        851        860        868        878        888        895        905
                     ....|....| ....|....| ....|....| ....|....| ....|....| ....|....| ....|....| ....|....|
Hs_PKAc_Nlobe        GTGSFGRVM- -LVKHKETGN HYAMKILDKQ KVV--KLKQI EHTLNEKRIL QAVNFPFLVK LEFSFKDNSN LYMVMEYVPG

Hs_BTK_Nlobe         GTGQFGVVKY GKWRGQY--- DVAIKMIKEG SMSEDEFIEE AKVMMN---L SHEKLVQLYG VCTKQR---P IFIITEYMAN

PF3D7_1106800        ---KYKKMKS RMFKGKYMGK EVAIKIL-VG KIK--NFKKL HQILYNLYNL RHSNLVLIMG VSIHYP---F VFIIYEYMKN

PFIT_1107600         ---KYKKMKS RMFKGKYMGK EVAIKIL-VG KIK--NFKKL HQILYNLYNL RHSNLVLIMG ISIHYP---F VFIIYEYMKN

PPRFG01_1107500      ---KYKKMKS RMFKGKYMGK EVAIKIL-VG KIK--NFKKL HQVLYNLYNL RHSNLVLIMG VSIHYP---F VFIIYEYMKN

PRCDC_1105300        ---KYKKMKS RMFKGKYMGK EVAIKIL-VG KIK--NFKKL HKILYNLYNL RHSNFVLIMG VSIHYP---F VFIIYEYMKN

PRG01_1104000        ---KYKKMKS RMFKGKYMGK EVAIKIL-VG KIK--NFKKL HKILYNLYNL RHSNFVLIMG VSIHYP---F VFIIYEYMKN

PBLACG01_1104300     ---KYKKMKS RMFKGKYMGK EVAIKIL-VG KIK--NFKKL HQILYNLYNL RHTNLVLIMG VSIHYP---F VFIIYEYMKN

PADL01_1105000       ---KYKKMKS RMFKGKYMGK EVAIKIL-VG KIK--NFKKL HQILYNLYNM RHSNFVLIMG VSIHYP---F IFIIYEYMKN

PGABG01_1103900      ---KYKKMKS RMFKGKYMGK EVAIKIL-VG KIK--NFKKL HQILYNLYNM RHSNFVLIMG VSIHYP---F IFIIYEYMKN

PGSY75_1106800       ---KYKKMKS RMFKGKYMGK EVAIKIL-VG KIK--NFKKL HQILYNLYNM RHSNFVLIMG VSIHYP---F IFIIYEYMKN

PBILCG01_1104000     ---KYKKMKS RMFKGKYMGK EVAIKVL-VG KIK--NFKKL HQTLYKLYNL RHSNLVLIMG VSIHYP---F LFIIYEYMKN

PGAL8A_00334100      ---KYTKLKS RVFKGKYMGK EVAIKVL-VG KIK--NFNKL HKILYKLYIL RHSNIVLIMG VSISYP---F VFIVYEYLKN

PRELSG_0904000       ---KYRKLKS RVFKGKYMGK EVAIKVL-VG KIK--NFNKL HKILYKLYIL RHSNIVLIMG ISISYP---F VFIVYEYLKN

PYYM_0941700         ---KPQKMKS RIFRGRYMGK DVAIKVL-VG NIK--NFTKF HKVLYNLYIL RHTNIALIMG VSISYP---F VFIIYEYIKN

PY02791              ---KPQKMKS RIFRGRYMGK DVAIKVL-VG NIK--NFTKF HKVLYNLYIL RHTNIALIMG VSISYP---F VFIIYEYIKN

PBANKA_0940100       ---KPQKMKS RVFRGRYMGK DVAIKVL-VG NIK--NFTKF HKVLYKLYIL RHTNIALIMG VSISYP---F VFIIYEYIKN

PCHAS_0904200        ---KPQKMKS RVFRGRYMGK DVAIKVL-VG NIK--NFTKF HKVLYKLYIL RHTNIALIMG VSISYP---F VFIIYEYVKN

YYG_01693            ---KPQKMKS RVFRGRYMGK DVAIKVL-VG NIK--NFTKF HKVLYKLYIL RHTNIALIMG VSISYP---F VFIIYEYVKN

YYE_02751            ---KPQKMKS RVFRGRYMGK DVAIKVL-VG NIK--NFTKF HKVLYKLYIL RHTNIALIMG VSISYP---F VFIIYEYVKN

PmUG01_09016400      ---NYRKLKS RVFKGKYMGK DVAIKVL-VG KIK--NFYHF HKMIYKLYVL RHSNIALIMG VAIYYP---F VFIVYEYLKN

PocGH01_09013500     ---RYKRLHS RVFLGKYMGK DVAIKVL-VG KIT--KFHKL YRILYKLYTL RHSNIVLIMG VSIKYP---F LFIVYEHVKN

C922_04039           ---NGKTMQS RTFRGKYLGK DVAIKVL-VG RVK--DFFQI HKIFYKLHLF GHGNIALMMG VSIRYP---F VFIIYEFLKN

PcyM_0908600         ---NGKIMGS RTFRGKYLGK DVAIKVL-VG RVK--DFSQI HKIFYKLHIL RHGNIALMMG VSIRYP---F VFIVYEFLKN

PVP01_0907600        ---NGKVMRS RTFRGKYLGK DVAIKVL-VG RVK--DFSQI HKIFYKLHLL RHGNIALMMG VSIRYP---F VFIIYEFLKN

PVX_091015           ---NGIVMRS RTFRGKYLGK DVAIKVL-VG RVK--DFSQI HKIFYKLHLL RHGNIALMMG VSIRYP---F VFIIYEFLKN

PKNOH_S120122800     ---NDKIMNS RTFRGKYLGK DVAIKVL-VG RVK--DFSEI HKVFYKLHLL RHGNIALMMG VSIRYP---F VFIISEFLKN

PKNH_0904400         ---NDKIMNS RTFRGKYLGK DVAIKVL-VG RVK--DFSEI HKVFYKLHLL RHGNIALMMG VSIRYP---F VFIISEFLKN
```

Supporting Information 4 - MAFFT Alignment Kinase Domains (phylogenetics analysis)

```
         10        20        30        40        50        60        70        80        90        100
....|....|....|....|....|....|....|....|....|....|....|....|....|....|....|....|....|....|....|....|
At_CDPK1 -------------------------------------------YILGR-----ELGRGEFGITYL-----------------------------------
At_CTR1 -------------------------------------------LNIKE-----KIGAGSFGTVHR-----------------------------------
FIKK1(PF3D7_0102600) ----------------------------------------------------------------------------------------------------
FIKK10.1(PF3D7_1016400) ----------------------------------------------------------------------------------------------------
FIKK10.2(PF3D7_1039000) ----------------------------------------------------------------------------------------------------
FIKK11(PF3D7_1149300) YNKKKFRVQEMFK-TVITSKNDDNKNNISLFIKKIPVDIWLKQFEMMELYNGEYLVNAENYVMEA-----------------------------------
FIKK12(PF3D7_1200800) ----------------------------------------------------------------------------------------------------
FIKK3(PF3D7_0301200) ----------------------------------------VKQYNLMTEYDGEYLLAGENAVMEA-----------------------------------
FIKK4.1(PF3D7_0424500) ----------------------------------------------------------------------------------------------------
FIKK5(PF3D7_0500900) YSVRTSRVQKMFK-TIIDPKKENAKDQVKAFIKKIPVDIWVKQFHSMNEYDGEFLVGGENFVMEA-----------------------------------
FIKK7.1(PF3D7_0726200) ----------------------------------------------------------------------------------------------------
FIKK8(PF3D7_0805700) --------------------------------------IWVKQFNLMNEYDGEYVTDGENFVMEA-----------------------------------
FIKK9.1(PF3D7_0902000) ----------------------------------------------------------------------------------------------------
FIKK9.2(PF3D7_0902100) ----------------------------------------------------------------------------------------------------
FIKK9.4(PF3D7_0902300) ----------------------------------------------------------------------------------------------------
FIKK9.5(PF3D7_0902400) ----------------------------------------------------------------------------------------------------
FIKK9.6(PF3D7_0902500) ----------------------------------------------------------------------------------------------------
FIKK9.7(PF3D7_0902600) -------------------------------------------YEKMELYNGEYILKGENYVMEA-----------------------------------
Hs_ABL1 -------------------------------------------ITMKH-----KLGGGQYGEVYE-----------------------------------
Hs_BTK -------------------------------------------LTFLK-----ELGTGQFGVVKY-----------------------------------
Hs_CAMK1 -------------------------------------------YDFRD-----VLGTGAFSEVIL-----------------------------------
Hs_CDK2 -------------------------------------------FQKVE-----KIGEGTYGVVYK-----------------------------------
Hs_CK1 -------------------------------------------YKLVR-----KIGSGSFGDIYL-----------------------------------
Hs_CLK1 -------------------------------------------YEIVD-----TLGEGAFGKVVE-----------------------------------
Hs_ERK1 -------------------------------------------YTQLQ-----YIGEGAYGMVSS-----------------------------------
Hs_FGFR1 -------------------------------------------LVLGK-----PLGEGCFGQVVL-----------------------------------
Hs_GCN2_2 -------------------------------------------FEELQ-----LLGKGAFGAVIK-----------------------------------
Hs_IGFR1 -------------------------------------------ITMSR-----ELGQGSFGMVYE-----------------------------------
Hs_IRAK1 ---------------------------------------------FSEE---LKIGEGGFGCVYR-----------------------------------
Hs_JAK2_1 -------------------------------------------LIFNE-----SLGQGTFTKIFK-----------------------------------
Hs_LCK -------------------------------------------LKLVE-----RLGAGQFGEVWM-----------------------------------
Hs_LYN -------------------------------------------IKLVK-----RLGAGQFGEVWM-----------------------------------
Hs_NEK1 -------------------------------------------YVRLQ-----KIGEGSFGKAIL-----------------------------------
Hs_PAK1 -------------------------------------------YTRFE-----KIGQGASGTVYT-----------------------------------
PfPK8(PF3D7_0203100) R---------------------------------------INEEHKNE-----GINKLTYHNMNK-----------------------------------
Hs_PKAca -------------------------------------------FERIK-----TLGTGSFGRVML-----------------------------------
Hs_RAF1 -------------------------------------------VMLST-----RIGSGSFGTVYK-----------------------------------
Hs_RIOK1 --------------------------------------------TEING----CISTGKEANVYH-----------------------------------
Hs_RIOK2 ---------------------------------------------VGN-----QMGVGKESDIYI-----------------------------------
Hs_RIOK3 --------------------------------------------ETITG----CISTGKESVVFH-----------------------------------
Hs_VGFR1 -------------------------------------------LKLGK-----SLGRGAFGKVVQ-----------------------------------
PBANKA_0940100 -------------------------------------------FTNES----------------------------------------------------
PbRIO1(PBANKA_1445600) TRDKRATVN-----SVLD-------NRTLLILKKL----KNTFFNEIYG----VVSSGKEAFVFNAHKILSNEEIKSVKEILFRYAKKWNTYKRKVNGNV
PbRIO2(PBANKA_0521400) ----ENKIYDGYKLTYLG-------YDFLALRAFL---NRGILKSVGN-----QIGVGKESDIYI-----------------------------------
PbTKL1(PBANKA_0308500) -------------------------------------------IWLLK-----LIGRGGYNNVYK-----------------------------------
PbTKL2(PBANKA_0927000) -------------------------------------------FSEEN-----KIAKGGNGIVYK-----------------------------------
PbTKL4(PBANKA_1122700) ---------------------------------------------VNEE----EENGNKDGEVYM-----------------------------------
PF3D7_1106800 -----------------------------------------------H-----NVLCGAYDNNDN---NINDNDIY------------------------
PfABCk1(PF3D7_0810200) ---------------------------------------------------------KEIGEDYE-----------------------------------
PfABCk2(PF3D7_1414500) ---------------------------------------------------------KELGNIYE-----------------------------------
PfARK1(PF3D7_0605300) -------------------------------------------FDIAG-----FLGDGAHGSVFL-----------------------------------
PfARK2(PF3D7_0309200) -------------------------------------------IVDKY-----PIGNGRTGLVFK-----------------------------------
PfARK3(PF3D7_1356800) -------------------------------------------FSDPG-----NIGEGGFGIVTK-----------------------------------
PfCDPK1(PF3D7_0217500) -------------------------------------------YFKVR-----KLGSGAYGEVLL-----------------------------------
PfCDPK2(PF3D7_0610600) -------------------------------------------YIIDE-----KLGQGTYGCVYK-----------------------------------
PfCDPK3(PF3D7_0310100) -------------------------------------------NLSKE-----PLGKGTYGCVYK-----------------------------------
PfCDPK4(PF3D7_0717500) -------------------------------------------YKGIK-----ILGKGSFGEVIL-----------------------------------
PfCDPK5(PF3D7_1337800) ------------------------------------------YEIDRY-----KLGKGSYGNVVK-----------------------------------
PfCDPK6(PF3D7_1122800) -------------------------------------------LSFKK-----ILGYGAFGEVHL-----------------------------------
PfCDPK7(PF3D7_1123100) -------------------------------------------YELHE-----QLGQGKFSTVYR-----------------------------------
PfCK1.1(PF3D7_1136500.2) -------------------------------------------YALGK-----KLGSGSFGDIYV-----------------------------------
PfCK2alpha(PF3D7_1108400) -------------------------------------------YEIMK-----KIGRGKYSEVFN-----------------------------------
PfCLK1(PF3D7_1445400) -------------------------------------------FLVIR-----KMGDGTFGRVLL-----------------------------------
PfCLK3(PF3D7_1114700) --------------------------------------------VVCE-----LVGKGVFSNVLK-----------------------------------
PfCRK1(PF3D7_0417800) -------------------------------------------YKKLN-----KISEGTYGAVYR-----------------------------------
PfCRK3(PF3D7_0415300) ---------------------------------------------------------SSY----------------------------------------
PfCRK4(PF3D7_0317200) ---------GVG-----------------------------------------KISRGVHNYAYKS----------------------------------
PfCRK5(PF3D7_0615500) -------------------------------------------FRKNV----------------------------------------------------
PfGSK3(PF3D7_0312400) -------------------------------------------YKLGN-----IIGNGSFGVVYE-----------------------------------
PfIK1(PF3D7_1444500) ------------------------------------------EKMKNE-----KIISGKIKNENI-----------------------------------
PfKIN(PF3D7_1454300) -------------------------------------------IVTKK-----KIGKGTFGKVCL-----------------------------------
PfMAPK1(PF3D7_1431500) -------------------------------------------YDILK-----KVGKGAYGVVFK-----------------------------------
PfMAPK2(PF3D7_1113900) -------------------------------------------YEIKH-----LIGRGSYGYVYL-----------------------------------
PfMRK(PF3D7_1014400) -------------------------------------------IFKPN-----FLGEGSYGKVYK-----------------------------------
PfNEK1(PF3D7_1228300) -------------------------------------------YEVIK-----KIGNGRFGEVFL-----------------------------------
PfNEK2(PF3D7_0525900) -------------------------------------------YEVVK-----SIGRGSFGIVTA-----------------------------------
PfNEK3(PF3D7_1201600) -----------------------------------------------D-----EIYISKVYDIYG-----------------------------------
PfNEK4(PF3D7_0719200) -------------------------------------------YEKIR-----DIGKGNYGNTIL-----------------------------------
PfPK1(PF3D7_0821100) ----------------------------------------------------------------------------------------------------
PfPK2(PF3D7_1238900) -------------------------------------------YVLNK-----KIGKGSFSTAYI-----------------------------------
PfPK4(PF3D7_0628200) ----------------------------------------------------------------------------------------------------
PfPK5(PF3D7_1356900) -------------------------------------------YHGLE-----KIGEGTYGVVYK-----------------------------------
PfPK6(PF3D7_1337100) -------------------------------------------FDFLY-----VIGKGTYGIVYK-----------------------------------
PfPK7(PF3D7_0213400) -------------------------------------------YRIIR-----TLNQGKFNKIIL-----------------------------------
PfPK9(PF3D7_1315100) -------------------------------------------YIVEK-----RIGSGGFGIVFQ-----------------------------------
PfPKA(PF3D7_0934800) -------------------------------------------FNFIR-----TLGTGSFGRVIL-----------------------------------
PfPKB(PF3D7_1246900) -------------------------------------------FNYLK-----VIGEGSYGKVML-----------------------------------
PfPKG(PF3D7_1436600) -------------------------------------------LETER-----IIGRGTFGTVKL-----------------------------------
PfPutativeCaMK2(PF3D7_1423600) -------------------------------------------YKFGK-----ILGCGSFGVVRE-----------------------------------
PfPutativeK(PF3D7_0107600) -------------------------------------------ILKGKY----CYNNGNFALLSY-----------------------------------
PfPutativeK(PF3D7_0321400) ----------------------------------------------YNC---IFLKKGEKRLVRK-----------------------------------
PfPutativeK(PF3D7_0926100) -----------------------------------------------KY---TVIEEGSFGVVYK-----------------------------------
```

```
PfPutativeK(PF3D7_1145200)    --------------------------------------------IVLS-----NLGKGTYAQVWK-----------------------------------
PfPutativeK(PF3D7_1148000)    -------------------------------------------IRMMN-----LIGRGGFAEVWE-----------------------------------
PfPutativeK(PF3D7_1316000)    -------------------------------------------YKLMK-----LIGKGTFGKVYS-----------------------------------
PfPutativeK(PF3D7_1331000)    -------------------------------------------------------NGNAYGHMKN-----------------------------------
PfPutativeK(PF3D7_1441300)    -------------------------------------------YIILE-----KINTGSVGQVHV-----------------------------------
PfRIO1(PF3D7_1230900)         TRDKRATIN-----SVLD-------NRTLIILRKL----KENVYNDIYG----VISSGKEAYVFNAIKYINDEELNSLKNIFIKHLEK---YKNNINIKI
PfRIO2(P3D7_0420100)          -------------------------------------------------------------DIYI-----------------------------------
PfSRPK1(PF3D7_0302100)        ----------------------------------------------------------------------------------------------------
PfSRPK2(PF3D7_1443000)        ----------------------------------------------------------PHNNINI-----------------------------------
PfTKL1(PF3D7_0211700)         -------------------------------------------LHILN-----LIGRGGYSNVYR-----------------------------------
PfTKL2(PF3D7_1121300)         -------------------------------------------FSEYN-----RIAKGGNGTVYK-----------------------------------
PfTKL3(PF3D7_1349300)         ----------------------------------------------------------------------------------------------------
PfTKL4(PF3D7_0623800)         -----------------------------------------------S-----TTHHDSEEIIYM-----------------------------------
PfVPS15(PF3D7_0823000)        ----------------------------------------NLDHYLSN-----KINTNNFYYFYI-----------------------------------
PVX_091015                    ----------------------------------------------------------------------------------------------------
Py02791                       ----------------------------------------------------------------------------------------------------
TGGT1_209050 (TgTKL5)         -------------------------------------------ICIVA-----PISQGSFGTVSR-----------------------------------
TGGT1_225770 (TgTKL8)         -------------------------------------------AGFSV-----ILGQGAYGNVYE-----------------------------------
TGGT1_234970 (TgTKL2)         -------------------------------------------YRLLN-----LLGKGGFAEVWE-----------------------------------
TGGT1_236240 (TgTKL6)         -------------------------------------------LREER-----QLSEGGFGFVSC-----------------------------------
TGGT1_237210 (TgTKL4)         -------------------------------------------VSMNG-----LIGKGATSSVYR-----------------------------------
TGGT1_239130 (TgTKL7)         -------------------------------------------AFCLD-----PAGDGSLSASTSLAVSLAPQLPVTQP---------------------
TGGT1_253860 (TgTKL3)         -------------------------------------------AESRQ-----SDNAGSEAELTRQ----------------------------------
TGGT1_290225                  ----------------------------------------------------------------------------------------------------
TGGT1_301270 (TgTKL1)         -------------------------------------------FDFRE-----KLGKGSFGEVWR--VRIKSE---------------------------

                                       110       120       130       140       150       160       170       180       190       200
                              ....|....|....|....|....|....|....|....|....|....|....|....|....|....|....|....|....|....|....|....|
At_CDPK1                      -------------------------------------------------------CTDRETHEA------------------------------------
At_CTR1                       -------------------------------------------------------AEWHGS---------------------------------------
FIKK1(PF3D7_0102600)          ----------------------------------------------------------------------------------------------------
FIKK10.1(PF3D7_1016400)       ----------------------------------------------------------------------------------------------------
FIKK10.2(PF3D7_1039000)       ----------------------------------------------------------------------------------------------------
FIKK11(PF3D7_1149300)         -------------------------------------------------------SILAFLNEYYQG---------------------------------
FIKK12(PF3D7_1200800)         -------------------------------------------------------VALSFLSEHHPG---------------------------------
FIKK3(PF3D7_0301200)          -------------------------------------------------------MALAFLNEYHPN---------------------------------
FIKK4.1(PF3D7_0424500)        ----------------------------------------------------------------------------------------------------
FIKK5(PF3D7_0500900)          -------------------------------------------------------VTSAFLTKYHPG---------------------------------
FIKK7.1(PF3D7_0726200)        -----------------------------------------------------------FV---------------------------------------
FIKK8(PF3D7_0805700)          -------------------------------------------------------TALAFLNEYHPG---------------------------------
FIKK9.1(PF3D7_0902000)        --------------------------------------------------------------EFY-----------------------------------
FIKK9.2(PF3D7_0902100)        ----------------------------------------------------------------------------------------------------
FIKK9.4(PF3D7_0902300)        -----------------------------------------------------------FL---------------------------------------
FIKK9.5(PF3D7_0902400)        ----------------------------------------------------------------------------------------------------
FIKK9.6(PF3D7_0902500)        ----------------------------------------------------------------------------------------------------
FIKK9.7(PF3D7_0902600)        -------------------------------------------------------VVSAFLSEYHPG---------------------------------
Hs_ABL1                       -------------------------------------------------------GVWKK----------------------------------------
Hs_BTK                        -------------------------------------------------------GKWRG----------------------------------------
Hs_CAMK1                      -------------------------------------------------------AEDKRTQKL------------------------------------
Hs_CDK2                       -------------------------------------------------------ARNKLTGEV------------------------------------
Hs_CK1                        -------------------------------------------------------AINITNGEE------------------------------------
Hs_CLK1                       -----------------C-------------------------------------IDHKAGGRH------------------------------------
Hs_ERK1                       -------------------------------------------------------AYDHVRKTR------------------------------------
Hs_FGFR1                      -------------------------------------------------------AEAIGLDKD------------------------------------
Hs_GCN2_2                     -------------------------------------------------------VQNKLDGCC------------------------------------
Hs_IGFR1                      -------------------------------------------------------GVAKGVVKD------------------------------------
Hs_IRAK1                      -------------------------------------------------------AVMRNT--V------------------------------------
Hs_JAK2_1                     -------------------------------------------------------GVRREVGDY------------------------------------
Hs_LCK                        -------------------------------------------------------GYYNG----------------------------------------
Hs_LYN                        -------------------------------------------------------GYYNN----------------------------------------
Hs_NEK1                       -------------------------------------------------------VKSTEDGRQ------------------------------------
Hs_PAK1                       -------------------------------------------------------AMDVATGQE------------------------------------
PfPK8(PF3D7_0203100)          -------------------------------------------------------NNISNENNYNDDD--------------------------------
Hs_PKAca                      -------------------------------------------------------VKHKETGNH------------------------------------
Hs_RAF1                       -------------------------------------------------------GKWHG----------------------------------------
Hs_RIOK1                      -------------------------------------------------------ASTANGESR------------------------------------
Hs_RIOK2                      -------------------------------------------------------VANEEGQQFALK---------------------------------
Hs_RIOK3                      ----------------------------------------------------AYGGSMEDEKED------------------------------------
Hs_VGFR1                      -------------------------------------------------------ASAFGIKKS------------------------------------
PBANKA_0940100                ----------------------------------------------------------------------------------------------------
PbRIO1(PBANKA_1445600)        NFEINNLINELSEI-SKCVEMNDV--SDCKENIDSDENEEESFEYLSETESLKINDLLRNSEKR------------------------------------
PbRIO2(PBANKA_0521400)        -------------------------------------------------------CKDVNDNLLCLK---------------------------------
PbTKL1(PBANKA_0308500)        -----------------CLY------------I----------------------NNIKKCRNYKMS-NY------------------------------
PbTKL2(PBANKA_0927000)        -------------------------------------------------------GILKNC-I-------------------------------------
PbTKL4(PBANKA_1122700)        ----------------------------------------------------------------------------------------------------
PF3D7_1106800                 ---------------CNNIYDNNI--NDNHIYCNNIND---NHIYCNNIYDHHKNTSLNSKEQNTDH---------------------------------
PfABCk1(PF3D7_0810200)        ----------------------------------------------------------------------------------------------------
PfABCk2(PF3D7_1414500)        ----------------------------------------------------------------------------------------------------
PfARK1(PF3D7_0605300)         -------------------------------------------------------ACERRTNFI------------------------------------
PfARK2(PF3D7_0309200)         -------------------------------------------------------AIIKKEENK------------------------------------
PfARK3(PF3D7_1356800)         -------------------------------------------------------MRFLTFPQY------------------------------------
PfCDPK1(PF3D7_0217500)        -------------------------------------------------------CREKHGHGE------------------------------------
PfCDPK2(PF3D7_0610600)        -------------------------------------------------------GIDKVTNQL------------------------------------
PfCDPK3(PF3D7_0310100)        -------------------------------------------------------ATDKLLKIS------------------------------------
PfCDPK4(PF3D7_0717500)        -------------------------------------------------------SRDKHTGHE------------------------------------
PfCDPK5(PF3D7_1337800)        -------------------------------------------------------AVSKRTGQQ------------------------------------
PfCDPK6(PF3D7_1122800)        -------------------------------------------------------VEDNICKLY------------------------------------
PfCDPK7(PF3D7_1123100)        -------------------------------------------------------GINKQTNSE------------------------------------
PfCK1.1(PF3D7_1136500.2)      -------------------------------------------------------AKDIVTMEE------------------------------------
PfCK2alpha(PF3D7_1108400)     -------------------------------------------------------GYDTECNRP------------------------------------
PfCLK1(PF3D7_1445400)         -------------------------------------------------------CQHIDNKKY------------------------------------
PfCLK3(PF3D7_1114700)         -------------------------------------------------------CYDMVNKIP------------------------------------
PfCRK1(PF3D7_0417800)         -------------------------------------------------------AQNKRTKKI------------------------------------
PfCRK3(PF3D7_0415300)         ----------------------------------------------------------------------------------------------------
PfCRK4(PF3D7_0317200)         -------------------------------------------------------SCRKKMRKG------------------------------------
PfCRK5(PF3D7_0615500)         ----------------------------------------------------------------------------------------------------
```

```
PfGSK3(PF3D7_0312400)               -------------------------------------------------------AICIDTSEQ------------------------------------
PfIK1(PF3D7_1444500)                -------------------------------------------------------KSENIKSEN------------------------------------
PfKIN(PF3D7_1454300)                -------------------------------------------------------GIHIYTHEI------------------------------------
PfMAPK1(PF3D7_1431500)              -------------------------------------------------------GRCKKNKNI------------------------------------
PfMAPK2(PF3D7_1113900)              -------------------------------------------------------AYDKNANKN------------------------------------
PfMRK(PF3D7_1014400)                -------------------------------------------------------AYDTILKKE------------------------------------
PfNEK1(PF3D7_1228300)               -------------------------------------------------------VKHKRTQEF------------------------------------
PfNEK2(PF3D7_0525900)               -------------------------------------------------------VKDENEK--------------------------------------
PfNEK3(PF3D7_1201600)               ----------------------------------------------------------------------------------------------------
PfNEK4(PF3D7_0719200)               -------------------------------------------------------VRD-RKNDH------------------------------------
PfPK1(PF3D7_0821100)                ----------------------------------------------------------------------------------------------------
PfPK2(PF3D7_1238900)                -------------------------------------------------------GTNILYGNR------------------------------------
PfPK4(PF3D7_0628200)                ----------------------------------------------------------------------------------------------------
PfPK5(PF3D7_1356900)                -------------------------------------------------------AQNN-YGET------------------------------------
PfPK6(PF3D7_1337100)                -------------------------------------------------------ALDKKENNF------------------------------------
PfPK7(PF3D7_0213400)                -------------------------------------------------------CE--KDNKF------------------------------------
PfPK9(PF3D7_1315100)                -------------------------------------------------------GVHIQTKQK------------------------------------
PfPKA(PF3D7_0934800)                -------------------------------------------------------ATYK-NGNYP-----------------------------------
PfPKB(PF3D7_1246900)                -------------------------------------------------------VKHVQNKKL------------------------------------
PfPKG(PF3D7_1436600)                -------------------------------------------------------VHHKPTKIR------------------------------------
PfPutativeCaMK2(PF3D7_1423600)      -------------------------------------------------------CINKMTKEV------------------------------------
PfPutativeK(PF3D7_0107600)          ----------------------------------------------------------------------------------------------------
PfPutativeK(PF3D7_0321400)          ----------------------------------------------------------------------------------------------------
PfPutativeK(PF3D7_0926100)          -------------------------------------------------------GWYKGMH--------------------------------------
PfPutativeK(PF3D7_1145200)          -------------------------------------------------------VKHKITNEI------------------------------------
PfPutativeK(PF3D7_1148000)          -------------------------------------------------------VFDSINLEM------------------------------------
PfPutativeK(PF3D7_1316000)          -------------------------------------------------------AIDMSTQEA------------------------------------
PfPutativeK(PF3D7_1331000)          ----------------------------------------------------------QQSKEF------------------------------------
PfPutativeK(PF3D7_1441300)          -------------------------------------------------------ALDKSTDTF------------------------------------
PfRIO1(PF3D7_1230900)               NNNENMIYNQMENIHDQCSDVHNYMSQDSERDINNYEQSEDTHFYNNDKYYNGFFNKNKNNKGDQSGDDTNNDIYNDIYNDIYNDIYDDLYNDIYNDIYN
PfRIO2(P3D7_0420100)                -------------------------------------------------------CKDVDDNLLCLK---------------------------------
PfSRPK1(PF3D7_0302100)              ----------------------------------------------------------------------------------------------------
PfSRPK2(PF3D7_1443000)              --------------------------------------------Y--------------NTKQF------------------------------------
PfTKL1(PF3D7_0211700)               -----------------CIYGN---------------------------------KNILRINKF------------------------------------
PfTKL2(PF3D7_1121300)               -------------------------------------------------------GVLKNC-I-------------------------------------
PfTKL3(PF3D7_1349300)               ----------------------------------------------------------------------------------------------------
PfTKL4(PF3D7_0623800)               ----------------------------------------------------------------------------------------------------
PfVPS15(PF3D7_0823000)              --------------------------------------------F-------------------------------------------------------
PVX_091015                          ----------------------------------------------------------------------------------------------------
Py02791                             ----------------------------------------------------------------------------------------------------
TGGT1_209050 (TgTKL5)               -------------------------------------------------------ATWRGQ---------------------------------------
TGGT1_225770 (TgTKL8)               -------------------------------------------------------GVLRDGV--------------------------------------
TGGT1_234970 (TgTKL2)               -------------------------------------------------------VFDPLTCDV------------------------------------
TGGT1_236240 (TgTKL6)               -------------------------------------------------------VRDVSSGET------------------------------------
TGGT1_237210 (TgTKL4)               -------------------------------------------------------ATWRDGE--------------------------------------
TGGT1_239130 (TgTKL7)               ---------------CPCMQAYPL-----PPPVPAFP------------QPRCCEGFASGSASFSGDM--------------------------------
TGGT1_253860 (TgTKL3)               ---------------------------------------------------ETHATQDEEDSQK------------------------------------
TGGT1_290225                        ----------------------------------------------------------------------------------------------------
TGGT1_301270 (TgTKL1)               --------------------------------------------------GRAVGAARRKPRER------------------------------------

                                             210       220       230       240       250       260       270       280       290       300
                                    ....|....|....|....|....|....|....|....|....|....|....|....|....|....|....|....|....|....|....|....|
At_CDPK1                            ------------------------------------------------------------------LACKS-I---------------------------
At_CTR1                             -----------------------------------------------------------------DVAVKI-L---------------------------
FIKK1(PF3D7_0102600)                ----------------------------------------------------------------------------------------------------
FIKK10.1(PF3D7_1016400)             ----------------------------------------------------------------------------------------------------
FIKK10.2(PF3D7_1039000)             --------------------------------------------------------------------------VVY-----------------------
FIKK11(PF3D7_1149300)               -----------------------------------------------------------------FIAPKL-YKILY-----------------------
FIKK12(PF3D7_1200800)               ------------------------------------------------------------------IAPKL-LKILY-----------------------
FIKK3(PF3D7_0301200)                ------------------------------------------------------------------IAPKF-YKLLY-----------------------
FIKK4.1(PF3D7_0424500)              ----------------------------------------------------------------------------------------------------
FIKK5(PF3D7_0500900)                ------------------------------------------------------------------ITAKL-YALLY-----------------------
FIKK7.1(PF3D7_0726200)              ---------------------------------------------------------------------KY-MEDII-----------------------
FIKK8(PF3D7_0805700)                ------------------------------------------------------------------ITPKL-YKILY-----------------------
FIKK9.1(PF3D7_0902000)              ----------------------------------------------------------------------------------------------------
FIKK9.2(PF3D7_0902100)              ----------------------------------------------------------------------------------------------------
FIKK9.4(PF3D7_0902300)              ----------------------------------------------------------------------------------------------------
FIKK9.5(PF3D7_0902400)              ------------------------------------------------------------------------YRLLY-----------------------
FIKK9.6(PF3D7_0902500)              ----------------------------------------------------------------------------------------------------
FIKK9.7(PF3D7_0902600)              ------------------------------------------------------------------ISPHF-YTLLY-----------------------
Hs_ABL1                             --------------------------------------------------------------YSLTVAVKT-L---------------------------
Hs_BTK                              --------------------------------------------------------------QY-DVAIKM-I---------------------------
Hs_CAMK1                            ------------------------------------------------------------------VAIKC-I---------------------------
Hs_CDK2                             ------------------------------------------------------------------VALKK-I---------------------------
Hs_CK1                              ------------------------------------------------------------------VAVKL-----------------------------
Hs_CLK1                             ------------------------------------------------------------------VAVKI-VK--------------------------
Hs_ERK1                             ------------------------------------------------------------------VAIKK-I---------------------------
Hs_FGFR1                            -----------------------------------------------------------KPNRVTKVAVKM-L---------------------------
Hs_GCN2_2                           ------------------------------------------------------------------YAVKR-IPI-------------------------
Hs_IGFR1                            -----------------------------------------------------------E-PET-RVAIKT-V---------------------------
Hs_IRAK1                            ------------------------------------------------------------------YAVKR-L---------------------------
Hs_JAK2_1                           ----------------------------------------------------------GQLHET-EVLLKV-LD--------------------------
Hs_LCK                              --------------------------------------------------------------HT-KVAVKS-L---------------------------
Hs_LYN                              --------------------------------------------------------------ST-KVAVKT-L---------------------------
Hs_NEK1                             ------------------------------------------------------------------YVIKE-I---------------------------
Hs_PAK1                             ------------------------------------------------------------------VAIKQ-M---------------------NLQQQP
PfPK8(PF3D7_0203100)                -----------------------------------------------------------SYDEDNLVSLKI-I---------------------------
Hs_PKAca                            ------------------------------------------------------------------YAMKI-L---------------------------
Hs_RAF1                             -----------------------------------------------------------------DVAVKI-L---------------------------
Hs_RIOK1                            -------------------------------------------------------------------AIKI-Y---------------------------
Hs_RIOK2                            ------------------------------------------------------------LHRLGRTSFRN-L---------------------------
Hs_RIOK3                            -----------------------------------------------------------SKVIPTECAIKV-F---------------------------
Hs_VGFR1                            -----------------------------------------------------------PTCRT--VAVKM-L---------------------------
PBANKA_0940100                      -----------------------------------------------------------------QNLLKN-----------------------------
PbRIO1(PBANKA_1445600)              ---EIIEFENNKTEIIDIKNEIN-----VFDIIDQLDKLIYKDE-----NYISDILE--NKKIAVSFATKI-Y---------------------------
PbRIO2(PBANKA_0521400)              ------------------------------------------------------------IHRLGRISFRT-I---------------------------
```

```
PbTKL1(PBANKA_0308500)             ---------------------------RNFN----------------------------INHSINNIALKICID--------------------------
PbTKL2(PBANKA_0927000)             -----------------------------------------------------------------NVAIKV-L---------------------------
PbTKL4(PBANKA_1122700)             --------------------------------------------------------------QI-PVAIKI--------------------------HDL
PF3D7_1106800                      -----------------------------------------------------------NIEQI-NECNKY-A---------------------------
PfABCk1(PF3D7_0810200)             ---------------------------------------------------------------------KK-----------------------------
PfABCk2(PF3D7_1414500)             ----------------------------------------------------------------------M-----------------------------
PfARK1(PF3D7_0605300)              ------------------------------------------------------------------CVLKC-I---------------------------
PfARK2(PF3D7_0309200)              -----------------------------------------------------------------KVALKV-M---------------------------
PfARK3(PF3D7_1356800)              ------------------------------------------------------------------YAIKK-I---------------------------
PfCDPK1(PF3D7_0217500)             ------------------------------------------------------------------KAIKV-I---------------------------
PfCDPK2(PF3D7_0610600)             ------------------------------------------------------------------YAIKE-E---------------------------
PfCDPK3(PF3D7_0310100)             ------------------------------------------------------------------RAVKV-V---------------------------
PfCDPK4(PF3D7_0717500)             ------------------------------------------------------------------YAIKV-I---------------------------
PfCDPK5(PF3D7_1337800)             ------------------------------------------------------------------RAIKI-I---------------------------
PfCDPK6(PF3D7_1122800)             ------------------------------------------------------------------KVVKI-L---------------------------
PfCDPK7(PF3D7_1123100)             ------------------------------------------------------------------FAIKV-I---------------------------
PfCK1.1(PF3D7_1136500.2)           ------------------------------------------------------------------FAVKL-----------------------------
PfCK2alpha(PF3D7_1108400)          ------------------------------------------------------------------CAIKV-L---------------------------
PfCLK1(PF3D7_1445400)              ------------------------------------------------------------------YAVKV-VR--------------------------
PfCLK3(PF3D7_1114700)              ------------------------------------------------------------------VAVKV-I---------------------------
PfCRK1(PF3D7_0417800)              ------------------------------------------------------------------VALKKLK---------------------------
PfCRK3(PF3D7_0415300)              ----------------------------------------------------------------------------------------------------
PfCRK4(PF3D7_0317200)              -----------------------------------------------------------------HVRIKENTRTIDK------------------LKYR
PfCRK5(PF3D7_0615500)              ------------------------------------------------------------------YAIKF-FR--------------------------
PfGSK3(PF3D7_0312400)              ------------------------------------------------------------------VAIKK-V---------------------------
PfIK1(PF3D7_1444500)               ------------------------------------------------------------------IKSEN-I---------------------------
PfKIN(PF3D7_1454300)               ------------------------------------------------------------------VAIKI-L---------------------------
PfMAPK1(PF3D7_1431500)             ------------------------------------------------------------------VAVKK-I---------------------------
PfMAPK2(PF3D7_1113900)             ------------------------------------------------------------------VAIKK-V---------------------------
PfMRK(PF3D7_1014400)               ------------------------------------------------------------------VAIKK-M---------------------------
PfNEK1(PF3D7_1228300)              ------------------------------------------------------------------FCWKA-I---------------------------
PfNEK2(PF3D7_0525900)              ----------------------------------------------------------I-------FVIKE-L---------------------------
PfNEK3(PF3D7_1201600)              ------------------------------------------------------------------------I---------------------------
PfNEK4(PF3D7_0719200)              ------------------------------------------------------------------YVMKI-I---------------------------
PfPK1(PF3D7_0821100)               ----------------------------------------------------------------------------------------------------
PfPK2(PF3D7_1238900)               ------------------------------------------------------------------VVVKE-V---------------------------
PfPK4(PF3D7_0628200)               ------------------------------------------------------------------TNNKE-----------------------------
PfPK5(PF3D7_1356900)               ------------------------------------------------------------------FALKK-I---------------------------
PfPK6(PF3D7_1337100)               ------------------------------------------------------------------VAIKKII---------------------------
PfPK7(PF3D7_0213400)               ------------------------------------------------------------------YALKK-Y---------------------------
PfPK9(PF3D7_1315100)               ------------------------------------------------------------------VALKF-I---------------------------
PfPKA(PF3D7_0934800)               -----------------------------------------------------------------PVAIKR-F---------------------------
PfPKB(PF3D7_1246900)               ------------------------------------------------------------------YAMKI-L---------------------------
PfPKG(PF3D7_1436600)               ------------------------------------------------------------------YALKC-V---------------------------
PfPutativeCaMK2(PF3D7_1423600)     ------------------------------------------------------------------YAVKI-I---------------------------
PfPutativeK(PF3D7_0107600)         ------------------------------------------------------------------INKKH-----------------------------
PfPutativeK(PF3D7_0321400)         ---------------------------------------------------------------VNNVMLKGWT---------------------------
PfPutativeK(PF3D7_0926100)         ------------------------------------------------------------------VAVKVPVD--------------------------
PfPutativeK(PF3D7_1145200)         ------------------------------------------------------------------YAAKL-L---------------------------
PfPutativeK(PF3D7_1148000)         ------------------------------------------------------------------YAAKI--------------------------H--
PfPutativeK(PF3D7_1316000)         ------------------------------------------------------------------VAIKR-----------------------------
PfPutativeK(PF3D7_1331000)         ------------------------------------------------------------------IDKKN-IR--------------------------
PfPutativeK(PF3D7_1441300)         ------------------------------------------------------------------VAAKA-I---------------------------
PfRIO1(PF3D7_1230900)              DDDEKQNFSKREDTVLEEKNKSNNTYEQTFDIIKEMNDISFYDNKKSQISHIINILDKTKEKKGIALATKV-Y---------------------------
PfRIO2(P3D7_0420100)               ------------------------------------------------------------IHRLGRISFRT-I---------------------------
PfSRPK1(PF3D7_0302100)             ----------------------------------------------------------------------------------------------------
PfSRPK2(PF3D7_1443000)             ------------------------------------------------------------------DKIKI-I---------------------------
PfTKL1(PF3D7_0211700)              -----------------------------FD----------------------------IHYSINNTALKIFLN--------------------------
PfTKL2(PF3D7_1121300)              -----------------------------------------------------------------NVAIKV-L---------------------------
PfTKL3(PF3D7_1349300)              ------------------------------------------------------------------V---------------------------------
PfTKL4(PF3D7_0623800)              --------------------------------------------------------------KT-PVAIKI--------------------------HDL
PfVPS15(PF3D7_0823000)             -----------------------------------------------------------------QIFLSI-I---------------------------
PVX_091015                         ----------------------------------------------------------------------------------------------------
Py02791                            ----------------------------------------------------------------------------------------------------
TGGT1_209050 (TgTKL5)              -----------------------------------------------------------------TVAVKQ-A---------------------------
TGGT1_225770 (TgTKL8)              -----------------------------------------------------------------AVAVKW-L---------------------------
TGGT1_234970 (TgTKL2)              ------------------------------------------------------------------LAAKL--------------------------H--
TGGT1_236240 (TgTKL6)              ------------------------------------------------------------------FALKR-ILC-------------------------
TGGT1_237210 (TgTKL4)              ------------------------------------------------------------------VACKI-VAFPV---------------------GG
TGGT1_239130 (TgTKL7)              -----------------------------------------------------------------RVAVKV-F---------------------------
TGGT1_253860 (TgTKL3)              -----------------------------------------------------------------ASASRA-TGRPC-------------------P---
TGGT1_290225                       ----------------------------------------------------------------------------------------------------
TGGT1_301270 (TgTKL1)              ----------------------------------------------------------KRSREEQDAGVRE-----------------------ENAVSE

                                            310       320       330       340       350       360       370       380       390       400
                                   ....|....|....|....|....|....|....|....|....|....|....|....|....|....|....|....|....|....|....|....|
At_CDPK1                           SKRK-----------------------------------------------L------RTAVDI------------------------------------
At_CTR1                            MEQD-----------------------------------------------F------HAERV-------------------------------------
FIKK1(PF3D7_0102600)               ----------------------------------------------------------------------------------------------------
FIKK10.1(PF3D7_1016400)            ----------------------------------------------------------------------------------------------------
FIKK10.2(PF3D7_1039000)            EPV--------------------------------------------------------NNNTN------------------------------------
FIKK11(PF3D7_1149300)              EENY-------------------------------------------------------EENNK------------------------------------
FIKK12(PF3D7_1200800)              D----------------------------------------------------------GKNVN------------------------------------
FIKK3(PF3D7_0301200)               EED--------------------------------------------------------NKNSS------------------------------------
FIKK4.1(PF3D7_0424500)             ----------------------------------------------------------------------------------------------------
FIKK5(PF3D7_0500900)               EPY--------------------------------------------------------RKYIQ------------------------------------
FIKK7.1(PF3D7_0726200)             EHNK-------------------------------------------------------RNNAN------------------------------------
FIKK8(PF3D7_0805700)               EPD--------------------------------------------------------NKYFN------------------------------------
FIKK9.1(PF3D7_0902000)             ----------------------------------------------------------------------------------------------------
FIKK9.2(PF3D7_0902100)             --------------------------------------------------------------SE------------------------------------
FIKK9.4(PF3D7_0902300)             ----------------------------------------------------------------------------------------------------
FIKK9.5(PF3D7_0902400)             EPHYY-----------------------------------------------------------------------------------------------
FIKK9.6(PF3D7_0902500)             ----------------------------------------------------------------------------------------------------
FIKK9.7(PF3D7_0902600)             EPLDYNENGNENGNENGNGDENGNENGNENGNENGNENGNENGNENGNGNGVENGNENGVENGN------------------------------------
Hs_ABL1                            KEDT------------------------------------------------------MEVE--------------------------------------
Hs_BTK                             KEGS------------------------------------------------------MSED--------------------------------------
```

```
Hs_CAMK1                        AKEA-----------------------------------------------L------EGKE--------------------------------------
Hs_CDK2                         RLDT------------------------------------------------------ETEGVP------------------------------------
Hs_CK1                          ESQKARH---------------------------------------------------------------------------------------------
Hs_CLK1                         NVDR-----------------------------------------------YC-----------------------------------------------
Hs_ERK1                         -SPF------------------------------------------------------EHQTYC------------------------------------
Hs_FGFR1                        KSDA------------------------------------------------------TEKDL-------------------------------------
Hs_GCN2_2                       NPASRQ---------------------------------------------F------------------------------------------------
Hs_IGFR1                        NEAA------------------------------------------------------SMRER-------------------------------------
Hs_IRAK1                        KENAD----------------------------------------------L------EWTAVK------------------------------------
Hs_JAK2_1                       KAHRN----------------------------------------------YS-----------------------------------------------
Hs_LCK                          KQGS------------------------------------------------------MSPD--------------------------------------
Hs_LYN                          KPGT------------------------------------------------------MSVQ--------------------------------------
Hs_NEK1                         NISR-----------------------------------------------M------SSKER-------------------------------------
Hs_PAK1                         KKEL-----------------------------------------------I------IN----------------------------------------
PfPK8(PF3D7_0203100)            NLKY-----------------------------------------------L------SKKNSL------------------------------------
Hs_PKAca                        DKQK-----------------------------------------------V------VKLKQI------------------------------------
Hs_RAF1                         KVVD------------------------------------------------------PTPEQF------------------------------------
Hs_RIOK1                        KTSI-----------------------------------------------L------VFKDRD------------------------------------
Hs_RIOK2                        KNKRD----------------------------------------------YHK----HRHNVSWL---------------------------------Y
Hs_RIOK3                        KTTLN-----------------------------------------------------EFKNRD------------------------------------
Hs_VGFR1                        KEGA------------------------------------------------------TASEY-------------------------------------
PBANKA_0940100                  KISQ------------------------------------------------------------------------------------------------
PbRIO1(PBANKA_1445600)          NTSI-----------------------------------------------L------VFKKRS------------------------------------
PbRIO2(PBANKA_0521400)          KNNRD----------------------------------------------YYG----KKKFRNWL---------------------------------Y
PbTKL1(PBANKA_0308500)          KKYS------------------------------------------------------------------------------------------------
PbTKL2(PBANKA_0927000)          KKNEN-----------------------------------------------------------------------------------------------
PbTKL4(PBANKA_1122700)          KDSKN----------------------------------------------L------------------------------------------------
PF3D7_1106800                   SETK-----------------------------------------------Y-------NIKKS------------------------------------
PfABCk1(PF3D7_0810200)          ---------------------------------------------------F------------------------------------------------
PfABCk2(PF3D7_1414500)          ---------------------------------------------------F------------------------------------------------
PfARK1(PF3D7_0605300)           SKSH-----------------------------------------------L------VKSTQE------------------------------------
PfARK2(PF3D7_0309200)           AKDT-----------------------------------------------I------MSLNIE------------------------------------
PfARK3(PF3D7_1356800)           SKDH-----------------------------------------------I------IKSQAA------------------------------------
PfCDPK1(PF3D7_0217500)          KKSQ-----------------------------------------------F------DKMKYS------------------ITNKI-----------EC
PfCDPK2(PF3D7_0610600)          KKDR-----------------------------------------------L------KNI---------------------------------------
PfCDPK3(PF3D7_0310100)          SKKK-----------------------------------------------L------KNI---------------------------------------
PfCDPK4(PF3D7_0717500)          SKKH-----------------------------------------------V------KRK----------------------TDK--------------
PfCDPK5(PF3D7_1337800)          EKKK-----------------------------------------------I------HNI---------------------------------------
PfCDPK6(PF3D7_1122800)          KKKS-----------------------------------------------M------KHI---------------------------------------
PfCDPK7(PF3D7_1123100)          DKRS-----------------------------------------------V------SIYEK-------------------------------------
PfCK1.1(PF3D7_1136500.2)        ESTRSKH---------------------------------------------------------------------------------------------
PfCK2alpha(PF3D7_1108400)       KPVK-----------------------------------------------------------K------------------------------------
PfCLK1(PF3D7_1445400)           NIKK-----------------------------------------------YT-----------------------------------------------
PfCLK3(PF3D7_1114700)           RDNDM----------------------------------------------MK-----------------------------------------------
PfCRK1(PF3D7_0417800)           NFSS-----------------------------------------------M------HNEGFA------------------------------------
PfCRK3(PF3D7_0415300)           ----------------------------------------------------------EDTTSS------------------------------------
PfCRK4(PF3D7_0317200)           KHSKK----------------------------------------------LK-----KIENKN------------------------------------
PfCRK5(PF3D7_0615500)           DDLRT----------------------------------------------I------NEEGIS------------------------------------
PfGSK3(PF3D7_0312400)           ------------------------------------------------------------LQDP------------------------------------
PfIK1(PF3D7_1444500)            KSEN-----------------------------------------------I------KSEKVK------------------------------------
PfKIN(PF3D7_1454300)            NKKR-----------------------------------------------L------IEI----------------------INY--------------
PfMAPK1(PF3D7_1431500)          FGAF------------------------------------------------------QNCTDA------------------------------------
PfMAPK2(PF3D7_1113900)          NRMF------------------------------------------------------EDLIDC------------------------------------
PfMRK(PF3D7_1014400)            KLNKIS-------------------------------------------NYI------DDCGIN------------------------------------
PfNEK1(PF3D7_1228300)           SYRG-----------------------------------------------L------KEREK-------------------------------------
PfNEK2(PF3D7_0525900)           DISC-----------------------------------------------M------NNKEK-------------------------------------
PfNEK3(PF3D7_1201600)           NEDD-----------------------------------------------L------------------------------------------------
PfNEK4(PF3D7_0719200)           NISQ-----------------------------------------------M------SQKEK-------------------------------------
PfPK1(PF3D7_0821100)            ----------------------------------------------------------------------------------------------------
PfPK2(PF3D7_1238900)            DKSK-----------------------------------------------V------KE----------------------------------------
PfPK4(PF3D7_0628200)            KRKKD-----------------------------------------------------------------------------------------------
PfPK5(PF3D7_1356900)            RLEK------------------------------------------------------EDEGIP------------------------------------
PfPK6(PF3D7_1337100)            NLCD------------------------------------------------------ENYGIS------------------------------------
PfPK7(PF3D7_0213400)            EKSL-----------------------------------------------L------EKKRDFT--KSN-------------NDKI-----------SI
PfPK9(PF3D7_1315100)            PKSN-----------------------------------------------F------LDV----------------------TDV--------------
PfPKA(PF3D7_0934800)            EKCK-----------------------------------------------I------IRQKQV------------------------------------
PfPKB(PF3D7_1246900)            RKEN-----------------------------------------------I------LSRNQL------------------------------------
PfPKG(PF3D7_1436600)            SKRS-----------------------------------------------I------INLNQQ------------------------------------
PfPutativeCaMK2(PF3D7_1423600)  KKKK-----------------------------------------------K------HKKSYN------------------------------------
PfPutativeK(PF3D7_0107600)      KHNKR----------------------------------------------Y------HQIKEK------------------------------------
PfPutativeK(PF3D7_0321400)      ---------------------------------------------------F------------------------------------------------
PfPutativeK(PF3D7_0926100)      KMAR------------------------------------------------------QDPYGLT-----------------------------------
PfPutativeK(PF3D7_1145200)      QPNQ-----------------------------------------------F------PKESFN------------------------------------
PfPutativeK(PF3D7_1148000)      KIEPS----------------------------------------------M------SNEIKN------------------------------------
PfPutativeK(PF3D7_1316000)      -----------------------------------------------------------SPKWR------------------------------------
PfPutativeK(PF3D7_1331000)      TQNKS----------------------------------------------II-----ERDNKK------------------------------------
PfPutativeK(PF3D7_1441300)      DKST-----------------------------------------------V------QGDIGLF-----------------------------------
PfRIO1(PF3D7_1230900)           NTSI-----------------------------------------------L------VFKKRS------------------------------------
PfRIO2(P3D7_0420100)            KNNRD----------------------------------------------YYG----KKCFRNWL---------------------------------Y
PfSRPK1(PF3D7_0302100)          ----------------------------------------------------------------------------------------------------
PfSRPK2(PF3D7_1443000)          DFNSC----------------------------------------------I------------------------------------------------
PfTKL1(PF3D7_0211700)           KKKN-----------------------------------------------I------------------------------------------------
PfTKL2(PF3D7_1121300)           KKNEN-----------------------------------------------------------------------------------------------
PfTKL3(PF3D7_1349300)           ----------------------------------------------------------------------------------------------------
PfTKL4(PF3D7_0623800)           KDSK---------------------------------------------N-L------------------------------------------------
PfVPS15(PF3D7_0823000)          QLHSLGI----------------------------------------YHGHI------KRENVLIQ----------------------------------
PVX_091015                      ----------------------------------------------------------------------------------------------------
Py02791                         ----------------------------------------------------------------------------------------------------
TGGT1_209050 (TgTKL5)           HGVLT------------------------------------------------------QEALR------------------------------------
TGGT1_225770 (TgTKL8)           HKPRE------------------------------------------------------A----------------------------------------
TGGT1_234970 (TgTKL2)           VLSS-----------------------------------------------V------EKESARW-----------------------------------
TGGT1_236240 (TgTKL6)           QEKER----------------------------------------------Y------------------------------------------------
TGGT1_237210 (TgTKL4)           SPAS------------------------------------------------------RAPQIR------------------------------------
TGGT1_239130 (TgTKL7)           RQRELRT--------------------------------------------LQ-----------------------------------------------
TGGT1_253860 (TgTKL3)           ETQGR----------------------------------------------VV----PKLDYFRYC----------------------------------
TGGT1_290225                    ----------------------------------------------------------------------------------------------------
```

```
TGGT1_301270 (TgTKL1)              EEERG--------------------------------------------KEVHSG--EERAEAPWRRETDEDGEADEAGERGEADEACRGDSGEPAEPEY

                                            410       420       430       440       450       460       470       480       490       500
                                   ....|....|....|....|....|....|....|....|....|....|....|....|....|....|....|....|....|....|....|....|
At_CDPK1                           --------EDVRREVAI-------------------------------------------MSTLP----E-HPN-VVKLKA-------------------
At_CTR1                            --------NEFLREVAI-------------------------------------------MKRL-----R-HPN-IVLFMG-------------------
FIKK1(PF3D7_0102600)               -------------KYVL-H-------------------------IDRN------------LDTF-------NER-LREEVN-------------------
FIKK10.1(PF3D7_1016400)            ----------------------------------------------------------------------------------------------------
FIKK10.2(PF3D7_1039000)            --------NNNDEKNNKKHSNDKKSKEDNDDMGYHTGDENDIFEDCNDSNSDRPIDGYDHLKKF-------NDM-LTKQLN-------------------
FIKK11(PF3D7_1149300)              --------ENMFPPYMFNEK-----KELN----------------INN------------LHEF-------KNF-LKERIN-------------------
FIKK12(PF3D7_1200800)              --------HDIMEEYKF-----------------------------KD------------IYEF-------NNM-LIERIN-------------------
FIKK3(PF3D7_0301200)               --------DCILDNEIF-----------------------------YD------------LNLF-------NDF-LCEKLN-------------------
FIKK4.1(PF3D7_0424500)             --------------------------------------------KCNN------------LKKF-------NDM-LINQLQ-------------------
FIKK5(PF3D7_0500900)               --------EDSPPKSSF-----------------------------EN------------IDSF-------NEM-LEEKIK-------------------
FIKK7.1(PF3D7_0726200)             --------NNVDNNNNI-HN-----HKNNIN-----------YCITNS------------DNKH-------DNN-NNDNNS-------------------
FIKK8(PF3D7_0805700)               --------EGLSQKSMY-----------------------------NN------------LNVF-------NDM-LSERLK-------------------
FIKK9.1(PF3D7_0902000)             --------ENVSSDYMF-----------------------------SS------------LDNF-------NKI-LSNGLE-------------------
FIKK9.2(PF3D7_0902100)             --------LKGLNELMF-----------------------------CD------------IDIF-------KNE-LIKIRN-------------------
FIKK9.4(PF3D7_0902300)             ---------NISTSHMS-----------------------------KD------------IISF-------NYI-LNRMLI-------------------
FIKK9.5(PF3D7_0902400)             --------FNLKSISCFEN--------------------------IND------------INIF-------NNL-LREHLK-------------------
FIKK9.6(PF3D7_0902500)             ----------------------------------------------------------------------------------------------------
FIKK9.7(PF3D7_0902600)             --------ENGNAIRSY---------ENN----------------IPN------------LNVF-------NDI-LKEQNK-------------------
Hs_ABL1                            ---------EFLKEAAV-------------------------------------------MKEI-----K-HPN-LVQLLG-------------------
Hs_BTK                             ---------EFIEEAKV-------------------------------------------MMNL-----S-HEK-LVQLYG-------------------
Hs_CAMK1                           --------GSMENEIAV-------------------------------------------LHKI-----K-HPN-IVALDD-------------------
Hs_CDK2                            --------STAIREISL-------------------------------------------LKEL-----N-HPN-IVKLLD-------------------
Hs_CK1                             --------PQLLYESKL-------------------------------------------YKIL-----QGGVG-IPHIRW-------------------
Hs_CLK1                            --------EAARSEIQV-------------------------------------------LEHLNTTDPNSTFR-CVQMLE-------------------
Hs_ERK1                            --------QRTLREIQI-------------------------------------------LLRF-----R-HEN-VIGIRD-------------------
Hs_FGFR1                           --------SDLISEMEM-------------------------------------------MKMIG----K-HKN-IINLLG-------------------
Hs_GCN2_2                          --------RRIKGEVTL-------------------------------------------LSRL-----H-HEN-IVRYYNAWIERHERPAGPGTPPPDS
Hs_IGFR1                           --------IEFLNEASV-------------------------------------------MKEF-----N-CHH-VVRLLG-------------------
Hs_IRAK1                           --------QSFLTEVEQ-------------------------------------------LSRF-----R-HPN-IVDFAG-------------------
Hs_JAK2_1                          --------ESFFEAASM-------------------------------------------MSKL-----S-HKH-LVLNYG-------------------
Hs_LCK                             ---------AFLAEANL-------------------------------------------MKQL-----Q-HQR-LVRLYA-------------------
Hs_LYN                             ---------AFLEEANL-------------------------------------------MKTL-----Q-HDK-LVRLYA-------------------
Hs_NEK1                            --------EESRREVAV-------------------------------------------LANM-----K-HPN-IVQYRE-------------------
Hs_PAK1                            -------------EILV-------------------------------------------MREN-----K-NPN-IVNYLD-------------------
PfPK8(PF3D7_0203100)               --------KNILREVNF-------------------------------------------LKMC-----E-HPN-VVKYFE-------------------
Hs_PKAca                           --------EHTLNEKRI-------------------------------------------LQAV-----N-FPF-LVKLEF-------------------
Hs_RAF1                            --------QAFRNEVAV-------------------------------------------LRKT-----R-HVN-ILLFMG-------------------
Hs_RIOK1                           --------KYVSGEFRF-------------------------------------------RHGYC----KGNPRKMVKTWA-------------------
Hs_RIOK2                           LSR-----LSAMKEFAY-------------------------------------------MKALY----E-RKFPVPKPID-------------------
Hs_RIOK3                           --------KYIKDDFRF-------------------------------------------KDRFS----KLNPRKIIRMWA-------------------
Hs_VGFR1                           --------KALMTELKI-------------------------------------------LTHIG----H-HLN-VVNLLG-------------------
PBANKA_0940100                     --------KKINKKLNF-------------------------------------------KAKI-------KIN-RPYAFP-------------------
PbRIO1(PBANKA_1445600)             --------KYIEGEFRF-------------------------------------------RNAYTK---NTNPRKMVKQWS-------------------
PbRIO2(PBANKA_0521400)             LSK-----IAATKEYAY-------------------------------------------LKALY----E-NDFPVPKPYD-------------------
PbTKL1(PBANKA_0308500)             --------YDFFSELKI-------------------------------------------LSIL-----R-HPN-VSLFLG-------------------
PbTKL2(PBANKA_0927000)             --------NGFENELII-------------------------------------------MSRY-----R-HNN-ILSLLG-------------------
PbTKL4(PBANKA_1122700)             --------KNFLREIEI-------------------------------------------YKNL-----Q-RSN-ICKFYG-------------------
PF3D7_1106800                      ---------NLKNNIIS-------------------------------------------HKNFQKCNQI-QMN-QPYTFP-------------------
PfABCk1(PF3D7_0810200)             --------DYFNYEPFA-------------------------------------------SASIG----QVHDA-IINKKKK------------------
PfABCk2(PF3D7_1414500)             --------EYIDKEPLA-------------------------------------------SASIG----QVHKA-KLKKYDKH-----------------
PfARK1(PF3D7_0605300)              --------ALLRKEIEL-------------------------------------------QAHL-----K-HPH-IACMYT-------------------
PfARK2(PF3D7_0309200)              --------RQVLKEIII-------------------------------------------QASL-----K-HIN-ILELIA-------------------
PfARK3(PF3D7_1356800)              --------GQAYLEAKY-------------------------------------------HSVL-----S-HVN-IIKMYG-------------------
PfCDPK1(PF3D7_0217500)             DDKI---HEEIYNEISL-------------------------------------------LKSL-----D-HPN-IIKLFD-------------------
PfCDPK2(PF3D7_0610600)             --------NRFFQEIEI-------------------------------------------MKKL-----D-HPN-IVKLYE-------------------
PfCDPK3(PF3D7_0310100)             --------PRFRQEIDI-------------------------------------------MKNL-----D-HPN-VVKLLE-------------------
PfCDPK4(PF3D7_0717500)             --------ESLLREVEL-------------------------------------------LKML-----D-HIN-IMKLYE-------------------
PfCDPK5(PF3D7_1337800)             --------ERLKREILI-------------------------------------------MKQM-----D-HPN-IIKLYE-------------------
PfCDPK6(PF3D7_1122800)             ---------KINEEINV-------------------------------------------LIYL-----D-HPN-IIKIFD-------------------
PfCDPK7(PF3D7_1123100)             --------ELLRSEISI-------------------------------------------LRLL-----R-HPN-VIYLKE-------------------
PfCK1.1(PF3D7_1136500.2)           --------PQLLYESKL-------------------------------------------YKIL-----GGGIG-VPKVYW-------------------
PfCK2alpha(PF3D7_1108400)          --------KKIKREIKI-------------------------------------------LQNL-----NGGPN-IIKLLD-------------------
PfCLK1(PF3D7_1445400)              --------RSAKIEADI-------------------------------------------LKKIQNDDIN-NNN-IVKYHG-------------------
PfCLK3(PF3D7_1114700)              --------KAAEKEISI-------------------------------------------LKKLNQYD-KDNKRHIIRLLS-------------------
PfCRK1(PF3D7_0417800)              --------MTSLREINI-------------------------------------------LLQL-----Q-HDN-ILSIKE-------------------
PfCRK3(PF3D7_0415300)              --------NSSHSNCS-------------------------------------------------------SSS-VSSFMS-------------------
PfCRK4(PF3D7_0317200)              --------NDYIENWDL----------------------------------------FLVIEKC-----DCSLNDILNKVK-------------------
PfCRK5(PF3D7_0615500)              --------CTTLRELSC-------------------------------------------LKNIG----R-HPN-ILRLIDVTIDRQK------------
PfGSK3(PF3D7_0312400)              --------QYKNRELMI-------------------------------------------MKNL-----N-HIN-IIYLKD-------------------
PfIK1(PF3D7_1444500)               --------NLYIEEIKY-------------------------------------------FWNHY----K-KNK-DPNVND-------------------
PfKIN(PF3D7_1454300)               --------DKIIKEIEI-------------------------------------------HKNI-----N-HNH-ICKFYD-------------------
PfMAPK1(PF3D7_1431500)             --------QRTFREIIF-------------------------------------------LYEL-----NGHDN-IIKLMD-------------------
PfMAPK2(PF3D7_1113900)             --------KRILREITI-------------------------------------------LNRL-----K-SDY-IIRLHD-------------------
PfMRK(PF3D7_1014400)               --------FVLLREIKI-------------------------------------------MKEI-----K-HKN-IMSALD-------------------
PfNEK1(PF3D7_1228300)              --------SQLVIEVNV-------------------------------------------MREL-----K-HKN-IVRYID-------------------
PfNEK2(PF3D7_0525900)              --------MNVVNEIRA-------------------------------------------LIKM-----SVHPF-IVRYKE-------------------
PfNEK3(PF3D7_1201600)              --------NKYMNELYI-------------------------------------------MNKL-----RNCEN-IVNIID-------------------
PfNEK4(PF3D7_0719200)              --------RQCLKEVEL-------------------------------------------LSKL-----N-HPF-IVKYIE-------------------
PfPK1(PF3D7_0821100)               ---------------------------------------------------------------L-----D-KL---------------------------
PfPK2(PF3D7_1238900)               --------SNVYTEIEV-------------------------------------------LRKV-----M-HKY-IIKLIS-------------------
PfPK4(PF3D7_0628200)               --------DDINKNMEK-------------------------------------------IKSY-----K-KKT-PVPEFS-------------------
PfPK5(PF3D7_1356900)               --------STTIREISI-------------------------------------------LKEL-----K-HSN-IVKLYD-------------------
PfPK6(PF3D7_1337100)               --------KCILRELTI-------------------------------------------LQKI-----K-HKN-IINLKY-------------------
PfPK7(PF3D7_0213400)               KSKY----DDFKNELQI-------------------------------------------ITDI-----K-NEY-CLTCEG-------------------
PfPK9(PF3D7_1315100)               --------HRVFIEIQT-------------------------------------------LRGL-----I-HNN-IIKMYD-------------------
PfPKA(PF3D7_0934800)               --------DHVFSERKI-------------------------------------------LNYI-----N-HPF-CVNLHG-------------------
PfPKB(PF3D7_1246900)               --------EHTKVERNI-------------------------------------------LKCV-----S-HPF-IVKMYY-------------------
PfPKG(PF3D7_1436600)               --------NNIKLEREI-------------------------------------------TAEN-----D-HPF-IIRLVR-------------------
PfPutativeCaMK2(PF3D7_1423600)     ---F---EKMVKNEIKY-------------------------------------------LSIM-----S-HEN-IIKFKD-------------------
PfPutativeK(PF3D7_0107600)         --------NGNNKETKY------------------------------------------------------KFN--------------------------
PfPutativeK(PF3D7_0321400)         --------HDLLKIRTLN------------------------------------------YECI-----NTHLNYLLRIYN-------------------
```

```
PfPutativeK(PF3D7_0926100)   --------KRSINEWKI-------------------------------------------LAKC-----D-HPN-IIKLCG-------------------
PfPutativeK(PF3D7_1145200)   --RI---VEMFTKEIIN-------------------------------------------LSIC-----Q-CPG-VIKLHK-------------------
PfPutativeK(PF3D7_1148000)   --KI---IQRAENEINI------------------------------------------HIHCH-----R-HIF-IVKLEF-------------------
PfPutativeK(PF3D7_1316000)   --------NKVSREVDL-------------------------------------------LKKM-----NGSTN-IVKIKS-------------------
PfPutativeK(PF3D7_1331000)   --------EINYEEISV---------------------------L--------------PYSDII----------ILKNKN-------------------
PfPutativeK(PF3D7_1441300)   --------EKLKDEIKI-------------------------------------------SCMM-----N-HPN-VVKTLN-------------------
PfRIO1(PF3D7_1230900)        --------QYIEGEFRF-------------------------------------------RNAYTK---NTNPRKMVKQWA-------------------
PfRIO2(P3D7_0420100)         LSR-----IAATKEYAY-------------------------------------------LKVLY----E-NNFPVPKPHD-------------------
PfSRPK1(PF3D7_0302100)       ----------------------------------------------------------------------------------------------------
PfSRPK2(PF3D7_1443000)       -------YESDKLEMYI-------------------------------------------QTRSY----R-SPE-V------------------------
PfTKL1(PF3D7_0211700)        --------LEYFTELYI-------------------------------------------VSNL-----R-HPN-VTLFLG-------------------
PfTKL2(PF3D7_1121300)        --------NGFENEIII-------------------------------------------MSRY-----R-HNN-ILSLLG-------------------
PfTKL3(PF3D7_1349300)        --------KYRNYEAKI-------------------------------------------LYSL-----RECKH-VIKLIG-------------------
PfTKL4(PF3D7_0623800)        --------KNFLREIEI-------------------------------------------YKNI-----Q-RPN-ICTFYG-------------------
PfVPS15(PF3D7_0823000)       -----NNMHIFLTDINI----------------------------LNDY--------LYFIPKIRY--EDERKGRLMKLQE-------------------
PVX_091015                   ---------------------------------------------------------------------L-HLN-LPES---------------------
Py02791                      ----------------------------------------------------------------------------------------------------
TGGT1_209050 (TgTKL5)        ---------SIAREMNS-------------------------------------------FRAMD----A-HPH-IVKYLG-------------------
TGGT1_225770 (TgTKL8)        ---------GFEKEVQV-------------------------------------------LSKF-----R-HPH-LVILLG-------------------
TGGT1_234970 (TgTKL2)        --HI---VKRVQNEIEI-------------------------------------------HKDIL---P--HPH-IVEMKA-------------------
TGGT1_236240 (TgTKL6)        --------RVARAEAKL-------------------------------------------MESL-----PPHPN-IVGFFGAAVET--------------
TGGT1_237210 (TgTKL4)        --QL---IHDFRKELVV-------------------------------------------ISRL-----N-HPN-LVGLRG-------------------
TGGT1_239130 (TgTKL7)        --------RSFFSELSV-------------------------------------------LGRL-----T-HPN-IAMLLG-------------------
TGGT1_253860 (TgTKL3)        -PQP---LKHRDYEADI-------------------------------------------LAAL-----QPHPN-IISLYG-------------------
TGGT1_290225                 ----------------------------------------------------------------------------------------------------
TGGT1_301270 (TgTKL1)        ALKLVPKSEENLPEAEI-------------------------------------------MRSY-----S-HPR-VVPFLG-------------------

                                      510       520       530       540       550       560       570       580       590       600
                             ....|....|....|....|....|....|....|....|....|....|....|....|....|....|....|....|....|....|....|....|

At_CDPK1                     --SY-----------EDNEN------------------VHL------VMELCE----------------------------GGE--L-FDRIV-------
At_CTR1                      --AV------------TQPPN-----------------LSI------VTEYLS----------------------------RGS--L-YRLLH-------
FIKK1(PF3D7_0102600)         --------------NNKKGY------------------VVM------VCEFF-----------------------------GED--I-FDYTI-------
FIKK10.1(PF3D7_1016400)      ---------------------------------------VI------ISELF-----------------------------GED--I-YTYLT-------
FIKK10.2(PF3D7_1039000)      --------------NNKKGY------------------VVF------ISELY-----------------------------GQD--L-FQYIN-------
FIKK11(PF3D7_1149300)        --------------KNVNGY------------------IVI------VSELY-----------------------------GQN--V-FEYIE-------
FIKK12(PF3D7_1200800)        --------------NNMDGY------------------IVM------VSELF-----------------------------GED--L-FDFNK-------
FIKK3(PF3D7_0301200)         --------------TNINGN------------------IVM------ISEYY-----------------------------GED--I-FDFIL-------
FIKK4.1(PF3D7_0424500)       --------------LNNKAN------------------VIM------ISELY-----------------------------GED--V-FKYVK-------
FIKK5(PF3D7_0500900)         --------------NNKKGN------------------VVL------IYELF-----------------------------GES--L-FTNLV-------
FIKK7.1(PF3D7_0726200)       --------------DNNCGY------------------VVM------VSEYY-----------------------------GED--I-FDFII-------
FIKK8(PF3D7_0805700)         --------------CNVGGN------------------IVI------VSEFF-----------------------------NED--I-LDFID-------
FIKK9.1(PF3D7_0902000)       --------------KNLDGN------------------IVI------VSELY-----------------------------GDD--I-KNYKK-------
FIKK9.2(PF3D7_0902100)       --------------RNKKGY------------------VVM------IWEFF-----------------------------GQN--L-KEFLH-------
FIKK9.4(PF3D7_0902300)       --------------KNMKGY------------------VVM------VSEYY-----------------------------GKD--T-YKYLR-------
FIKK9.5(PF3D7_0902400)       --------------MNMIGY------------------VVM------ISEFF-----------------------------GED--L-ENYIY-------
FIKK9.6(PF3D7_0902500)       --------------------------------------ILM------VSESF-----------------------------GED--L-ETYLC-------
FIKK9.7(PF3D7_0902600)       --------------HKSIGN------------------IVM------VFEFF-----------------------------GED--L-DCFMN-------
Hs_ABL1                      --VC----------T-REPP------------------FYI------ITEFMT----------------------------YGN--L-LDYL--------
Hs_BTK                       --VC----------T-KQRP------------------IFI------ITEYMA----------------------------NGC--L-LNYL--------
Hs_CAMK1                     --IY-----------ESGGH------------------LYL------IMQLVS----------------------------GGE--L-FDRIV-------
Hs_CDK2                      --VI-----------HTENK------------------LYL------VFEFLH----------------------------Q-D--L-KKFMD-------
Hs_CK1                       ------Y-------GQEKDY------------------NVL------VMDLLGPS--------------------------LED--L-FNFCS-------
Hs_CLK1                      --WF----------E-HHGH------------------ICI------VFELLG----------------------------LST----YDFI--------
Hs_ERK1                      --IL-----------RASTLEAM--RD-----------VYI------VQDLME----------------------------T-D--L-YKLLK-------
Hs_FGFR1                     --AC----------T-QDGP------------------LYV------IVEYAS----------------------------KGN--L-REYL--------
Hs_GCN2_2                    GPLAKDD--RAARGQPASDTDGLDSVE------AAAPPPILSS----SVEWSTSGERSASARFPATGPGSSDDEDDDEDEHGGV--FSQSFLPASDSESD
Hs_IGFR1                     --VV----------S-QGQP------------------TLV------IMELMT----------------------------RGD--L-KSYL--------
Hs_IRAK1                     --YC----------A-QNGF------------------YCL------VYGFLP----------------------------NGS--L-EDRL--------
Hs_JAK2_1                    --VCV-----------CGDE------------------NIL------VQEFVK----------------------------FGS--L-DTYLK-------
Hs_LCK                       --VV----------T--QEP------------------IYI------ITEYME----------------------------NGS--L-VDFL--------
Hs_LYN                       --VV----------T-REEP------------------IYI------ITEYMA----------------------------KGS--L-LDFL--------
Hs_NEK1                      --SF-----------EENGS------------------LYI------VMDYCE----------------------------GGD--L-FKRINA------
Hs_PAK1                      --SY-----------LVGDE------------------LWV------VMEYLA----------------------------GGS--L-TDVVT-------
PfPK8(PF3D7_0203100)         --SFF------------------------------WPPCYLV----IVCEYLS----------------------------GGT--L-YDLYK-------
Hs_PKAca                     --SF-----------KDNSN------------------LYM------VMEYVP----------------------------GGE--M-FSHLR-------
Hs_RAF1                      --YM------------TKDN------------------LAI------VTQWCE----------------------------GSS--L-YKHLHV------
Hs_RIOK1                     ----------------EKEMRNL--IRLNTAEIPCPEPIMLRS-HVLVMSFIG----------------------------KDD--MPAPLL--------
Hs_RIOK2                     --YN----------R-----------------------HAV------VMELIN----------------------------GYP--L-------------
Hs_RIOK3                     ----------------EKEMHNL--ARMQRAGIPCPTVVLLKK-HILVMSFIG----------------------------HDQ--VPAPKL--------
Hs_VGFR1                     --AC----------TKQGGP------------------LMV------IVEYCK----------------------------YGN--L-SNYL--------
PBANKA_0940100               --PL------------QEDFN-----------------FYL--------EKKK----------------------------KKKKIL-FSYL--------
PbRIO1(PBANKA_1445600)       ----------------EKEFRNL--RRILIHGLRCPYPLVLKS-NFIVMSMLG----------------------------NLD--VSCSKM--------
PbRIO2(PBANKA_0521400)       --LN----------R-----------------------HMI------LMSYVN----------------------------GYP--L-------------
PbTKL1(PBANKA_0308500)       --------------G-IRDP------------------QAI------ALEYIP----------------------------YGS--I-FDIL--------
PbTKL2(PBANKA_0927000)       --YA----------M-NTNY------------------FYL------IYEYVS----------------------------LGD--L-RTLLFNHYY---
PbTKL4(PBANKA_1122700)       --IC----------I-KHNK------------------LML------LLEYYA----------------------------KGN--L-FNFL--------
PF3D7_1106800                --PY----------Q-KELS------------------SYL------KNEKIK----------------------------RKRKVL-FSYL--------
PfABCk1(PF3D7_0810200)       --------------------------------------VAV------KIQYPG---------------------------------V-YESID-------
PfABCk2(PF3D7_1414500)       --ICD------IDSRKHKDY-----------------NVII------KIQHEG---------------------------------I-DQFLS-------
PfARK1(PF3D7_0605300)        --WF-----------HTSSH------------------VFF------VMEYCS----------------------------NGD--L-FTYLN-------
PfARK2(PF3D7_0309200)        --YF-----------EDKTR------------------LFL------ILELAN----------------------------GGS--V-RNKMKQ------
PfARK3(PF3D7_1356800)        --CM-----------QDEEY------------------IYH------VLEFCS----------------------------KGS--I-YSISKN------
PfCDPK1(PF3D7_0217500)       --VF-----------EDKKY------------------FYL------VTEFYE----------------------------GGE--L-FEQII-------
PfCDPK2(PF3D7_0610600)       --TY-----------ENDNY------------------IYL------IMELCS----------------------------GRE--L-FDSII-------
PfCDPK3(PF3D7_0310100)       --TF-----------EDSNQ------------------IYL------VMELCT----------------------------GGE--L-FDKIV-------
PfCDPK4(PF3D7_0717500)       --FF-----------EDNNY------------------YYL------VSDVYT----------------------------GGE--L-FDEII-------
PfCDPK5(PF3D7_1337800)       --VY-----------EDNEK------------------LYL------VLELCD----------------------------GGE--L-FDKIV-------
PfCDPK6(PF3D7_1122800)       --VY-----------ENVDC------------------TYI------VMELCE----------------------------GGE--L-MSRIK-------
PfCDPK7(PF3D7_1123100)       --II-----------NTKET------------------LYI------SMELVK----------------------------GGE--L-YDFLL-------
PfCK1.1(PF3D7_1136500.2)     ------Y-------GIEGDF------------------TIM------VLDLLGPS--------------------------LED--L-FTLCN-------
PfCK2alpha(PF3D7_1108400)    --IV-----------KDPVT-----KT-----------PSL------IFEYIN----------------------------N-I--D-FKTLY-------
PfCLK1(PF3D7_1445400)        --KFM-----------YYDH------------------MCL------IFEPLGPS-------------------------------L-YEIIT-------
PfCLK3(PF3D7_1114700)        --SI----------K-YKNH------------------LCL------VFEWM-----------------------------WGN--L-RIAL--------
PfCRK1(PF3D7_0417800)        --VV-----------FGKHL-----ND-----------IYL------VMEYIE----------------------------H-E--L-KMILD-------
PfCRK3(PF3D7_0415300)        --YD-----------KNKEK-----KS----------CIWM------VFEYVP----------------------------F-D--L-SGYSELLREERN
PfCRK4(PF3D7_0317200)        ----------------KKHS------------------LFIQHIKQCTAQYLPNERID----------------------MTYDH-I-RNYVKYVYLP--
```

```
PfCRK5(PF3D7_0615500)            --LISEYINRQILQHYTSNYHHHHHSKLDITPLAADQKFIF-----AAYEYCD----------------------------GGD--L-KKLIQ-------
PfGSK3(PF3D7_0312400)            --YY-----------YTESFKKN-EKN-----------IFLN----VVMEYIP----------------------------Q-T--V-HKYMKY------
PfIK1(PF3D7_1444500)             --KY----------------------------------LYI------LMEYCP----------------------------GKT--L-REAI--------
PfKIN(PF3D7_1454300)             --VY-----------QNKNN------------------IYM------ILEYVE----------------------------NGN--L-LTYIY-------
PfMAPK1(PF3D7_1431500)           --VI-----------KAKNDN-----D-----------IYL------IFDFME----------------------------T-D--L-HEVIK-------
PfMAPK2(PF3D7_1113900)           --LI-----------IPEDLLKF--DE-----------LYI------VLEIAD----------------------------S-D--L-KKLFK-------
PfMRK(PF3D7_1014400)             --LY-----------CEKDY------------------INL------VMEIMD----------------------------Y-D--L-SKIIN-------
PfNEK1(PF3D7_1228300)            --RFL---------NKANQK------------------LYI------LMEFCD----------------------------AGD--L-SRNIQK------
PfNEK2(PF3D7_0525900)            --AF------------VEDC--------------V---LYV------AMDYCI----------------------------NGD--L-GKVIKK------
PfNEK3(PF3D7_1201600)            --YI----------K-ENDT------------------LSF------ILEFCN----------------------------QGD--L-HSDIL-------
PfNEK4(PF3D7_0719200)            --SY-----------IEGDT------------------LRI------VMKHCK----------------------------GGD--L-YHYIQN------
PfPK1(PF3D7_0821100)             --------------------------------------------------YIN----------------------------S-L--M-YKY---------
PfPK2(PF3D7_1238900)             --AY-----------EQEGF------------------VYL------VLEYLK----------------------------GGE--L-FEYLN-------
PfPK4(PF3D7_0628200)             --IV----------------------------------LLL------QMELCK----------------------------GYT--L-RKWLDRSTRS--
PfPK5(PF3D7_1356900)             --VI-----------HTKKR------------------LVL------VFEHLD----------------------------Q-D--L-KKLLD-------
PfPK6(PF3D7_1337100)             --VF-----------YGKDIEDK-LKGENL--E--NSCLYL------AFEYCD----------------------------I-D--L-FNLIK-------
PfPK7(PF3D7_0213400)             --II----------T-NYDE------------------VYI------IYEYME----------------------------NDS--I-LKFD--------
PfPK9(PF3D7_1315100)             --VN-----------HFQNY------------------VCL------IMEYAI----------------------------NGD--L-KNYIK-------
PfPKA(PF3D7_0934800)             --SF-----------KDDSY------------------LYL------VLEFVI----------------------------GGE--F-FTFLR-------
PfPKB(PF3D7_1246900)             --AF-----------QTKQK------------------LYF------ILEYCP----------------------------GGE--L-FFHLS-------
PfPKG(PF3D7_1436600)             --TF-----------KDSKY------------------FYF------LTELVT----------------------------GGE--L-YDAIR-------
PfPutativeCaMK2(PF3D7_1423600)   --FF-----------EDKNK------------------FYI------VLEKCE----------------------------GGE--L-FYKVV-------
PfPutativeK(PF3D7_0107600)       --------------------------------------LYI------RMEYCKS------------------------------T-L-ENYI--------
PfPutativeK(PF3D7_0321400)       --IF----------E-DQDF------------------AYV------VSENCS----------------------------GGF--L-FDVL--------
PfPutativeK(PF3D7_0926100)       --GI------------IHSY-----FD-----------IWL------VTKLVN----------------------------GLD--L-HTIK--------
PfPutativeK(PF3D7_1145200)       --VIG-----------GKEG------------------WIL------IQDFAN----------------------------DGT--L-WKEN--------
PfPutativeK(PF3D7_1148000)       FFVFG-----------SATN------------------LLV------GMELCDID-------------------------------L-DKYIK-------
PfPutativeK(PF3D7_1316000)       --VF-----------YTTTK-----KGYRI--------QNI------VFKYMT----------------------------Y-S--L-GRYIRMKKQEKR
PfPutativeK(PF3D7_1331000)       --VH------------TESN---------------------------IAGFLTPFLL------------------------NGN--I-KKHIS-------
PfPutativeK(PF3D7_1441300)       --IL-----------ETKDK------------------IIQ------IMEYCD----------------------------AGD--L-ISYVR-------
PfRIO1(PF3D7_1230900)            ----------------EKEFRNL--RRILICGLRCPYPLVLRS-NVIVMSMIG----------------------------YIDN-A-CPKM--------
PfRIO2(P3D7_0420100)             --IN----------R-----------------------HMI------IMSYIK----------------------------GYP--L-------------
PfSRPK1(PF3D7_0302100)           --------------------------------------------------------------------------------------V-------------
PfSRPK2(PF3D7_1443000)           --LL----------QQNYDR---------------------------KIDIWS----------------------------LGCI-L-FEFLT-------
PfTKL1(PF3D7_0211700)            --------------A-INNP------------------RAI------VLEYIQ----------------------------YGT--L-FDIL--------
PfTKL2(PF3D7_1121300)            --YA----------T-NKNN------------------FYL------IYEYVN----------------------------LGD--L-RTLLFNHYY---
PfTKL3(PF3D7_1349300)            --VC----------SLREGE------------------ESL------ILQHCP----------------------------GGS--L-EKYIY-------
PfTKL4(PF3D7_0623800)            --IC----------I-KSNK------------------LML------LLEYYA----------------------------KGN--L-FNFL--------
PfVPS15(PF3D7_0823000)           -DIF----------N-----------------------LGI-----LILEILLR---------------------------DKD--VSYFFLNENCA---
PVX_091015                       --PL----------K-ED----------------------------------------------------------------------------------
Py02791                          ----------------------------------------------------------------------------------------------------
TGGT1_209050 (TgTKL5)            --MC----------L-DPGC------------------VGI------VMEYLP----------------------------GGS--L-FDLL--------
TGGT1_225770 (TgTKL8)            --FA----------R-HGKD------------------RFL------VYELLS----------------------------GGD--V-GMRL--------
TGGT1_234970 (TgTKL2)            --CF----------EMGNDV------------------LAS------ILEFCE----------------------------GGD--V-DHFLK-------
TGGT1_236240 (TgTKL6)            --VAA---------TSTTGS----ARE-----------VLL------LLELCE----------------------------GGH--L-LDLLD-------
TGGT1_237210 (TgTKL4)            --------------AGTRNP-----------------PLFM------LTELCA----------------------------GGS--L-FDLLHKGSNKRV
TGGT1_239130 (TgTKL7)            --VV------------SAPL------------------YGL------VTEYVP----------------------------AGS--L-FDLL--------
TGGT1_253860 (TgTKL3)            --RC----------HLRPGE------------------EAL------IIEYCK----------------------------KGS--L-DALVFP------
TGGT1_290225                     --------------RGTGED-------------------------------------------------------------SSD--L-------------
TGGT1_301270 (TgTKL1)            --VFEGF--QMLEDRSKKKVKN--------------TSLCF------LMDIADQS-------------------------------L-ESLL--------

                                          610       620       630       640       650       660       670       680       690       700
                                 ....|....|....|....|....|....|....|....|....|....|....|....|....|....|....|....|....|....|....|....|
At_CDPK1                         ---------------------A------R---------------GH------------------------------------------------------
At_CTR1                          ---------------------K------SGAR------------EQ------------------------------------------------------
FIKK1(PF3D7_0102600)             ---------------------S------E---------------KE--------------------RMGTE-----------------------------
FIKK10.1(PF3D7_1016400)          ---------------------K------Q---------------KK--------------------KNIFA-----------------------------
FIKK10.2(PF3D7_1039000)          ---------------------N------K---------------NE--------------------NKETV-----------------------------
FIKK11(PF3D7_1149300)            ---------------------K------R---------------QK--------------------ENNNI-----------------------------
FIKK12(PF3D7_1200800)            ---------------------R------F---------------TK--------------------EKSDV-----------------------------
FIKK3(PF3D7_0301200)             ---------------------R------E---------------TE--------------------GFNTF-----------------------------
FIKK4.1(PF3D7_0424500)           ---------------------N------K---------------RK--------------------EGCFG-----------------------------
FIKK5(PF3D7_0500900)             ---------------------K------S---------------RR--------------------KPILK-----------------------------
FIKK7.1(PF3D7_0726200)           ---------------------K------R---------------RK--------------------NIFLK-----------------------------
FIKK8(PF3D7_0805700)             ---------------------R------R---------------QK--------------------KYNMK-----------------------------
FIKK9.1(PF3D7_0902000)           ---------------------K------M---------------RK--------------------VYPKV-----------------------------
FIKK9.2(PF3D7_0902100)           ---------------------S------E---------------KE--------------------NLV-------------------------------
FIKK9.4(PF3D7_0902300)           ---------------------K------K---------------GG--------------------IYRDI-----------------------------
FIKK9.5(PF3D7_0902400)           ---------------------N------I---------------RN--------------------KKTYK-----------------------------
FIKK9.6(PF3D7_0902500)           ---------------------T------V---------------NN--------------------KKIFK-----------------------------
FIKK9.7(PF3D7_0902600)           ---------------------R------M---------------CI--------------------KGYST-----------------------------
Hs_ABL1                          ---------------------R------E---------------CN--------------------RQEVN-----------------------------
Hs_BTK                           ---------------------R------E----------------M--------------------RHRFQ-----------------------------
Hs_CAMK1                         ---------------------E------K---------------GF------------------------------------------------------
Hs_CDK2                          --------------------AS------AL--------------TG------------------------------------------------------
Hs_CK1                           --------------------------------------------RR------------------------------------------------------
Hs_CLK1                          ---------------------K------E---------------NGFLP---------------------------------------------------
Hs_ERK1                          ---------------------S------Q---------------Q-------------------------------------------------------
Hs_FGFR1                         ---------------------Q------A---------------RR--------------------PPGLEYCYNPSHNPE------------EQ-----
Hs_GCN2_2                        I-----------IFDNEDENSK------SQNQDEDCNE------KN-----------------GCHESEPS-----------------------------
Hs_IGFR1                         ---------------------R------S----------------L--------------------RPEMEN--NPVLAP--------------------
Hs_IRAK1                         ---------------------H------C---------------QT--------------------QACPP-----------------------------
Hs_JAK2_1                        ---------------------K------N---------------KN----C-------------------------------------------------
Hs_LCK                           ---------------------K------T---------------PS--------------------GIKLT-----------------------------
Hs_LYN                           ---------------------K------S---------------DE--------------------GGKVL-----------------------------
Hs_NEK1                          --------------------QKG----VL-----------------------------------------------------------------------
Hs_PAK1                          ---------------------E----------------------TC------------------------------------------------------
PfPK8(PF3D7_0203100)             ----------------------------NY--------------GR------------------------------------------------------
Hs_PKAca                         ---------------------R------I---------------GR------------------------------------------------------
Hs_RAF1                          ---------------------Q------E---------------TK------------------------------------------------------
Hs_RIOK1                         ---------------------K------N---------------VQ------------------------------------------------------
Hs_RIOK2                         --------------------------------------------CQ------------------------------------------------------
Hs_RIOK3                         ---------------------K------E---------------VK------------------------------------------------------
Hs_VGFR1                         ---------------------K------S---------------KRDLFFLNKDAALHMEPKKEKMEPGLEQGKKPRLDSVTSSESFASSGFQEDKS---
PBANKA_0940100                   ---------------------K------T---------------HSYF-----------------KSKKCDSR---------------------------
PbRIO1(PBANKA_1445600)           ---------------------K------D---------------LN------------------------------------------------------
```

```
PbRIO2(PBANKA_0521400)               --------------------------------------------SH------------------------------------------------------
PbTKL1(PBANKA_0308500)               ---------------------H------K---------------KK-----------------------------------TK-----------------
PbTKL2(PBANKA_0927000)               ------------FYNSQN---K------ENIDFNNLSNMKLNNYNNIP------------------KNK----YITSLNNYTN-----------------
PbTKL4(PBANKA_1122700)               ---------------------K------N---------------KN--------------------K---------------------------------
PF3D7_1106800                        ---------------------K------T---------------HIHF-----------------NSQQ--------INDQ-------------------
PfABCk1(PF3D7_0810200)               --------------------------------------------SD------------------------------------------------------
PfABCk2(PF3D7_1414500)               --------------------------------------------SD------------------------------------------------------
PfARK1(PF3D7_0605300)                ---------------------E------N---------------GP------------------------------------------------------
PfARK2(PF3D7_0309200)                ---------------------K------K---------------QP------------------------------------------------------
PfARK3(PF3D7_1356800)                --------------------FK------K---------------RR------------------------------------------------------
PfCDPK1(PF3D7_0217500)               ---------------------N------R---------------HK------------------------------------------------------
PfCDPK2(PF3D7_0610600)               ---------------------E------N---------------GS------------------------------------------------------
PfCDPK3(PF3D7_0310100)               ---------------------K------K---------------GC------------------------------------------------------
PfCDPK4(PF3D7_0717500)               ---------------------S------R---------------KR------------------------------------------------------
PfCDPK5(PF3D7_1337800)               ---------------------K------Y---------------GS------------------------------------------------------
PfCDPK6(PF3D7_1122800)               ---------------------N------S---------------ES------------------------------------------------------
PfCDPK7(PF3D7_1123100)               ---------------------A------E---------------TR------------------------------------------------------
PfCK1.1(PF3D7_1136500.2)             --------------------------------------------RK------------------------------------------------------
PfCK2alpha(PF3D7_1108400)            --------------------------------------------PK------------------------------------------------------
PfCLK1(PF3D7_1445400)                ---------------------R------NN--------------YN------G-----------------------------------------------
PfCLK3(PF3D7_1114700)                ---------------------K------KY--------------GN------GHG---------------------------------------------
PfCRK1(PF3D7_0417800)                --------------------NK------S---------------PS------------------------------------------------------
PfCRK3(PF3D7_0415300)                E--------------KERY-KY------A---------------NL------------------------------------------------------
PfCRK4(PF3D7_0317200)                -------------------LKK-----IEN--------------RSFY------------------PEMPS-----------------------------
PfCRK5(PF3D7_0615500)                ---------------------K----------------------TKIS------------------DDQAG-----------------------------
PfGSK3(PF3D7_0312400)                ------------------YSRN------N---------------QA------------------------------------------------------
PfIK1(PF3D7_1444500)                 ---------------------D------C-------------------------------------GFIC------------------------------
PfKIN(PF3D7_1454300)                 ---------------------N------N---------------YN------------------------------------------------------
PfMAPK1(PF3D7_1431500)               ---------------------A------D---------------L-------------------------------------------------------
PfMAPK2(PF3D7_1113900)               ---------------------T------P---------------IF------------------------------------------------------
PfMRK(PF3D7_1014400)                 --------------------RK----------------------IF------------------------------------------------------
PfNEK1(PF3D7_1228300)                --------------------CYK----MF---------------GK------------------------------------------------------
PfNEK2(PF3D7_0525900)                --------------------HKE----LE---------------TP------------------------------------------------------
PfNEK3(PF3D7_1201600)                --------------------------------------------RK------------------KLNNEI------------------------------
PfNEK4(PF3D7_0719200)                --------------------KKK----QN---------------TP------------------------------------------------------
PfPK1(PF3D7_0821100)                 ----------------------------------------------------------------------------------------------------
PfPK2(PF3D7_1238900)                 ---------------------N------N---------------GP------------------------------------------------------
PfPK4(PF3D7_0628200)                 --------------------DKPLHFTYSD--------------KK------------------------------------------------------
PfPK5(PF3D7_1356900)                 --------------------VC------E---------------GG------------------------------------------------------
PfPK6(PF3D7_1337100)                 --------------------KH------N-----------------------------------------------------------------------
PfPK7(PF3D7_0213400)                 ---------------------E------YF--------FV---LDK------------------NYT-CF------------------------------
PfPK9(PF3D7_1315100)                 ---------------------NK----FN---------------GF------------------------------------------------------
PfPKA(PF3D7_0934800)                 ---------------------R------N---------------KR------------------------------------------------------
PfPKB(PF3D7_1246900)                 ---------------------K------L---------------RE------------------------------------------------------
PfPKG(PF3D7_1436600)                 ---------------------K------L---------------GL------------------------------------------------------
PfPutativeCaMK2(PF3D7_1423600)       ---------------------K------N---------------KC------------------------------------------------------
PfPutativeK(PF3D7_0107600)           ---------------------N------TR--------------EN------------------------------------------------------
PfPutativeK(PF3D7_0321400)           --------------------------------------------KN--------------------NDTI------------------------------
PfPutativeK(PF3D7_0926100)           ---------------------N------N---------------MN--------------------KDEKV-----------------------------
PfPutativeK(PF3D7_1145200)           --------------------------------------------LS------------------------------------------------------
PfPutativeK(PF3D7_1148000)           --------------------YH----------------------GP------------------------------------------------------
PfPutativeK(PF3D7_1316000)           --------------------EN------KY--------------DR------------------------------------------------------
PfPutativeK(PF3D7_1331000)           --------------------------------------------KN--V-----------------QPYIKY----------------------------
PfPutativeK(PF3D7_1441300)           ---------------------N------K---------------LY------------------------------------------------------
PfRIO1(PF3D7_1230900)                ---------------------K------D---------------LN------------------------------------------------------
PfRIO2(P3D7_0420100)                 --------------------------------------------SH------------------------------------------------------
PfSRPK1(PF3D7_0302100)               ----------------------------------------------------------------------------------------------------
PfSRPK2(PF3D7_1443000)               --------------------------------------------KKIL----------------------------------------------------
PfTKL1(PF3D7_0211700)                ---------------------H------K---------------YK-----------------------------------IN-----------------
PfTKL2(PF3D7_1121300)                ------------YYNSKN---K------ENPE---LSYYNISCYENYL------------------RKKKSSSSCSSTNYYTST-------FYKQNIF--
PfTKL3(PF3D7_1349300)                ---------------------K------DE--------------KK--------------------KNSP-YAKS-------------------------
PfTKL4(PF3D7_0623800)                ---------------------K------N---------------KN--------------------K---------------------------------
PfVPS15(PF3D7_0823000)               --------------------YK------ND--------------DNFY---------------SGRRKQSG-----------------------------
PVX_091015                           ----------------------------------------------------------------------------------------------------
Py02791                              ----------------------------------------------------------------------------------------------------
TGGT1_209050 (TgTKL5)                ---------------------Y------E---------------NKVL----------------------------------------------------
TGGT1_225770 (TgTKL8)                ---------------------Q------K---------------GP-L----------------------------------------------------
TGGT1_234970 (TgTKL2)                --------------------LS----------------------GP------------------------------------------------------
TGGT1_236240 (TgTKL6)                ---------------------R------HN--------------GS------------------------------------------------------
TGGT1_237210 (TgTKL4)                TGEQASSASRAQASESAQVETR------QDPSDDPYRAAVASMLEKRL----GAET-----RLDGSNAESVSEESGGFWSFLGKFSF----FPKKEPRVP
TGGT1_239130 (TgTKL7)                ---------------------H------V---------------RRIP----------------------------------------------------
TGGT1_253860 (TgTKL3)                ---------------------R------EN--------------ER--------------------RGGPKLWKA-------------------------
TGGT1_290225                         ----------------------------------------------------------------------------------------------------
TGGT1_301270 (TgTKL1)                ---------------------E------E---------------HE--------------------KANTC-----------------------------

                                              710       720       730       740       750       760       770       780       790       800
                                     ....|....|....|....|....|....|....|....|....|....|....|....|....|....|....|....|....|....|....|....|
At_CDPK1                             --------------------------Y-TERAAAAV------------------------------ARTIAE-----VVMMCHS---NG-------VMHR
At_CTR1                              --------------------------L-DERRRLSM------------------------------AYDVAK-----GMNYLHN---RN--P---PIVHR
FIKK1(PF3D7_0102600)                 --------------------------E-WFEDVKKI------------------------------LFKSLK-----LLIRLHD---VG-------ITHL
FIKK10.1(PF3D7_1016400)              --------------------------LHSYMKRKKI------------------------------LFECLN-----VLRKLHD---AG-------LCHL
FIKK10.2(PF3D7_1039000)              --------------------------V-SVEEKKKI------------------------------MKECLK-----LLIKLHN---AG-------LAHL
FIKK11(PF3D7_1149300)                --------------------------L-SDREKKKI------------------------------LYECLK-----LLIKLHN---VG-------IAHL
FIKK12(PF3D7_1200800)                --------------------------RNSDEFKKEL------------------------------LYKCLR-----LLVRLHS---AG-------LSHL
FIKK3(PF3D7_0301200)                 --------------------------L-NKNKKKKI------------------------------LYESLH-----LLVKLHD---AG-------FSHL
FIKK4.1(PF3D7_0424500)               --------------------------N-NLEEKKKI------------------------------LHESLK-----LITTLHE---TG-------LSHL
FIKK5(PF3D7_0500900)                 --------------------------N-RYAKKKKI------------------------------IYDSLN-----LLIRLHD---AG-------LTHL
FIKK7.1(PF3D7_0726200)               --------------------------I-RRKDKINI------------------------------LHACLK-----LLARLHD---AG-------LCHL
FIKK8(PF3D7_0805700)                 --------------------------I-SNNEKSFI------------------------------LYQCLK-----LLIRLHD---AG-------LSHL
FIKK9.1(PF3D7_0902000)               --------------------------F-NKKSKKKI------------------------------LFECLK-----LIDKLHD---TG-------LSHL
FIKK9.2(PF3D7_0902100)               ----------------------------ITKERKKI------------------------------LFECLK-----LINKLHK---AG-------LTHL
FIKK9.4(PF3D7_0902300)               ----------------------------SENERKKI------------------------------IHEWIK-----LVSRLHD---TG-------LSHL
FIKK9.5(PF3D7_0902400)               --------------------------F-KKNDKKKI------------------------------MLECLK-----LINKLHQ---VG-------ICHL
FIKK9.6(PF3D7_0902500)               --------------------------S-RKKNKKKL------------------------------LFESLK-----LINKLHQ---AG-------ICHL
FIKK9.7(PF3D7_0902600)               --------------------------L-GRKAKKKI------------------------------MLSCLK-----LINRLHK---IG-------LCHL
Hs_ABL1                              -----------------------------AVVLLYM------------------------------ATQISS-----AMEYLEK---KN-------FIHR
```

```
Hs_BTK                          -----------------------------TQQLLEM------------------------------CKDVCE-----AMEYLES---KQ-------FLHR
Hs_CAMK1                        --------------------------Y-TERDASRL------------------------------IFQVLD-----AVKYLHD---LG-------IVHR
Hs_CDK2                         --------------------------I-PLPLIKSY------------------------------LFQLLQ-----GLAFCHS---HR-------VLHR
Hs_CK1                          --------------------------F-TMKTVLML------------------------------ADQMIS-----RIEYVHT---KN-------FIHR
Hs_CLK1                         --------------------------F-RLDHIRKM------------------------------AYQICK-----SVNFLHS---NK-------LTHT
Hs_ERK1                         --------------------------L-SNDHICYF------------------------------LYQILR-----GLKYIHS---AN-------VLHR
Hs_FGFR1                        --------------------------L-SSKDLVSC------------------------------AYQVAR-----GMEYLAS---KK-------CIHR
Hs_GCN2_2                       --------------------------V-TTEAVHYLYIQMEYCEKSTLRDTIDQGLYRDTVRLWRLFREILD-----GLAYIHE---KG-------MIHR
Hs_IGFR1                        --------------------------P-SLSKMIQM------------------------------AGEIAD-----GMAYLNA---NK-------FVHR
Hs_IRAK1                        --------------------------L-SWPQRLDI------------------------------LLGTAR-----AIQFLHQ---DS--P---SLIHG
Hs_JAK2_1                       --------------------------I-NILWKLEV------------------------------AKQLAW-----AMHFLEE---NT-------LIHG
Hs_LCK                          -----------------------------INKLLDM------------------------------AAQIAE-----GMAFIEE---RN-------YIHR
Hs_LYN                          -----------------------------LPKLIDF------------------------------SAQIAE-----GMAYIER---KN-------YIHR
Hs_NEK1                         --------------------------F-QEDQILDW------------------------------FVQICL-----ALKHVHD---RK-------ILHR
Hs_PAK1                         --------------------------M-DEGQIAAV------------------------------CRECLQ-----ALEFLHS---NQ-------VIHR
PfPK8(PF3D7_0203100)            --------------------------I-SEDLLVYI------------------------------LDDVLN-----GLNYLHN---ECSSP----LIHR
Hs_PKAca                        --------------------------F-SEPHARFY------------------------------AAQIVL-----TFEYLHS---LD-------LIYR
Hs_RAF1                         --------------------------F-QMFQLIDI------------------------------ARQTAQ-----GMDYLHA---KN-------IIHR
Hs_RIOK1                        --------------------------L-SESKAREL------------------------------YLQVIQ-----YMRRMYQD--AR-------LVHA
Hs_RIOK2                        --------------------------IHHVEDPASV------------------------------YDEAME-----LIVKLAN---HG-------LIHG
Hs_RIOK3                        --------------------------L-NSEEMKEA------------------------------YYQTLH-----LMRQLYHE--CT-------LVHA
Hs_VGFR1                        -----LSDVEEEEDSDGFYKEP----I-TMEDLISY------------------------------SFQVAR-----GMEFLSS---RK-------CIHR
PBANKA_0940100                  -------------------KNK----L-SDHQIMKI------------------------------IMDITL-----ACSYLEK---QK-------VRWI
PbRIO1(PBANKA_1445600)          --------------------------V-SILKWKEL------------------------------YIECIC-----ILRLLYSI--CK-------LVHA
PbRIO2(PBANKA_0521400)          --------------------------V-KISNPFKV------------------------------IDFLLN-----TIIKFAK---SD-------IIHG
PbTKL1(PBANKA_0308500)          --------------------------I-KILDIIKM------------------------------CKDITS-----FMSFLHN---KG-------ILHC
PbTKL2(PBANKA_0927000)          -----LNYSKTS----NLSENQTPLFL-SFSTRINI------------------------------LVQIIN-----VLCYLHT---SS--PI---VYHR
PbTKL4(PBANKA_1122700)          --------------------------I-HKKQRLEW------------------------------AIQMCS-----IVHELHS---HN--P---PIING
PF3D7_1106800                   -------------------HNR----L-SVQKIMKI------------------------------ITDVTL-----ACTYLEK---EK-------MSPI
PfABCk1(PF3D7_0810200)          ----------------------------------------------------------------------IK-----NLLFINQY--TN-------LILK
PfABCk2(PF3D7_1414500)          ----------------------------------------------------------------------IS-----TLKKVSWA--FG-------LIDK
PfARK1(PF3D7_0605300)           --------------------------F-CEKKVAEM------------------------------LFEIIW-----AIRTCHD---KR-------IAHL
PfARK2(PF3D7_0309200)           --------------------------L-NEEEVALY------------------------------VFQIAD-----ALSYLHN---FN-------IIHR
PfARK3(PF3D7_1356800)           --------------------------I-PDELAYKY------------------------------FCHVVN-----GLYYLNQ---MG-------IFHR
PfCDPK1(PF3D7_0217500)          --------------------------F-DECDAANI------------------------------MKQILS-----GICYLHK---HN-------IVHR
PfCDPK2(PF3D7_0610600)          --------------------------F-TEKNAATI------------------------------MKQIFS-----AIFYLHS---LN-------IVHR
PfCDPK3(PF3D7_0310100)          --------------------------F-VETFASFI------------------------------MKQIFS-----VLNYLHI---RN-------ICHR
PfCDPK4(PF3D7_0717500)          --------------------------F-YEIDAARI------------------------------IKQILS-----GITYMHK---NN-------VVHR
PfCDPK5(PF3D7_1337800)          --------------------------F-SEYEAYKI------------------------------MKQIFS-----ALYYCHS---KN-------IMHR
PfCDPK6(PF3D7_1122800)          --------------------------F-NETYIKNI------------------------------MFQILC-----AIAYMHS---NN-------IAHK
PfCDPK7(PF3D7_1123100)          --------------------------L-SEIHANKI------------------------------ITQLIK-----TVAYLHR---CG-------IIHR
PfCK1.1(PF3D7_1136500.2)        --------------------------F-SLKTVLMT------------------------------ADQMLN-----RIEYVHS---KN-------FIHR
PfCK2alpha(PF3D7_1108400)       --------------------------F-TDKDIRYY------------------------------IYQILK-----ALDYCHS---QG-------IMHR
PfCLK1(PF3D7_1445400)           --------------------------F-HIEDIKLY------------------------------CIEILK-----ALNYLRK---MS-------LTHT
PfCLK3(PF3D7_1114700)           --------------------------L-NATAVHCY------------------------------TKQLFI-----ALRHMRK---CR-------IMHA
PfCRK1(PF3D7_0417800)           --------------------------F-TISELKCL------------------------------LKQLLS-----GINYLHR---NW-------VMHR
PfCRK3(PF3D7_0415300)           --------------------------F-SIGEIKNI------------------------------FIQLLK-----ALDYCHK---NN-------IIHR
PfCRK4(PF3D7_0317200)           --------------------------L-TEIQTKVV------------------------------IYQMLQ-----GINHFHK---KF-------IIHR
PfCRK5(PF3D7_0615500)           --------------------------L-NLKEAKWL------------------------------SFQLLN-----GLAYLHN---NK-------MCHR
PfGSK3(PF3D7_0312400)           --------------------------L-PMFLVKLY------------------------------SYQLCR-----ALSYIHS---KF-------ICHR
PfIK1(PF3D7_1444500)            --------------------------R-NEKLIWEL------------------------------IKQILK-----GISYIHD---MK-------IMHR
PfKIN(PF3D7_1454300)            --------------------------I-NENNARRI------------------------------LYQLIN-----AIEYLHE---IK-------IVHR
PfMAPK1(PF3D7_1431500)          --------------------------L-EEIHKKYI------------------------------IYQLLR-----ALKYIHS---GG-------LLHR
PfMAPK2(PF3D7_1113900)          --------------------------L-TEQHVKTI------------------------------LYNLLL-----GEKFIHE---SG-------IIHR
PfMRK(PF3D7_1014400)            --------------------------L-TDSQKKCI------------------------------LLQILN-----GLNVLHK---YY-------FMHR
PfNEK1(PF3D7_1228300)           --------------------------I-EEHAIIDI------------------------------TRQLLH-----ALAYCHN---LKDGPNGERVLHR
PfNEK2(PF3D7_0525900)           --------------------------I-PEKKIKRW------------------------------LLQIIM-----AIKFIHD---KK-------LIHR
PfNEK3(PF3D7_1201600)           --------------------------Y-TESEIFNI------------------------------LHQILN-----GLNIIHQ---NG-------IIHG
PfNEK4(PF3D7_0719200)           --------------------------I-KEKRILIW------------------------------LTQILT-----ALKFLHS---NH-------ILHR
PfPK1(PF3D7_0821100)            ----------------------------------------------------------------------------------------------------
PfPK2(PF3D7_1238900)            --------------------------Y-TEQVAKKA------------------------------MKRVLI-----ALEALHS---NG-------VVHR
PfPK4(PF3D7_0628200)            --------------------------M-NHPLEFDL------------------------------FKQLIK-----GLKDIHA---TC-------FIHR
PfPK5(PF3D7_1356900)            --------------------------L-ESVTAKSF------------------------------LLQLLN-----GIAYCHD---RR-------VLHR
PfPK6(PF3D7_1337100)            --------------------------L-NIKEIKYI------------------------------IFELLL-----ALSYFHS---NN-------YIHR
PfPK7(PF3D7_0213400)            --------------------------I-PIQVIKCI------------------------------IKSVLN-----SFSYIHN---EK------NICHR
PfPK9(PF3D7_1315100)            --------------------------L-SEKEAHDL------------------------------FLQIVK-----GVYYCHS---KH-------IVHR
PfPKA(PF3D7_0934800)            --------------------------F-PNDVGCFY------------------------------AAQIVL-----IFEYLQS---LN-------IVYR
PfPKB(PF3D7_1246900)            --------------------------F-SEETAKFY------------------------------SSEIIL-----ALEYLHD---LN-------IIYR
PfPKG(PF3D7_1436600)            --------------------------L-SKSQAQFY------------------------------LGSIIL-----AIEYLHE---RN-------IVYR
PfPutativeCaMK2(PF3D7_1423600)  --------------------------L-MESESALI------------------------------VRQICC-----ALQYLHS---NN-------IIHR
PfPutativeK(PF3D7_0107600)      --------------------------M-NINRNYEI------------------------------IQMIIL-----GLYSIHN---NN-------IMHR
PfPutativeK(PF3D7_0321400)      --------------------------I-SEQSLSTW------------------------------LYQIIT-----ALLFMEE---HD-------IYHG
PfPutativeK(PF3D7_0926100)      --------------------------L-GIDISLKM------------------------------CRQLAE-----VINFLHT---PIKNKK-NVIIHR
PfPutativeK(PF3D7_1145200)      --------------------------S-NMNEAFLY------------------------------FIQLLQ-----GMWYIQE---MN-------IVHR
PfPutativeK(PF3D7_1148000)      --------------------------I-NELLALCW------------------------------IKQILL-----GLLYMKNL-PTG-------KVHH
PfPutativeK(PF3D7_1316000)      --------------------------I-NPADLKNI------------------------------IYQICI-----GIKDLHK---ND-------FAHR
PfPutativeK(PF3D7_1331000)      -------------------------HF-NNQLIYKN------------------------------LCNIIK-----LMCYLQD---CN-------ICHG
PfPutativeK(PF3D7_1441300)      --------------------------L-DEVSAQYF------------------------------FRKIVE-----GLKYMHK---NN-------IAHR
PfRIO1(PF3D7_1230900)           --------------------------F-DILKWKEL------------------------------YIECIC-----ILRFLFFN--CK-------LVHA
PfRIO2(P3D7_0420100)            --------------------------V-KLNNPYKI------------------------------IDTLFN-----ILVKFAK---AD-------IIHG
PfSRPK1(PF3D7_0302100)          ------------------------------------------------------------------------------------------------YYHE
PfSRPK2(PF3D7_1443000)          --------------------------F-DYQNIYRF------------------------------IYSIVSYIGPFPFYMINN---CR-------IPHI
PfTKL1(PF3D7_0211700)           --------------------------M-KLQDIIKI------------------------------SKDITA-----FMSFLHN---KG-------IMHC
PfTKL2(PF3D7_1121300)           ---PYFNNSKYSPFQQNSFDNNTPLFL-SFNIRINI------------------------------LVQIIN-----VLCYLHT---SS--PI---VYHR
PfTKL3(PF3D7_1349300)           --------------------------L-TRPKLVKI------------------------------FQQVAE-----GMYNIHT---NQ-------CFHR
PfTKL4(PF3D7_0623800)           --------------------------I-HKKQRLEW------------------------------AIQISC-----IVHELHA---HN--P---PIING
PfVPS15(PF3D7_0823000)          --------------------------LTFLKKMESIDED--------------------------DKNDVENYHHD-SNDYHHN---EN--YY-NMNQHY
PVX_091015                      --------------------RR----L-SVQRILKI------------------------------TTDVTL-----ACSYLEK---HL-------SHPM
Py02791                         ---------------------------------------------------------------------ITL-----ACSYLEK---QK-------IRWL
TGGT1_209050 (TgTKL5)           --------------------------V-AAERRLVL------------------------------SRQLTQ-----AVHYMHH---EK------RMVHR
TGGT1_225770 (TgTKL8)           --------------------------L-SWQDRLSL------------------------------ALDSAS-----ALSHLQH---HS--P---QVYHR
TGGT1_234970 (TgTKL2)           --------------------------L-RESLAAEW------------------------------TRQILE-----ALLYLKRQ-PVG-------VIHH
TGGT1_236240 (TgTKL6)           --------------------------L-KEEWILHI------------------------------VKEITA-----GLAHLHS---QP-TP----VAHR
TGGT1_237210 (TgTKL4)           TPASFFLPPKTPSNEDILFSRGPAVSL-DWKLRAQI------------------------------ALDLAR-----GCQYLHS---VN-------VVHR
TGGT1_239130 (TgTKL7)           --------------------------L-SFSQVARF------------------------------ARDICH-----GMRYLHE---QG-------VLHC
TGGT1_253860 (TgTKL3)           --------------------------L-SRPKLVQI------------------------------FIDVAK-----GMAHVHS---RN-------ILHR
```

```
TGGT1_290225                    --------------------------W---------------------------------------------------------------------LKHA
TGGT1_301270 (TgTKL1)           --------------------------L-DVEFILTI------------------------------LLDTAR-----GMNYLHSPSATK--PH---LLHR

                                       810       820       830       840       850       860       870       880       890       900
                                ....|....|....|....|....|....|....|....|....|....|....|....|....|....|....|....|....|....|....|....|
At_CDPK1                        -DLKPENFLF------------------A-----------------N----------KKENSP--------------------L----KA-IDF------
At_CTR1                         -DLKSPNLLV------------------D-----------------K----------KYT-----------------------V----KV-CDF------
FIKK1(PF3D7_0102600)            -DLTPENVLI------------------T-----------------K----------NFD-----------------------I----RF-CDF------
FIKK10.1(PF3D7_1016400)         -DISPQNILM------------------S-----------------Y----------NFE-----------------------I----RL-CDL------
FIKK10.2(PF3D7_1039000)         -DISPENILI------------------S-----------------N----------NSE-----------------------F----RL-CDL------
FIKK11(PF3D7_1149300)           -DISLENILM------------------T-----------------E----------NYE-----------------------F----LL-CDF------
FIKK12(PF3D7_1200800)           -DLTAENVLI------------------T-----------------D----------DYD-----------------------I----RL-CDF------
FIKK3(PF3D7_0301200)            -DLSPENILI------------------S-----------------D----------KYQ-----------------------M----LF-CDF------
FIKK4.1(PF3D7_0424500)          -DISPENILI------------------G-----------------N----------NCE-----------------------L----KL-CDF------
FIKK5(PF3D7_0500900)            -DFTPENILI------------------S-----------------E----------NNE-----------------------L----RL-CDL------
FIKK7.1(PF3D7_0726200)          -DLTPDNILI------------------S-----------------K----------SMD-----------------------L----RL-CDF------
FIKK8(PF3D7_0805700)            -DLTPENILI------------------S-----------------D----------NYE-----------------------L----RF-CDL------
FIKK9.1(PF3D7_0902000)          -DFTPENILI------------------S-----------------D----------NFE-----------------------F----RI-CDF------
FIKK9.2(PF3D7_0902100)          -DISPENILI------------------G-----------------E----------NYE-----------------------M----RL-CDF------
FIKK9.4(PF3D7_0902300)          -DISPENTLI------------------G-----------------E----------NHK-----------------------M----RL-CDF------
FIKK9.5(PF3D7_0902400)          -DFSIDNILI------------------S-----------------K----------NGD-----------------------M----RL-CDF------
FIKK9.6(PF3D7_0902500)          -DFTLDNILI------------------S-----------------K----------NGD-----------------------M----RL-CDF------
FIKK9.7(PF3D7_0902600)          -DISLENILM------------------Q-----------------D----------NYE-----------------------M----RI-CDF------
Hs_ABL1                         -DLAARNCLV------------------G-----------------E----------NHL-----------------------V----KV-ADF------
Hs_BTK                          -DLAARNCLV------------------N-----------------D----------QGV-----------------------V----KV-SDF------
Hs_CAMK1                        -DLKPENLLY------------------Y-----------------S----------LDEDSK--------------------I----MI-SDF------
Hs_CDK2                         -DLKPQNLLI------------------N-----------------T----------EGA-----------------------I----KL-ADF------
Hs_CK1                          -DIKPDNFLM------------------GIG---------------R----------HCN----------------------KL----FL-IDF------
Hs_CLK1                         -DLKPENILFV---------------QSD---------------YTEA----YNPKIKRDERT-------------------LINPDIKV-VDF------
Hs_ERK1                         -DLKPSNLLI------------------N-----------------T----------TCD-----------------------L----KI-CDF------
Hs_FGFR1                        -DLAARNVLV------------------T-----------------E----------DNV-----------------------M----KI-ADF------
Hs_GCN2_2                       -DLKPVNIFL------------------D-----------------S----------DDH-----------------------V----KI-GDF------
Hs_IGFR1                        -DLAARNCMV------------------A-----------------E----------DFT-----------------------V----KI-GDF------
Hs_IRAK1                        -DIKSSNVLL------------------D-----------------E----------RLT-----------------------P----KL-GDF------
Hs_JAK2_1                       -NVCAKNILLIREE--------------DR----------------K----------TGN-------------PP-------FI----KL-SDP------
Hs_LCK                          -DLRAANILV------------------S-----------------D----------TLS-----------------------C----KI-ADF------
Hs_LYN                          -DLRAANVLV------------------S-----------------E----------SLM-----------------------C----KI-ADF------
Hs_NEK1                         -DIKSQNIFL------------------T-----------------K----------DGT-----------------------V----QL-GDF------
Hs_PAK1                         -DIKSDNILL------------------G-----------------M----------DGS-----------------------V----KL-TDF------
PfPK8(PF3D7_0203100)            -DIKPTNIVL------------------S-----------------K----------DGI-----------------------A----KI-IDF------
Hs_PKAca                        -DLKPENLLI------------------D-----------------Q----------QGY-----------------------I----QV-TDF------
Hs_RAF1                         -DMKSNNIFL------------------H-----------------E----------GLT-----------------------V----KI-GDF------
Hs_RIOK1                        -DLSEFNMLY------------------------------------H----------GGG-----------------------V----YI-IDV------
Hs_RIOK2                        -DFNEFNLIL------------------D-----------------E----------SDH-----------------------I----TM-IDF------
Hs_RIOK3                        -DLSEYNMLW------------------------------------H----------AGK-----------------------V----WL-IDV------
Hs_VGFR1                        -DLAARNILL------------------S-----------------E----------NNV-----------------------V----KI-CDF------
PBANKA_0940100                  -NLKPTNILL------------------D-----------------G----------SLN-----------------------A----KI-SDF------
PbRIO1(PBANKA_1445600)          -DFSEYNLLY----------------FYN-----------------H------------------------------------I----YI-IDV------
PbRIO2(PBANKA_0521400)          -DFNEFNILI------------------D-----------------D----------NEN-----------------------I----TI-IDF------
PbTKL1(PBANKA_0308500)          -DLKSPNILL------------------S-----------------E----------SGE-----------------------I----KI-CDF------
PbTKL2(PBANKA_0927000)          -DLKSANILI------------------D-----------------E----------KFN-----------------------A----KL-GDF------
PbTKL4(PBANKA_1122700)          -DIKTSNILI------------------N-----------------N----------NMD-----------------------L----VM-CDF------
PF3D7_1106800                   -NLKPTNILL------------------D-----------------E----------SLN-----------------------A----KI-SDF------
PfABCk1(PF3D7_0810200)          -NLYIENLCN------------------VI----------------Q----------KE------------------------L----KCECDY------
PfABCk2(PF3D7_1414500)          -NFYFTDFID------------------EW----------------Q----------DS------------------------A----SRELNY------
PfARK1(PF3D7_0605300)           -DLKPENVLV------------------N-----------------H----------EEK-----------------------C----KL-ADF------
PfARK2(PF3D7_0309200)           -DLKPDNILI------------------H-----------------Y----------SNEHLNNKI------------YKYGVI----KL-ADF------
PfARK3(PF3D7_1356800)           -DIKMENVLV------------------D-----------------H----------KDN-----------------------A----KL-SDF------
PfCDPK1(PF3D7_0217500)          -DIKPENILL------------------E-----------------------------NKHSLL------------------NI----KI-VDF------
PfCDPK2(PF3D7_0610600)          -DLKPENFLF------------------Q-----------------S----------ENKDSL--------------------L----KI-IDF------
PfCDPK3(PF3D7_0310100)          -DIKPENFLF------------------Y-----------------D----------MTPESL--------------------I----KI-IDF------
PfCDPK4(PF3D7_0717500)          -DLKPENILL------------------E-----------------T----------KNKEDMI-------------------I----KI-IDF------
PfCDPK5(PF3D7_1337800)          -DLKPENILY------------------V-----------------D----------NTEDSP--------------------I----QI-IDW------
PfCDPK6(PF3D7_1122800)          -DLKPENILF------------------K-----------------E----------KGDDT---------------------L----KI-IDF------
PfCDPK7(PF3D7_1123100)          -DIKPENILL------------------T-----------------D----------KSRDAQ--------------------I----KL-TDF------
PfCK1.1(PF3D7_1136500.2)        -DIKPDNFLI------------------GRG---------------K----------KVT----------------------LI----HI-IDF------
PfCK2alpha(PF3D7_1108400)       -DVKPHNIMI------------------D-----------------H----------ENR--Q--------------------I----RL-IDW------
PfCLK1(PF3D7_1445400)           -DLKPENILL------------------D-------------DPYFEKSLITVRRVTDGKKIQ----IYRTKST--------GI----KL-IDF------
PfCLK3(PF3D7_1114700)           -DLKPDNILI------------------N-----------------E----------KFN----------------------AL----KV-CDL------
PfCRK1(PF3D7_0417800)           -DLKPTNLLY------------------S-----------------N----------KGI-----------------------L----KI-CDF------
PfCRK3(PF3D7_0415300)           -DIKIANLLI------------------D-----------------N----------NGI-----------------------L----KL-ADF------
PfCRK4(PF3D7_0317200)           -DIKPANTLIKNIQ-----------YLSD--G----------L---N----------DPKE--------------------WIV----KI-ADF------
PfCRK5(PF3D7_0615500)           -DLKPENVML------------------Q-----------------ET--------SNHK---------------------YLL----KI-GDL------
PfGSK3(PF3D7_0312400)           -DLKPQNLLI------------------D-----------------P----------RTH--T--------------------L----KL-CDF------
PfIK1(PF3D7_1444500)            -DIKPSNIFL------------------QI----------------T----------DNI--------------L-------IA----KI-GDF------
PfKIN(PF3D7_1454300)            -DLKPENILL------------------D-----------------N----------NNN-----------------------V----KL-IDF------
PfMAPK1(PF3D7_1431500)          -DIKPSNILV------------------N-----------------S----------ECH-----------------------I----KV-ADF------
PfMAPK2(PF3D7_1113900)          -DLKPANCLL------------------N-----------------Q----------DCS-----------------------V----KI-CDF------
PfMRK(PF3D7_1014400)            -DLSPANIFI------------------N-----------------K----------KGE-----------------------V----KL-ADF------
PfNEK1(PF3D7_1228300)           -DLKPQNIFL------------------STGIRHIGKISSQANNLNS----------RPI-----------------------A----KI-GDF------
PfNEK2(PF3D7_0525900)           -DLKCNNIFL------------------D-----------------E----------KER-----------------------A----KI-GDF------
PfNEK3(PF3D7_1201600)           -DLKSTNIFI------------------------------------K----------DNK-----------------------I----KI-GDF------
PfNEK4(PF3D7_0719200)           -DMKSLNILI------------------D-----------------S----------DKR-----------------------V----RL-CDF------
PfPK1(PF3D7_0821100)            -----------------------------------------------------------------------------------I----KL-CDF------
PfPK2(PF3D7_1238900)            -DLKMENLML------------------E-----------------N----------PNDPSS--------------------L----KI-IDF------
PfPK4(PF3D7_0628200)            -DLKPENIFV------------------D--------------P--D----------TYT-----------------------L----KI-GDL------
PfPK5(PF3D7_1356900)            -DLKPQNLLI------------------N-----------------R----------EGE-----------------------L----KI-ADF------
PfPK6(PF3D7_1337100)            -DIKPENIFI------------------T-----------------S----------EGE-----------------------I----KL-GDL------
PfPK7(PF3D7_0213400)            -DVKPSNILM------------------D-----------------K----------NGR-----------------------V----KL-SDF------
PfPK9(PF3D7_1315100)            -DLKLENILL------------------D-----------------E----------KMT-----------------------C----KI-ADF------
PfPKA(PF3D7_0934800)            -DLKPENLLL------------------D-----------------K----------DGF-----------------------I----KM-TDF------
PfPKB(PF3D7_1246900)            -DLKPENVLL------------------D-----------------E----------LGH-----------------------I----RL-TDF------
PfPKG(PF3D7_1436600)            -DLKPENILL------------------D-----------------K----------QGY-----------------------V----KL-IDF------
PfPutativeCaMK2(PF3D7_1423600)  -DIKAENFLF------------------K-----------------N----------KNTKN---------------------I----KL-IDF------
PfPutativeK(PF3D7_0107600)      -DLKPSNIFI------------------S-----------------D----------NNI-----------------------V----KI-GDF------
PfPutativeK(PF3D7_0321400)      -NVNGYCIFF-K----------------D-----------------E----------DRK----------------------EI----RV-SLL------
```

```
PfPutativeK(PF3D7_0926100)     -DIKPENLII------------------D-----------------S----------DWN-----------------------I----HL-CDF------
PfPutativeK(PF3D7_1145200)     -DLKPTNILR-----------------------------------YD----------NKR-----------------------I----VI-ADF------
PfPutativeK(PF3D7_1148000)     CDLKPANLLI------------------------------------K----------DGI-----------------------I----KI-SDF------
PfPutativeK(PF3D7_1316000)     -DLKPDNILI------------------D-----------------L----------DSSNIK--------------------I----EI-CDL------
PfPutativeK(PF3D7_1331000)     -NIKPSNLFI------------------S-----------------N----------DGL----------------------HI----LL-GNFIPR--L
PfPutativeK(PF3D7_1441300)     -DLKPENIFL------------------C-----------------K----------IQISQKEKTLIRVGKLPSCIEYD---L----KI-GDF------
PfRIO1(PF3D7_1230900)          -DFSEYNLLY----------------FCN-----------------H------------------------------------I----YI-IDV------
PfRIO2(P3D7_0420100)           -DYNEFNILV------------------D-----------------D----------DEN-----------------------V----TI-IDF------
PfSRPK1(PF3D7_0302100)         ----------------------------------------------K----------SCY----------------------------KI-CDL------
PfSRPK2(PF3D7_1443000)         -FTKHGLIIL------------------K-----------------------------------------------------------KFTTDN------
PfTKL1(PF3D7_0211700)          -DLKSSNILI------------------SI---------------------------TRD-----------------------I----KI-CDF------
PfTKL2(PF3D7_1121300)          -DLKSANILI------------------D-----------------D----------QFN-----------------------A----KL-GDF------
PfTKL3(PF3D7_1349300)          -DLKLSNILL------------------D-----------------E----------NQN-----------------------A----VI-SDF------
PfTKL4(PF3D7_0623800)          -DIKTSNILI------------------D-----------------D----------DMN-----------------------L----VM-CDF------
PfVPS15(PF3D7_0823000)         -DYHSDNYYKCNRS-----------NYKEE--------------YTN----------DEN----------------------------KKNGEYQKKYVH
PVX_091015                     -NLKPTNILL------------------D-----------------E----------ALN-----------------------A----KI-TDF------
Py02791                        -NLKPTNILL------------------D-----------------E----------SLN-----------------------A----KI-SDF------
TGGT1_209050 (TgTKL5)          -DLKTANLIV------------------D-----------------G----------VSG-----------------------L----KL-CDF------
TGGT1_225770 (TgTKL8)          -DIKTANILL------------------D-----------------K----------HNS-----------------------A----KV-ADF------
TGGT1_234970 (TgTKL2)          LDIKPGNVLL------------------------------------Q----------RGK-----------------------C----KL-ADF------
TGGT1_236240 (TgTKL6)          -DLKIENVLCCEKNRDSEGRREREDKRGDKGGRE------------E----------KSEERTGET--PEGKSP---------LDFSFKL-CDF------
TGGT1_237210 (TgTKL4)          -DIKSLNVLLTN----------------DCS---------------EGLK-------SGTK----------------------PIA---KL-ADF------
TGGT1_239130 (TgTKL7)          -DLKSPNVLL------------------G-----------------R----------RGE-----------------------I----KL-CDF------
TGGT1_253860 (TgTKL3)          -DLKLSNILL------------------D-----------------G----------DTG----------------------------KV-ADF------
TGGT1_290225                   NWASPEELLC------------------------------------------------------------------------------QI----------
TGGT1_301270 (TgTKL1)          -DLKPANILL------------------------------------K----------DGR-----------------------A----MV-TDF------

                                        910       920       930       940       950       960       970       980       990      1000
                               ....|....|....|....|....|....|....|....|....|....|....|....|....|....|....|....|....|....|....|....|
At_CDPK1                       GLSV---FFKP-------------------------------------------------------------GDK-----F-------------------
At_CTR1                        GLSRL---------------------------------------------KASTF-----------------LSS-------------------------
FIKK1(PF3D7_0102600)           GKSAP--VYTT---------------------------------------KLRHT-----------------KEMNKMI---------------------
FIKK10.1(PF3D7_1016400)        AKSTP--IYTN---------------------------------------NLRHL-----------------KNMNGTYLF-------------------
FIKK10.2(PF3D7_1039000)        AKSAP--MYTY---------------------------------------NLRHI-----------------KGDEK-----------------------
FIKK11(PF3D7_1149300)          CKSTP--IYTT---------------------------------------TLRHV-----------------KEMNHICLF-------------------
FIKK12(PF3D7_1200800)          AKSTP--LYSD---------------------------------------KLRHID---------------KKKKKKVYLF-------------------
FIKK3(PF3D7_0301200)           AKSTP--LYSF---------------------------------------KLRHL-----------------KHFEGLYSF-------------------
FIKK4.1(PF3D7_0424500)         ANSAP--IYTY---------------------------------------NNRHL-----------------KGNKRLRYY-------------------
FIKK5(PF3D7_0500900)           AKSTP--IYTR---------------------------------------KLRHV-----------------QETKGLCLF-------------------
FIKK7.1(PF3D7_0726200)         AKSTP--MYSN---------------------------------------KLRHL-----------------KESEDSYKF-------------------
FIKK8(PF3D7_0805700)           SKSTP--IYTY---------------------------------------NLRHI-----------------KDMNRLYLF-------------------
FIKK9.1(PF3D7_0902000)         GKSTP--LYTN---------------------------------------KIRHI-----------------KKVNTMCSF-------------------
FIKK9.2(PF3D7_0902100)         GKTTP--LYV-----------------------------------------LNNID---------------EHNKGHLQRF-------------------
FIKK9.4(PF3D7_0902300)         AKSTP--LYTY---------------------------------------YLRHR-----------------KNPNGLCLF-------------------
FIKK9.5(PF3D7_0902400)         SKSTP--KYSY---------------------------------------YLRHT-----------------KKMKNLCLF-------------------
FIKK9.6(PF3D7_0902500)         AKCTP--MYSY---------------------------------------YLRHT-----------------KKMDSLC---------------------
FIKK9.7(PF3D7_0902600)         AKCTP--RYTY---------------------------------------NLRHI-----------------RNPNGLCLF-------------------
Hs_ABL1                        GLSR---LMT----------------------------------------GDTYT-----------------AHA-------------------------
Hs_BTK                         GLSR---YVL----------------------------------------DDEYT-----------------SSV-------------------------
Hs_CAMK1                       GLSK---MEDP-------------------------------------------------------------GSV-----L-------------------
Hs_CDK2                        GLAR---AFGVP--------------------------------------V----------------------RTY------------------------
Hs_CK1                         GLAKK--YRDNR--------------------------------------TRQHI------------P---YRED-------------------------
Hs_CLK1                        GSAT---YDD--------------------------------------------------------------EHH-------------------------
Hs_ERK1                        GLAR---IADP-------------------------------------------------------------EHDHTGF-L-------------------
Hs_FGFR1                       GLAR---DIH----------------------------------------HIDYY-----------------KKT-------------------------
Hs_GCN2_2                      GLATDHLAFSA---------------------------------------DSKQD-----------------DQTGDLIKSDPS----------------
Hs_IGFR1                       GMTR---DIY----------------------------------------ETDYY-----------------RKG-------------------------
Hs_IRAK1                       GLAR---FSRFA---------------------------------GSSPSQSSMV----------------ART--------------------------
Hs_JAK2_1                      GISIT--VLPK-----------------------------------------------------------------------------------------
Hs_LCK                         GLAR---LIE----------------------------------------DNEYT-----------------ARE-------------------------
Hs_LYN                         GLAR---VIE----------------------------------------DNEYT-----------------ARE-------------------------
Hs_NEK1                        GIAR---VLNS-----------------------------------------------------------TVELA-------------------------
Hs_PAK1                        GFCA---QITP-------------------------------------------------------------EQSK----R-------------------
PfPK8(PF3D7_0203100)           GSCE---ELKN------------------------------------------------------------SDQS-------------------------
Hs_PKAca                       GFAK---RVKG-------------------------------------------------------------R-T-----W-------------------
Hs_RAF1                        GLAT---V------------------------------------------KSRWS----------------GSQQV------------------------
Hs_RIOK1                       SQSVE-----------------------------------------------------------------------------------------------
Hs_RIOK2                       ----------------------------------------------------------------------------------------------------
Hs_RIOK3                       SQSVE-----------------------------------------------------------------------------------------------
Hs_VGFR1                       GLAR---DIY----------------------------------------KNPDY-----------------VRK-------------------------
PBANKA_0940100                 GIKE---IEQ---------CLDINIDYSYIVFPNNVI---------KFN-NKHF------------------KNKIKKIKIVNKGSEDMLHVFSSKNHIY
PbRIO1(PBANKA_1445600)         SQSME-----------------------------------------------------------------------------------------------
PbRIO2(PBANKA_0521400)         ----------------------------------------------------------------------------------------------------
PbTKL1(PBANKA_0308500)         GLSI----------------------------------------------QNFDN-----------------KPK-------------------------
PbTKL2(PBANKA_0927000)         GLSFI--YIN----------------------------------------NNNIF---------------------------------------------
PbTKL4(PBANKA_1122700)         GKAR---FKN--------------------------------------------------------------SKL-------------------------
PF3D7_1106800                  GISK---IEN---------CLDMNIDYSYKISSNSVI---------KIN-KKEYE-----------------QKKAKKIKIVNKNNNDLLYLYDHNNNVY
PfABCk1(PF3D7_0810200)         ----------------------------------------------------------------------------------------------------
PfABCk2(PF3D7_1414500)         ----------------------------------------------------------------------------------------------------
PfARK1(PF3D7_0605300)          GLSA---HIGS-------------------------------------------------------------KHKKKG--I-------------------
PfARK2(PF3D7_0309200)          GFSC---QLKN------------------------------------------------------------KRQK-----R-------------------
PfARK3(PF3D7_1356800)          GLSA---MILG-------------------------------------------------------------KKS-----H-------------------
PfCDPK1(PF3D7_0217500)         GLSS---FFSK-------------------------------------------------------------DNK-----L-------------------
PfCDPK2(PF3D7_0610600)         GLSK---NLGT-------------------------------------------------------------GEF-----T-------------------
PfCDPK3(PF3D7_0310100)         GLAS---YFTH-------------------------------------------------------------NNYE----M-------------------
PfCDPK4(PF3D7_0717500)         GLST---HFEY-------------------------------------------------------------SKK-----M-------------------
PfCDPK5(PF3D7_1337800)         GFAS---KCMN-------------------------------------------------------------NHN-----L-------------------
PfCDPK6(PF3D7_1122800)         GLAE---LINK-------------------------------------------------------------SEGI----S-------------------
PfCDPK7(PF3D7_1123100)         GLST---LCAP-------------------------------------------------------------NEL-----L-------------------
PfCK1.1(PF3D7_1136500.2)       GLAKK--YRDSR--------------------------------------SHTHI------------P---YKEG-------------------------
PfCK2alpha(PF3D7_1108400)      GLAE---FYHPG--------------------------------------QEY-----------------------------------------------
PfCLK1(PF3D7_1445400)          GCAT---FKS--------------------------------------------------------------DYH-------------------------
PfCLK3(PF3D7_1114700)          GSAS---DIS--------------------------------------------------------------ENE-------------------------
PfCRK1(PF3D7_0417800)          GMAR---KFGHV--------------------------------------T---------------------NHNF------------------------
PfCRK3(PF3D7_0415300)          GLAR---FHSDI--------------------------------------NASNM---------------------------------------------
PfCRK4(PF3D7_0317200)          GLGVYDHFLKA-------------------------------------------------------------ETK-------------------------
```

```
PfCRK5(PF3D7_0615500)            GLCRE------------------------------------------------------------------LKNDGDM----------------------
PfGSK3(PF3D7_0312400)            GSAK---NLLAG--------------------------------------QRS-----------------------------------------------
PfIK1(PF3D7_1444500)             GLTT---RIG----------------------------------------D---------------------TQI-------------------------
PfKIN(PF3D7_1454300)             GLST---IYRK-------------------------------------------------------------NCL-----L-------------------
PfMAPK1(PF3D7_1431500)           GLAR---SISTH-----------------------------------------------------------VNENKVPI-L-------------------
PfMAPK2(PF3D7_1113900)           GLAR---TINSD--------------------------------------KDIHIVNDLEEKEENEEP-GPHNKNLKKQ-L-------------------
PfMRK(PF3D7_1014400)             GLCT---KYGYD--------------------------------------MYS---DKL------------FRDKYKKN-LN------------------
PfNEK1(PF3D7_1228300)            GLSK-----NI---------------------------------------G--------------------IESM-----A-------------------
PfNEK2(PF3D7_0525900)            GLAK---FIEQ------------------------------------------------------------TEQT-------------------------
PfNEK3(PF3D7_1201600)            GISS--------------------------------------------------------------------EQSS------------------------
PfNEK4(PF3D7_0719200)            GISK---VLEN-----------------------------------------------------------TLDYA-------------------------
PfPK1(PF3D7_0821100)             NTSI---KLKEN--------------------------------------YKY-----------------------------------------------
PfPK2(PF3D7_1238900)             GLAS---FLNS-------------------------------------------------------------PS------M-------------------
PfPK4(PF3D7_0628200)             GLVR---FIEEK----------------------------------KREKDFNNIDC-----YKDN-IYTDINQNAITSQIS------------------
PfPK5(PF3D7_1356900)             GLAR---AFGIP--------------------------------------V----------------------RKY------------------------
PfPK6(PF3D7_1337100)             GMSV---EKSDH--------------------------------------M-------------------------------------------------
PfPK7(PF3D7_0213400)             GESEY--MVD----------------------------------------K--------------K--------------I-------------------
PfPK9(PF3D7_1315100)             GLSD---FVNV-------------------------------------------------------------DQN-----I-------------------
PfPKA(PF3D7_0934800)             GFAK---IVET-------------------------------------------------------------R-T-----Y-------------------
PfPKB(PF3D7_1246900)             GLSKE-GITET-------------------------------------------------------------NLT-----K-------------------
PfPKG(PF3D7_1436600)             GCAK---KVQG-------------------------------------------------------------R-A-----Y-------------------
PfPutativeCaMK2(PF3D7_1423600)   GMAK---RVNC-------------------------------------------------------------EY------L-------------------
PfPutativeK(PF3D7_0107600)       GLASY--DYTYP--------------------------------------KNRKR-----------------KKR-------------------------
PfPutativeK(PF3D7_0321400)       SKNS---KYDN------------------------------------------------------------FDED-------------------------
PfPutativeK(PF3D7_0926100)       GDAE---ECE----------------------------------------DGIVT---------------------------------------------
PfPutativeK(PF3D7_1145200)       GWSE---HID----------------------------------------SCNLH------------P--------------------------------
PfPutativeK(PF3D7_1148000)       GLAKL--ILP----------------------------------------DTHQY---------------------------------------------
PfPutativeK(PF3D7_1316000)       GSAK---KVQRN--------------------------------------IIS-----------------------------------------------
PfPutativeK(PF3D7_1331000)       KLQNFY-FFVI-----------------------------------------------------------------------------------------
PfPutativeK(PF3D7_1441300)       GACC---INEK-------------------------------------------------------------NKL-----H-------------------
PfRIO1(PF3D7_1230900)            SQSME-----------------------------------------------------------------------------------------------
PfRIO2(P3D7_0420100)             ----------------------------------------------------------------------------------------------------
PfSRPK1(PF3D7_0302100)           GNSL---WID--------------------------------------------------------------ESR-------------------------
PfSRPK2(PF3D7_1443000)           MYEN------------------------------------------------------------------------------------------------
PfTKL1(PF3D7_0211700)            GLSV----------------------------------------------FNKYN-----------------KPK-------------------------
PfTKL2(PF3D7_1121300)            GLSFV--YMN----------------------------------------NNNVF---------------------------------------------
PfTKL3(PF3D7_1349300)            GLSTN--FSSN---------------------------------------DSPTA---------------------------------------------
PfTKL4(PF3D7_0623800)            GKAR---FKN--------------------------------------------------------------TKL-------------------------
PfVPS15(PF3D7_0823000)           ALSLP--SIKK----------------------------------KKKQIKNEEY------------PY--FNKNQNI----------------------
PVX_091015                       GICE---IEK---------CLDTHVDHSYVVYANGVTTFDAALADRKVH-RMEFS-----------------RNAFNDVPLSDVPLSDVLRVYDDEDKLH
Py02791                          GIKE---IEE---------CLDINIDYSYIVFPNNVI---------KFN-NNHF------------------KNKIKKIKIVNKGSEDMLHVFSSKNHIY
TGGT1_209050 (TgTKL5)            GKTR---SLE------------------------------------AS--GKLLL-----------------DDN-------------------------
TGGT1_225770 (TgTKL8)            GLAC---LAK------------------------------------KG--ENGCA----------------VKQTA------------------------
TGGT1_234970 (TgTKL2)            GLSR---VIPRE--------------------------------------QVTHL----------------AEGANGQALL-------------------
TGGT1_236240 (TgTKL6)            GSSNTR-QVDTA-------CC-----------------------------SREEL--------------LRTEDE-------------------------
TGGT1_237210 (TgTKL4)            GCAV---LADG------------------------------------GPGSSTGA----------------PGQSAGA----------------------
TGGT1_239130 (TgTKL7)            GLAT---LIETAPDAADARCEDGE-------------------RRDPAGGDAAAA------------QWGREARKPQLQ---------------------
TGGT1_253860 (TgTKL3)            GVATA--FVPS---------------------------------------DSPSV---------------------------------------------
TGGT1_290225                     ----------------------------------------------------------------------------------------------------
TGGT1_301270 (TgTKL1)            GVAR---VAEV---------------------------------------SFEHE-----------M---------------------------------

                                          1010      1020      1030      1040      1050      1060      1070      1080      1090      1100
                                 ....|....|....|....|....|....|....|....|....|....|....|....|....|....|....|....|....|....|....|....|
At_CDPK1                         ------------TEIVGSPYYMAPEVL---------------------------------------------------KR--------------------
At_CTR1                          ------------KSAAGTPEWMAPEVL---------------------------------------------R-----DE--------------------
FIKK1(PF3D7_0102600)             ----------------------------------------------------------------------------------------------------
FIKK10.1(PF3D7_1016400)          ---------ESCVPTIGKIRSMPPECW-------------------------------------------------------------------------
FIKK10.2(PF3D7_1039000)          ----------------------------------------------------------------------------------------------------
FIKK11(PF3D7_1149300)            ---------ESCVPKIGKISYAPPECI-QLR-----------------------------------------K---IHEKMDIKNPL----------SDL
FIKK12(PF3D7_1200800)            ---------ESCVPTIGKREY-------------------------------------------------------------------------------
FIKK3(PF3D7_0301200)             ---------ESCEPSIGKIEYMPPECW-NLL-----------------------------------------R---KYKKMKIKDPM----------RNL
FIKK4.1(PF3D7_0424500)           ---------E------------------------------------------------------------------------------------------
FIKK5(PF3D7_0500900)             ---------ESCVPTVGKSAYMPPECW-KIY-----------------------------------------K---RHRILNIKNPL----------QHL
FIKK7.1(PF3D7_0726200)           ---------ESYETHVAKSAYTPPECW-EIY-----------------------------------------W---RYYE--------------------
FIKK8(PF3D7_0805700)             ---------ESCEPTIAKGAYMPPECW-KIY-----------------------------------------W---KYDTM-------------------
FIKK9.1(PF3D7_0902000)           ---------ESCSPYVGKVPFIPPECL-KLY-----------------------------------------K---LFRERSIREPL----------KYL
FIKK9.2(PF3D7_0902100)           ---------RSYIPYVGKTKYAPPECW-NLK-----------------------------------------K---KY----------------------
FIKK9.4(PF3D7_0902300)           ---------QSCCPTVGKPKYEPPECV-DLW-----------------------------------------R---KLKEMKINNAL----------MHL
FIKK9.5(PF3D7_0902400)           ---------ESCIPSVGKTRYIPPECW-DLV-----------------------------------------K---IYIKENVDYPF----------EYL
FIKK9.6(PF3D7_0902500)           ----------------------------------------------------------------------------------------------------
FIKK9.7(PF3D7_0902600)           ---------ESCIPTIGKIEYIPPECC-EIE-----------------------------------------K---IYKELKITKPF----------SYL
Hs_ABL1                          ------------GAK-FPIKWTAPESL---------------------------------------------A-----YN--------------------
Hs_BTK                           ------------GSK-FPVRWSPPEVL---------------------------------------------M-----YS--------------------
Hs_CAMK1                         ------------STACGTPGYVAPEVL---------------------------------------------A-----QK--------------------
Hs_CDK2                          ------------THEVVTLWYRAPEILL--------------------------------------------G-----CK--------------------
Hs_CK1                           ------------KNLTGTARYASINAHL--------------------------------------------------GI--------------------
Hs_CLK1                          ------------STLVSTRHYRAPEVI---------------------------------------------L-----AL--------------------
Hs_ERK1                          ------------TEYVATRWYRAPEIML--------------------------------------------N-----SK--------------------
Hs_FGFR1                         ------------TNGRLPVKWMAPEAL---------------------------------------------F-----DR--------------------
Hs_GCN2_2                        ---------GHLTGMVGTALYVSPEVQ-----------------------------------------------G---ST--------------------
Hs_IGFR1                         ------------GKGLLPVRWMSPESL---------------------------------------------K-----DG--------------------
Hs_IRAK1                         ------------QTVRGTLAYLPEEYI---------------------------------------------K-T---GR--------------------
Hs_JAK2_1                        ------------DILQERIPWVPPECI---------------------------------------------E-----NP--------------------
Hs_LCK                           ------------GAK-FPIKWTAPEAI---------------------------------------------N-----YG--------------------
Hs_LYN                           ------------GAK-FPIKWTAPEAI---------------------------------------------N-----FG--------------------
Hs_NEK1                          ------------RTCIGTPYYLSPEIC---------------------------------------------E-----NK--------------------
Hs_PAK1                          ------------STMVGTPYWMAPEVV---------------------------------------------T-----RK--------------------
PfPK8(PF3D7_0203100)             ------------KELVGTIYYISPEIL---------------------------------------------M-----RT--------------------
Hs_PKAca                         -------------TLCGTPEYLAPEII---------------------------------------------L-----SK--------------------
Hs_RAF1                          ------------EQPTGSVLWMAPEVI---------------------------------------------RMQ--DNN--------------------
Hs_RIOK1                         ------------HDHPHALEFLRKDCA---------------------------------------------------NV--------------------
Hs_RIOK2                         -----------------------PQMV---------------------------------------------------ST--------------------
Hs_RIOK3                         ------------PTHPHGLEFLFRDCR---------------------------------------------------NV--------------------
Hs_VGFR1                         ------------GDTRLPLKWMAPESI---------------------------------------------F-----DK--------------------
PBANKA_0940100                   KYNTREINVSSNTHNSSVFFWTSPEIL---------------------------------------------K-G---KQ--------------------
PbRIO1(PBANKA_1445600)           ------------HDHPHSLEFLKRDCL---------------------------------------------------NI--------------------
```

```
PbRIO2(PBANKA_0521400)                 -----------------------PQIV---------------------------------------------------SL--------------------
PbTKL1(PBANKA_0308500)                 -----------YLGIVGTYQWTAPEIL---------------------------------------------R-----GE--------------------
PbTKL2(PBANKA_0927000)                 ------------NLTGGTPGYADPYYI---------------------------------------------------ST--------------------
PbTKL4(PBANKA_1122700)                 ------------YSNFGSYRYMAPETF---------------------------------------------S----CTS--------------------
PF3D7_1106800                          KYNTQYIDVTYNNSYPSIFYWTPPEIL---------------------------------------------R-G---KK--------------------
PfABCk1(PF3D7_0810200)                 ----------------------------------------------------------------------------------------------------
PfABCk2(PF3D7_1414500)                 ----------------------------------------------------------------------------------------------------
PfARK1(PF3D7_0605300)                  ------------SHYRGTHDYWSPEQC---------------------------------------------A-----RHQKK-----------------
PfARK2(PF3D7_0309200)                  ------------STFCGTIDYMPPEII---------------------------------------------N-----QI--------------------
PfARK3(PF3D7_1356800)                  ------------SSLCGTLVYFSPEIT---------------------------------------------S-----GT--------------------
PfCDPK1(PF3D7_0217500)                 ------------RDRLGTAYYIAPEVL---------------------------------------------R-----KK--------------------
PfCDPK2(PF3D7_0610600)                 ------------TTKAGTPYYVAPQVL---------------------------------------------D-----GK--------------------
PfCDPK3(PF3D7_0310100)                 ------------KTKAGTPYYVAPQVL---------------------------------------------T-----GS--------------------
PfCDPK4(PF3D7_0717500)                 ------------KDKIGTAYYIAPDVL---------------------------------------------H-----GT--------------------
PfCDPK5(PF3D7_1337800)                 ------------KSVVGTPYYIAPEIL---------------------------------------------R-----GK--------------------
PfCDPK6(PF3D7_1122800)                 ------------KTAAGTVLYMAPEVF---------------------------------------------K-----KK--------------------
PfCDPK7(PF3D7_1123100)                 ------------KEPCGTLAYVAPEVI---------------------------------------------T-----LQ--------------------
PfCK1.1(PF3D7_1136500.2)               ------------KNLTGTARYASINTHL--------------------------------------------------GI--------------------
PfCK2alpha(PF3D7_1108400)              ------------NVRVASRYYKGPELLI--------------------------------------------D-----LQ--------------------
PfCLK1(PF3D7_1445400)                  ------------GSIINTRQYRAPEVI---------------------------------------------L-----NL--------------------
PfCLK3(PF3D7_1114700)                  ------I-----TSYLVSRFYRAPEII---------------------------------------------L-----GF--------------------
PfCRK1(PF3D7_0417800)                  ------------TKNVVTLWYRAPELLL--------------------------------------------G-----EQ--------------------
PfCRK3(PF3D7_0415300)                  ------------TNRVITLWYRPPELLL--------------------------------------------G-----SE--------------------
PfCRK4(PF3D7_0317200)                  ------------DSNIITLQYRPPEILC--------------------------------------------------NS--------------------
PfCRK5(PF3D7_0615500)                  ------------TPTVCTIYYRPLEVLL--------------------------------------------------SK--------------------
PfGSK3(PF3D7_0312400)                  ------------VSYICSRFYRAPELML--------------------------------------------G-----ST--------------------
PfIK1(PF3D7_1444500)                   ------------NPSAGTIHYISPEQL-----------------------------------------------N---GE--------------------
PfKIN(PF3D7_1454300)                   ------------TTSCGSPFYTSPEIL---------------------------------------------L-----GQ--------------------
PfMAPK1(PF3D7_1431500)                 ------------TDYVATRWYRAPEILL--------------------------------------------G-----ST--------------------
PfMAPK2(PF3D7_1113900)                 ------------TSHVVTRWYRAPELIL--------------------------------------------L-----QE--------------------
PfMRK(PF3D7_1014400)                   -----------LTSKVVTLWYRAPELLL--------------------------------------------G-----SN--------------------
PfNEK1(PF3D7_1228300)                  ------------HSCVGTPYYWSPELLL-------------------------------------------HE-----TK--------------------
PfNEK2(PF3D7_0525900)                  ------------NTLCGTIGYMAPEIC---------------------------------------------K-----NI--------------------
PfNEK3(PF3D7_1201600)                  ------------NNNLGTLNCLSYESI---------------------------------------------K---------------------------
PfNEK4(PF3D7_0719200)                  ------------NTLIGTPYYLSPELC---------------------------------------------K-----DK--------------------
PfPK1(PF3D7_0821100)                   ------------FSYVCSRYYRAPELLF--------------------------------------------G-----SN--------------------
PfPK2(PF3D7_1238900)                   ------------NMRCGSPGYVAPEIL---------------------------------------------K-----CA--------------------
PfPK4(PF3D7_0628200)                   ------I-K---GQIIGTPGYTAPEGG-------------------------------------------------------------------------
PfPK5(PF3D7_1356900)                   ------------THEVVTLWYRAPDVLM--------------------------------------------G-----SK--------------------
PfPK6(PF3D7_1337100)                   ------------TPTVVTLWYRAPEILL--------------------------------------------K-----ST--------------------
PfPK7(PF3D7_0213400)                   ------------KGSRGTYEFMPPEFF-----------------------------------------------S---NE--------------------
PfPK9(PF3D7_1315100)                   ------------KTEAGTKAYIAPEII---------------------------------------------F-----NQT-------------------
PfPKA(PF3D7_0934800)                   -------------TLCGTPEYIAPEIL---------------------------------------------L-----NV--------------------
PfPKB(PF3D7_1246900)                   -------------SLCGTPEYLAPEII---------------------------------------------E-----QK--------------------
PfPKG(PF3D7_1436600)                   -------------TLVGTPHYMAPEVI---------------------------------------------L-----GK--------------------
PfPutativeCaMK2(PF3D7_1423600)         ------------TELCGSPHYISPELI---------------------------------------------R-----KK--------------------
PfPutativeK(PF3D7_0107600)             ------------KRKSSNLSY-------------------------------------------------------------------------------
PfPutativeK(PF3D7_0321400)             ------------GNLYGLFYIRSPQEI---------------------------------------------------RK--------------------
PfPutativeK(PF3D7_0926100)             ------------NVSGATWIYAPPELL---------------------------------------------TCHP-LKQSSDYNF--------------
PfPutativeK(PF3D7_1145200)             ------------TEWPGTLEINPPEVL---------------------------------------------R-----NT--------------------
PfPutativeK(PF3D7_1148000)             ------------YNGGGTLYYQPPECL---------------------------------------------K-----NK--------------------
PfPutativeK(PF3D7_1316000)             ------------IPYICSRWYRAPELLC--------------------------------------------G-----SM--------------------
PfPutativeK(PF3D7_1331000)             ------------HKKKNIPKYISPEIFFYL------------------------------------------------NKKIA-----------------
PfPutativeK(PF3D7_1441300)             ------------HDIVGTLSYAAPEVL---------------------------------------------N-----CNNN------------------
PfRIO1(PF3D7_1230900)                  ------------HDHPYSLEFLKRDCL---------------------------------------------------NI--------------------
PfRIO2(P3D7_0420100)                   -----------------------PQIV---------------------------------------------------SL--------------------
PfSRPK1(PF3D7_0302100)                 ------------YAEIQTRQYRAPEVI---------------------------------------------L-----KS--------------------
PfSRPK2(PF3D7_1443000)                 --------------------YIKEEQL---------------------------------------------N-----QEDD------------------
PfTKL1(PF3D7_0211700)                  -----------YLGIVGTYQWTAPEVL---------------------------------------------R-----SE--------------------
PfTKL2(PF3D7_1121300)                  ------------NLTGGTPGYADPYYI---------------------------------------------------ST--------------------
PfTKL3(PF3D7_1349300)                  ------------YAIYGNIFYAAPEVL---------------------------------------------K-----GE--------------------
PfTKL4(PF3D7_0623800)                  ------------YSNFGSYRYMAPETF---------------------------------------------S----CTT--------------------
PfVPS15(PF3D7_0823000)                 ------------KMNIDKYKYYNYHYI---------------------------------------------------NN--------------------
PVX_091015                             LYSTERIVASPSSAYPSVSFWTPPEIL---------------------------------------------R-G---QR--------------------
Py02791                                KYNTRKIDVSSNNHDSSVSFWTAPEIL---------------------------------------------K-R---KQ--------------------
TGGT1_209050 (TgTKL5)                  ------------G---GSPRYMAPECF---------------------------------------------A-T---GT--------------------
TGGT1_225770 (TgTKL8)                  ----------------GTIGYADPKYI---------------------------------------------S-----SA--------------------
TGGT1_234970 (TgTKL2)                  ------------WEGGGTLWYQPPECLIFQRQRQVKRACTSEDGRNLSPRKRALSRNPAPPGEPRAAPSTSPRESREASRRHEAKLRRGGEEDREEKQNL
TGGT1_236240 (TgTKL6)                  ------------IERNTTLMYRPPEMV---------------------------------------------------DV--------------------
TGGT1_237210 (TgTKL4)                  ------------AGWAGTVLWMAPEAL---------------------------------------------A-----KQ--------------------
TGGT1_239130 (TgTKL7)                  ---------EAHLGCVGTHHWMAPEVL---------------------------------------------R-----GE--------------------
TGGT1_253860 (TgTKL3)                  ------------LALFGNLFYAAPEVL---------------------------------------------R-----GD--------------------
TGGT1_290225                           ----------------------HPQTR---------------------------------------------------GA--------------------
TGGT1_301270 (TgTKL1)                  ------------TQGPGTEGYIAPEQR---------------------------------------------------TA--------------------

                                               1110      1120      1130      1140      1150      1160      1170      1180      1190      1200
                                       ....|....|....|....|....|....|....|....|....|....|....|....|....|....|....|....|....|....|....|....|

At_CDPK1                               ---------------------DYGPG----VDVWSA----GVIIYILL----------CGV-PP-------F---WA-----------------ET----
At_CTR1                                ---------------------PSNEK----SDVYSF----GVILWELA----------TLQ-QP-------W---GNLN---------------------
FIKK1(PF3D7_0102600)                   ----------------------------------------------------------------------------------------------------
FIKK10.1(PF3D7_1016400)                ----------------------------------------------------------------------------------------------------
FIKK10.2(PF3D7_1039000)                ----------------------------------------------------------------------------------------------------
FIKK11(PF3D7_1149300)                  NYIKDIE---------ERRKYYFDVTS---ADIYML----GVLFLRIW----------NSK-PL-------WLI--------------------ANIE--
FIKK12(PF3D7_1200800)                  ----------------------------------------------------------------------------------------------------
FIKK3(PF3D7_0301200)                   NYITNQD---------KRKDFYYDVSN---ADKYML----GVFFIWVW----------NNG-YL-------WSR--------------------SDPE--
FIKK4.1(PF3D7_0424500)                 ----------------------------------------------------------------------------------------------------
FIKK5(PF3D7_0500900)                   NAITVQD---------ERKKHYFNVTC---ADKYML----GILFIWIW----------NDG-HI-------WHC--------------------SDAS--
FIKK7.1(PF3D7_0726200)                 ----------------------------------------------------------------------------------------------------
FIKK8(PF3D7_0805700)                   ----------------------------------------------------------------------------------------------------
FIKK9.1(PF3D7_0902000)                 HSIRDTE---------ERRKYYFDVTS---ADKYML----GILFIWIW----------NDH-HL-------WEC--------------------SDPL--
FIKK9.2(PF3D7_0902100)                 ----------------------------------------------------------------------------------------------------
FIKK9.4(PF3D7_0902300)                 RNIEDQE---------ERKKYYFDVEC---VDKYML----GIMLIWIW----------DYK-YL-------WNK--------------------ADSL--
FIKK9.5(PF3D7_0902400)                 KNMYDDE---------ERKQYYFDVSC---VDKYML----GILFIAIW----------NNG-YL-------WYK--------------------SDPL--
FIKK9.6(PF3D7_0902500)                 ----------------------------------------------------------------------------------------------------
FIKK9.7(PF3D7_0902600)                 KTIIDQD---------ERKKNYFDVTS---ADNYML----GILFILIW----------VYH-FF-------WNK--------------------ADAS--
Hs_ABL1                                ---------------------KFSIK----SDVWAF----GVLLWEIA---------TYGM-SP-------Y---PGID---------------------
```

```
Hs_BTK                            ---------------------KFSSK----SDIWAF----GVLMWEIY---------SLGK-MP-------Y---ERFT---------------------
Hs_CAMK1                          ---------------------PYSKA----VDCWSI----GVIAYILL----------CGY-PP-------F---YD-----------------EN----
Hs_CDK2                           ---------------------YYSTA----VDIWSL----GCIFAEMV----------TRR-AL-------F---PG-----------------DSE---
Hs_CK1                            ---------------------EQSRR----DDMESL----GYVLMYFN----------RTS-LP-------W---QGLKAA-------------TKKQ--
Hs_CLK1                           ---------------------GWSQP----CDVWSI----GCILIEYY----------LGF-TV-------F---PTH----------------DSKE--
Hs_ERK1                           ---------------------GYTKS----IDIWSV----GCILAEML----------SNR-PI-------F---PG-----------------KHY---
Hs_FGFR1                          ---------------------IYTHQ----SDVWSF----GVLLWEIF---------TLGG-SP-------Y---PGVP---------------------
Hs_GCN2_2                         -------------------KSAYNQK----VDLFSL----GIIFFEMS---------Y----HP-------M------V---------------TASE--
Hs_IGFR1                          ---------------------VFTTY----SDVWSF----GVVLWEIA---------TLAE-QP-------Y---QGLS---------------------
Hs_IRAK1                          ----------------------LAVD----TDTFSF----GVVVLETL----------AGQ-RA-------V---KTH----------------GARTKY
Hs_JAK2_1                         --------------------KNLNLA----TDKWSF----GTTLWEIC----------SGGDKP-------L---SAL----------------DSQ---
Hs_LCK                            ---------------------TFTIK----SDVWSF----GILLTEIV---------THGR-IP-------Y---PGMT---------------------
Hs_LYN                            ---------------------CFTIK----SDVWSF----GILLYEIV---------TYGK-IP-------Y---PGRT---------------------
Hs_NEK1                           ---------------------PYNNK----SDIWAL----GCVLYELC----------TLK-HA-------F----E-----------------AGS---
Hs_PAK1                           ---------------------AYGPK----VDIWSL----GIMAIEMI----------EGE-PP-------Y---LN-----------------EN----
PfPK8(PF3D7_0203100)              ---------------------NYDCS----SDIWSL----GITIYEIV----------LCT-LP-------W---KR-----------------NQS---
Hs_PKAca                          ---------------------GYNKA----VDWWAL----GVLIYEMA----------AGY-PP-------F---FA-----------------DQ----
Hs_RAF1                           ---------------------PFSFQ----SDVYSY----GIVLYELM----------TGE-LP-------Y---SHI----------------NN----
Hs_RIOK1                          ------------------------------NDFFMRH---SVAVMTVR---------ELFE----------F--VTDPS---------I-----THEN--
Hs_RIOK2                          --------------------SHPNAEWYFDRDV-------KC----------------------------------------------------------
Hs_RIOK3                          ------------------------------SQFFQK----GGVKEALS----------ERE---------LFNAVSGLN---------I-----TADN--
Hs_VGFR1                          ---------------------IYSTK----SDVWSY----GVLLWEIF---------SLGG-SP-------Y---PGVQ---------------------
PBANKA_0940100                    ---------------------SPSLY----SDVYAY----GIILWELM----------TNS-VP-------F---NYR----------------FK----
PbRIO1(PBANKA_1445600)            ------------------------------TNFFKK----------------------------------------------------------------
PbRIO2(PBANKA_0521400)            --------------------HHENGKMYFERDV-------KCVISHFY-----------KK----------Y----------------------------
PbTKL1(PBANKA_0308500)            ---------------------GYTKK----ADIYSF----GVILWELL----------HRT-IP-------F----------------------ND----
PbTKL2(PBANKA_0927000)            --------------------HEINEQ----TEIYSF----GALILEML----------VSK-SPAIHVGKNY---SCIY---------------TRNEK-
PbTKL4(PBANKA_1122700)            ---------------------EVTEK----IDIWSL----ACCIVEIF----------CSK-YP-------Y---YNF----------------SK----
PF3D7_1106800                     ---------------------NKKFY----SDIYAF----GIILWEML----------SND-IP-------Y---NYP----------------FA----
PfABCk1(PF3D7_0810200)            ---------------------INEAKY-------------YALFKNIF---------KNSK-Y--------F----------------------------
PfABCk2(PF3D7_1414500)            ---------------------KYELYH-------------QLLAYNSF---------KKSG-IP-------V----------------------------
PfARK1(PF3D7_0605300)             ----------------KQNFGEFDQK----TDIWTL----GILAFELK----------FGR-PP-------F---GS-----------------TNE---
PfARK2(PF3D7_0309200)             ---------------------PYDCN----VDLWCL----GIVIFELL----------VGF-PP-------F---TD-----------------DT----
PfARK3(PF3D7_1356800)             ---------------------GYDWR----SDIWSL----GVLLYEML----------VGD-VP-------F---DG-----------------TK----
PfCDPK1(PF3D7_0217500)            ----------------------YNEK----CDVWSC----GVILYILL----------CGY-PP-------F---GG-----------------QN----
PfCDPK2(PF3D7_0610600)            ----------------------YDKK----CDIWSS----GVIMYTLL----------CGY-PP-------F---YG-----------------DT----
PfCDPK3(PF3D7_0310100)            ----------------------YNYK----CDMWSS----GVLFYILL----------CGY-PP-------F---FG-----------------ES----
PfCDPK4(PF3D7_0717500)            ----------------------YDEK----CDIWSC----GVILYILL----------SGC-PP-------F---NG-----------------SN----
PfCDPK5(PF3D7_1337800)            ----------------------YDKR----CDIWSS----GVIMYILL----------CGY-PP-------F---NG-----------------KN----
PfCDPK6(PF3D7_1122800)            ----------------------FTIK----CDIWSA----GVIMFFLF----------TKS-LP-------F---GG-----------------NT----
PfCDPK7(PF3D7_1123100)            ---------------------GYNHK----VDAWSI----GIILYLLL----------SGK-LP-------F---PI-----------------NK----
PfCK1.1(PF3D7_1136500.2)          ---------------------EQSRR----DDIEAL----GYVLMYFL----------RGS-LP-------W---QGLKAI-------------SKKD--
PfCK2alpha(PF3D7_1108400)         ---------------------LYDYS----LDIWSL----GCMLAGMI----------FKKEPF-------F---CG-----------------HDN---
PfCLK1(PF3D7_1445400)             ---------------------GWDVS----SDMWSF----GCVLAELY----------TGS-LL-------F---RTH----------------EHME--
PfCLK3(PF3D7_1114700)             ---------------------RYDAQ----IDVWSA----AATVFELA----------TGK-IL-------F---PGKS---------------------
PfCRK1(PF3D7_0417800)             ---------------------CYTNK----IDIWSA----GCIFAEMI----------LKK-PL-------F---VG-----------------ENE---
PfCRK3(PF3D7_0415300)             ---------------------NYMAS----VDMWSC----GCVLAELL----------TSN-PL-------F---SA-----------------ENE---
PfCRK4(PF3D7_0317200)             --------------------TLYNYS----VDIWSV----GITMCECL----------LGF-VPVT---SKFE--SSVLFKIL----VFRGI-PNEN---
PfCRK5(PF3D7_0615500)             -----------------------------------------------------------------------F----------------------------
PfGSK3(PF3D7_0312400)             ---------------------NYTTH----IDLWSL----GCIIAEMI----------LGY-PI-------F---SG-----------------QSS---
PfIK1(PF3D7_1444500)              ---------------------PFNEK----ADIFSL----GVVFFEMF--------H-----EP-------F---ST-----------------SME---
PfKIN(PF3D7_1454300)              ---------------------KYEAEL---TDVWSL----GIILFLLL----------NHR-LP-------F---NN-----------------SD----
PfMAPK1(PF3D7_1431500)            ---------------------HYTED----VDMWSL----GCIMGELL----------CGK-PL-------F---TG-----------------NST---
PfMAPK2(PF3D7_1113900)            ---------------------NYTNS----IDIWST----GCIFAELLNMMKSHINNPTNRFPL-------F---PGSSCFPLSPDHNSKKVHEKSN---
PfMRK(PF3D7_1014400)              ---------------------KYNSS----IDMWSF----GCIFAELL----------LQK-AL-------F---PG-----------------ENE---
PfNEK1(PF3D7_1228300)             ---------------------SYDDK----SDMWAL----GCIIYELC----------SGK-TP-------F---HK-----------------ANN---
PfNEK2(PF3D7_0525900)             ---------------------NYSFP----ADIWSL----GIILYELI----------SLK-PP-------F---KS-----------------NNSN--
PfNEK3(PF3D7_1201600)             --------------------FKKTNKL---SDLFQV----GCILYELA----------TLS-SP-------F---CA-----------------TN----
PfNEK4(PF3D7_0719200)             ---------------------KYSWP----SDVWAT----GCLIYELA----------TFR-TP-------F---HS-----------------TKG---
PfPK1(PF3D7_0821100)              ---------------------YYSQA----IDTWSI----GCVMGELL----------LGK-PL-------F---LG-----------------ECA---
PfPK2(PF3D7_1238900)              ---------------------SYGTK----VDIFSL----GVILFNIL----------CGY-PP-------F---RG-----------------NN----
PfPK4(PF3D7_0628200)              --------------------ALCDEK----ADIYSA----ALILLELL--------------CP------RF---TTI----------------MERY--
PfPK5(PF3D7_1356900)              ---------------------KYSTT----IDIWSV----GCIFAEMV----------NGT-PL-------F---PG-----------------VSE---
PfPK6(PF3D7_1337100)              ---------------------NYDQK----VDIWSL----GCLFMELI----------QGR-PL-------F---PG-----------------KND---
PfPK7(PF3D7_0213400)              --------------------SSYNGAK---VDIWSL----GICLYVMF----------YNV-VP-------F---SL-----------------KIS---
PfPK9(PF3D7_1315100)              --------------------INYSVFK---LDIWSL----GILLFIMT----------QGF-AP-------F----------------------KY----
PfPKA(PF3D7_0934800)              ---------------------GHGKA----ADWWTL----GIFIYEIL----------VGC-PP-------F---YA-----------------NE----
PfPKB(PF3D7_1246900)              ---------------------GHGKA----VDWWSL----GIMLYEML----------TGE-LP-------F---NN-----------------TN----
PfPKG(PF3D7_1436600)              ---------------------GYGCT----VDIWAL----GICLYEFI----------CGP-LP-------F---GN-----------------DEE---
PfPutativeCaMK2(PF3D7_1423600)    ----------------------YTMS----SDIWAL----GVMVFFML----------TGK-YP-------F---EG-----------------KN----
PfPutativeK(PF3D7_0107600)        ---------------------EYNIRK---TNILNLQ---------------------KNN-TP-------FNI-DKY----------------------
PfPutativeK(PF3D7_0321400)        ---------------------LYHDK----NNTWYI----GVLLYLFL----------FGT-YP-------F----------------I-----SNNV--
PfPutativeK(PF3D7_0926100)        -----------------LDHTKLSYK----WDIWSM----GCVFQEMM-----------NLPSP-------F----------------------QHYII-
PfPutativeK(PF3D7_1145200)        --------------------GPMTEK----IDNYAL----GMNMILFI----------SGR----------F---VCR----------------QKGI--
PfPutativeK(PF3D7_1148000)        ------------------KNLLITDK----IDIWSL----GCILYEML----------FCE-RP-------F------Q---------------FNYLE-
PfPutativeK(PF3D7_1316000)        ---------------------FYTTE----VDLWSL----GCIIFELINLC-----------PL-------F---PG----------KFKKDEYSEE---
PfPutativeK(PF3D7_1331000)        --IVDTK--------GKKKKPKHIEKYFYKNDIFCL----GLCFYYII---------MMNEDIL-------YYIN-------------------------
PfPutativeK(PF3D7_1441300)        --------------------NGYNSEK---ADIWSL----GIILYAML----------FGL-LP-------Y---DS-----------------EDK---
PfRIO1(PF3D7_1230900)             ------------------------------TNFFKK----------------------------------------------------------------
PfRIO2(P3D7_0420100)              --------------------RHVNAKMYFERDI-------NCVINHFF-----------KK----------Y----------------------------
PfSRPK1(PF3D7_0302100)            ---------------------GFNET----ADIWSF----ACMVFELV----------TGD-FL-------FNPQKGDR--------------YDKNEE-
PfSRPK2(PF3D7_1443000)            ------------------EEIVFNSN-----DFFRLNKKDNILTKDLL----------KNK-NP-------N---TSTP----------RKR--NNN---
PfTKL1(PF3D7_0211700)             ---------------------GYTKE----ADIYSF----GVILWEMI----------HRK-IP-------F----------------------SD----
PfTKL2(PF3D7_1121300)             --------------------HEINEQ----TEIYSF----GALILEML----------VSK-SPAIHVGKNY---NCIY---------------SKNEK-
PfTKL3(PF3D7_1349300)             ---------------------GFFKE----SDVWSF----AVSLWEAL----------TKK-IA-------Y---DGIS---------------------
PfTKL4(PF3D7_0623800)             ---------------------EVTEK----IDIWSA----ACCIVEIF----------SSK-YP-------Y---YNL----------------TK----
PfVPS15(PF3D7_0823000)            --------------------VNYINIY---NDIYNN----------------------V---SP-------F---SSI----------------DYN---
PVX_091015                        ---------------------EKPFY----ADVYAL----GIVLWEML----------THS-VP-------F---NYP----------------FK----
Py02791                           ---------------------NPSLY----SDVYAY----GVILWELM----------TNS-VP-------F---NYR----------------FK----
TGGT1_209050 (TgTKL5)             ---------------------LIDEK----ADIWGL----ACCLIEIF----------GGP-IP-------F---EEI----------------HS----
TGGT1_225770 (TgTKL8)             ---------------------VVTEM----TEVYSF----GMVLLELL----------T-A-RP-------P---AVL----------------NA----
TGGT1_234970 (TgTKL2)             LALADKDLEAFFRALPVIRSVPLNDK----IDIWAV----GCILYEML----------FNK-RP-------FG--PSPA---------------QNT---
TGGT1_236240 (TgTKL6)             -----------------YRGLLIGPQ----ADMWML----GCILYTLC----------FFR-HP-------F----------------------QDA---
TGGT1_237210 (TgTKL4)             ---------------------SCTEK----ADVYSF----AIVLYELL----------SNR-IP-------F---EEL----------------QGTP--
TGGT1_239130 (TgTKL7)             ---------------------PFTAA----ADVYSF----GMVLWEMV----------ARK-IP-------F---EGM----------------NS----
TGGT1_253860 (TgTKL3)             ---------------------AFYPA----SDVWSY----GVAFWEAL----------TGT-LA-------Y---EGFP---------------------
```

```
TGGT1_290225                                 ------------------------------SDIHAF----GVILWQMV----------TGL-VPH-----QF----------------MS----------
TGGT1_301270 (TgTKL1)                        ---------------------AYDRP----ADVWAF----GVVCARLL---------GLRE----------W---KKL----------------SDP---

                                                   1210      1220      1230      1240      1250      1260      1270      1280      1290      1300
                                             ....|....|....|....|....|....|....|....|....|....|....|....|....|....|....|....|....|....|....|....|
At_CDPK1                                     -----------------------EQGVA-LAIL------------RGV-------------------LDFKRDPWPQI----------------------
At_CTR1                                      -----------------------PAQVV-------------------------------------AAVGFKCKRLE------------------------
FIKK1(PF3D7_0102600)                         ----------------------------------------------------------------------------------------------------
FIKK10.1(PF3D7_1016400)                      ----------------------------------------------------------------------------------------------------
FIKK10.2(PF3D7_1039000)                      ----------------------------------------------------------------------------------------------------
FIKK11(PF3D7_1149300)                        -----------------------EDLNF-SKIF------------EAD----------------MNFDKFVIAK--------------------------
FIKK12(PF3D7_1200800)                        ----------------------------------------------------------------------------------------------------
FIKK3(PF3D7_0301200)                         -----------------------QDHVF-YELS------------QAD----------------MNFNMLERTK--------------------------
FIKK4.1(PF3D7_0424500)                       ----------------------------------------------------------------------------------------------------
FIKK5(PF3D7_0500900)                         -----------------------TDENF-YQFE------------KCD----------------MSLDVFQLTS--------------------------
FIKK7.1(PF3D7_0726200)                       ----------------------------------------------------------------------------------------------------
FIKK8(PF3D7_0805700)                         ----------------------------------------------------------------------------------------------------
FIKK9.1(PF3D7_0902000)                       -----------------------TDKNY-MKFE------------KNN----------------MSLDTFRVTK--------------------------
FIKK9.2(PF3D7_0902100)                       ----------------------------------------------------------------------------------------------------
FIKK9.4(PF3D7_0902300)                       -----------------------EDNDF-ILFK------------ENN----------------MNLDIFPTTQ--------------------------
FIKK9.5(PF3D7_0902400)                       -----------------------QDKDY-LKYS------------KSN----------------MNFNKFWTTF--------------------------
FIKK9.6(PF3D7_0902500)                       ----------------------------------------------------------------------------------------------------
FIKK9.7(PF3D7_0902600)                       -----------------------VDKEY-AEFA------------RVN----------------MDFHKVEKTY--------------------------
Hs_ABL1                                      -----------------------LSQVY-ELLE----------------------------------KDYRMERPE------------------------
Hs_BTK                                       -----------------------NSETA-EHIA----------------------------------QGLRLYRPH------------------------
Hs_CAMK1                                     -----------------------DAKLF-EQIL------------KAE-------------------YEFDSPYWDDI----------------------
Hs_CDK2                                      --------------------IDQLFRIF-RTLG------------TPDEVVWPGVTSMPDYK-----PSFPKWARQDF----------SKVVPP------
Hs_CK1                                       ----------------------KYEKISEKKMS------------TPV----------------------------------------------------
Hs_CLK1                                      -------------------HLAMMERIL-GPLP------------KHMIQ-------------KTRKRKYFHHDRLDWDEHSSAGRYVSRRCKPLKE---
Hs_ERK1                                      --------------------LDQLNHIL-GILG------------SPSQEDLNCIINMKARNY---LQSLPSKTKVAW----------AKLFPK------
Hs_FGFR1                                     -----------------------VEELF-KLLK----------------------------------EGHRMDKPS------------------------
Hs_GCN2_2                                    -----------------------RIFVL-NQLR------------DPT------------------SPKFPED---------------------------
Hs_IGFR1                                     -----------------------NEQVL-RFVM----------------------------------EGGLLDKPD------------------------
Hs_IRAK1                                     LKDLVEEEAEEAGVALRSTQ---------STLQ-------------AG----------------LAADAWAAPIAMQIYKKHLDPR--------------
Hs_JAK2_1                                    -----------------------------RKLQFY----------EDR----------------------------------------------------
Hs_LCK                                       -----------------------NPEVI-QNLE----------------------------------RGYRMVRPD------------------------
Hs_LYN                                       -----------------------NADVM-TALS----------------------------------QGYRMPRVE------------------------
Hs_NEK1                                      -----------------------MKNLV-LKII------------SGS------------------FPPVS-----------------------------
Hs_PAK1                                      -----------------------PLRAL-YLIA------------TNG------------------TPELQ--NPEKL----------------------
PfPK8(PF3D7_0203100)                         -----------------------FENYI-KTII------------NSS-----------------PKINIT----EGY----------------------
Hs_PKAca                                     -----------------------PIQIY-EKIV------------SGK-------------------VRFP----SHF----------------------
Hs_RAF1                                      -----------------------RDQII-FMVG----------------------------------RGYASPDLSKLYK--------------------
Hs_RIOK1                                     -----------------------MDAYLSKAMEIASQ------RTKEERS----------SQDHVDEEVFKRAYIPRTLN--------------------
Hs_RIOK2                                     ----------------------------------------------------------------------------------------------------
Hs_RIOK3                                     -----------------------EADFL-AEIE-------------------------------------------------------------------
Hs_VGFR1                                     -----------------------MDEDFCSRLR----------------------------------EGMRMRAPE------------------------
PBANKA_0940100                               -----------------------SHLL--ASV------------------------------------GYAKESL----------------PFQ------
PbRIO1(PBANKA_1445600)                       ------------------------------KIGYV-----------------------------------------------------------------
PbRIO2(PBANKA_0521400)                       ----------------------------------------------------------------------------------------------------
PbTKL1(PBANKA_0308500)                       -----------------------LKHPL-DIIA---------------------------------QVGYLNKQLIINN---------------------
PbTKL2(PBANKA_0927000)                       -----------CPIHCHRDKNDNDDNVFDYLIN------------------------------HINTNDYKSIYSILDYS--------------------
PbTKL4(PBANKA_1122700)                       -----------------------NTKIR-HELIV-----------NKR------------------TPHIPS----------------------------
PF3D7_1106800                                -----------------------SHIM--AVV------------------------------------GYANEEL----------------SFN------
PfABCk1(PF3D7_0810200)                       ------------------------------YVP-------------------------------------------------------------------
PfABCk2(PF3D7_1414500)                       ------------------------------KIP-------------------------------------------------------------------
PfARK1(PF3D7_0605300)                        ---------------------ERENVIM-NRIQ------------DYHWSQL-------------FCEKVKQDLIDKL----------------------
PfARK2(PF3D7_0309200)                        -----------------------QERIF-DQIK------------ELN-------------------FHFP----KSV----------------------
PfARK3(PF3D7_1356800)                        ------------------------TQIV-ESIF------------SCN-------------------LKFP----DFV----------------------
PfCDPK1(PF3D7_0217500)                       -----------------------DQDII-KKVE------------KGK-------------------YYFDFNDWKNI----------------------
PfCDPK2(PF3D7_0610600)                       -----------------------DNEVL-KKVK------------KGE-------------------FCFYENDWGSI----------------------
PfCDPK3(PF3D7_0310100)                       -----------------------DHEIL-SMVK------------KGK-------------------YQFKGKEWNNI----------------------
PfCDPK4(PF3D7_0717500)                       -----------------------EYDIL-KKVE------------AGK-------------------YTFDLPQFKKI----------------------
PfCDPK5(PF3D7_1337800)                       -----------------------NDEIL-KKVE------------KGE-------------------FVFDSNYWARV----------------------
PfCDPK6(PF3D7_1122800)                       -----------------------YDEVK-QSIF------------RDE-------------------PDYKSLK-SKL----------------------
PfCDPK7(PF3D7_1123100)                       -----------------------NTEM---NIQ------------KNY------------------VLSFKDYIWKSI----------------------
PfCK1.1(PF3D7_1136500.2)                     ----------------------KYDKIMEKKIS------------TSV----------------------------------------------------
PfCK2alpha(PF3D7_1108400)                    --------------------YDQLVKIA-KVLG------------TEDLHAYLKKYNIKLKPH--YLNILGEYERKPW----------SHFLTQ-SN---
PfCLK1(PF3D7_1445400)                        -------------------HLAMMESII-QPIP------------KNMLY----------EATKTNGSKYVNKDELKLAWPENAS--SINSIKHVKKCLP
PfCLK3(PF3D7_1114700)                        -----------------------NNHMI-KLMM-------------------------------------------------------------------
PfCRK1(PF3D7_0417800)                        --------------------IDQLFKIL-CLLG------------LPDKESYPEFYEYSFISK---NKELFKKKKIKMNV-----NNIRSHFPNIAN---
PfCRK3(PF3D7_0415300)                        --------------------TDILKIIV-NKLG------------FPNERDI------------------------------------------------
PfCRK4(PF3D7_0317200)                        -----------------------FDDLLKKEFI-------------------------------GELPKFKIDRLKMLQII-------------------
PfCRK5(PF3D7_0615500)                        ----------------------------------------------------------------------------------------------------
PfGSK3(PF3D7_0312400)                        --------------------VDQLVRII-QVLG------------TPTEDQLKEMNPNYAD------IKFPDVKSKDL----------RKVFPK------
PfIK1(PF3D7_1444500)                         -----------------------RSITL-SNLL------------KGI------------------YPEYMK----------------------------
PfKIN(PF3D7_1454300)                         -----------------------MNKLF-TQII------------KGI-------------------LYFQ----PYV----------------------
PfMAPK1(PF3D7_1431500)                       --------------------MNQLEKII-QVIG------------KPNKKDIEDIRSPFAEKI---ISSFVDLKKKNL----------KDICYK------
PfMAPK2(PF3D7_1113900)                       --------------------RDQLNIIF-NVIG------------TPPEEDLKCITKQEVIKY---IKLFPTRDGIDL----------SKKYSS------
PfMRK(PF3D7_1014400)                         --------------------IDQLGKIF-FLLG------------TPNENNWPEALCLPLYT------EFTKATKKDF----------KTYFKI------
PfNEK1(PF3D7_1228300)                        -----------------------FAQLI-SELK------------RGP------------------ELPIK-----GK----------------------
PfNEK2(PF3D7_0525900)                        -----------------------MLSVA-QKIC------------EDE------------------PDPLP----DSF----------------------
PfNEK3(PF3D7_1201600)                        -----------------------INDMI-SLFE------------DKN------------------YKSYIIKNISSIY---------------------
PfNEK4(PF3D7_0719200)                        -----------------------IQQLC-YNIR------------YAP------------------IPDLP-----------------------------
PfPK1(PF3D7_0821100)                         --------------------SDQLVEII-KILG------------TPNDEDFLSFRSVYKN------IKFPDIKPITL----------EKVIRH------
PfPK2(PF3D7_1238900)                         -----------------------VKEIF-KKNM------------RCH-------------------ISFNTKHWINK----------------------
PfPK4(PF3D7_0628200)                         -----------------------------KRLN------DF----RNY----------------YTVPDYVKIHLNPWYI--------------------
PfPK5(PF3D7_1356900)                         --------------------ADQLMRIF-RILG------------TPNSKNWPNVTELPKYD-----PNFTVYEPLPW----------ESFLKG------
PfPK6(PF3D7_1337100)                         --------------------CTQLELIY-LLLG------------DKDKLTTVDKERKDMFP------YFEINMLKD---------------AI------
PfPK7(PF3D7_0213400)                         -----------------------LVELF-NNIR------------TKN-------------------IEYPLDRNHFLYPLTN----KKSTCSN------
PfPK9(PF3D7_1315100)                         -----------------------MEKEL-KNFE------------SNT-------------------LNYA----NDI----------------------
PfPKA(PF3D7_0934800)                         -----------------------PLLIY-QKIL------------EGI-------------------IYFP----KFL----------------------
PfPKB(PF3D7_1246900)                         -----------------------RNVLF-ESIK------------YQK-------------------LNYP----KNL----------------------
PfPKG(PF3D7_1436600)                         ----------------------DQLEIF-RDIL------------TGQ-------------------LTFP----DYV----------------------
PfPutativeCaMK2(PF3D7_1423600)               -----------------------TPKVV-DEIL------------NKN-------------------INWKGKEFSSL----------------------
PfPutativeK(PF3D7_0107600)                   -----------------------NNNII------------------------------------------------------------------------
```

```
PfPutativeK(PF3D7_0321400)    -----------------------LLNYY-NIVS------------KDI------------------LFNTSQIQNKNF----------------------
PfPutativeK(PF3D7_0926100)    -------------------TFDESDQIY-EKLV----------------------------------DVFTKKLPPCIHS--------------------
PfPutativeK(PF3D7_1145200)    ----------------------ECSKVA-QTIL------------KTV-------------------HNLRHSKPPSRF---------------------
PfPutativeK(PF3D7_1148000)    ---------------------KCSKELLVNKMK--------------------------------NGLTYPKINQ-------------------------
PfPutativeK(PF3D7_1316000)    --------------------CSQIINLI-EVIG------------SPQMSFFENIKDHTSKKNTLLIKELCELNIKPL--------CWDEILGNILE---
PfPutativeK(PF3D7_1331000)    -----------------------DQYIFQSKVEMM----------QAY----------------------------------------------------
PfPutativeK(PF3D7_1441300)    ----------------------DVKEAY-NEII------------KKK-------------------IVFPKNRVNKF----------------------
PfRIO1(PF3D7_1230900)         ------------------------------KIGTIV----------------------------------------------------------------
PfRIO2(P3D7_0420100)          ----------------------------------------------------------------------------------------------------
PfSRPK1(PF3D7_0302100)        -------------------HLSFIIEVL-GNIP------------KHMID------------AGYNSHKYFNKNNYRLKNIRNIKKYGLYKILKYK----
PfSRPK2(PF3D7_1443000)        -----------------------NNEIY-YDVCYP----------SDN----------------LLKNNFQ-----------------------------
PfTKL1(PF3D7_0211700)         -----------------------MKNPL-DIIA---------------------------------HVGYANKKLSVTNK--------------------
PfTKL2(PF3D7_1121300)         -----------CPIFYHKKKHDHDDNVFDYLVN------------------------------HLNMSDYKSIYSILDYS--------------------
PfTKL3(PF3D7_1349300)         -----------------------ASEVF-CKIS------------SGELF--------------------------------------------------
PfTKL4(PF3D7_0623800)         -----------------------NVKIR-HELLV-----------NKK------------------TPHIPN----------------------------
PfVPS15(PF3D7_0823000)        -----------------------GENIF-Y---------------END-------------------SNFFKRQPIKYSN--------------------
PVX_091015                    -----------------------SHLV--ASV------------------------------------GYANEEL----------------TYD------
Py02791                       -----------------------SHLL--ASV------------------------------------GYAKETL----------------PFQ------
TGGT1_209050 (TgTKL5)         -----------------------NDGVI-DAI------------------------------------MCKRMRP-------------------------
TGGT1_225770 (TgTKL8)         -----------------------DGSIS-YLLSTIG---------TSI----------------RRTLGYIDPRA-------------------------
TGGT1_234970 (TgTKL2)         -----------------------TDPTL-ALIRDAYRGPEFPGRATPSEASRQK------RRKPRETFSLSDDRDKQLSP--------------------
TGGT1_236240 (TgTKL6)         -----------------------------SSLA-------------------------------IANANYTVPSN-------------------------
TGGT1_237210 (TgTKL4)         -----------------------AYKKLPELIPCGL---------RPN----------------ISPHALPR----------------------------
TGGT1_239130 (TgTKL7)         -----------------------PAHII-TAVG----------------------------------YGGASPVLS------------------------
TGGT1_253860 (TgTKL3)         -----------------------AGFVF-TRVA------------AGALG--------------------------------------------------
TGGT1_290225                  -----------------------TAQII-GAVA-------------------------------VGRDMLPEVDP-------------------------
TGGT1_301270 (TgTKL1)         -----------------------QHRVALSDFA-------------------------------------------------------------------

                                       1310      1320      1330      1340      1350      1360      1370      1380
                              ....|....|....|....|....|....|....|....|....|....|....|....|....|....|....|....|....
At_CDPK1                      -----------SESAKSLVKQMLDPDPTK--RL------TAQQVLAH-----------------PWI-----------------
At_CTR1                       ------IPRNLNPQVAAIIEGCWTNEPWK--RP------SFATIMDLLR---------------PLI-----------------
FIKK1(PF3D7_0102600)          ------------------------------------------------------------------------------------
FIKK10.1(PF3D7_1016400)       ------------------------------------------------------------------------------------
FIKK10.2(PF3D7_1039000)       ------------------------------------------------------------------------------------
FIKK11(PF3D7_1149300)         ---------NWPKEFKKIIQQLLHMTSRK--NL------SLKELSKN-----------------PWW-----------------
FIKK12(PF3D7_1200800)         ------------------------------------------------------------------------------------
FIKK3(PF3D7_0301200)          ---------KWPDDFKFILKKLLHMEHRR--NL------DLKDLCKH-----------------PWF-----------------
FIKK4.1(PF3D7_0424500)        ------------------------------------------------------------------------------------
FIKK5(PF3D7_0500900)          ---------TWPSGLKNILNELLHIEKRK--ML------VLRNLLSY-----------------PWF-----------------
FIKK7.1(PF3D7_0726200)        ------------------------------------------------------------------------------------
FIKK8(PF3D7_0805700)          ------------------------------------------------------------------------------------
FIKK9.1(PF3D7_0902000)        ---------SWPEDIKIMIKNLIDIKYRK--NI------KLKNLIKH-----------------PWW-----------------
FIKK9.2(PF3D7_0902100)        ------------------------------------------------------------------------------------
FIKK9.4(PF3D7_0902300)        ---------TWPEELKYIISQLLVLETRK--NL------QFKDLINH-----------------PWF-----------------
FIKK9.5(PF3D7_0902400)        ---------FWPKELKIILRQLLDLECRK--NL------NLNDLISH-----------------PWF-----------------
FIKK9.6(PF3D7_0902500)        ------------------------------------------------------------------------------------
FIKK9.7(PF3D7_0902600)        ---------HWPDGIKFIIQQLLHFESRK--NL------DLNDLINH-----------------PWF-----------------
Hs_ABL1                       ---------GCPEKVYELMRACWQWNPSD--RP------SFAEIHQAF------------------------------------
Hs_BTK                        ---------LASEKVYTIMYSCWHEKADE--RP------TFKILLSNIL---------------DVM-----------------
Hs_CAMK1                      -----------SDSAKDFIRHLMEKDPEK--RF------TCEQALQH-----------------PWI-----------------
Hs_CDK2                       ----------LDEDGRSLLSQMLHYDPNK--RI------SAKAALAH-----------------PFF-----------------
Hs_CK1                        ----EVLCKGFPAEFAMYLNYCRGLRFEE--AP---DYMYLRQLFRILF-----------------------------------
Hs_CLK1                       -FML--SQDVEHERLFDLIQKMLEYDPAK--RI------TLREALKH-----------------PFF-----------------
Hs_ERK1                       ----------SDSKALDLLDRMLTFNPNK--RI------TVEEALAH-----------------PYL-----------------
Hs_FGFR1                      ---------NCTNELYMMMRDCWHAVPSQ--RP------TFKQLVEDLDRIVA-----LTSN-QEYL-----------------
Hs_GCN2_2                     ------FDDGEHAKQKSVISWLLNHDPAK--RP------TATELLKS-----------------ELL-----------------
Hs_IGFR1                      ---------NCPDMLFELMRMCWQYNPKM--RP------SFLEIISSIK---------------EEMEPGF-------------
Hs_IRAK1                      ---PGPCPPELGLGLGQLACCCLHRRAKR--RP------PMTQVYERL----------------EKL-----------------
Hs_JAK2_1                     ----HQLPAPKWAELANLINNCMDYEPDF--RP------SFRAIIRDLN---------------SLF-----------------
Hs_LCK                        ---------NCPEELYQLMRLCWKERPED--RP------TFDYLRSVLE---------------DFF-----------------
Hs_LYN                        ---------NCPDELYDIMKMCWKEKAEE--RP------TFDYLQSVLD---------------DFY-----------------
Hs_NEK1                       --------LHYSYDLRSLVSQLFKRNPRD--RP------SVNSILEK-----------------GFI-----------------
Hs_PAK1                       -----------SAIFRDFLNRCLEMDVEK--RG------SAKELLQH-----------------QFL-----------------
PfPK8(PF3D7_0203100)          -----------SKHLCYFVEKCLQKKPEN--RG------NVKDLLNH-----------------KFL-----------------
Hs_PKAca                      -----------SSDLKDLLRNLLQVDLTK--RFG-NLKNGVNDIKNH-----------------KWF-----------------
Hs_RAF1                       ---------NCPKAMKRLVADCVKKVKEE--RP------LFPQILSSIE---------------LL------------------
Hs_RIOK1                      -------EVKNYERDMDIIMKLKEEDMAMNAQQDNILYQTVTGLKKDLSG-V--------------------------------
Hs_RIOK2                      ----------------------------------------IKDF----------------------------------------
Hs_RIOK3                      -----------------ALEKMNEDHVQKNGRK------AASFLKDDGDP--------------PLLYDE--------------
Hs_VGFR1                      ---------YSTPEIYQIMLDCWHRDPKE--RP------RFAELVEKLG---------------DLL-----------------
PBANKA_0940100                ---------NIPPFIKNLIKSCINRNKYK--RP---TFDRILIEISM-----------------IYEQINPKEEDALMSFMDG-
PbRIO1(PBANKA_1445600)        -------------------------------KH------TKSTWIKQ-------------------------------------
PbRIO2(PBANKA_0521400)        -------------------------------KI------RIEDYPLY-----------------EDIAS---------------
PbTKL1(PBANKA_0308500)        ---------NINNKLLYVLTSCLHKDKRK--RK------SFFFWSEYF------------------------------------
PbTKL2(PBANKA_0927000)        ----VNFPDFLVEKLTKLSFLCLNPNIKN--RP------SSKLVNLIL------------------------------------
PbTKL4(PBANKA_1122700)        ---------FLPNSIKKCLQKCFSFNPEE--RP------SAYEMYKAL------------------------------------
PF3D7_1106800                 ---------NIPVSIQSLIKACVNRNKYK--RP---TFEHILKTIST-----------------LYQKANTKVEDALISFMDGT
PfABCk1(PF3D7_0810200)        ------------------------------------------SIY---------------------------------------
PfABCk2(PF3D7_1414500)        ------------------------------------------KIY---------------------------------------
PfARK1(PF3D7_0605300)         -----------SPEFKDFLNLCLDKNPKK--RP------TAESLIQH-----------------PFI-----------------
PfARK2(PF3D7_0309200)         -----------SLLAQELILKLCSRTAEE--RI------SADEVKSH-----------------PWI-----------------
PfARK3(PF3D7_1356800)         -----------NPLAIDLIKKTLVVDVNK--RI------RLCELSSD-----------------PWM-----------------
PfCDPK1(PF3D7_0217500)        -----------SEEAKELIKLMLTYDYNK--RI------TAKEALNS-----------------KWI-----------------
PfCDPK2(PF3D7_0610600)        -----------SSDAKNLITKLLTYNPNE--RC------TIEEALNH-----------------PWI-----------------
PfCDPK3(PF3D7_0310100)        -----------SEEAKDLIKRCLTMDADK--RI------CASEALQH-----------------PWF-----------------
PfCDPK4(PF3D7_0717500)        -----------SDKAKDLIKKMLMYTSAV--RI------SARDALEH-----------------EWI-----------------
PfCDPK5(PF3D7_1337800)        -----------SDDAKDLICQCLNYNYKE--RI------DVEQVLKH-----------------RWF-----------------
PfCDPK6(PF3D7_1122800)        -----------SQTALHMLKLMLQKDYNK--RP------MASVLLHH-----------------PWF-----------------
PfCDPK7(PF3D7_1123100)        -----------SSSAKDLISKLLELNVEK--RI------SANEALEH-----------------IWV-----------------
PfCK1.1(PF3D7_1136500.2)      ----EVLCRNASFEFVTYLNYCRSLRFED--RP---DYTYLRRLLKDLF-----------------------------------
PfCK2alpha(PF3D7_1108400)     ----IDI---AKDEVIDLIDKMLIYDHAK--RI------APKEAMEH-----------------PYF-----------------
PfCLK1(PF3D7_1445400)         LYKI-----IKHELFCDFLYSILQIDPTL--RP------SPAELLKH-----------------KFL-----------------
PfCLK3(PF3D7_1114700)         ---------------------------------------EYKGKFSH-----------------KMIK----------------
PfCRK1(PF3D7_0417800)         ----QFSGLYLSDNGLDLLQKMLHYNPQC--RI------SAQEALNH-----------------PYF-----------------
PfCRK3(PF3D7_0415300)         --------------------KYLR------------------------------------------------------------
```

```
PfCRK4(PF3D7_0317200)            -FTDIYGRRILSDEGLDLIDQFLSYDYKN--RI------TANEALKH-----------------KWF-----------------
PfCRK5(PF3D7_0615500)            ---------------------------------------ELSNKHHHKSHA---------------------------------
PfGSK3(PF3D7_0312400)            ---------GTPDEAINLITQFLKYEPLK--RL------NPIEALAD-----------------PFF-----------------
PfIK1(PF3D7_1444500)             ----------ADNKKFQFLSSLLAINPQE--RC------CAYNLLHE-----------------SVL-----------------
PfKIN(PF3D7_1454300)             -----------SKNAKHLLQNMLNVNFKK--RF------TMNKIKNH-----------------IWF-----------------
PfMAPK1(PF3D7_1431500)           ----------ASNESLDLLEKLLQFNPSK--RI------SAENALKH-----------------KYV-----------------
PfMAPK2(PF3D7_1113900)           ----------ISKEGIDLLESMLRFNAQK--RI------TIDKALSH-----------------PYL-----------------
PfMRK(PF3D7_1014400)             ----------DDDDCIDLLTSFLKLNAHE--RI------SAEDAMKH-----------------RYF-----------------
PfNEK1(PF3D7_1228300)            -----------SKELNLLIKNLLNLSAKE--RP------SALQCLGY-----------------QII-----------------
PfNEK2(PF3D7_0525900)            -----------SKDLINLCYWMLKKDWKD--RP------TIYDIIST-----------------DYI-----------------
PfNEK3(PF3D7_1201600)            -----------SQKLVNVISKLLSLNTLE--RL------EV-------------------------------------------
PfNEK4(PF3D7_0719200)            --------NIYSKELNNIYKSMLIREPSY--RA------TVQQLLVS-----------------DIV-----------------
PfPK1(PF3D7_0821100)             ---------NCSKESLDLLSELLQFNPQK--RI------KLCHALLH-----------------NYF-----------------
PfPK2(PF3D7_1238900)             -----------SESVKEIILWMCCKNPDD--RC------TALQALGH-----------------QWF-----------------
PfPK4(PF3D7_0628200)             -----------------LMLQMSKPNPAD--RP------SAADVYSKI------------------------------------
PfPK5(PF3D7_1356900)             ----------LDESGIDLLSKMLKLDPNQ--RI------TAKQALEH-----------------AYF-----------------
PfPK6(PF3D7_1337100)             ----------DDEHTLDLISKMLIYDPNY--RI------SSKEALKH-----------------PCF-----------------
PfPK7(PF3D7_0213400)             --------NFLSNEDIDFLKLFLRKNPAE--RI------TSEDALKH-----------------EWL-----------------
PfPK9(PF3D7_1315100)             -----------SDDLKDLISLMLNVDPNK--RP------IIVEILNH-----------------RWF-----------------
PfPKA(PF3D7_0934800)             -----------DNNCKHLMKKLLSHDLTK--RYG-NLKKGAQNVKEH-----------------PWF-----------------
PfPKB(PF3D7_1246900)             -----------SPKAVDLLTKLFEKNPKK--RLG-SGGTDAQEIKKH-----------------PFF-----------------
PfPKG(PF3D7_1436600)             ----------TDTDSINLMKRLLCRLPQG--RIG-CSINGFKDIKDH-----------------PFF-----------------
PfPutativeCaMK2(PF3D7_1423600)   -----------SIEAVDFLKRLLERNEKK--RL------TAYQALHH-----------------PWI-----------------
PfPutativeK(PF3D7_0107600)       -----------RKKKEILFKNCCTKNNIPT-KE--I------------------------------------------------
PfPutativeK(PF3D7_0321400)       -----------SPFVLDFLEKALEKNYVK--RP------TLKELLKH-----------------PWI-----------------
PfPutativeK(PF3D7_0926100)       --------KIENSPFADIIRLCLNYDPNL--RP------TASEIVQL-----------------L-------------------
PfPutativeK(PF3D7_1145200)       ---------RENLKAWDLFVKLTSSNPIE--RL------SLQNVLDH-------------------------------------
PfPutativeK(PF3D7_1148000)       ---------KISNATLSYIQYLLNFDYEL--RP------SIEEALSY-----------------PIF-----------------
PfPutativeK(PF3D7_1316000)       ----I-----EEKELINIIDGLLKWNPNE--RL------NIDAVLSN-----------------PYF-----------------
PfPutativeK(PF3D7_1331000)       ---------ITKPELFSLVRSMLIYDYKQ--RP---DWSALAQII---------------------------------------
PfPutativeK(PF3D7_1441300)       -----------STSVRSLLLAMLNINPQN--RL------SLDEVMKH-----------------EWL-----------------
PfRIO1(PF3D7_1230900)            -------------------------------QQ-----TQPTNLYTQ-------------------------------------
PfRIO2(P3D7_0420100)             -------------------------------KI------KIQEYPL--------------------------------------
PfSRPK1(PF3D7_0302100)           ----YNLPEKEISPLCSFLLPMLSVDPQT--RP------SAYTMLQH-----------------PWL-----------------
PfSRPK2(PF3D7_1443000)           ---------ISDTLFVDFLSSLLQIDPSK--RC------NAMEALKH-----------------PWL-----------------
PfTKL1(PF3D7_0211700)            ---------NIPDQLKYILHSCLHKNTHK--RK------SFLFWSEY-------------------------------------
PfTKL2(PF3D7_1121300)            ----VNFPDFLVEKLTKLSFLCLNPNIKN--RP------SSKLVN---------------------------------------
PfTKL3(PF3D7_1349300)            ----LPIPKDIPMELSELLKSMLEYDFTK--RP------LFNVIAKKL------------------------------------
PfTKL4(PF3D7_0623800)            ---------FLPNSIKKCLQKCFSFQPED--RP------TAYEMYKS-----------------L-------------------
PfVPS15(PF3D7_0823000)           -------THNIKEQLNDDFQENIKYNDKNIYRQ-----KQNEQVTQKKSQG---------------------------------
PVX_091015                       ---------DIPEPIQGLIKSCVHRNMYK--RP---TFGHILAVLSR-----------------LYEKANTKCEDALMSFMDGT
Py02791                          ---------NIPPFIKNLIKSCVNRNKYK--RP---TFDRILIELSM-----------------IYEKINPKEEDALMSFMDG-
TGGT1_209050 (TgTKL5)            -----AVPSWFHPAARRLFERCFSWSPRE--RP------SALEIARTLE---------------LF------------------
TGGT1_225770 (TgTKL8)            -----RFPPHLAQSLAILAFSCIQADERH--RP------PFRSIVQQL----------------QLL-----------------
TGGT1_234970 (TgTKL2)            ---HSGDENEPSDACLDLLARLLSYDPSG--RP------TVEEALCH-----------------PFF-----------------
TGGT1_236240 (TgTKL6)            ---------KYSEELIDLLHWLLSADPND--RP------TSAQLLSLLDS--------------SSL-----------------
TGGT1_237210 (TgTKL4)            ---------DLPEGLRDLMESCWRQEPAN--RP---PFTHIVKALEII------------------------------------
TGGT1_239130 (TgTKL7)            ---------PSPPPLREILERCLSPSPQN--RP------SFAWCAQQLQ---------------SLY-----------------
TGGT1_253860 (TgTKL3)            ----LHIPSDFPPPLRFLMSRILAFEPSE--RP------SFEEVAQALTDLQANALSEIERDLDDFF-----------------
TGGT1_290225                     ---------HVPFSLRVLIRCCTHPDPRL--RP------SFHQIVSL-------------------------------------
TGGT1_301270 (TgTKL1)            ---------LKDAFLINLCLACLERRPLM--RP------TFHQIVQRLID--------------KIF-----------------
```

Supporting Information 5 – MAFFT Alignment – *Pf*pTKL orthologs and homologs

```
                             10        20        30        40        50        60        70        80        90       100
                    ....|....|....|....|....|....|....|....|....|....|....|....|....|....|....|....|....|....|....|....|
PF3D7_1106800       M----------------------------------------------------------------GNTLDSNKPKNF-------VTYADYKYIGKLNNK-
PFIT_1107600        M----------------------------------------------------------------GNTLDSNKPKNF-------VTYADYKYIGKLNNK-
PPRFG01_1107500     M----------------------------------------------------------------GNTLDSNKPKNF-------VTYADYKYIGKLNNK-
PRCDC_1105300       M----------------------------------------------------------------GNTLDSNKPENF-------VTYADYKYIGKLNNK-
PRG01_1104000       M----------------------------------------------------------------GNTLDSNKPENF-------VTYADYKYIGKLNNK-
PBILCG01_1104000    M----------------------------------------------------------------GNTLDSNKPKNF-------VTYADYNYIGKLNNN-
PBLACG01_1104300    M----------------------------------------------------------------GNTLDSNKPKNS-------VTYENYKYIGKLDNK-
PADL01_1105000      M----------------------------------------------------------------GNTLDSNKSKNL-------FTYENYKYRGKLNNN-
PGABG01_1103900     M----------------------------------------------------------------GNTLDSNKSKNL-------FNYENYKYRGKLNNN-
PGSY75_1106800      M----------------------------------------------------------------GNTLDSNKSKNL-------FNYENYKYRGKLNNN-
PGAL8A_00334100     M----------------------------------------------------------------GNNVSS-KINNF-------VEFENYKYIGELDKN-
PRELSG_0904000      M----------------------------------------------------------------GNDGS--KANNY-------VEFDNYKYIGELNKN-
PocGH01_09013500    M----------------------------------------------------------------GNSVDS-KPGGS-------VHFEKQKYVGELNNE-
PYYM_0941700        M----------------------------------------------------------------GNGNSV-QAKKY-------IKFEKYYYVGDLNVD-
PY02790             M----------------------------------------------------------------GNGNSV-QAKKY-------IKFEKYYYVGDLNVD-
PY02791             ----------------------------------------------------------------------------------------------------
PBANKA_0940100      M----------------------------------------------------------------GNINSI-QTKNY-------IKFEKYYYAGDLNVD-
PCHAS_0904200       M----------------------------------------------------------------GNTNST-ETNNY-------IKFDKYYYIGDLNVD-
PCHAS_090420        M----------------------------------------------------------------GNTNST-ETNNY-------IKFDKYYYIGDLNVD-
YYE_02751           M----------------------------------------------------------------GNTNSS-ETKNY-------IKFDKYYYIGDLNVD-
YYG_01693           M----------------------------------------------------------------GNTNSS-ETKNY-------IKFDKYYYIGDLNVD-
PmUG01_09016400     M----------------------------------------------------------------GNTTSS-KTSKY-------VEFENYRYIGELNND-
C922_04039          M----------------------------------------------------------------GNSPDS-KPIKG-------REFSTYRYIGELNSQ-
PcyM_0908600        M----------------------------------------------------------------GNSADS-KPIKG-------REFSTYRYIGELNNQ-
PKNOH_S120122800    M----------------------------------------------------------------GNSSNS-KPIKG-------RDFSTYRYIGELNSE-
PKNH_0904400        M----------------------------------------------------------------GNSSNS-KPIKG-------RDFSTYRYIGELNSE-
PVP01_0907600       M----------------------------------------------------------------GNSPDS-KPIRG-------RDFRGYRYIGELNDE-
PVX_091015          M----------------------------------------------------------------GNSPDS-KPIRG-------RDFRGYRYIGELNDE-
THRCLA_22740        M----------------------------------------------------------------------------------------QLYRGYTKATI
THRCLA_04632        M---------------------------------------------------------------------------------------------------
BESB_019990         M----------------------------------------------------------------NYNKHS-------------------RYGLGCNGR-
BN1204_042460       M----------------------------------------------------------------GNRPLSPSPYDVDVPLGYTLLVSGDFYKGSLNDR-
TGGT1_290225        M----------------------------------------------------------------GNRPVLPSPYDLDVPMGYTLLVSGDFYRGRLDRR-
TGDOM2_290225       ----------------------------------------------------------------------------------------------------
BN1205_083850       M----------------------------------------------------------------GNRPVLPSPYDLDVPMGYTLLVSGDFYRGRLDRR-
HHA_290225          M----------------------------------------------------------------GNRPVSPSPYDLDVPLGYTLLVSGDFYKGRLDRR-
ETH_00000110        M----------------------------------------------------------------GSSNVRPRVEAAFP-RGTLLLSTGDRYKGPTDPS-
ENH_0020460         M----------------------------------------------------------------GSSNVRPRVEAAFP-RGTLLLSTGDRYKGPIDPS-
EMWEY_00023200      M----------------------------------------------------------------DSSNVRAEVEGL-----------------------
Hs_RAF1             M----------------------------------------------------------------EHIQGAWKTISNGF---------------------
Pa_RAF1             M----------------------------------------------------------------EHIQGAWKTISNGF---------------------
Mm_RAF1             M----------------------------------------------------------------EHIQGAWKTISNGF---------------------
Rn_RAF1             M----------------------------------------------------------------EHIQGAWKTISNGF---------------------
Bt_RAF1             M----------------------------------------------------------------EHIQGAWKTISNGF---------------------
Gg_RAF1             M----------------------------------------------------------------EHIQGAWKTISNGF---------------------
Xl_RAF1             M----------------------------------------------------------------EHIQGAWKTLSNGF---------------------
Ce_RAF1             M----------------------------------------------------------------SRINFKKSSASTTP---------------------
GNI_162980          MVELQSPHLHPSPLLPPQVPRLTTAPGCLQNVRPPEAEHGDGTPDLNGRNRVRQVSTRHLTTQHLAEEQESPYTARSGQSGG--LEASGLRFRGGSNTAV

                            110       120       130       140       150       160       170       180       190       200
                    ....|....|....|....|....|....|....|....|....|....|....|....|....|....|....|....|....|....|....|
PF3D7_1106800       ----------NEHH------------GIGIILYNSGESFYGSFINGKKEGKGIYIDKNLTRYIN------------------TWVDNKVFGKVK----VV
PFIT_1107600        ----------NEHH------------GIGIILYNSGESFYGSFINGKKEGKGIYIDKNLTRYIN------------------TWVDNKVFGKVK----VV
PPRFG01_1107500     ----------NEHH------------GIGIILYNSGESFYGSFINGKKEGKGIYIDKNLTRYIN------------------TWVDNKVFGKVK----VV
PRCDC_1105300       ----------NQHH------------GIGIILYNSGESFYGSFINGKKEGKGIYIDKNLTRYIN------------------TWVDNNVFGKVK----VV
PRG01_1104000       ----------NQHH------------GIGIILYNSGESFYGSFINGKKEGKGIYIDKNLTRYIN------------------TWVDNNVFGKVK----VV
PBILCG01_1104000    ----------NEPH------------GIGIILYNSGESFYGSFINGKKEGKGIYIDKNLTRYIN------------------TWVDDKVLGKVK----VV
PBLACG01_1104300    ----------NEPH------------GIGIILYNSGESFYGSFINGKKEGKGIYIDKYLTRYIN------------------TWVDDKVLGKVK----VV
PADL01_1105000      ----------NQPH------------GKGIILYNSGESFYCSFINGKKEGKGIYIDKNLTRYIN------------------IWKDDKVIGKVK----VL
PGABG01_1103900     ----------NQPH------------GKGIILYNSGESFYCSFINGKKEGKGIYIDKNLTRYIN------------------IWKDDKVIGKVK----VL
PGSY75_1106800      ----------NQPH------------GKGIILYNSGESFYCSFINGKKEGKGIYIDKNLTRYIN------------------IWKDDKVIGKVK----VL
PGAL8A_00334100     ----------NLPH------------GKGLILYNSGESFYGYFMNGKRNGKGIYIDKKLTKYKC------------------NWKDDKVFKKLK----IN
PRELSG_0904000      ----------NLPN------------GKGLILYNSGESFYGYFLNGKKNGRGIYIDRKLTKYKC------------------TWKDDKVFKKLK----IN
PocGH01_09013500    ----------NLPH------------GKGTVFHYTGESFYGSFYNGKRNGRGIFIDRYLTKYIC------------------NWKDNKVFDKVT----VK
PYYM_0941700        ----------NLPH------------GRGLILYENGDSFFGHFINGKKHGKGIYINKNLTKYIS------------------KWKYDNISNKVK----VK
PY02790             ----------NLPH------------GRGLILYENGDSFFGHFINGKKHGKGIYINKNLTKYIS------------------KWKYDNISNKVK----VK
PY02791             ----------------------------------------------------------------------------------------------------
PBANKA_0940100      ----------NLPH------------GRGLMLYENGNSFFGHFINGKKHGKGIYIDKNLTKYIS------------------KWKYDHISNKVK----VK
PCHAS_0904200       ----------NLPH------------GRGLILYENGNSFYGNFIKGKKHGQGIYIDKNLTKYIS------------------NWAYGNILNKVK----VK
PCHAS_090420        ----------NLPH------------GRGLILYENGNSFYGNFIKGKKHGQGIYIDKNLTKYIS------------------NWAYGNILNKVK----VK
YYE_02751           ----------NLPH------------GRGLILYENGDSFFGHFINGKKHGQGIYIDQNLIKYIS------------------YWAYGNILNKVK----VK
YYG_01693           ----------NLPH------------GRGLILYENGNSFFGHFINGKKHGQGIYIDKNLTTYIS------------------NWAYGNILNKVK----VK
PmUG01_09016400     ----------RMPN------------GKGVVLYNSGESFYGSFLNGRKNGKGVYIDRMLTKYIC------------------NWKNDKVFRGVH----II
C922_04039          ----------GVPN------------GKGLILHNSGEAFYGFFSNGKKYGIGIYIDKDLTKYIC------------------NWVNDKVDRELK----VK
PcyM_0908600        ----------REPN------------GKGLILHNSGEAFYGFFSNGKKNGIGIYIDKKLTKYIC------------------KWVNDKAHRELK----VK
PKNOH_S120122800    ----------SVPD------------GKGIILHNSGEAFYGYFSNGKKNGIGIYIDKKLTKYIS------------------NWVNDKVDGELK----VK
PKNH_0904400        ----------SVPD------------GKGIILHNSGEAFYGYFSNGKKNGIGIYIDKKLTKYIS------------------NWVNDKVDGELK----VK
PVP01_0907600       ----------REPN------------GKGLILHNSGEAFYGSFSNGKKNGIGIYINNKLTKYIC------------------NWVNDTVDGELK----VK
```

```
PVX_091015          ----------REPN------------GKGLILHNSGEAFYGSFSNGKKNGIGIYINNKLTKYIC------------------NWVNDTVDGELK----VK
THRCLA_22740        ---------YAIPV-------------------------------------------------------------------SVYK---------------
THRCLA_04632        ----------------------------------------------------------------------------------------------------
BESB_019990         ----------ASPS----------------LLPRSA-----SFSPGASAGSFASLSPCACAPVA-----------------------AGAAHLR----QA
BN1204_042460       ----------RMPC------------GRGILLLREGHCHVGSFWNGLRHGHGAFVEMRRGGCIA--IHS------------GTWWRDAAVGQFR----SF
TGGT1_290225        ----------RMPL------------GRGILLLREGHCHVGSFQDGLRHGPGSFVEMRKGGCVA--IHS------------GTWSRDAANGFFR----SV
TGDOM2_290225       ----------------------------------------------------------------------------------------------------
BN1205_083850       ----------RMPL------------GRGILLLREGHCHVGSFQDGLRHGPGSFVEMRKGGCVA--IHS------------GTWSRDAANGFFR----SV
HHA_290225          ----------RMPC------------GRGILLLREGHCHVGSFQGGLRHGHGSFVEMRKGGCVA--IHS------------GTWSRDAANGCFR----SV
ETH_00000110        ----------GVPC------------GRGILLLKDGHCFSGTFNKGRREGPGVFVELRSGGGLA--IHQ------------GTWRECFLHGPCR----SL
ENH_0020460         ----------GVPS------------GRGILLLKDGHCFSGTFNKGRREGPGVFVELRSGGGLA--IHQ------------GTWHECFLHGPCR----SL
EMWEY_00023200      ----------STEL-------------------ADGHCFSGTFKKGLREGPGVFIELRTGGGIA--IHQALSRVSSRGYNIGEWVGCFLHGPCR----SL
Hs_RAF1             --------------------------GFKDAVFDGSSCISPTIVQQFGYQRRASDDGKL-TD-----------------------PSKTSNTIR-----V
Pa_RAF1             --------------------------GFKDAVFDGSSCISPTIVQQFGYQRRASDDGKL-TD-----------------------PSKTSNTIR-----V
Mm_RAF1             --------------------------GLKDAVFDGSSCISPTIVQQFGYQRRASDDGKL-TD-----------------------SSKTSNTIR-----V
Rn_RAF1             --------------------------GLKDAVFDGSSCISPTIVQQFGYQRRASDDGKL-TD-----------------------SSKTSNTIR-----V
Bt_RAF1             --------------------------GFKDTVFDGTSCISPTIVQQFGYQRRASDDGKL-TD-----------------------PSKTSNTIR-----V
Gg_RAF1             --------------------------GLKDSVFDGPNCISPTIVQQFGYQRRASDDGKI-SD-----------------------TSKTSNTIR-----V
Xl_RAF1             --------------------------GFKESVFEGSSCMSPTIVHQFGYQRRASDDGKL-SD-----------------------TSKTSSTMR-----V
Ce_RAF1             ----------TSPH------------CPSPRLISLPRCASSSIDRKDQASPMASPSTPLYPKHSDSLHS-LSGHHSAG---GAGTSDKEPPKFKYKMIMV
GNI_162980          SVAQRAAQVVATPQSPARPTAPPDKGGNGVILYPDGSAYAGRVVAGKRHGRGTFIHEATDVQYD-----------------GEWFEDLWDGEGR----LY

                           210       220       230       240       250       260       270       280       290       300
                    ....|....|....|....|....|....|....|....|....|....|....|....|....|....|....|....|....|....|....|....|
PF3D7_1106800       PYNSNRVYYFYYKNYMIEKCIYFDNN--------------------------------------------------------------------------
PFIT_1107600        PYNSNRVYYFYYKNYMIEKCIYFDNN--------------------------------------------------------------------------
PPRFG01_1107500     PYNSNRVYYFYYKNYIIEKCIYFDNN--------------------------------------------------------------------------
PRCDC_1105300       PYNSNRVYYFYYKNYMIEKCIYFDNN--------------------------------------------------------------------------
PRG01_1104000       PYNSNRVYYFYYKNYMIEKCIYFDNN--------------------------------------------------------------------------
PBILCG01_1104000    PYNSNRVYYFYYKNDMIEKCIYFYNK--------------------------------------------------------------------------
PBLACG01_1104300    PYNSNIVYYFYYKNDMIEKCIYFYNN--------------------------------------------------------------------------
PADL01_1105000      PYNSNRVYYFYYKHDIIEKCIYFDN---------------------------------------------------------------------------
PGABG01_1103900     PYNSNRVYYFYYKHDIIEKCIYFDN---------------------------------------------------------------------------
PGSY75_1106800      PYNSNRVYYFYYKHDIIEKCIYFDN---------------------------------------------------------------------------
PGAL8A_00334100     PFNSNKVFYFFYNNGLIESCKIYNTSKN------------------------------------------------------------------------
PRELSG_0904000      PFNSNTVFYFFYNNGVIESCKIYNTAKN------------------------------------------------------------------------
PocGH01_09013500    PFNSDTVFVFLYKDGAIRTCKICKTKGE------------------------------------------------------------------------
PYYM_0941700        QFDNDTVYLFYYKNGLIDHCKIYDYISNKKKKKKKKKKKKKKKKKXXXXXXXXXXXXXXXXXXXXXXXXXXXXXXXXXXXXXXXXXXXXXXXXXXXXXX
PY02790             QFDNDTVYLFYYKNGLIDHCKIYXYISN------------------------------------------------------------------------
PY02791             ----------------------------------------------------------------------------------------------------
PBANKA_0940100      QFDSDIVYLFYYKNGLIDHCKVYEYISN------------------------------------------------------------------------
PCHAS_0904200       RFDNDAVYLFYYKNDIIDHCKVYGYIS-------------------------------------------------------------------------
PCHAS_090420        RFDNDAVYLFYYKNDIIDHCKVYGYIS-------------------------------------------------------------------------
YYE_02751           RFDNDAFMTIY-----------------------------------------------------------------------------------------
YYG_01693           RFDNDAVYLFYYKNGLIDHCKVYDYI--------------------------------------------------------------------------
PmUG01_09016400     PFNSDRVFFMLYENGQVASCEMFGIVKRNSSARSDENIDKKSSGRSSGRNSGRNSGRNSGR---------------------------------------
C922_04039          PLHAEKVFSFFYSNGVIKSCRVYRVGPK------------------------------------------------------------------------
PcyM_0908600        PFHAKKVFSFFYSNEVIESCKVYRVGPK------------------------------------------------------------------------
PKNOH_S120122800    PFHSEKVFSFLYSNGVIESCTIYRSRPK------------------------------------------------------------------------
PKNH_0904400        PFHSEKVFSFLYSNGVIESCTIYRSRPK------------------------------------------------------------------------
PVP01_0907600       PFHAEKVFSFFYSNGVIHSCRVYRGGPK------------------------------------------------------------------------
PVX_091015          PFHAEKVFSFFYSNGVIHSCRVYRGGPK------------------------------------------------------------------------
THRCLA_22740        ----------------------------------------------------------------------------------------------------
THRCLA_04632        ----------------------------------------------------------------------------------------------------
BESB_019990         PPSSFGRIASAW----------------------------------------------------------------------------------------
BN1204_042460       FLPQKFFILSEWREGILCRASSPQLF--------------------------------------------------------------------------
TGGT1_290225        FLPQKLFIDSEWREGSLCWASSAQPL--------------------------------------------------------------------------
TGDOM2_290225       ----------------------------------------------------------------------------------------------------
BN1205_083850       FLPQKLFIDSEWREGSLCWASSAQPL--------------------------------------------------------------------------
HHA_290225          FLPQKVFIVSEWREGSLCRASAAQPL--------------------------------------------------------------------------
ETH_00000110        LLPERLLVYSEWSLGRR-----------------------------------------------------------------------------------
ENH_0020460         LLPERLLVYSEWSLGRR-----------------------------------------------------------------------------------
EMWEY_00023200      LLPERLLIYSEFYGGKR-----------------------------------------------------------------------------------
Hs_RAF1             FLPNKQRTVVNVRNGMSL----------------------------------------------------------------------------------
Pa_RAF1             FLPNKQRTVVNVRNGMSL----------------------------------------------------------------------------------
Mm_RAF1             FLPNKQRTVVNVRNGMSL----------------------------------------------------------------------------------
Rn_RAF1             FLPNKQRTVVNVRNGMSL----------------------------------------------------------------------------------
Bt_RAF1             FLPNKQRTVVNVRNGMSL----------------------------------------------------------------------------------
Gg_RAF1             FLPNKQRTVVNVRNGMTL----------------------------------------------------------------------------------
Xl_RAF1             YLPNKQRTVVNVRSGMSL----------------------------------------------------------------------------------
Ce_RAF1             HLPFDQHSRVEVRPGETA----------------------------------------------------------------------------------
GNI_162980          SVRDMTVLHGLWSRGELLDQGIRRDLACGCT---------------------------------------------------------------------

                           310       320       330       340       350       360       370       380       390       400
                    ....|....|....|....|....|....|....|....|....|....|....|....|....|....|....|....|....|....|....|....|
PF3D7_1106800       --------------------------------------------------I--NNKESH-HKNNI-------------------YNNY-DNNSYNNNSCD
PFIT_1107600        --------------------------------------------------I--NNKESH-HKNNI-------------------YNNY-DNNSYNNNSCD
PPRFG01_1107500     --------------------------------------------------I--NNKESH-HKNNI-------------------YNNY-DNNSYNNNSCD
PRCDC_1105300       --------------------------------------------------I--NNKESY-HENNIYNNYHNSS---YNNYDNNSYNNY-HDSSYNNCSCD
PRG01_1104000       --------------------------------------------------I--NNKESY-HENNI-------------------YNNY-HDSSYNNCSCD
PBILCG01_1104000    --------------------------------------------------I--NNKEPY-HKNNI-------------------Y---------NNSYCD
PBLACG01_1104300    --------------------------------------------------I--YNKKSY-HKNNI-------------------YDYY-NNCFHNNCYCD
```

```
PADL01_1105000    --------------------------------------------------I--NNKESY-HKHNN-------------------YSNDLHNNSHNHSYSD
PGABG01_1103900   --------------------------------------------------I--NNKEYY-HKHNT-------------------YTNYLHNNSHNHSYSD
PGSY75_1106800    --------------------------------------------------I--NNKEYY-HKHNT-------------------YTNYLHNNSHNHSYSD
PGAL8A_00334100   -----------------------------------------------------KEKKNK-NKNKILYKGNNYELRNEDKINR-----YDNNNKINNNNNQ
PRELSG_0904000    --------------------------------------------------------NKK-KKKKILHKRNSQEMKGTDQINKNNNEIVNNNEKKNNNNNE
PocGH01_09013500  -----------------------------------------------------TKEGRK-SSNQSIPCISH-----TNACVHGKKGYEAGYEGMREGKQK
PYYM_0941700      XXXXXXXXXXXXXXXXXXXXXXXXXXXXXXXXXXXXXXXXXXXXXXFFFNLKKKKKKKK-KKKKTILEDTK----------------IQNCKEKSFVNKK
PY02790           ---------------------------------------------------KXKKKKKK-KKNDXILEDTK----------------IQNCKEKSFVNKK
PY02791           ----------------------------------------------------------------------------------------------------
PBANKA_0940100    -----------------------------------------------------KKKKKK-KKNDGILEDSEIQ-NRSINFEKDNIMIIQNCQEKSIVNKK
PCHAS_0904200     -----------------------------------------------------SHKKKT-IKHEQVLVDSE----------------IQNCQEKSVDNQK
PCHAS_090420      -----------------------------------------------------SHKKKT-IKHEQVLVDSE----------------IQNCQEKSVDNQK
YYE_02751         ------------------------------------------------------QKKKK-KKNNVLLVDSE----------------IQNWEEKNVDNQK
YYG_01693         -----------------------------------------------------SKKKKK-KKNDLLLVDSE----------------IQNCQEKNIDNQK
PmUG01_09016400   -------------------------------------------------N-SGRNSGRS-SGRNSGRSSGHSSGRSSGNLNMSDLNPLRSSNGVTVSASA
C922_04039        ---------------------------------------------------LGRSLDAE-PKGGTPR-WGE-----LHSPDI--DNEPDRADVYTIEGGD
PcyM_0908600      ---------------------------------------------------PEHSLDAQ-PKRRTPK-GGE-----THSAEI--KNGLDRAHVSTVESGD
PKNOH_S120122800  ---------------------------------------------------PERSLHAK-WK-RTPK-GGE-----THSVYI--NDELAMADACTVEDAN
PKNH_0904400      ---------------------------------------------------PERSLHAK-WK-RTPK-GGE-----THSVYI--NDELAMADACTVEDAN
PVP01_0907600     ---------------------------------------------------SERGLDEE-QSRGTPKGGGE-----PQSVNI--RNKL-----------D
PVX_091015        ---------------------------------------------------SERGLDEE-QSRGTPKGGGE-----PQSVNI--RNKL-----------D
THRCLA_22740      ----------------------------------------------------------------------------------------------------
THRCLA_04632      ----------------------------------------------------------------------------------------------------
BESB_019990       ----------------------------------------------------------------------------------------------------
BN1204_042460     --------------------------------------------------LDIRNTSSVPLKDDALGQLAE-----------------------------
TGGT1_290225      --------------------------------------------------LPMHGPSSL-LKDATLSQAGR-----------------------------
TGDOM2_290225     ----------------------------------------------------------------------------------------------------
BN1205_083850     --------------------------------------------------LPMHGPSSL-LKDATLSQAGR-----------------------------
HHA_290225        --------------------------------------------------LRMHGPSSL-LSEPTLSHAGR-----------------------------
ETH_00000110      --------------------------------------------------VLVRGPGVL-SEARSPVAA-------------------------------
ENH_0020460       --------------------------------------------------VLVRGPGLL-SEARSPVAA-------------------------------
EMWEY_00023200    --------------------------------------------------VQLRGPAPL-YEGPPP----------------------------------
Hs_RAF1           -------------------------------------------------------HDCL-MK--------------------------------------
Pa_RAF1           -------------------------------------------------------HDCL-MK--------------------------------------
Mm_RAF1           -------------------------------------------------------HDCL-MK--------------------------------------
Rn_RAF1           -------------------------------------------------------HDCL-MK--------------------------------------
Bt_RAF1           -------------------------------------------------------HDCL-MK--------------------------------------
Gg_RAF1           -------------------------------------------------------HDCL-MK--------------------------------------
Xl_RAF1           -------------------------------------------------------HDCL-MK--------------------------------------
Ce_RAF1           -------------------------------------------------------RDAI-SK--------------------------------------
GNI_162980        -------------------------------------------------WAEEYCRGVL-VNHALVAVQGE-----------------------------

                          410       420       430       440       450       460       470       480       490       500
                  ....|....|....|....|....|....|....|....|....|....|....|....|....|....|....|....|....|....|....|....|
PF3D7_1106800     DEE-----------KRKY----PIGVTKFKEDLSN--------YIHSTHI-MKKN-------------------------NKLFNKN--D-------KEY
PFIT_1107600      DEE-----------KRKY----PIGVTKFKEDLSN--------YIHSTHI-MKKN-------------------------NKLFNKN--D-------NEY
PPRFG01_1107500   DEE-----------KRKY----PIGVTKFKEDLSN--------YIHSTHI-MKKN-------------------------NKLFNKN--D-------NEY
PRCDC_1105300     DEE-----------KRKY----PIGDIKFKEDLSN--------YIHSTHI-KKKN-------------------------NKLFNKN--D-------KEY
PRG01_1104000     DEE-----------KRKY----PIGDIKFKEDLSN--------YIHSTHI-KKKN-------------------------NKLFNKN--D-------KEY
PBILCG01_1104000  DEE-----------KRKR----SFGDTKFKEDLSN--------YIHTSHI-KKKN-------------------------NKIFNKN-DD-------KEY
PBLACG01_1104300  DEE-----------KRKY----SHGDTNFKEQLSN--------YIHTTHL-DKKN------------------------NNKLFNKN--D-------KEY
PADL01_1105000    KEE-----------KRNY----SIEHTNYKEELYN--------YIDTTHM-KKKNNNNNNNNNK----IFNNNNIF---NNKIFNNN--H-------SEY
PGABG01_1103900   KEE-----------KRNY----SIEHTNYKEQLYN--------YIDTTHI-KKKNNNNKNNNN-----NNNNNNNF---SNDIFNKN--H-------DEY
PGSY75_1106800    KEE-----------KRNY----SIEHTNYKEQLYN--------YIDTTHI-KKKNNNNNKNNNKNNS-NNNNNNNF---SNDIFNKN--H-------NEY
PGAL8A_00334100   NNDVHITSV-----KNKY----PNRENFDINKC-N--------I-----LESGNNTYINDNENNFNN-NESNKDNL--SDNKEKSIK-KE-------EIE
PRELSG_0904000    NEDINIKSV-----KNNF----IKRESFDINGNIN----------ENRQTENGNNAYINNGENNYND-DKSNKIDI--YNNKKRNIK-KK-------EIK
PocGH01_09013500  DTHIDGDTP-----KELYKGGNLPRKSPPFEEKGS--------GSENGNNANNKSNGINNYSYSSSS-DEDLQNDLITGSDKIKNKK-GD-------NLD
PYYM_0941700      DEIID---------KDFE--------KDDSDKVDN--------FSNKINLEQSQNNMLKYKKKKKKF-DKMNYLN------------------------D
PY02790           DEIID---------KDFE--------KDDSDKVDN--------FSNKINLEQSQNNMLKYKKKKKKF-DKMNYLN------------------------D
PY02791           ----------------------------------------------------------------------------------------------------
PBANKA_0940100    EEIIDIPNN-----KYNE--------NDDTDKVDN--------FSKKINLEQSQNNMLKYKKKKTF--DEMNSLN------------------------D
PCHAS_0904200     DETIDIPND-----KHKV-----------------------------------KNDILKYKKTKKTS-DKINPLN------------------------D
PCHAS_090420      DETIDIPND-----KHKV-----------------------------------KNDILKYKKTKKTS-DKINPLN------------------------D
YYE_02751         DETIDIPND-----KHKV-----------------------------------KNDILKYKKKKNIS-DDINSLN------------------------N
YYG_01693         DETIDIPND-----KHKV-----------------------------------KNDILKYKKKKKIS-DEMNSLN------------------------D
PmUG01_09016400   QEEINRERSEEGPNKNGVTNDCIKRENNLMQDPSH--------LNEKINVSEEKQTCIYNSQHYGAS-KREENNNLYKNSNK-KEVK-ID-------SLT
C922_04039        KGCASILPVS----KPSYV--LPFGRIQQGTAASR----------QSGHREDAIESSRMGQLQISME-DSHDEANI--PVNELTKKR-SDKQLKEGGTWK
PcyM_0908600      KGCVSILPVS----KPGSVCFLPRGRVQQGRTASREGGHHGSGRLQSGHREDAIESSRVDQLQVAMEKESHDEANI--PVNERPKKI-IDEELKKNGTWE
PKNOH_S120122800  KGWVSTLPVR----KPGYV--LPIGRIQQGRTVSR----------EIGYHEDVIEICPMDQRQVSME-NPHDEANI--QMNELTKKK-IDEQLKKE-ILE
PKNH_0904400      KGWVSTLPVR----KPGYV--LPIGRIQQGRTVSR----------EIGYHEDVIEICPMDQRQVSME-NPHDEANI--QMNELTKKK-IDEQLKKE-ILE
PVP01_0907600     GEGVSFIPVS----QPGYF--FPAGRIHHGRTAGP----------GSDHREDAIE-------------SSHDEANI--PADELNKKR-IDEPLKGDDTWK
PVX_091015        GEGVSFIPVS----QPGYF--FPAGRIHHGRTAGP----------GSDHREDAIE-------------SSHDEANI--PADELNKKR-IDEPLKGDDTWK
THRCLA_22740      ----------------------------------------------------------------------------------------------------
THRCLA_04632      ----------------------------------------------------------------------------------------------------
BESB_019990       ------------------------------------------------------------------------------PFSSSPSRR-TD----------
BN1204_042460     ---------------------------RKREEAVSR------------------------------------------PLSCAHATR-TD-------TEK
TGGT1_290225      ----------------------------RREEAGTR------------------------------------------AGSRAFGVSCTD-------AIT
TGDOM2_290225     ----------------------------------------------------------------------------------------------------
BN1205_083850     ----------------------------RREE---------------------------------------------------------D-------AIT
HHA_290225        ----------------------------TREEASSG------------------------------------------PGSRAFGTPCTD-------ASK
ETH_00000110      ------------------------------------------------------------------------------PGPTACSAH--D-------AVG
ENH_0020460       ------------------------------------------------------------------------------PGPTACSAH--D-------AVG
```

```
EMWEY_00023200      ------------------------------------------------------------------------------PFPGAPWTG-------------
Hs_RAF1             ----------------------------------------------------------------------------------------------------
Pa_RAF1             ----------------------------------------------------------------------------------------------------
Mm_RAF1             ----------------------------------------------------------------------------------------------------
Rn_RAF1             ----------------------------------------------------------------------------------------------------
Bt_RAF1             ----------------------------------------------------------------------------------------------------
Gg_RAF1             ----------------------------------------------------------------------------------------------------
Xl_RAF1             ----------------------------------------------------------------------------------------------------
Ce_RAF1             ----------------------------------------------------------------------------------------------------
GNI_162980          ----------------------------------------------------------------------------------------AD----------

                           510       520       530       540       550       560       570       580       590       600
                    ....|....|....|....|....|....|....|....|....|....|....|....|....|....|....|....|....|....|....|....|
PF3D7_1106800       NIFSSSLSYSSDSEN-INLLDILKKKKNKKNKKN-----------------------------------------KKK-----KNKKTKNTQILSCTQHK
PFIT_1107600        NIFSSSLSYSSDSEN-INLLDILKKKKKKKKKKK-----------------------------------------KKK-----KNKKTKNTQILSCTQHK
PPRFG01_1107500     NIFSSSLSYSSDSEN-INLLDILKKKKNKKN--------------------------------------------KKK-----KNKKTKNTQILSCTQHK
PRCDC_1105300       NIFPSSLSYSSDSEN-INLLDILKKKKKKKKKKKKKKXXXXXXXXXXXXXXXXXXXXXXXXXXXXXXXXXKKKKKKKK-----KKKKKKHTQILSCTRHK
PRG01_1104000       NISPSSLSYSSDSEN-INLLDIL----------------------------------------------------KKK-----KKKKKKHTQILSCTQHK
PBILCG01_1104000    DNLSSLLSYSSDSEN-INLLDILKKKI------------------------------------------------KKK-----KKKKKKNTQMLFCTQHK
PBLACG01_1104300    NIFSSSLSYSSDSEN-INLLDILKKK-------------------------------------------------IKK-----KNQKNKKKQISFCTQHN
PADL01_1105000      NTSSSSLSYSSDSGN-INLLDIL----------------------------------------------------KKK-----KKKS----QILFPTQHK
PGABG01_1103900     NNSSSSLSYSSDSGN-INLLDIL-----------------------------------------------------KK-----KKKS----QILFSTQDK
PGSY75_1106800      NNSSSSISYSSDSGN-INLLDILK---------------------------------------------------KKK-----KKKS----QILFSTQHK
PGAL8A_00334100     NFFCSNSYNSLVS-G-SSFIDILK---------------------------------------------------KK------KKKNLYISNNLE-----
PRELSG_0904000      DLFCSSSYDSSLS-D-SSLINILK---------------------------------------------------KKKNNNKNKKKRQYISDYLEVRKEN
PocGH01_09013500    DFFFSTSNDSLQSFG-STYIEVSE---------------------------------------------------KKKK----KKRKSQHTGGGEVPNEG
PYYM_0941700        SIFYSSCESISKSIG-SDYISDFE---------------------------------------------------KKE-----KQINKQEEDKFKKTNEN
PY02790             SIFYSSCESISKSIG-SDYISDFE---------------------------------------------------KKE-----KQINKQEEDKFKKTNEN
PY02791             ----------------------------------------------------------------------------------------------------
PBANKA_0940100      SIFCSSCESTSKSIG-SDYISHFE---------------------------------------------------KKE-----KQINKQEDE-LKKSKEN
PCHAS_0904200       SIFYSSCESTSKSIG-SDYISDFE---------------------------------------------------KKE-----KQTNEQEEKGFEKVKEN
PCHAS_090420        SIFYSSCESTSKSIG-SDYISDFE---------------------------------------------------KKE-----KQTNEQEEKGFEKVKEN
YYE_02751           SIFYSSCESTFKSIG-SDYISNFE----------------------------------------------------------------HEENGFEKVKEN
YYG_01693           SIFYSSCESTSKSIG-SDYISDFE---------------------------------------------------KKE-----KQTNEQEEKGVEKVKEN
PmUG01_09016400     NFFYSSSDCSDEFVS-SNYITSST---------------------------------------------------KKE-----LKKKANCSPSFHSPKEE
C922_04039          DIFCTSSDSCSHFIR-PHFVKLGK-----------------------------------------------------------KKKCYRDTHQSGTTRKD
PcyM_0908600        DIFYTSSDSCSHFIRPPHFVKLGG-----------------------------------------------------------KKKCYRDTYQCGTTRKD
PKNOH_S120122800    NIFYTSSDSSNHFIR-PHFVMLGR-----------------------------------------------------------RKKCYRDTYPCETTPKD
PKNH_0904400        NIFYTSSDSSNHFIR-PHFVMLGR-----------------------------------------------------------RKKCYRDTYPCETTPKD
PVP01_0907600       DIFYTSSDSCSHLIG-AHFIKMGG-----------------------------------------------------------KKKCHRDARLGGTTRKD
PVX_091015          DIFYTSSDSCSHLIG-AHFIKMGG-----------------------------------------------------------KKKCHRDARLGGTTRKD
THRCLA_22740        ----------------------------------------------------------------------------------------------------
THRCLA_04632        ----------------------------------------------------------------------------------------------------
BESB_019990         -----------------------------------------------------------------------------------DEQPAPLAASAS-----
BN1204_042460       EAALSAAGYVGNE----------------------------------------------------------------------REEPGDREAGTETAALD
TGGT1_290225        E-TFSARNVCLRG----------------------------------------------------------------------REASGEKAGKPETRAER
TGDOM2_290225       ----------------------------------------------------------------------------------------------------
BN1205_083850       E-TFSARNVCLRG----------------------------------------------------------------------REASGEKAGKQETRA--
HHA_290225          E-IFSSRNVCLRD----------------------------------------------------------------------REAPGDQTGKPETRA--
ETH_00000110        PLGSLVQDETAQG------ATQGS---------------------------------------------------CS------TEGRGSLSSGRSDAA--
ENH_0020460         PLASPVQDETAQG------APQGS---------------------------------------------------CS------TGGRGSLSSGRSDAA--
EMWEY_00023200      ---GPLYGGGPQGRG-----PQGG---------------------------------------------------PSPY----GQQGGPPRSVQEVNG--
Hs_RAF1             --------------A--------------------------------------------------------------------LKVRGLQPECCAVFRL-
Pa_RAF1             --------------A--------------------------------------------------------------------LKVRGLQPECCAVFRL-
Mm_RAF1             --------------A--------------------------------------------------------------------LKVRGLQPECCAVFRL-
Rn_RAF1             --------------A--------------------------------------------------------------------LKVRGLQPECCAVFRL-
Bt_RAF1             --------------A--------------------------------------------------------------------LKVRGLQPECCAVFRL-
Gg_RAF1             --------------A--------------------------------------------------------------------LKVRGLQPECCAVFRL-
Xl_RAF1             --------------S--------------------------------------------------------------------LKVRGLQPECCAVFRL-
Ce_RAF1             ---------------------------------------------------------------------L-------------LKKRNITPQLCHVNA--
GNI_162980          -----ADDSSSESSS--------------------------------------------------------------------RDTRLPEPQSVRHRTGS

                           610       620       630       640       650       660       670       680       690       700
                    ....|....|....|....|....|....|....|....|....|....|....|....|....|....|....|....|....|....|....|....|
PF3D7_1106800       M----Y-EHNMNESNFTKKDNVNCEHTDKMNISLHEK------------NDKKNEKKNEKKNKKKKLFKYFS--NNIENLIIENYQTWSLREVIQWLML-
PFIT_1107600        M----Y-EHNMNESNFTKKDNVNCEHTDKMNISLHEK------------NDKKNEKKNEKKNKKKKLFKYFS--NNIENLIIENYQTWSLREVIQWLML-
PPRFG01_1107500     M----Y-EHNMNESNFTKKDNVNCEHTDKMNISLHEKNDKKNDKKNEKKNDKKNEKKNEKKNKKKKLFKYFS--NNIENLIIENYQTWSLREVIQWLML-
PRCDC_1105300       M----Y-EHNMNESNFTKKDNLNYEHTNKVNMSFYE------------------------KNKKKKLFKYFS--NNIENLIIENYQTWSLREVIQWLML-
PRG01_1104000       M----Y-EHNMNESNFTKKDNLNYEHTNKMNMSFYE------------------------KNKKKKLFKYFS--NNIENLIIENYQTWSLREVIQWLML-
PBILCG01_1104000    M----Y-EHNMNQSNFTKKHNLNCQHMNKMNMSMYQ------------------------KNEKRYIFKYFS--NNIENLNIENYQVWSLREVIQWLML-
PBLACG01_1104300    M----Y-EHNMNQSKIIKKENLNCQSINKMYIPIYQ------------------------NNETKNLFKYFS--NNIENLIIENYQSWSLREVIQWLML-
PADL01_1105000      I----F-HHNINQSKFIKNDNLNGQRKNKMNISIYK------------------------KNERKNIFKYFS--NNIENLNIENYETWSLKEVIQWLML-
PGABG01_1103900     I----Y-HHNTNQSKFIKNENLNNQRNNKMNISIYK------------------------KNERKNIFKYFS--NNIENLNIENYEAWSLKEVIQWLLL-
PGSY75_1106800      I----Y-HHNTNQSKFIKNENLNNQRNNKMNISIYK------------------------KNERKNIFKYFS--NNIENLNIENYEAWSLKEVIQWLLL-
PGAL8A_00334100     -----------------------------------------------------------KKKKKKKKHTLKSFINNNDNLHIENYELWSTKEVVQWLVL-
PRELSG_0904000      HNE--M-KDREFNSNLEDKEKG-------------------------------------KKKKKKTTDTLRSFIDKNENLNIENYELWERKEVVQWLVL-
PocGH01_09013500    ----------EGEDGSEEGEQKNV--------------------------------------EGTGLISLGG--RNGSTLKIEAYELWTRREVAQWLTL-
PYYM_0941700        Y----M-NHSENTNSSSNFENNKIM----------------------------------NISDESIILRLNNLINNNTDLKIENYEMWNKEQVAQWLTM-
PY02790             Y----M-NHSENTNSSSNFENNKIM----------------------------------NISDESIILRLNNLINNNTDLKIENYEMWNKEQVAQWLTM-
PY02791             ----------------------------------------------------------------------------------------------------
PBANKA_0940100      Y----M-NHSEYSNSSTNFENNKIK----------------------------------DISDESIMLRLKNIINNNTDLKIENYELWNKEQVAQWLSL-
PCHAS_0904200       R----M-DHIEGSKPSTIFENDNIMDNKKV---------------------------RTDMSEEMIMLRLKDIINNNTDLKIENYELWNKEQVAHWLSL-
```

```
PCHAS_090420          R----M-DHIEGSKPSTIFENDNIMDNKKV---------------------------RTDMSEEMIMLRLKDIINNNTDLKIENYELWNKEQVAHWLSL-
YYE_02751             -------------KSSTNFENDNIMDNKKV---------------------------GEDMSEENIMLRLKDIINNNTDLKIENYELWNKEQVAHWLSL-
YYG_01693             C----M-GHSECSKSSTNFENDNIMDNKKV---------------------------RTDMSEENIMLKLKDIINNNTDLKIENYELWNKEQVAHWLSL-
PmUG01_09016400       KERDGN-INKQIKQQLKENEMSNHIDDLRVYGKVHSA--------------------NRRYKNKYMLHNIGN--PNKGNINIENYEMWSRKEIAQWLTL-
C922_04039            LTI--I-THTSTKKD--------------------------------------------ERKTKHMIKRLSK--ENSSSLKIDQYESWSRKEVAQWLSL-
PcyM_0908600          LTI--I-THTSTKKE--------------------------------------------QRKTKHMIKRLTK--QNSSSLKIDKYESWSRKEVAQWLSL-
PKNOH_S120122800      ITI--M-THTSTKKE--------------------------------------------EKKTKHIIKRLTK--QNSNSLKIEKYEAWSRKEVAHWLSL-
PKNH_0904400          ITI--M-THTSTKKE--------------------------------------------EKKTKHIIKRLTK--QNSNSLKIEKYEAWSRKEVAHWLSL-
PVP01_0907600         LSI--V-THTLAKKE--------------------------------------------ERKTKHMIKRLTK--QNSNSLRIDKYESWSRREVAQWLSL-
PVX_091015            LSI--V-THTLAKKE--------------------------------------------ERKTKHMIKRLTK--QNSNSLRIDKYESWSRREVAQWLSL-
THRCLA_22740          ----------------------------------------------------------------------------------------------------
THRCLA_04632          ----------------------------------------------------------------------------------------------------
BESB_019990           -----------------------------------------------------------GFSPSHASASANAAKEAGEAPRLQVEPALS-----------
BN1204_042460         A----H-ARREPGAHAL----------------------------------------TLPASPWHLDLAASQNGPSPRAEAFVDPYAWSPEELCARLSSF
TGGT1_290225          R----LRERRLRERDLL----------------------------------------TLPANAWHLNLAASPSGRTPTLEAFVDPYSWSSEELCSRLSTF
TGDOM2_290225         ----------------------------------------------------------------------------------------------------
BN1205_083850         -------ERRLRERDLL----------------------------------------TLPANAWHLNLAASPSGRTPTLEAFVDPYSWSSEELCSRLSTF
HHA_290225            -------ERWLRERDVL----------------------------------------TLPANAWHVDLAASPSGRTPRLQAFVDPYSWSSEELCSRLSSF
ETH_00000110          -----------------------------------------------------------NGAARHSRSAAVAAAAAASGRSCLEPVHWTPSELARWLRLA
ENH_0020460           -----------------------------------------------------------NGTARHSRSAA-AAAAAASDRSCLEPVHWTPSELARWLRLA
EMWEY_00023200        -----------------------------------------------------------GSGYGHSAASVSAASAAAAAASPVSVEADSENAEHSW----
Hs_RAF1               -----------------------------------------------------------LHEHKGKKARL----------------DWNTDAA----SLI
Pa_RAF1               -----------------------------------------------------------LHEHKGKKARL----------------DWNTDAA----SLI
Mm_RAF1               -----------------------------------------------------------LQEHKGKKARL----------------DWNTDAA----SLI
Rn_RAF1               -----------------------------------------------------------LQEHKGKKARL----------------DWNTDAA----SLI
Bt_RAF1               -----------------------------------------------------------LHEHKGKKARL----------------DWNTDAA----SLI
Gg_RAF1               -----------------------------------------------------------VTEPKGKKVRL----------------DWNTDAA----SLI
Xl_RAF1               -----------------------------------------------------------IQDPKG-KLRL----------------DWNTDAM----SLV
Ce_RAF1               -----------------------------------------------------------SSDPKQESIEL----------------SLTMEEIAS--RLP
GNI_162980            R---------------------------------------------------------LGSGPGSGCCQHQV--VSPNPGSVLHPAALPKHGSAQYLSSG

                               710       720       730       740       750       760       770       780       790       800
                      ....|....|....|....|....|....|....|....|....|....|....|....|....|....|....|....|....|....|....|....|
PF3D7_1106800         ----------CNVPVKWLISFYKNNITGDKLKYININTIRNELGI-----IAYGHAIKILQLIKNLQVMAYNKKF-------------------------
PFIT_1107600          ----------CNVPVKWLISFYKNNITGDKLKYININTIRNELGI-----IAYGHAIKILQLIKNLQVMAYNKKF-------------------------
PPRFG01_1107500       ----------CNVPVKWLISFYKNNITGDKLKYININTIRNELGI-----IAYGHAIKILQLIKNLQVMAYNKKF-------------------------
PRCDC_1105300         ----------CNVPVKWLISFYNNNITGDKLKYININTLRNELGI-----IAYGHAIKILQLIKNLQVMAYNKKF-------------------------
PRG01_1104000         ----------CNVPVKWLISFYNNNITGDKLKYININTLRNELGI-----IAYGHAIKILQLIKNLQVMAYNKKF-------------------------
PBILCG01_1104000      ----------CNVPVKWIISFYKNNITGDKLKYININTVRNELGI-----IAYGHAIKILQLIKNLQVMAYNKKF-------------------------
PBLACG01_1104300      ----------CNVPIKWIISFYKNNITGDKLKYININTIRNELGI-----IAYGHAIKILQLIKNLQVMAYNKKF-------------------------
PADL01_1105000        ----------CNVPIKWIISFYKNNITGDKLKYININTVRNEFGI-----IAFGHAIKILQLIKNLQVTAYNKKF-------------------------
PGABG01_1103900       ----------CYVPIKWIISFYKNNITGDKLKYININTVRNELGI-----IAYGHAIKILQLIKNLQVMAYNKKF-------------------------
PGSY75_1106800        ----------CYVPIKWIISFYKNNITGDKLKYININTVRNELGI-----IAYGHAIKILQLIKNLQVMAYNKKF-------------------------
PGAL8A_00334100       ----------CNVPIKWIMAIYKNNITGFKLNNLNLNTIRNNLGI-----FSYGHAIKLLQLIKNLRIAAYNKRL-------------------------
PRELSG_0904000        ----------CNVPIKWIIAIYKNNITGYKLNNLNLNTIRNNLGI-----LSYGHAIKLLQLIKNLRIMAYNKRL-------------------------
PocGH01_09013500      ----------CNVPIKWVLGAYKNKITGEKLNSLNIDAIRNELGI-----LPYGHAIKMLQLIKNLRVMAYNKRF-------------------------
PYYM_0941700          ----------CKVPMKWALTVYKNNINGQKLNNLNLYFIRNKLGI-----LSYGQSIKFLQLIKNLRVTAYNTRL-------------------------
PY02790               ----------CKVPMKWALTVYKNNINGQKLNNLNLYFIRNKLGI-----LSYGQSIKFLQLIKNLRVTAYNTRL-------------------------
PY02791               ----------------------------------------------------------------------------------------------------
PBANKA_0940100        ----------CNVPMKWALSVYKNNINGQRLNNLNLYFIRNKLGI-----LSYGQAIKFLQLIKNLRVTAYNTRF-------------------------
PCHAS_0904200         ----------CSVPMKWVVSVYKNNITGHKLNNINLHYIRNKLGI-----LPYGQAIKLLQLIKNLRVTAYNTRL-------------------------
PCHAS_090420          ----------CSVPMKWVVSVYKNNITGHKLNNINLHYIRNKLGI-----LPYGQAIKLLQLIKNLRVTAYNTRL-------------------------
YYE_02751             ----------CSVPMKWVVSVYKNNISGQKLNNINLHYIRNKLEI-----LPYGQAIKLLQLIKNLRVTAYNTRL-------------------------
YYG_01693             ----------CSVPMKWVVSVYKNNITGPKLNNINLHYIRNKLGI-----LPYGQAIKLLQLIKNLRVTAYNTRL-------------------------
PmUG01_09016400       ----------CNVPIKWIISVYKNNITGNMLNDLNIDTIREQLGI-----LPYGHAIKLLQLIKNLRIMAYNKKF-------------------------
C922_04039            ----------CNAPIKWITAFYRNNVTGDRLDRINIEIIRNQLGI-----LPYGHAIKLLQLIKNLR---------------------------------
PcyM_0908600          ----------CNAPIKWITAFYRNNVTGSMLDTMNIEVIRNQLGI-----LPYGHAIKLLQLIKNLRVMAYNKRF-------------------------
PKNOH_S120122800      ----------CNVPIKWITAFYRNNVTGSALDRINIEMIRNQMGI-----LPYGHAIKLLQLIKNLRVMAYNKRF-------------------------
PKNH_0904400          ----------CNVPIKWITAFYRNNVTGSALDRINIEMIRNQMGI-----LPYGHAIKLLQLIKNLRVMAYNKRF-------------------------
PVP01_0907600         ----------CNTPIKWITSFYRNNITGDMLDRMNIEIVRNQLGI-----LPYGHAIKLLQLIKNLRVMAYNKRF-------------------------
PVX_091015            ----------CNTPIKWITSFYRNNITGDMLDRMNIEIFRNQLGI-----LPYGHAIKLLQLIKNLRVMAYNKRF-------------------------
THRCLA_22740          --------------------------------------------------------------INNLDIS-------------------------------
THRCLA_04632          ----------------------------------------------------------------------------------------------------
BESB_019990           ----------GARPPSFPPSLAPQAGGG----------------------------------LEALRALA------------------------------
BN1204_042460         ATDG------GKLPAEFIDRCRAKALTGARFMNLTQDILRRDLGL-----TTFGQRSRVLQLVKALKVDYNKHSRYYSGALPRSPPSSSFPFP------G
TGGT1_290225          VMEG------GKLPPDFIERCRKKALTGAEFMSLTHETLRRDLEL-----STFGQRSRVLQLVKSLKAEYTKHSRY-SSAFSPSP-SYSSPFPAPSLLSS
TGDOM2_290225         -------------------------------MSLTHETLRRDLEL-----STFGQRSRVLQLVKSLKAEYTKHSRY-SSAFSPSP-SYSSPFPAPSLLSS
BN1205_083850         VMEG------GKLPPDFIERCRKKALTGAEFMSLTHETLRRDLDL-----STFGQRSRVLQLVKSLKAEYTKHSRY-SSAFSPSP-SYSSPFPAPSLLSS
HHA_290225            AMEG------GRLPPDFIERCRKKTLTGAAFMRLTHETLRRDLEL-----STFGQRSRVLQLVKSLKVEYTKHSRY-SSGSSRLPSSSSSPFPPPSLLSS
ETH_00000110          SSEL----RSQKLPEGFIEALLERRVGGENFLSLNAARLKREFGM-----ATFGGRNRVLMFVKLLRLQASRRRR-------------------------
ENH_0020460           SSEL----RSQKLPEGFIEALLARRVGGESFLSLNAARLKREFGM-----ATFGGRNRVLMFVKLLRLQASRRRR-------------------------
EMWEY_00023200        ----------------------PEEAEGEEFLCLTAAQLKRDFGL-----ITFGGRNRVLMFVKLLRLQASRRRR-------------------------
Hs_RAF1               G---------EELQVDFLDHV----------PLTTHNFARKTFLK-----LAFCDICQKF-LLNGFRCQTCGYKF-------------------------
Pa_RAF1               G---------EELQVDFLDHV----------PLTTHNFARKTFLK-----LAFCDICQKF-LLNGFRCQTCGYKF-------------------------
Mm_RAF1               G---------EELQVDFLDHV----------PLTTHNFARKTFLK-----LAFCDICQKF-LLNGFRCQTCGYKF-------------------------
Rn_RAF1               G---------EELQVDFLDHV----------PLTTHNFARKTFLK-----LAFCDICQKF-LLNGFRCQTCGYKF-------------------------
Bt_RAF1               G---------EELQVDFLDHV----------PLTTHNFARKTFLK-----LAFCDICQKF-LLNGFRCQTCGYKF-------------------------
Gg_RAF1               G---------EELQVDFLDHV----------PLTTHNFARKTFLK-----LAFCDICQKF-LLNGFRCQTCGYKF-------------------------
Xl_RAF1               G---------AELQVDFLDHV----------PLTTHNFVRKTFLK-----LAFCDICQKF-LLNAFRCQTCGYKF-------------------------
Ce_RAF1               GNE-------LWVHSEYLNTV----------SSIKHAIVRRTFIP-----PKSCDVCNNPIWMMGFRCEFCQFKF-------------------------
GNI_162980            DPNFNTYPGSGSSAAKFVSTVEGTAAPTNGYGPIFANLDGKGGGISRLFPSRYSDTQDSGSQARGSQARGSQDRRS------------------------
```

```
                             810       820       830       840       850       860       870       880       890       900
                    ....|....|....|....|....|....|....|....|....|....|....|....|....|....|....|....|....|....|....|....|
PF3D7_1106800       ----------------------------------------------------------------------------------------------------
PFIT_1107600        ----------------------------------------------------------------------------------------------------
PPRFG01_1107500     ----------------------------------------------------------------------------------------------------
PRCDC_1105300       ----------------------------------------------------------------------------------------------------
PRG01_1104000       ----------------------------------------------------------------------------------------------------
PBILCG01_1104000    ----------------------------------------------------------------------------------------------------
PBLACG01_1104300    ----------------------------------------------------------------------------------------------------
PADL01_1105000      ----------------------------------------------------------------------------------------------------
PGABG01_1103900     ----------------------------------------------------------------------------------------------------
PGSY75_1106800      ----------------------------------------------------------------------------------------------------
PGAL8A_00334100     ----------------------------------------------------------------------------------------------------
PRELSG_0904000      ----------------------------------------------------------------------------------------------------
PocGH01_09013500    ----------------------------------------------------------------------------------------------------
PYYM_0941700        ----------------------------------------------------------------------------------------------------
PY02790             ----------------------------------------------------------------------------------------------------
PY02791             ----------------------------------------------------------------------------------------------------
PBANKA_0940100      ----------------------------------------------------------------------------------------------------
PCHAS_0904200       ----------------------------------------------------------------------------------------------------
PCHAS_090420        ----------------------------------------------------------------------------------------------------
YYE_02751           ----------------------------------------------------------------------------------------------------
YYG_01693           ----------------------------------------------------------------------------------------------------
PmUG01_09016400     ----------------------------------------------------------------------------------------------------
C922_04039          ----------------------------------------------------------------------------------------------------
PcyM_0908600        ----------------------------------------------------------------------------------------------------
PKNOH_S120122800    ----------------------------------------------------------------------------------------------------
PKNH_0904400        ----------------------------------------------------------------------------------------------------
PVP01_0907600       ----------------------------------------------------------------------------------------------------
PVX_091015          ----------------------------------------------------------------------------------------------------
THRCLA_22740        ----------------------------------------------------------------------------------------------------
THRCLA_04632        ----------------------------------------------------------------------------------------------------
BESB_019990         ----------------------------------------------------------------------------------------------------
BN1204_042460       VLPPSEFFLPEPRPALTLAHP---------------------HPAASLPA---------PRGQNPPT------ADL----------PSCP--PGVSICPS
TGGT1_290225        LLRPPGSAFP-SIPA--LAHPCPPSSAIASSPLVSCTRFPAAHPSSALCSQTRAPCVRGPSG-RLPSEFASSQASLREASRREEAGPRVPELPLASVSSS
TGDOM2_290225       LLRPPGSAFP-SIPA--LAHPCPPSSAIASSPLVSCTRFPAAHPSSALCSQTRAPCVRGPSG-RLPSEFASSQASLREASRREEAGPRVPEFPLASVSSS
BN1205_083850       LLRPPGSAFP-SIPA--LAHPCPPSSAIASSPLVSCTRFPAAHPSSALCSQTRAPCVRGPSG-RLPSEFASSQASLREASRREEAGPRVPELPLASVSSS
HHA_290225          LLRPPGSTLPSSIPA--LAHPCPPSSARASSPLLACALFADARPSASLCSQTLTPCTLGRRGERLPSEFASSCASLRETSSREETGPRVPELSLACVSSS
ETH_00000110        ----------------------------------------------------------------------------------------------------
ENH_0020460         ----------------------------------------------------------------------------------------------------
EMWEY_00023200      ----------------------------------------------------------------------------------------------------
Hs_RAF1             ----------------------------------------------------------------------------------------------------
Pa_RAF1             ----------------------------------------------------------------------------------------------------
Mm_RAF1             ----------------------------------------------------------------------------------------------------
Rn_RAF1             ----------------------------------------------------------------------------------------------------
Bt_RAF1             ----------------------------------------------------------------------------------------------------
Gg_RAF1             ----------------------------------------------------------------------------------------------------
Xl_RAF1             ----------------------------------------------------------------------------------------------------
Ce_RAF1             ----------------------------------------------------------------------------------------------------
GNI_162980          -------------------------------------------------------------G--------------------------------------

                             910       920       930       940       950       960       970       980       990      1000
                    ....|....|....|....|....|....|....|....|....|....|....|....|....|....|....|....|....|....|....|....|
PF3D7_1106800       ---------------NNLIQIEEYKNYIRQKENTNKNIKKGKNIK--KEKNI-----------------K---------KEKNI------KKEKKKKK--
PFIT_1107600        ---------------NNLIQIEEYKNYIRQKENTNKNIKKGKNIK--KGKNI-----------KKEKNKK---------KEKNI------KKEKKKKK--
PPRFG01_1107500     ---------------NNLIQIEEYKNYIRQKENTNKNIKKGKNIK--KGKNI-----------KKEKNKK---------KEKNIKKGKNKKKEKNKKK--
PRCDC_1105300       ---------------NNLIQIEEYKNYIRQKENTK----KKKNIK--KG-----------------------------------------KKKKRKKK--
PRG01_1104000       ---------------NNLIQIEEYKNYIRQKENTK----KKKNIK--KR-----------------------------------------KKKKTKKK--
PBILCG01_1104000    ---------------NNLIQIEEYKNYIRQKESTNKNI----------------------------------------------------KKGKKKKK--
PBLACG01_1104300    ---------------NNLIQIEEYKNYIRQKKNINKNK----------------------------------------------------KKGKKKKK--
PADL01_1105000      ---------------NNFIQIEEYKNYIRQKDNIT-------------------------------------------------------KKKKKKNK--
PGABG01_1103900     ---------------NNFIQIEEYKNYIRQKDNIT------------------------------------------------------KKKKKKKSK--
PGSY75_1106800      ---------------NNFIQIEEYKNYIRQKDNIT-------------------------------------------------------KKKKKKSK--
PGAL8A_00334100     ---------------LTCLNLEEYENHIKKKKNKNKIKNKLKNKI--NKQ----------------------------------MNNF-IVDNIFHKE--
PRELSG_0904000      ---------------LTCLNLEEYENHIRKKKKKKKKKKKKKKD--------------------------------------EKINNV-IDENTLQKE--
PocGH01_09013500    ---------------CKSLHLHQYERYLKREKGRR-IKGKRRKGK--ESGAKCGQ--------NWEEERKE-------ERKEERKEGKKEGKKEGKEE--
PYYM_0941700        ---------------ANTLNLEEYEIYLKKKMEKKKMKKKKMKEK--KMEKK------------KMEKKKM-------EKKMNEMNEMNEKKDGADTA--
PY02790             ---------------ANTLNLEEYEIYLKKKMEKKKMKKKKMKEK--KMEKK------------KMEKKKM-------EKKMNEMNEMNEKKDGADTA--
PY02791             ----------------------------------------------------------------------------------------------------
PBANKA_0940100      ---------------SNTLNLEEYEIYLKKKMKKKKMR---------------------------------------------------ENKDGEYTA--
PCHAS_0904200       ---------------ANTLNLEEYENYLEKKIKKKKIKDKKTREK--KTR---------------------------------------EKKGDADIE--
PCHAS_090420        ---------------ANTLNLEEYENYLEKKIKKKKIKDKKTREK--KTR---------------------------------------EKKGDADIE--
YYE_02751           ---------------ANTLNLEEYENYLKKKIKKKKKREKKKR----------------------------------------------EKKDDADIA--
YYG_01693           ---------------ANTLNLEEYENYLKKKIKKKKIKRKKKREK--KTR---------------------------------------EKKDDADII--
PmUG01_09016400     ---------------ARNLHLEEYAGYLKRVGKKQREKEQKKKEQ--RKK----------------EQRK--------------KEQKKKKQKGKEKK--
C922_04039          ----------------------------------------------------------------------------------------------------
PcyM_0908600        ---------------ARCVNIQECEHFLRR--KKKKKKKKKKIHT--RYGSR-------------GETRR---------GSDNRSQSRRHGETLHMFH--
PKNOH_S120122800    ---------------ARCVNIQE-------------------------HDSR-------------GVNRR---------RSENLSRSKSNGKEMNRFH--
PKNH_0904400        ---------------ARCVNIQECKHFLKRKKKKKKKKKKKKIHR--EHDSR-------------GVNRR---------RSENLSRSKSNGKEMNRFH--
PVP01_0907600       ---------------ARCVNIQECEHFLRRRKKKKKKIRTRCAP---------------------------------------------RGKKMHRFH--
PVX_091015          ---------------ARCVNIQECEHFLRRRKKKKKKIRTRCAP---------------------------------------------RGKKMHRFH--
THRCLA_22740        ----------------------------------------------------------------------------------------------------
```

```
THRCLA_04632        ----------------------------------------------------------------------------------------------------
BESB_019990         ----------------------AARRSLSSSPSRGTTSSSEYEGTAPNRDPALGDAC-----------------------------AGNDRRGSQD----
BN1204_042460       PF-PLFLANLAPHQGGEALRALAARRSVSSSPSGGTTSSGEDEIA--REPETEG---------ARAAARRCVAASIFGPETQAWAPARREEEG-------
TGGT1_290225        PSGQVALERLALDQGGEALRALAARRSFSSSLSGGTTSNEEETGK--ETGDAAGREAGNEERGERANSRRALLGRDA--DSRLFLEQRRRGEG-------
TGDOM2_290225       PSGQVALERLALDQGGEALRALAARRSFSSSLSGGTTSNEEETGK--ETGDAAGREAGNEERGERANSRRALLGRDA--DSRLFLEQRRRGEG-------
BN1205_083850       PSGQVALERLALDQGGEALRALAARRSFSSSLSGGTTSNEEETGK--ETGDAAGREAGNEERGERANSRRALLGRDA--DSRLFLEQRRRGEG-------
HHA_290225          PSGQAALQRLAPDQGGEALRALAARRSFSSSLSGGTTSNEEETGK--ETGDAAGRQAGNEER-ERTNSRRTLLCRDA-EDTRFFLQQGRRGEGTKEEEE-
ETH_00000110        ----------------------VHRLFLSSSPSSPSVDS-------------------------------------------------------------
ENH_0020460         ----------------------VHRLFLSSSPSSPSVDS-------------------------------------------------------------
EMWEY_00023200      ----------------------IHRLFLSSS---------------------------------------------------------------------
Hs_RAF1             -----------------------HEHCSTKVPTMC-----------------------------------------------------------------
Pa_RAF1             -----------------------HEHCSTKVPTMC-----------------------------------------------------------------
Mm_RAF1             -----------------------HEHCSTKVPTMC-----------------------------------------------------------------
Rn_RAF1             -----------------------HEHCSTKVPTMC-----------------------------------------------------------------
Bt_RAF1             -----------------------HEHCSTKVPTMC-----------------------------------------------------------------
Gg_RAF1             -----------------------HEHCSTKVPTMC-----------------------------------------------------------------
Xl_RAF1             -----------------------HEHCSTKVPTMC-----------------------------------------------------------------
Ce_RAF1             -----------------------HQRCSSFAPLYCDLLQSV-----------------------------------------------------------
GNI_162980          --------------QESWGSGEEDRGTQKREPGAEDRGRDSVSQDLPSQGDQAGSSAFGRFSQNAQDGRK--------------IPPGRERRGVFRSQNF

                          1010      1020      1030      1040      1050      1060      1070      1080      1090      1100
                    ....|....|....|....|....|....|....|....|....|....|....|....|....|....|....|....|....|....|....|....|
PF3D7_1106800       -----KETKKFNNMDK-------------------------------------------------------KYIDLAIHKNVK----------------N
PFIT_1107600        -----KETKKFNNMDK-------------------------------------------------------KYIDLAIHKNVK----------------N
PPRFG01_1107500     -----KETKKFNNMDK-------------------------------------------------------KYIDLAIHKNVK----------------N
PRCDC_1105300       -----KKKKKKKKKKKEKK------------------------------------KTNK------KIQQHGKYIHLAIHKNVK----------------N
PRG01_1104000       -----KQTKKFNNMDK-------------------------------------------------------KYIHLAIHKNVK----------------N
PBILCG01_1104000    -----KERKKLNNIDK-------------------------------------------------------KYIDLAIHKNVN----------------N
PBLACG01_1104300    -----KDRKILNNMDK-------------------------------------------------------KYIDLAIHKNVK----------------N
PADL01_1105000      -----E-TKKSNNMDK-------------------------------------------------------KYIDLAIHKNVK----------------N
PGABG01_1103900     -----EKKKKSNNMHK-------------------------------------------------------KYIHLAIHENVK----------------N
PGSY75_1106800      -----EKKKKSNNMHK-------------------------------------------------------KYIHLAIHENVK----------------N
PGAL8A_00334100     -----NDKPEINNASKNSN------------------------------------SLLE--------NLNKEIEMENKHINSK----------DALFLDG
PRELSG_0904000      -----NNSTEINSENKNSK------------------------------------FLLE--------HLNEGIEMENKHLHCK----------DVLISEN
PocGH01_09013500    -----ERGTKWEDWWGDIR------------------------------------KEER--------RKNGKEEREDKQKGQKKVSPFIKGTPDAKFSQR
PYYM_0941700        -----TTSSQAEDFQKNKK------------------------------------KI-----------MSNQTKSMIDQKNDD----------NKSFSDK
PY02790             -----TTSSQAEDFQKNKK------------------------------------KI-----------MSNQTKSMIDQKNDD----------NKSFSDK
PY02791             ----------------------------------------------------------------------------------------------------
PBANKA_0940100      -----TTSSQAEDFQKNNQ------------------------------------KI-----------IMNQIKSMIDQKNDD----------DKSFSDK
PCHAS_0904200       -----TSSSQIDIFQKDDQ------------------------------------KII----------MNKQIKSMINQKNVN----------DKSFSDK
PCHAS_090420        -----TSSSQIDIFQKDDQ------------------------------------KII----------MNKQIKSMINQKNVN----------DKSFSDK
YYE_02751           -----MSSSQIDIFQKDDQ------------------------------------KII----------MNKQIKSMINQKNVN----------DKSFSDK
YYG_01693           -----MSSSQIDIFQKDDQ------------------------------------KII----------MNKQIKSMINQKNVN----------DKSFSDK
PmUG01_09016400     -----EEEQKEEENQRGEN------------------------------------KIH----------PTAVTNQSASEQNLE----------DAQVNNS
C922_04039          ----------SHQTHQSKH------------------------------------KLTLGEPPTSSAPPEKEAEAVFTKKMLHN--------GNAINQEK
PcyM_0908600        -----PSPDSCQQIHQGKH------------------------------------KLTLGEPPTSSAPPEEEEAAVFTKKMLHS--------GDAVCHEE
PKNOH_S120122800    -----PPPNSSQ---QSKH------------------------------------MLTLGEPITSDAPPEEE-VPVLTKKILYN--------GDAVYQEE
PKNH_0904400        -----PPPNSSQ---QSKH------------------------------------MLTLGEPITSDAPPEEE-VPVLTKKILYN--------GDAVYQEE
PVP01_0907600       -----PPPSEIQPIKQSKHIQHIQQIQPIQPIQPIQPIQPIQPIQSIQPIQPIQGKLPLGEPPTSRAPPEEE-AAVLTEKMLRS--------GGALYQEE
PVX_091015          -----PPPSEIQPIKQSKH---IQQIQPIQPIQSIQSIQP------IQSIQPIQGKLPLGEPPTSRAPPEEE-AAVLTEKMLRS--------GGALYQEE
THRCLA_22740        ----------------------------------------------------------------------------------------------------
THRCLA_04632        ----------------------------------------------------------------------------------------------------
BESB_019990         -----ASEASTHSPASAKS-----------------------------------PHVGAGARQ----PRGRRRVRVRQKGEGG---------A-------
BN1204_042460       -----RGESAFEKLRRPTA---------------------------------------AGART--CTECGGDFSRQDRDKEGAF--------G----GDE
TGGT1_290225        -----PREEEWTETESGAS------------------------------------RISGGACD-ACTPREQDRLKLEVDSECH----------SKKDGCE
TGDOM2_290225       -----PREEEWTETESSAS------------------------------------RISGGACD-ACTPREQDRLKLEVDSECH----------CKKDGCE
BN1205_083850       -----PREEEWTETESSAS------------------------------------RISGGACD-ACTPREQDRLKLEVDSECH----------SKKDGCE
HHA_290225          --KENEKEEEWTGTERSAS------------------------------------GVSSGARD-ACRPREQDLLKFQVDSERH----------GKKDGHE
ETH_00000110        ----------------------------------------------------------------------------------------------------
ENH_0020460         ----------------------------------------------------------------------------------------------------
EMWEY_00023200      ----------------------------------------------------------------------------------------------------
Hs_RAF1             ----------------------------------------------------------------------------------------------------
Pa_RAF1             ----------------------------------------------------------------------------------------------------
Mm_RAF1             ----------------------------------------------------------------------------------------------------
Rn_RAF1             ----------------------------------------------------------------------------------------------------
Bt_RAF1             ----------------------------------------------------------------------------------------------------
Gg_RAF1             ----------------------------------------------------------------------------------------------------
Xl_RAF1             ----------------------------------------------------------------------------------------------------
Ce_RAF1             -------------------------------------------------------------------PKNEDLVKEL-----------------------
GNI_162980          LCYTRMSQSEGDSLEPGGV-----------------------------------SPARAGAHTCAGHACSGQGSSLSVQQCAT----------AAGTTTA

                          1110      1120      1130      1140      1150      1160      1170      1180      1190      1200
                    ....|....|....|....|....|....|....|....|....|....|....|....|....|....|....|....|....|....|....|
PF3D7_1106800       IQNDTF---------YNKHENI---YNC------------------------------KNQTNFIYQNDSEI-----------------------KKIMN
PFIT_1107600        IQNDTF---------YNKHENI---YNC------------------------------KNQTNFIYQNDSEI-----------------------KKIMN
PPRFG01_1107500     IQNDTF---------YNKHENI---YNC------------------------------KNQTNFIYQYDSEI-----------------------KNIMN
PRCDC_1105300       IQNDTF---------YNKDENI---YNC------------------------------KNQTNFIHQNYSEI-----------------------KKIVN
PRG01_1104000       IQNDTF---------YNKDENI---YNC------------------------------KNQTNFIHQNYSEI-----------------------KKIVN
PBILCG01_1104000    IQDDTF---------YNKDENI---YNC------------------------------KYQKNFSHQNDSEK-----------------------KNLMN
PBLACG01_1104300    IQDDTF---------YNKDEHI---YNC------------------------------KNQKYYIHQNDSEK-----------------------KYTMN
PADL01_1105000      IKNDTF---------QYNDDNI---NNC------------------------------KNQKNYIHQNDYEN-----------------------KNIMN
PGABG01_1103900     IKNDTF---------QYNNDNI---NNC------------------------------KNQQNYIQQNDYEN-----------------------INIMN
```

```
PGSY75_1106800      IKNDTF---------QYNNDNI---NNC------------------------------KNQQNYIQQNDYEN-----------------------INIMN
PGAL8A_00334100     INSFTY----------VSSDSM---SKS--------------NKIENCAPE-------INLDNTTINYIKEA--------------------NIHTKKKK
PRELSG_0904000      LNSFTC----------VSSNSI---RKP--------------NKIENGASE-------INLDNPTLNYIKET-------------------NTYAKKKKK
PocGH01_09013500    KNRKSR----------EKNSST---ASS---GVSGHGGFFLRRKLLRGAGDQ----YPSNSADTCTSTSHNP----------G--------QDIQKERTD
PYYM_0941700        IKNGFF----------KGNTNI---KNL------------------------------QNLQNLVINSIWN-----------------------------
PY02790             IKNGFF----------KGNTNI---KNL------------------------------QNLQNLVINSIWN-----------------------------
PY02791             ----------------------------------------------------------------------------------------------------
PBANKA_0940100      IKNGFF----------KGNTNI------------------------------------RNLQNLVINSIWN-----------------------------
PCHAS_0904200       IKNGFL----------QGNTNI------------------------------------KNLQNFVINTIWD-----------------------------
PCHAS_090420        IKNGFL----------QGNTNI------------------------------------KNLQNFVINTIWD-----------------------------
YYE_02751           IKNGFL----------QGNTNI------------------------------------KNLQNFVINTIWD-----------------------------
YYG_01693           IKNGFL----------QGNTNI------------------------------------KNLQNFVINTIWD-----------------------------
PmUG01_09016400     INRDTIPPMEKNIINDKKKTST---SKPINSCIST----MPYNCVDKSQGKHGHTIKFIEDKSTIYRCIENA----------GI---RNEENGWAGKTTD
C922_04039          DPNSCT-----STTSTTSTTSF---NSP------------------------------RTLKEEAYGKREEL----------G-------EEDAEEEHHH
PcyM_0908600        DPNSC-----------TSTTSF---NST------------------------------RTLKEEDYGKREEL----------G-------EEDAEEEHHH
PKNOH_S120122800    DPNSC-----------TSTTSF---NST------------------------------RTLKEEVYGKMEKL----------G-------EQHAEEQHHL
PKNH_0904400        DPNSC-----------TSTTSF---NST------------------------------RTLKEEVYGKMEKL----------G-------EQHAEEQHHL
PVP01_0907600       DPDSC-----------TSTTSF---NST------------------------------HTLKE------EEV----------G-------EEGAQEGHHQ
PVX_091015          DPDSC-----------TSTTSF---NST------------------------------HTLKE------EEV----------G-------EEGAQEGHHQ
THRCLA_22740        ----------------------------------------------------------------------------------------------------
THRCLA_04632        ----------------------------------------------------------------------------------------------------
BESB_019990         ----------------DSASPCFLQLSP---------------------SP-------PFLS--------------------G-----EPAEVAAPPR-D
BN1204_042460       GRVPSL----------VGAAQV---PGP------------------------------PGLRNE-----------------RG-------GRHGRGEEAR
TGGT1_290225        AKALSS----------RGYEEVFLVESP--------------------HAPQ------PGTNNEGNTENQEVIAEAA-SCRRGMEAVLPPGEESETKRVR
TGDOM2_290225       AKALSS----------RGYEEVFLVESP--------------------HAPQ------PGTNNEGNTENQEVIAEAA-SCRRGMEAVLPPGEESETKRVR
BN1205_083850       AKALSS----------RGYEEVFLVESP--------------------HAPQ------PGTNNEGNTENQEVIAEAA-SCRRGMEAVLPPGEESETKRVR
HHA_290225          AKASSF----------RGYEEVSL-KSP--------------------HAPQ------PETHNGENTENQEVIATASFSCRQGTEAVLPPGEESETKHVR
ETH_00000110        ----------------QAAYSL--RGSP------------------------------------------------------G-----------------
ENH_0020460         ----------------QTAYSL--RGSP------------------------------------------------------G-----------------
EMWEY_00023200      ----------------------------------------------------------------------------------G-----------------
Hs_RAF1             ----------------VDWSNI------------------------------------------------------------------------------
Pa_RAF1             ----------------VDWSNI------------------------------------------------------------------------------
Mm_RAF1             ----------------VDWSNI------------------------------------------------------------------------------
Rn_RAF1             ----------------VDWSNI------------------------------------------------------------------------------
Bt_RAF1             ----------------VDWSNI------------------------------------------------------------------------------
Gg_RAF1             ----------------VDWSNI------------------------------------------------------------------------------
Xl_RAF1             ----------------VDWSNI------------------------------------------------------------------------------
Ce_RAF1             -----F----------GIASQV---EGP------------------------------------------------------------------------
GNI_162980          AGATTA----------ADATTV---AGA------------------------------TTAAGATTAAGATTA--------AGV------TTSAAVCPCQ

                          1210      1220      1230      1240      1250      1260      1270      1280      1290      1300
                    ....|....|....|....|....|....|....|....|....|....|....|....|....|....|....|....|....|....|....|....|
PF3D7_1106800       KKKVSF----------------------------------------------------------------------------------------EYDN--
PFIT_1107600        KKKVSF----------------------------------------------------------------------------------------EYDN--
PPRFG01_1107500     KKKVSF----------------------------------------------------------------------------------------EYDN--
PRCDC_1105300       KKKVSF----------------------------------------------------------------------------------------EHDN--
PRG01_1104000       KKKVSF----------------------------------------------------------------------------------------EHDN--
PBILCG01_1104000    KKKVSF----------------------------------------------------------------------------------------EHDN--
PBLACG01_1104300    KKKVSF----------------------------------------------------------------------------------------QHDN--
PADL01_1105000      KKNLSF----------------------------------------------------------------------------------------EYDN--
PGABG01_1103900     KKNLSF----------------------------------------------------------------------------------------KYDN--
PGSY75_1106800      KKNLSF----------------------------------------------------------------------------------------KYDN--
PGAL8A_00334100     KKK------------------------KKKIR-------------------KKKGE--------------------------------------QINE--
PRELSG_0904000      KKKKE----------------------KKKMRENEE---------------KEKEE--------------------------------------NKKE--
PocGH01_09013500    ERKDSFD--------CQT-----VYTTREKIRAQSA--------------EDEKEGSI-----------------------------------CHLRE--
PYYM_0941700        KSKDEF---------------------KIPINENNIP------------MKIEKGK--------------------------------------ELNT--
PY02790             KSKDEF---------------------KIPINENNIP------------MKIEKGK--------------------------------------ELNT--
PY02791             ----------------------------------------------------------------------------------------------------
PBANKA_0940100      KSKDEF---------------------KIPIDENNVP------------MNIEKGK--------------------------------------ELNT--
PCHAS_0904200       KNKDEF---------------------EKPIEENNIP------------IHVEKEK--------------------------------------ELNT--
PCHAS_090420        KNKDEF---------------------EKPIEENNIP------------IHVEKEK--------------------------------------ELNT--
YYE_02751           KNKDEF---------------------EKPIEENNSP------------MHIEKEK--------------------------------------ELNT--
YYG_01693           KNKDEF---------------------EKPIEENNSS------------MHAEKEK--------------------------------------ELNT--
PmUG01_09016400     QSKEEVEE-GT--DKCRTNGSIEVSLIKKKKKEEEKDDVDRARKGE-TTNHVEKTGKKGLNNHVDGTGQMELNNHVDGTGQMELNNHVDGTGQMELNNHV
C922_04039          QQ--------------QK---------RHHVGETSAP--------------NEPPEPQ------------------------------------TPND--
PcyM_0908600        HQQQQR--------RKQK---------RQHVGETSAP--------------NEPVGPP------------------------------------TPSD--
PKNOH_S120122800    QE---------------------------RVCDRSAP--------------NEPVCHP------------------------------------TSND--
PKNH_0904400        QE---------------------------RVCDRSAP--------------NEPVCHP------------------------------------TSND--
PVP01_0907600       KR--------------QK---------WQHAEGSSAP--------------NEPQEPL------------------------------------RPND--
PVX_091015          KR--------------QK---------WQHAEGSSAP--------------NEPQEPL------------------------------------RPND--
THRCLA_22740        ----------------------------------------------------------------------------------------------------
THRCLA_04632        ----------------------------------------------------------------------------------------------------
BESB_019990         APSSRLSA-------CASAG-------------------------------------------------------------------------LAPAS--
BN1204_042460       LPQSHASS------CCDTSQ---------------------------LFRGTGGAVP------------------AGPLGPRL----------ALPSP--
TGGT1_290225        PPRPHLAL-------CHSSSS----SSRQTFERHPQADPKHLKQPPSFFTASSSPSPSP-------------PRHSSPSPPSPSPPRHSPSSPRSPSP--
TGDOM2_290225       PPRPHLAL-------CHSSSS----SSRQTFERHPQADPKHLKQPPSFFTASSSPSPSP-------------PRHSSPSPP-------------SPSP--
BN1205_083850       PPRPHLAL-------CHSSSS----SSRQTFERHPQADPKHLKQPPSFFTASSSPSPSP-------------PRHSSPSPPSPSPPRHSPSSPRSPSP--
HHA_290225          PPRPHLAL-------CHPSSS----SSRQAFERHEQADPGHLRQPPSFFTASSSPSP--------------------------------------PSP--
ETH_00000110        ----------------------------------------------------------------------------------------------APPL--
ENH_0020460         ----------------------------------------------------------------------------------------------APPL--
EMWEY_00023200      ----------------------------------------------------------------------------------------------------
Hs_RAF1             ---RQL---------------------------------------------------L-----------------------------------LFPNS--
```

```
Pa_RAF1             ---RQL---------------------------------------------------L-----------------------------------LFPNS--
Mm_RAF1             ---RQL---------------------------------------------------L-----------------------------------LFPNS--
Rn_RAF1             ---RQL---------------------------------------------------L-----------------------------------LFPNS--
Bt_RAF1             ---RQL---------------------------------------------------L-----------------------------------LFPNS--
Gg_RAF1             ---RQL---------------------------------------------------L-----------------------------------LFPNS--
Xl_RAF1             ---RQL---------------------------------------------------L-----------------------------------LFPNP--
Ce_RAF1             --DRSVAE---------------------IVLANLAP--------------TSGQSPA-----------------------------------ATPDS--
GNI_162980          MDAKSFSKWLKSFRPCSR-----------EVVAYMAARKWTWRD-----MERALAEPRG----------------------------------RRPAT--

                             1310      1320      1330      1340      1350      1360      1370      1380      1390      1400
                    ....|....|....|....|....|....|....|....|....|....|....|....|....|....|....|....|....|....|....|....|
PF3D7_1106800       -------------------------NEEKKKK------NIIKFIKN-N--KSLQNSNGEYYLINHLSKGICSDSIFYKSSQSKSS--------------S
PFIT_1107600        -------------------------NEEKKKK------NIIKFIKN-N--KSLQNSNGEYYLINHLSKGICSDSIFYKSSQSKSS--------------S
PPRFG01_1107500     -------------------------NEEKKKK------NIIKFIKN-N--KSLQNSNGEYYLINDLSKGICSDSIFYKSSQSS----------------S
PRCDC_1105300       -------------------------NEEKKKI------NIMKFIKN-N--KSFQNSNGEYYLINDLSNGICSDSIFYKSLQSKSSSPSSSPSSPSSPSPS
PRG01_1104000       -------------------------NEEKKKI------NIMKFIKN-N--KSFQNSNGEYYLINDLSNGICSDSIFYKSLQSKSSSPSS----------S
PBILCG01_1104000    -------------------------N-EQKKK------KIIKFKK--N--KSFQNSNGEYYLINDLSKGICSDRIFYKSSPSHSTPP------------S
PBLACG01_1104300    -------------------------NGKKKKK------KIIKFKK--N--KPLKNSNVEYYLINDLSKGICTDSIFSNSSSSSKSLSPS-----KSQSPS
PADL01_1105000      -------------------------N--EKKK------KIIKCKK--N--KSLESSNDQYDLINDLSKDICSDSIFYNS---------------------
PGABG01_1103900     -------------------------N--EKKK------KIIKCKK--N--KSLENSNGHYDLINDLSKDICSDSIFYNSS--------------------
PGSY75_1106800      -------------------------N--EKKK------KIIKCKK--N--KSLENSNCQYDLINDLSKDICSDSIFYNSS--------------------
PGAL8A_00334100     -------------------------KENKNEK------KEDEIEN--E--NFIFYFQEELSSKNNSS---------NFSN--------------------
PRELSG_0904000      -------------------------KENKNEE------KKKKIDK--E--KFIYNFHEKLSLINYSS---------YDSN--------------------
PocGH01_09013500    -------------------------GENKQRG------KEKRTKK--E--KFIRNFHQKFSLIENLS---------DTPSG-------------------
PYYM_0941700        -------------------------NDQSTGK------KKKKLKK--N--KYINHVQKNISMLKKLH---------NTSNE-------------------
PY02790             -------------------------NDQSTGK------KKKKLKK--N--KYINHVQKNISMLKKLH---------NTSNE-------------------
PY02791             ----------------------------------------------------------------------------------------------------
PBANKA_0940100      -------------------------NDQITGK------KKKKLKN-----KYINHIQKNISMLKKLN---------NASNE-------------------
PCHAS_0904200       -------------------------NEDSAEK------EVTELKK--D--KHTNHVQQNISMLKNMH---------TESNE-------------------
PCHAS_090420        -------------------------NEDSAEK------EVTELKK--D--KHTNHVQQNISMLKNMH---------TESNE-------------------
YYE_02751           -------------------------NEDSTKK------VVTELKK--D--KYTN-VQKRISMLKNVD---------SEYNE-------------------
YYG_01693           -------------------------NEDNVEK------KITELKK--D--KYTNHVQKKISMLKNMH---------SESNE-------------------
PmUG01_09016400     DGTNESETNNRLGGIDQRETNTEDIKDEKRKK------SKREIKK--EKRKLLYNFHEKYTLINDII---------NTSNENLYV---------------
C922_04039          ----------------------TQKDTNKKRR------KDKKKQK--E--QFIRSFHKNFSLINDFG---------DTFNG-------------------
PcyM_0908600        ----------------------PQKDTNKQKRKDKKNQKDKKKQK--E--QFIRSFHKNFSLINDFG---------HTFNG-------------------
PKNOH_S120122800    ----------------------RQKDTNKQRR------EDKKEKK--K--KFIRSFHKNFSLINDFG---------NTING-------------------
PKNH_0904400        ----------------------RQKDTNKQRR------EDKKEKK--K--KFIRSFHKNFSLINDFG---------NTING-------------------
PVP01_0907600       ----------------------PHINTDRKKR------KKKKKKK--E--QFIRSFHEKFSLINDFA---------HTFNG-------------------
PVX_091015          ----------------------PHINTDRKKR------KKKKKKK--E--QFIRSFHEKFSLINDFA---------HTFNG-------------------
THRCLA_22740        ----------------------------------------------------------------------------------------------------
THRCLA_04632        ----------------------------------------------------------------------------------------------------
BESB_019990         ---------------------PPWGGHSTRGE----GGARRKEPV-----------GREAAARSSLS-------SGSAPQS-------------------
BN1204_042460       ---------------------SSEASLASRAR----DGAETQAPR--DC-GSPFAACSPPSLASTRS-------PFLASSS-----------------VG
TGGT1_290225        ---------------------PRHSSPFPRSS------SPSSPPRHLSCPSPPSSPSSPSSLHSPPS-------SFSYLSSALSS--------------S
TGDOM2_290225       ---------------------PRHSSPCPRSP------SPSSPPRHLSCPSPPSSPSSPSSLHSPPS-------SFSYLSSALSS--------------S
BN1205_083850       ---------------------PRHSSPSPRSS------SPSSPPRHLSCPSPPSSPSSPSSLHSPPS-------SFSYLSSALSS--------------S
HHA_290225          -------------------------SFSPRS-------SPSSPPR------SSSSPSSPSSLPSPPS-------SFSSPSSALSS--------------S
ETH_00000110        ------------------------------------IGAQAPSPY--QGTSLLGTPGCGAATRTSTS-------VFSETDG-------------------
ENH_0020460         ------------------------------------IGAHAPSPY--QGPSLLGAPGCGAATRTSTS-------VFSETDG-------------------
EMWEY_00023200      -----------------------------------------------------------------AS-------VLPETST-------------------
Hs_RAF1             --------------------TIGDSGVPAL-------------PS-----------LTMRRMRESVS-------RMPVSSQ------------------H
Pa_RAF1             --------------------TIGDSGVPAL-------------PS-----------LTMRRMRESVS-------RMPVSSQ------------------H
Mm_RAF1             --------------------TVGDSGVPAP-------------PS-----------FPMRRMRESVS-------RMPASSQ------------------H
Rn_RAF1             --------------------TASDSGVPAP-------------PS-----------FTMRRMRESVS-------RMPASSQ------------------H
Bt_RAF1             --------------------TVGDGGVPAL-------------PS-----------LTMRRMRESVS-------RIPPGSQ------------------H
Gg_RAF1             --------------------NISDSGVPAL-------------PP-----------LTMRRMRESVS-------RIPVSSQ------------------H
Xl_RAF1             --------------------NNIEGGSHTL-------------PS-----------LTMRRIGESV--------RIPVSSQ------------------Q
Ce_RAF1             ----------------------SHPDLTSIKR----TGGVKRHPM-------------------AVS-------PQNETSQ-------------------
GNI_162980          --------------------NARRRDPPRKKQ--VVFRASSKEPN--S--KDKSNKDRASDLVASGSKE-ASGQALAPVSSAMKA---------------

                             1410      1420      1430      1440      1450      1460      1470      1480      1490      1500
                    ....|....|....|....|....|....|....|....|....|....|....|....|....|....|....|....|....|....|....|....|
PF3D7_1106800       QLSSPLP----SSSPSSSPSSSPS--SSPSSSPSPSSSPSPSS----SPSS--------------SPSSSP-SSPPSPLSYKDNFPISS-SCSSLERLP-
PFIT_1107600        QLSSPL----------SSPLSSPS--SSPSSSPSPSSSP--------------------------SPSSSP-SSPPSPLSYKDNFPISS-SCSSLERLP-
PPRFG01_1107500     QLSSSSP------SPLSSPSSSPSQLSSPSSSSSPSPSPSPSS----SP----------------SPSSSP-SSPSSPLSYKDNFPISS-SCSSLERLP-
PRCDC_1105300       PSSSPSP----SSSPSPSPSSPPS--PSPSSSPSPSSSPSPSSSSPPSPSSSSPPSPSPSPSPSSSPSPSP-PSPSPPLSYKDNFSSSS-SCSSLERLP-
PRG01_1104000       QSKSSSP----SSPPSPSPSSPPS--PSPSSS--PSSSPSPSSSSPPSPSSSSPP----------SPSPSP-PSPSPPLSYKDNFSSSS-SCSSLERLP-
PBILCG01_1104000    QSSSPSQ------SSSPSQSSSPSQSSSPSQSSSPSQSSSPSQSS--SPSQSSSP----------SQSSSL-PSPSFPLSYKDNLSILS-SCSSLERLP-
PBLACG01_1104300    QLLSPSKPLPSSHPSSPSPSSHPS-SPSPSSSSSPSSSSSPSSSSP-SPSQSSSP----------SPSQSS-SSPSSSLSYKDSLPISS-SCSSLERLP-
PADL01_1105000      ------------------------------------------------------------------------SSLSSPLSYSNSFSDSS-SCSSLERLP-
PGABG01_1103900     -------------------------------------------------------------------SQTT-SSLSSPLSFNNSFSDSS-SCSSLERLP-
PGSY75_1106800      -------------------------------------------------------------------SQTT-SSLSSPLSFNNNFSDSS-SCSSLERLP-
PGAL8A_00334100     ------------------------------------------------------------------KSSSF-S-TSSTLSSFTSSTISS-SNSSISTHI-
PRELSG_0904000      ------------------------------------------------------------------KNSSY-S-CSSTLSSFTSSTVTS-SDSSIYSHI-
PocGH01_09013500    -----------------------------------------------------------------SVTCAY-SEVGEESATPSLSSFTA-TSSNLST---
PYYM_0941700        -----------------------------------------------------------------SQSSNE-S-ITQTLSTSSESSFTN-IHSDLSSKIL
PY02790             -----------------------------------------------------------------SQSSNE-S-ITQTLSTSSESSFTN-IHSDLSSKIL
PY02791             ----------------------------------------------------------------------------------------------------
PBANKA_0940100      -----------------------------------------------------------------SQSSNE-S-VTQILSTSSESSFAN-IHSGLSSKML
PCHAS_0904200       -----------------------------------------------------------------SESSTT-S-ITQTLSTSSDSTFTN-IHSDESSKI-
PCHAS_090420        -----------------------------------------------------------------SESSTT-S-ITQTLSTSSDSTFTN-IHSDESSKI-
YYE_02751           -----------------------------------------------------------------SESSNT-S-ITETLSTSSDSSFTN-IHSDESSKG-
```

```
YYG_01693             -----------------------------------------------------------------SESSNT-S-ITQTLSTSSDSSFTN-IHSDESSKI-
PmUG01_09016400       -----------------------------------------------------------------SDSALE-VSVTSSLSSFSYTTTTS-GSLTLSES--
C922_04039            -----------------------------------------------------------------GSSRAYSGAEGKNLAVSDAPKEVPYGSCSLSSFSV
PcyM_0908600          -----------------------------------------------------------------GNSKDY-SRAGEKRAVSGDPKDDSYGSCSLSSFSI
PKNOH_S120122800      -----------------------------------------------------------------GISRIY-SGEGENRALSGDPKDDDYGSCSLSSFSI
PKNH_0904400          -----------------------------------------------------------------GISRIY-SGEGENRALSGDPKDDDYGSCSLSSFSI
PVP01_0907600         -----------------------------------------------------------------GSSRAY-SGEGANRAAPGDPKDEAYGSCSLSSFSI
PVX_091015            -----------------------------------------------------------------GSSRAY-SGEGANRAAPGDPKDEAYGSCSLSSFSI
THRCLA_22740          ----------------------------------------------------------------------------------------------------
THRCLA_04632          ----------------------------------------------------------------------------------------------------
BESB_019990           RRATPKG--KCPLCRRPL-----------------------------------------------RPPAPV-S-----------------SAAPLSGLD-
BN1204_042460         TRLRPRH--ACSLCRRSLHAVSFSETLSPG---TPSC---------------------------SSPSASVATGQGCSSPHGRPASHS--AGSDGEGA--
TGGT1_290225          VRLRPRQ--VCSMCERPLHASEPGRFTSAA---APA----------------------------RSPPASV-SALGGPSPPGEFSASL--PRDSCDGV--
TGDOM2_290225         VRLRPRQ--VCSMCERPLHASEPGRFTSPA---APA----------------------------RSPPASV-SALGGPSPPGEFSAFL--PRDSCDGV--
BN1205_083850         VRLRPRQ--VCSMCERPLHASEPGRFTSPA---APA----------------------------RSPPASV-SALGGPSPPGEFSAFL--PRDSCDGV--
HHA_290225            VRRRPRQ--VCLMCERPLHASEPGRFSSPA---TPA----------------------------RSPPASV-SGLGESSLPGEFSAFP--SRGSCDGV--
ETH_00000110          ---------------------------FPG---APRGLS--------------------------YPTTPP-------------------EGPSMGASR-
ENH_0020460           ---------------------------LPG---APRGFS--------------------------YPTTPP-------------------EGPSIGASR-
EMWEY_00023200        ---------------------------SPG---GPHG----------------------------GPQERY-S-----------------SSGTLEG---
Hs_RAF1               ---------------------------RYS---TPHAFT-------------------------FNTSSPS-------------------SEGSL-----
Pa_RAF1               ---------------------------RYS---TPHAFT-------------------------FNTSSPS-------------------SEGSL-----
Mm_RAF1               ---------------------------RYS---TPHAFT-------------------------FNTSSPS-------------------SEGSL-----
Rn_RAF1               ---------------------------RYS---TPHAFT-------------------------FNTSSPS-------------------SEGSL-----
Bt_RAF1               ---------------------------RYS---TPHAFT-------------------------FSASSPS-------------------SEGSL-----
Gg_RAF1               ---------------------------RYS---TPHVFT-------------------------FNTSNPS-------------------SEGTL-----
Xl_RAF1               ---------------------------RYS---TPHPFS-------------------------FSTPSPV-------------------SECSL-----
Ce_RAF1               --------------------------LSPS---GPYPRD-------------------------RSSSAPNINAINDEATVQHNQR----ILDALEAQRL
GNI_162980            ---------VGNLLRVVLSAGLPS--GLPKRVNFPTGLP--------------------------RHKSPV-SSLSYDSRVVNFTAASRAATSSADSGR-

                               1510      1520      1530      1540      1550      1560      1570      1580      1590      1600
                      ....|....|....|....|....|....|....|....|....|....|....|....|....|....|....|....|....|....|....|....|
PF3D7_1106800         SYEKKLLSSSQSNIEHIK------NLPLDVLS----NNNSSA-------NIKIKK------------SKSKYNNDKKEQ--K-KLPL-------------
PFIT_1107600          SYEKKLLSSSQSNIEHIK------NLPLDVLS----NNNSSA-------NIKIKK------------SKSKYNNDKKEQ--K-KLPL-------------
PPRFG01_1107500       SYEKKLLSSSQSNIEHIK------NLPLDVLS----NNNSSA-------NIKIKK------------SKSKYNNDKKEQ--K-KLPL-------------
PRCDC_1105300         SYEKKLLSSSQSNIEHIK------NLPLDVLS----NNNNSA-------NIKIKK------------SKSKYNNDKKEQ--K-EFPL-------------
PRG01_1104000         SYEKKLLSSSQSNIEHIK------NLPLDVLS----NNNNSA-------NIKIKK------------SKSKYNNDKKEQ--K-EFPL-------------
PBILCG01_1104000      SYEKKLLSSSQSNIEHIK------NLPLDTLS----NNNNSE-------NIKIKR------------SKSKYINGKKEQ--K-KLSF-------------
PBLACG01_1104300      SYEKKLLSLSHSNIEHIK------NLPLDVLS----NNNNSA-------NIKIKR------------SKSKYKNDKKKQ--K-KLSF-------------
PADL01_1105000        SYEKKLLSSSQSNIEHIK------NLPLDVLS----NNNNSE-------NIKIKR------------SKSKYNNDKKKQ--K-QFSF-------------
PGABG01_1103900       SYEKKLLSSSQSNIEHIK------NLPLDILS---NNNNNSE-------NIKIKR------------SKSKYNNDKKKKTNK-QFSF-------------
PGSY75_1106800        SYEKKLLSSSQSNIEHIK------NLPLDILS-NNNNNNNSE-------NIKIKR------------SKSKYNNDKKKKKKK-QFSF-------------
PGAL8A_00334100       --SKKKTSLNSLSSL---------------------NSSESS-------NLKSDL------------SSINNLNHINYKNVK-KI---------------
PRELSG_0904000        --SKKEDSSSSSYSL---------------------TSSESL-------HLKKDL------------SSLSNLNHINNQKLK-KISH-------------
PocGH01_09013500      --SSTENHSPLSRRS---------------------NAQKRT-------KMKERG------------GHKHE-------KRK-AFSS-------------
PYYM_0941700          FHDKREYTSIESNEE---------------------KNPNNP-------NLSLSI------------NSKNETNSLNGSNPT-KL---------------
PY02790               FHDKREYTSIESNEE---------------------KNPNNP-------NLSLSI------------NSKNETNSLNGSNPT-KL---------------
PY02791               ----------------------------------------------------------------------------------------------------
PBANKA_0940100        FHDKLEPEPIKPNKE---------------------KEENNP-------NLSPII------------NSKNETNLLNDSNPT-KLQD-------------
PCHAS_0904200         FHEKREHTSIASNEE---------------------KEVNEL-------NLSSSI------------NSKSETNLLNNSSPT-KLQN-------------
PCHAS_090420          FHEKREHTSIASNEE---------------------KEVNEL-------NLSSSI------------NSKSETNLLNNSSPT-KLQN-------------
YYE_02751             FHEKREHTSIASNEE---------------------KEVKEP-------NLFPNI------------NSKNETNLLNNSSPT-KLQN-------------
YYG_01693             FHEKREHISIASNEE---------------------KEIKEP-------NLSPSI------------NSKSETNLLNNSSPT-KLQN-------------
PmUG01_09016400       --SIRTKTSSLLSVE---------------------SQSKKI-------NPQSRI------------IYARGKSHMSDKNVKMKFSSPLSLPLI------
C922_04039            TSPSAQSSHPDFSSE-------------------GSIETDAC-------HVSTCE------------GESKGGDN-------------------------
PcyM_0908600          TSTSSQSSRPNHSSE-------------------ESGESDAS-------TLPTCE------------GEPKGGTN-------------------------
PKNOH_S120122800      TSTFSQSSRPDLSYE-------------------ESNKSDSS-------NVPTWQ------------GESKGEEY-------------------------
PKNH_0904400          TSTFSQSSRPDLSYE-------------------ESNKSDSS-------NVPTWQ------------GESKGEEY-------------------------
PVP01_0907600         TSTSSQSSGPDLSSE-------------------ESGESNGT-------SVPTCE------------EKPKGGDN-------------------------
PVX_091015            TSTSSQSSGPDLSSE-------------------DSGESNGT-------SVPTCE------------EKPKGGDN-------------------------
THRCLA_22740          ----------------------------------------------------------------------------------------------------
THRCLA_04632          ----------------------------------------------------------------------------------------------------
BESB_019990           --RRDDHEDSLLSLAYRGLISFSFLIPFHQLIFAEPLHAPAA-------AASAQG------------VSARWRGSWLGKPVSLKLLPS------------
BN1204_042460         --ARGEGGSCLLSLAFRGFISFSFLIPFHQLTFVQPLLSPPSPLCSWPSSCSACASCAS-CRETHSRCSTRWQGSWLGKSVCLELVPAFHLP--------
TGGT1_290225          --VPRPDSSSLHSLAFRGFLPFSFLIPFHQLTFIQPLHSPAP-------SCSSCSAFASPCRETRW-RRAKWKGSWLGKTVCLQLVPVSLLPSCQEEGSP
TGDOM2_290225         --VPRADSSSLHSLAFRGFLPFSFLIPFHQLTFIQPLHSPAP-------SCSSCSAFASPCRETRW-RRAKWKGSWLGKTVCLQLVPVSLLPSCQEEGSP
BN1205_083850         --VPRADSSSLHSLAFRGFLPFSFLIPFHQLTFIQPLHSPAP-------SCSSCSAFASPCRETRW-RRAKWKGSWLGKTVCLQLVPVSLLPSCQEEGSP
HHA_290225            --FARGESSSLHSLAFRGFVPFSFLIPFHQLTFIHPLHSPPP-------SCSSCSAFASPCRETRT-RRAKWKGSWLGKTVCLQLVPACLLPSCQEEGSP
ETH_00000110          --CSIDKGTPNLGSL----------IPFEQLHFVRCLGGRR---------------------------TSVFYGFWLGKEVAIKVFRKQHF---------
ENH_0020460           --CSIDKGTPNLGSL----------IPFEQLHFVRCLGGRR---------------------------TSVFYGFWLGKEVAIKVFRKQHF---------
EMWEY_00023200        --AGGQQGAPFSSGGRVG-----------------------------------------------------------GKEVAIKVFKKQHF---------
Hs_RAF1               --SQRQRSTSTPNVHMVS----------------------------------------------------------------------------------
Pa_RAF1               --SQRQRSTSTPNVHMVS----------------------------------------------------------------------------------
Mm_RAF1               --SQRQRSTSTPNVHMVS----------------------------------------------------------------------------------
Rn_RAF1               --SQRQRSTSTPNVHMVS----------------------------------------------------------------------------------
Bt_RAF1               --SQRQRSTSTPNVHMVS----------------------------------------------------------------------------------
Gg_RAF1               --SQRQRSTSTPNVHMVS----------------------------------------------------------------------------------
Xl_RAF1               --SQRQRSTSTPNVHMVS----------------------------------------------------------------------------------
Ce_RAF1               EEESRDKTGSLLSTQARH--------------------------------------------------RPHFQSG--------HILSGARM---------
GNI_162980            --TSRWSTGAAGATA---------------------TGATAT-------GTNCLG------------TKASETNT--------RLLSAK-----------
```

```
                        1610      1620      1630      1640      1650      1660      1670      1680      1690      1700
                   ....|....|....|....|....|....|....|....|....|....|....|....|....|....|....|....|....|....|....|....|
PF3D7_1106800      -----------------------------ILN---------------KSSSEFSPSHSYTSKSYH--YNIKPSLQSSSNNSSDSSYSISSTCSSSSSY--
PFIT_1107600       -----------------------------ILN---------------KSSSEFSPSHSYTSKSYH--YNIKPSLQSSSNNSSDSSYSISSTCSSSSSY--
PPRFG01_1107500    -----------------------------ILN---------------KSSSEFSPSHSYTSKSYH--YNIKPSLQSSSNNSSDSSYSISSTCSSSSSY--
PRCDC_1105300      -----------------------------ILN---------------KSSSEFSPSHSYTSESYD--YNIKPSLQSASNNSLDSSCSIYSTCSSSSSY--
PRG01_1104000      -----------------------------ILN---------------KSSSEFSPSHSYTSESYD--YNIKPSLQSASNNSLDSSCSIYSTCSSSSSY--
PBILCG01_1104000   -----------------------------ILY---------------KSSCEFSPSHSYTSESYH--YNIKSSLHPASNNSFDSSCSISSTCSSSSSY--
PBLACG01_1104300   -----------------------------ILC---------------KSSSELSPSHSYTSESYQ--YNIKPSLQSSSNNSLDSSCSISSTCPSSSSY--
PADL01_1105000     -----------------------------VLN---------------KSSSEVSSSHSYTSESYR--YNIKPSLQSSYNNSLVSSCSMSSTCPSSSSY--
PGABG01_1103900    -----------------------------IVN---------------KSSSEVSSSHSYTSESYR--YNINPSLQSSYNNSLVSSCSMSSTCPSSSSY--
PGSY75_1106800     -----------------------------IVN---------------KSSSEVSSSHSYTSESYC--YNINPSLQSSYNNSLVSSCSMSSTCPSSSSY--
PGAL8A_00334100    -------------------------------------------------ASFSDSSTSFSSSSSS--------------SSSSSSSSLSSSSSSSALV--
PRELSG_0904000     -----------------------------SSA---------------ISSSFSSSSSSSSSSSSS--------------SSSSSSSSLSSYSSSSALA--
PocGH01_09013500   -----------------------------SAP---------------SASSASSASSASSASSAS--------------SASSASSTL---STSSTVT--
PYYM_0941700       ------------------------------LP---------------KQPCSLASENMAENMSEKLSENMAENMAEKLSASSSSSSSSASASASASSSSE-
PY02790            ------------------------------LP---------------KQPCSLASENMAENMSEKLSENMAENMAEKLSASSSSSSSSASASASASSSSE-
PY02791            ----------------------------------------------------------------------------------------------------
PBANKA_0940100     -----------------------------ELP---------------KSPCSIDSE-----SSSE--------------------------KSSETSSE-
PCHAS_0904200      -----------------------------ELP---------------KLSHSSGLETSFSSSSSE--------------------------KSSETFS--
PCHAS_090420       -----------------------------ELP---------------KLSHSSGLETSFSSSSSE--------------------------KSSETFS--
YYE_02751          -----------------------------ELP---------------KPSHSSGSETSFSSSSSE--------------------------KSSETFS--
YYG_01693          -----------------------------ELP---------------KPVHSSGSETSFSSSSSE--------------------------KSSETLS--
PmUG01_09016400    --------------------------SPQKTP---------------QPPASNLSTNPASSSSSS--------------SSSSSASS----CTSSLLS--
C922_04039         -----------------------------WII---------------ASSRKLEFSY--TSTSSS---------------SASTSLDTSPHASTSSVS--
PcyM_0908600       -----------------------------SML---------------ESSAELELSVASTSSSSS---------------SASASLNTSSHASTSSVS--
PKNOH_S120122800   -----------------------------SML---------------ASSAELEFSV--TSTSSS---------------SASSSLDTSPPSSTSSVS--
PKNH_0904400       -----------------------------SML---------------ASSAELEFSV--TSTSSS---------------SASSSLDTSPPSSTSSVS--
PVP01_0907600      -----------------------------SIR---------------ASSAELEISA--ASTSSS---------------SASATLDTSPHASTSSVS--
PVX_091015         -----------------------------SIR---------------ASSAELEISA--ASTSSS---------------SASATLDTSPHASTSSVS--
THRCLA_22740       ----------------------------------------------------------------------------------------------------
THRCLA_04632       ----------------------------------------------------------------------------------------------------
BESB_019990        ----------------------------FDSP---------------LGDALEARGRGERSASSSF-------------ASSACWPRSRFRQSHEPEAGR
BN1204_042460      ----GEAAEDGDAAHDTQSPETKHCEFSLQEP-NACVSERSHASRGQTETGKTDPQVASSSSSSS-------------SSSSSSSSSSSSLSSSSPCVVR
TGGT1_290225       VWGSGGKSTDRRLAGNSQDPRGR---VSRSHSGGETLTQQRARSRSYAASSSLP---SSSSRSASGPASI------ASSSSSPCPSSLPSFSSSSSLFQR
TGDOM2_290225      VWGSGGKSTDRRRAGNSQDPRGR---VSRSHSGGETLTQQRARSRSYAASSSLP---SSSSRSASGPASV------ASSSSSPCSSSLPSFSSSSSLFQR
BN1205_083850      VWGSGRKSTDRRRAGNSQDPRGR---VSRSHSGGETLTQQRARSRSYAASSSLPSSSSSSSRSASGPASV------ASSSSSPCSSSLPSFSSSSSLFQR
HHA_290225         FCTTGRKSTDTQRAGDVQGPCGR---VSRSQS-TETLTQEHTRNRSFSSSSSLSSSSSSLSSSSS--------------SSSPSSPS-PSFSSSSSLFQP
ETH_00000110       ---------------------------FYHMP---------------TSAAVPAAAAAAAAAAAA--------------AAAAGPPWNEGPLTFPLLEG-
ENH_0020460        ---------------------------FYHMP---------------TSAAVPAAAAAAAAAAAA--------------AAAAGPPWNEGPLTFPLLEG-
EMWEY_00023200     ---------------------------FYHMP---------------TSAAAPAAAASAAAAASA--------------AAAAGPPWNEGPLTFASLTGG
Hs_RAF1            ----------------------------TTLP---------------VDSRMIEDAIRSHSESAS-------------PSALSSSPNNLSPTGWSQPKT-
Pa_RAF1            ----------------------------TTLP---------------VDSRMIEDAIRSHSESAS-------------PSALSSSPNNLSPTGWSQPKT-
Mm_RAF1            ----------------------------TTLH---------------VDSRMIEDAIRSHSESAS-------------PSALSSSPNNLSPTGWSQPKT-
Rn_RAF1            ----------------------------TTLP---------------VDSRMIEDAIRSHSESAS-------------PSALSSSPNNLSPTGWSQPKT-
Bt_RAF1            ----------------------------ATLP---------------VDSRMIEDAIRSHSESGS-------------PSALSSSPNNLSPTGWSQPKT-
Gg_RAF1            ----------------------------TTMP---------------VDSRIIEDAIRNHSESAS-------------PSALSGSPNNMSPTGWSQPKT-
Xl_RAF1            ----------------------------TTMA---------------VDSRVIEDALRSHSE--------------------SGSPNNLSPTGWSNAKA-
Ce_RAF1            ---------------------------NRLHP--------------LVDCTPLGSNSPSSTCSSP-PGGL------IGQPTLGQSPNVSGSTTSSLVAAH
GNI_162980         -----------------------------------------------THTSVAEPGDGNRSQDRA-----------LAESAVSSSSSSASSAKTDELL--

                        1710      1720      1730      1740      1750      1760      1770      1780      1790      1800
                   ....|....|....|....|....|....|....|....|....|....|....|....|....|....|....|....|....|....|....|
PF3D7_1106800      ---------------------------------VSSLYSNRSNDILNFYRNKIIKYCNNI-----------YMNTKLAYSYMNGFII-------------
PFIT_1107600       ---------------------------------VSSLYSNRSNDILNFYRNKIIKYCNNI-----------YMNTKLAYSYMNGFII-------------
PPRFG01_1107500    ---------------------------------VSSLYSNRSNDILNFYRNKIIKYCNNI-----------YMNTKLAYSYMNGFII-------------
PRCDC_1105300      ---------------------------------VSSLYSNRSNDILNFSRNKIIKYCNNI-----------YMNTKLAYSYMNGFII-------------
PRG01_1104000      ---------------------------------VSSLYSNRSNDILNFSRNKIIKYCNNI-----------YMNTKLAYSYMNGFII-------------
PBILCG01_1104000   ---------------------------------ISSLCSNGSNDIMDFSRNKIIKYCNNI-----------YMNTKLAYSYINGFII-------------
PBLACG01_1104300   ---------------------------------VSSLCSMGSNDILNFSRNKIIKYCNNI-----------YMNTKLAYSYMNGFII-------------
PADL01_1105000     ---------------------------------ISSLFSNASNDIVNFNRNKIIKYCNNI-----------YMNTKLAYSYMNGFII-------------
PGABG01_1103900    ---------------------------------ISSLCSNASNDIVNFNRNKIIKYCNNI-----------YMNTKLAYSYMNGFII-------------
PGSY75_1106800     ---------------------------------ISSLCSNASNDIVNFNRNKIIKYCNNI-----------YMNTKLAYSYMNGFII-------------
PGAL8A_00334100    ---------------------------------SSSFCSSRSDDRIFYPRNKIIKYTNSI-----------YMNNNLTFSYLYSFII-------------
PRELSG_0904000     ---------------------------------SSSFCSSKSDDIIFYPRNKIIKYTNNI-----------YMNNNLTFSYLYSFII-------------
PocGH01_09013500   ---------------------------------SSSFCSGGSNDALFRVRNKIIQHSSNI-----------YMNNHLTFSYLYSFMI-------------
PYYM_0941700       ---------------------------------TSSFCSEHSELTNFHNNNKIIKYSNNI-----------YINSSLAFSYIYSFII-------------
PY02790            ---------------------------------TSSFCSEHSELTNFHNNNKIIKYSNNI-----------YINSSLAFSYIYSFII-------------
PY02791            ----------------------------------------------------------------------------------------------------
PBANKA_0940100     ---------------------------------TSSFCSEHSEFANFHNNNKIVKYSNNI-----------YINSSLAFSYIYSFII-------------
PCHAS_0904200      ---------------------------------SSSFCSEHSEMDNFHNSNKIVKYSNNI-----------YINSSLAFSYIYSFII-------------
PCHAS_090420       ---------------------------------SSSFCSEHSEMDNFHNSNKIVKYSNNI-----------YINSSLAFSYIYSFII-------------
YYE_02751          ---------------------------------SSSFCSEHSEMDNFHDSNKIVKYSNNI-----------YINSTLAFSYIYSFII-------------
YYG_01693          ---------------------------------SSSFCSEHSEMDNFHNSNKIVKYSNNI-----------YINSSLAFSYIYSFII-------------
PmUG01_09016400    ---------------------------------SSSFCSSRREDIIFSTRNKIIKYSNNI-----------YMNNNLTFSYLYSFII-------------
C922_04039         ---------------------------------SSSYCSNKMDDAPFYGRSQIIKYPSNI-----------YLNNNLAFSYLYSFII-------------
PcyM_0908600       ---------------------------------SSSYCSNRTDDAPFYGRSQIIKYPSNI-----------YLNNSLAFSYLYSFII-------------
PKNOH_S120122800   ---------------------------------SSSYCSNRTDDAPFYGRSQIIKYPSNI-----------YLNNSLAFSYLYSFII-------------
PKNH_0904400       ---------------------------------SSSYCSNRTDDAPFYGRSQIIKYPSNI-----------YLNNSLAFSYLYSFII-------------
PVP01_0907600      ---------------------------------SSSYCSNRTDDAPFYGRSQIIKYPSNI-----------YLNSNLAFSYLYSFII-------------
PVX_091015         ---------------------------------SSSYCSNRTDDAPFYGRSQIIKYPSNI-----------YLNSNLAFSYLYSFII-------------
THRCLA_22740       ----------------------------------------------------------------------------------------------------
```

```
THRCLA_04632      ----------------------------------------------------------------------------------------------------
BESB_019990       A--------------------------ARGAAVDGGGREETREDAGSQERQAAVDTAH--A-SARRCKEKKTRKQKERNGASDAQTE-------------
BN1204_042460     GRGSSGSSQRRSRLERSGRSPHAKGS-HGVAEGEPGAVGSASGGAGERRRRQRGKREKKPK-----EGPAARPDAGEEGAAEPPARLERSQGEKTGEARA
TGGT1_290225      GGLAASSESLRLRGD-ADKAERTKGHPAGSALATPGEAVQEKSDGEGRRRRRRGKREERPAGTSREKAAADERNAENRTEAAQATVLLDSDRHQIRERRA
TGDOM2_290225     GGLAASSESLRLRGD-ADKAEITKGHPAGSALATPGEAVHEKSDGEGRRRRRRGKREERPAGTSREKAAADERNAENRTEAAQATVLLDSDRHQIRERRA
BN1205_083850     GGLAASSESLRLRGD-ADKAERTKGHPAGSALATPGEAVHEKSDGEGSRRRRRGKREERPAGTSREKAAADERNAENRTEAAQATVLLDSDRHQIRERRA
HHA_290225        GGLAASSAILRVRGD-TGETESTKGPPAGSALATPGEAVRGKSDGEGCRRRRRGKREESPAGTSREKAAAEERNAEKKTETARPTVL-DSDRNQIRQRYA
ETH_00000110      ----------------------------------------------------------------------------------------------------
ENH_0020460       ----------------------------------------------------------------------------------------------------
EMWEY_00023200    ----------------------------------------------------------------------------------------------------
Hs_RAF1           ---------------------------------PVPAQRERAPVSGTQEKNKIRPRGQ-------------------RDSSYYWEIE-------------
Pa_RAF1           ---------------------------------PVPAQRERAPVSGTQEKNKIRPRGQ-------------------RDSSYYWEIE-------------
Mm_RAF1           ---------------------------------PVPAQRERAPGSGTQEKNKIRPRGQ-------------------RDSSYYWEIE-------------
Rn_RAF1           ---------------------------------PVPAQRERAPGSGTQEKNKIRPRGQ-------------------RDSSYYWEIE-------------
Bt_RAF1           ---------------------------------PAPAQRERAPGSSTQEKNKIRPRGQ-------------------RDSSYYWEIE-------------
Gg_RAF1           ---------------------------------PVPAQRERAPGTNTQEKNKIRPRGQ-------------------RDSSYYWEIE-------------
Xl_RAF1           ---------------------------------PAPTHREKAASSTGQEKNKIRARGQ-------------------RDSSYYWEII-------------
Ce_RAF1           ---------------------------LHTLPLTPPQSAPPQKISPGFFRNRSRSPGERL--------DAQRPRPPQKPHHEDWEIL-------------
GNI_162980        ---------------------------------PSPGLPLGIRLELEQLRDQIRRCGAVP----LEGSGCKLSGCKLSGCKLFGCGL-------------

                          1810      1820      1830      1840      1850      1860      1870      1880      1890      1900
                  ....|....|....|....|....|....|....|....|....|....|....|....|....|....|....|....|....|....|....|....|
PF3D7_1106800     -----------PHEDLIFIHPIENYYMDNTNEKNNINNPYTKEK----IMNHNFSFNTKNNT-------------------SFIDINTNIFSS--NKQQN
PFIT_1107600      -----------PHEDLIFIHPIENYYMDNTNEKNNINNPYTKEK----IMNHNFSFNTKNNT-------------------SFIDINTNIFSS--NKQQN
PPRFG01_1107500   -----------PHEDLIFIHPIENYYMDNTNEKNNINNPYTKEK----IMNHNFSFNTKNNT-------------------SFIDINTNIFSS--NKQQN
PRCDC_1105300     -----------PHEDLIFIHPIENYYMDNTNENNNINNRYTKEK----IMNHNFSFNTKNNT-------------------SFIDINTNIFSS--NKQQN
PRG01_1104000     -----------PHEDLIFIHPIENYYMDNTNENNNINNRYTKEK----IMNHNFSFNTKNNT-------------------SFIDINTNIFSS--NKQQN
PBILCG01_1104000  -----------PHEDLIFIHPIENYYIDNTNENNNINNRYTKEK----MINHNFTFNTKSNV-------------------PFVDINTNIFST--NKQQN
PBLACG01_1104300  -----------PHEDLIFIHPIENYYMDNTNENNNINNCYTKEK----IINNNFSFNTKNNT-------------------SFIDMNTNIFST--NKQQN
PADL01_1105000    -----------PHEDLIFIHPIENYYIDNTNEIYNINNRYIKDN----IINHNFSFNKKNNT-------------------SFIDINTNIFST--NKEQN
PGABG01_1103900   -----------PHEDLIFIHPIENYYIDNTNEIYNINNSYIKDN----IINHNFSFNKKKKK-------------------SFIDINTNIFST--NKETN
PGSY75_1106800    -----------PHEDLIFIHPIENYYIDNTNEIYNINNTYIKDN----IINHNFSFNNKKKK-------------------SFIDINTNIFST--NKETN
PGAL8A_00334100   -----------PHEDLIFLHQIENYYFGDMYNNDNNNIDFN--------FEEKFQFMKKIKA-------------------LTLDNEENKYS-----KKN
PRELSG_0904000    -----------PHEDLIFLHPIENYYISDINNSDDNNINFN--------IEEKFLFKNNIKV-------------------PFLKNVEN--------NYN
PocGH01_09013500  -----------SHEDLIFLRPIENFYVWDATNHSN----IS--------SEGSFSYGSTMKA--------------------IFPNS--------GIYER
PYYM_0941700      -----------PPEDLTFLYQIQNYYIRDVENDLNRNDQVD--------FCDSFNFYKNFEIIKNRKINPDDRMCSYSSHHSKFEQNKNKNDFNYNDCKN
PY02790           -----------PPEDLTFLYQIQNYYIRDVENDLNRNDQVD--------FCDSFNFYKNFEIIKNRKINPDDRMCSYSSHHSKFEQNKNKNDFNYNDCKN
PY02791           ----------------------------------------------------------------------------------------------------
PBANKA_0940100    -----------PPENLTFLYQIRNYYVRDVENDLNPNNELD--------FCDSFNFYKNCEIIKNRIINPGIRNCSYASNHSKFQQNKNRKYFNYSDCKN
PCHAS_0904200     -----------PPENLTFLYQIKNYYIRDVENDLSPTNEVD--------FVDKFNFYTNREI-----------------------------------AKN
PCHAS_090420      -----------PPENLTFLYQIKNYYIRDVENDLSPTNEVD--------FVDKFNFYTNREI-----------------------------------AKN
YYE_02751         -----------PPENLTFLYQIKNYYIRDSENDLNPNNEID--------FFDKFNFYKNREM-----------------------------------AKN
YYG_01693         -----------PPENLTFLYQIKNYYIRDIENDLNPNNEVD--------FFDKFNFYKNREM-----------------------------------AKN
PmUG01_09016400   -----------PHEDLTFLHHIENYYIIDENQSSINSKDIT--------IDENFHFKKKEKN-------------------SLLNYKRGSSCS----EKG
C922_04039        -----------PHEDLTFLHLIRNHYEVRSGRCKNVRQGQGQMQ------GQNLLATNSNPN---------------------------------KLKSM
PcyM_0908600      -----------PHEDLTFLHLIRNHYDIRTRRSEHEWQGQGQQQNPNPNWNQNLLATNSKQN---------------------------------KLKRM
PKNOH_S120122800  -----------PHEHLTFLHLIRNHYHIRTRRCKDGRKDQEEEQ--------NLFVTNTKSN---------------------------------KFKSI
PKNH_0904400      -----------PHEHLTFLHLIRNHYHIRTRRCKDGRKDQEEEQ--------NLFVTNTKSN---------------------------------KFKSI
PVP01_0907600     -----------PHEELTFLHLIRSHYEIRTCRGKLARKKRGQGQWQNRGQWQSLPPTNSEPK---------------------------------KPNPM
PVX_091015        -----------PHEELTFLHLIRSHYEIRTCRGKLARKKRGQGLWQNRGQWQSLPPTNSEPK---------------------------------KPNPM
THRCLA_22740      -----------PHNA-------------------------------------------------------------------------------------
THRCLA_04632      ----------------------------------------------------------------------------------------------------
BESB_019990       ----------VPLRT-------EGEDAKQEAEMSDGAAKIG-------NEGEESDAASTPRR-------------SL----SPFALPQSDTLPA-ACRAT
BN1204_042460     VTDCAA----LGEEAGIERPSSRHSTPQGPTNLPAETRPRGETR----CWTEALSHASCEER-------------SEHSQEPLFTAPFLSAAPV-VRRPS
TGGT1_290225      NISPAAARP-TPAERCDRQGEVQKEAQREVQGEDEEERGRG--------RHDASSHGVFERR-------------D-RVQVSSLPASLAAFHPT-TRRPS
TGDOM2_290225     NISPAAARP-TPAERCDRQGEVQKEAQREVQGEDEEERGRD--------RHDASSHGVFERR-------------D-RVQVSSLPASLAAFHPT-TRRPS
BN1205_083850     NISPAAARP-TPAERCDRQGEVQKEAQREVQGEDEEERGRD--------RQDASSHGVFERR-------------D-RVQVSSLLASLAAFHPT-TRRPS
HHA_290225        HASPGAARPIPPEERRDREREAQNEAQRELQGEDEAERGKG--------RHESSSHGVFERR-------------G-RVHVPSLPASLAAFHPT-TRRPS
ETH_00000110      -----------------------------------------------------------------------------------------------DTRSS
ENH_0020460       -----------------------------------------------------------------------------------------------ETRSS
EMWEY_00023200    -----------------------------------------------------------------------------------------------AQNTN
Hs_RAF1           -----------ASEVML------------------------------------------------------------------------------STRIG
Pa_RAF1           -----------ASEVML------------------------------------------------------------------------------STRIG
Mm_RAF1           -----------ASEVML------------------------------------------------------------------------------STRIG
Rn_RAF1           -----------ASEVML------------------------------------------------------------------------------STRIG
Bt_RAF1           -----------ASEVML------------------------------------------------------------------------------STRIG
Gg_RAF1           -----------ASEVML------------------------------------------------------------------------------STRIG
Xl_RAF1           -----------ASEVML------------------------------------------------------------------------------SSRIG
Ce_RAF1           -----------PNEFII------------------------------------------------------------------------------QYKVG
GNI_162980        -----------PSESFTSISSLVREFLIPIEEL-------------------------------------------------------------------

                          1910      1920      1930      1940      1950      1960      1970      1980      1990      2000
                  ....|....|....|....|....|....|....|....|....|....|....|....|....|....|....|....|....|....|....|....|
PF3D7_1106800     INNFG-KYKKM------KSRMFKGKYMGKEVAIK---ILVGKIKNFKKLHQILYNLYNLRHSNLVLIMGVSIHY--------PFVF-IIYEYMKNKCLFS
PFIT_1107600      INNFG-KYKKM------KSRMFKGKYMGKEVAIK---ILVGKIKNFKKLHQILYNLYNLRHSNLVLIMGISIHY--------PFVF-IIYEYMKNKCLFS
PPRFG01_1107500   INNFG-KYKKM------KSRMFKGKYMGKEVAIK---ILVGKIKNFKKLHQVLYNLYNLRHSNLVLIMGVSIHY--------PFVF-IIYEYMKNKCLFS
PRCDC_1105300     INNFG-KYKKM------KSRMFKGKYMGKEVAIK---ILVGKIKNFKKLHKILYNLYNLRHSNFVLIMGVSIHY--------PFVF-IIYEYMKNKCLFS
PRG01_1104000     INNFG-KYKKM------KSRMFKGKYMGKEVAIK---ILVGKIKNFKKLHKILYNLYNLRHSNFVLIMGVSIHY--------PFVF-IIYEYMKNKCLFS
PBILCG01_1104000  INNFA-KYKKM------KSRMFKGKYMGKEVAIK---VLVGKIKNFKKLHQTLYKLYNLRHSNLVLIMGVSIHY--------PFLF-IIYEYMKNNCLFS
PBLACG01_1104300  INNFG-KYKKM------KSRMFKGKYMGKEVAIK---ILVGKIKNFKKLHQILYNLYNLRHTNLVLIMGVSIHY--------PFVF-IIYEYMKNKCLFS
PADL01_1105000    INNFV-KYKKM------KSRMFKGKYMGKEVAIK---ILVGKIKNFKKLHQILYNLYNMRHSNFVLIMGVSIHY--------PFIF-IIYEYMKNKCLFS
PGABG01_1103900   INNFM-KYKKM------KSRMFKGKYMGKEVAIK---ILVGKIKNFKKLHQILYNLYNMRHSNFVLIMGVSIHY--------PFIF-IIYEYMKNKCLFS
```

```
PGSY75_1106800    INNFV-KYKKM------KSRMFKGKYMGKEVAIK---ILVGKIKNFKKLHQILYNLYNMRHSNFVLIMGVSIHY--------PFIF-IIYEYMKNKCLFS
PGAL8A_00334100   KENEV-KYTKL------KSRVFKGKYMGKEVAIK---VLVGKIKNFNKLHKILYKLYILRHSNIVLIMGVSISY--------PFVF-IVYEYLKNLCLFS
PRELSG_0904000    KNKSP-KYRKL------KSRVFKGKYMGKEVAIK---VLVGKIKNFNKLHKILYKLYILRHSNIVLIMGISISY--------PFVF-IVYEYLKNLCLFS
PocGH01_09013500  EKTSI-RYKRL------HSRVFLGKYMGKDVAIK---VLVGKITKFHKLYRILYKLYTLRHSNIVLIMGVSIKY--------PFLF-IVYEHVKNLCLFS
PYYM_0941700      EREKT-KPQKM------KSRIFRGRYMGKDVAIK---VLVGNIKNFTKFHKVLYNLYILRHTNIALIMGVSISY--------PFVF-IIYEYIKNLCLFS
PY02790           EREKT-KPQKM------KSRIFRGRYMGKDVAIK---VLVGNIKNFTKFHKVLYNLYILRHTNIALIMGVSISY--------PFVF-IIYEYIKNLCLFS
PY02791           ----------------------------------------------------------------------------------------------------
PBANKA_0940100    ERKKT-KPQKM------KSRVFRGRYMGKDVAIK---VLVGNIKNFTKFHKVLYKLYILRHTNIALIMGVSISY--------PFVF-IIYEYIKNLCLFS
PCHAS_0904200     ERDNI-KPQKM------KSRVFRGRYMGKDVAIK---VLVGNIKNFTKFHKVLYKLYILRHTNIALIMGVSISY--------PFVF-IIYEYVKNLCLFS
PCHAS_090420      ERDNI-KPQKM------KSRVFRGRYMGKDVAIK---VLVGNIKNFTKFHKVLYKLYILRHTNIALIMGVSISY--------PFVF-IIYEYVKNLCLFS
YYE_02751         GKEYT-KPQKM------KSRVFRGRYMGKDVAIK---VLVGNIKNFTKFHKVLYKLYILRHTNIALIMGVSISY--------PFVF-IIYEYVKNLCLFS
YYG_01693         GKENT-KPQKM------KSRVFRGRYMGKDVAIK---VLVGNIKNFTKFHKVLYKLYILRHTNIALIMGVSISY--------PFVF-IIYEYVKNLCLFS
PmUG01_09016400   NGNTF-NYRKL------KSRVFKGKYMGKDVAIK---VLVGKIKNFYHFHKMIYKLYVLRHSNIALIMGVAIYY--------PFVF-IVYEYLKNRCLFS
C922_04039        KGDLK-NGKTM------QSRTFRGKYLGKDVAIK---VLVGRVKDFFQIHKIFYKLHLFGHGNIALMMGVSIRY--------PFVF-IIYEFLKNGCLFS
PcyM_0908600      KGDLK-NGKIM------GSRTFRGKYLGKDVAIK---VLVGRVKDFSQIHKIFYKLHILRHGNIALMMGVSIRY--------PFVF-IVYEFLKNTCLFS
PKNOH_S120122800  KGNLK-NDKIM------NSRTFRGKYLGKDVAIK---VLVGRVKDFSEIHKVFYKLHLLRHGNIALMMGVSIRY--------PFVF-IISEFLKNGCLFS
PKNH_0904400      KGNLK-NDKIM------NSRTFRGKYLGKDVAIK---VLVGRVKDFSEIHKVFYKLHLLRHGNIALMMGVSIRY--------PFVF-IISEFLKNGCLFS
PVP01_0907600     RGDLQ-NGKVM------RSRTFRGKYLGKDVAIK---VLVGRVKDFSQIHKIFYKLHLLRHGNIALMMGVSIRY--------PFVF-IIYEFLKNACLFS
PVX_091015        RGDLQ-NGIVM------RSRTFRGKYLGKDVAIK---VLVGRVKDFSQIHKIFYKLHLLRHGNIALMMGVSIRY--------PFVF-IIYEFLKNACLFS
THRCLA_22740      -------------------------------------VLIGT---------TIWGLICHRHANIIPLIGLC-SYLM------NNIY-LAMESMNQGSLRS
THRCLA_04632      ------------------------------------------------------------------------------------------ESLNQGSLRS
BESB_019990       IDALPLSLSSFFPGSAPSSSSLVAPASGAAI------PDFLSPRELAVL--LLYRLSLLRHPHLVLVLGVSEGS-CVVCGQGPVVW-AVTEFLPWRSLAF
BN1204_042460     ASSLSSSFSVF------SSSACPSSAAFQSQRQ----PLLSHPRAVATL--ILYRLSLLRHPHLVLLLGVADGV-CPLCGSGPALW-LVTEFLPRRSLSF
TGGT1_290225      ASSLS-SFSAF------SSSAFPSFAGGQRLGEV---PRFFSPRELAVL--ILYRISLLRHPHLVLLLGVSDGA-CTLCGRGPALW-LVTEFLPKRSLSF
TGDOM2_290225     ASSLS-SFSAF------SSSAFPSFAGGQRLGEV---PRFFSPRELAVL--ILYRISLLRHPHLVLLLGVSDGA-CTLCGRGPALW-LVTEFLPKRSLSF
BN1205_083850     ASSLS-SFSAF------SSSAFPSFAGGQRLGEV---PRFFSPRELAVL--ILYRISLLRHPHLVLLLGVSDGA-CTLCGRGPALW-LVTEFLPKRSLSF
HHA_290225        ASSLS-SFSAF------SSSACPSFAGGQPLGEA----RLFSPRELAVL--ILYRLSLLRHPHLVLLLGVSDGV-CTLCGRGPALW-LVTEFLPKRSLSL
ETH_00000110      RVSMG------------SPRHLSRDAAG-KLREK---LVEVQCNNTDLLLQHLLQLQQLRHPHLVMLLGVSLSH--------SFHVCLVMEYLSKGPLGA
ENH_0020460       RASMG------------SPSHLSGDAAG-KLREK---LVEVQCNNTDLLLQHLLQLQQLRHPHLVMLLGVSLSH--------SFHVCLVMEYLSKGPLGA
EMWEY_00023200    SGGGG---APM------GGGPLK------EIEDK---LLDVLCNNTDLLLLQLLQLQQLRHPHLVMLLGVSLSH--------SFHVCLVMEYMPKGPLGM
Hs_RAF1           SGSFG--------------TVYKGKWHG-DVAVKILKVVDPTPEQFQAFRNEVAVLRKTRHVNILLFMGYMTK---------DNLA-IVTQWCEGSSLYK
Pa_RAF1           SGSFG--------------TVYKGKWHG-DVAVKILKVVDPTPEQFQAFRNEVAVLRKTRHVNILLFMGYMTK---------DNLA-IVTQWCEGSSLYK
Mm_RAF1           SGSFG--------------TVYKGKWHG-DVAVKILKVVDPTPEQLQAFRNEVAVLRKTRHVNILLFMGYMTK---------DNLA-IVTQWCEGSSLYK
Rn_RAF1           SGSFG--------------TVYKGKWHG-DVAVKILKVVDPTPEQLQAFRNEVAVLRKTRHVNILLFMGYMTK---------DNLA-IVTQWCEGSSLYK
Bt_RAF1           SGSFG--------------TVYKGKWHG-DVAVKILKVVDPTPEQFQAFRNEVAVLRKTRHVNILLFMGYMTK---------DNLA-IVTQWCEGSSLYK
Gg_RAF1           SGSFG--------------TVYKGKWHG-DVAVKILKVVDPTPEQFQAFRNEVAVLRKTRHVNILLFMGYMTK---------DNLA-IVTQWCEGSSLYK
Xl_RAF1           SGSFG--------------TVYKGKWHG-DVAVKILKVTDPTPEQLQAFRNEVAVLRKTRHVNILLFMGYMTK---------DNLA-IVTQWCEGSSLYY
Ce_RAF1           SGSFG--------------TVYRGEFFG-TVAIKKLNVVDPTPSQMAAFKNEVAVLKKTRHLNVLLFMGWVRE---------PEIA-IITQWCEGSSLYR
GNI_162980        ------KFEALLPGA--TSSTYRGSWLGKTVAIK---VFTGKLIDSHAWVQYCKMLAALAHPNLIQVLGVAMKP--------PFLWCVVSEYLHKGSLAN

                           2010      2020      2030      2040      2050      2060      2070      2080      2090      2100
                  ....|....|....|....|....|....|....|....|....|....|....|....|....|....|....|....|....|....|....|....|
PF3D7_1106800     YLHCIKYKHVYISTFLQRYKTLLHITQQEKIK-------KTNNINNNNNINHNNIN--------NNNI-NHNNINHNNINNNNINNNNIN----------
PFIT_1107600      YLHCIKYKHVYISTFLQRYKTLLHITQQEKIK-------KTNNINNNNNINHNNIN--------NNNI-NHNNINHNNINNNNINNNNIN----------
PPRFG01_1107500   YLHCIKYKHVYISTFLQRYKTLLHITQQEKIK-------KTNNINNNNINNNNNIN-------NNNNINNNNNINHNNINNNNINNNNIN----------
PRCDC_1105300     YLHCIKYKHVYISTFLQRYKTLLHITQQEKIK-------KTSNINN------------------------------------------------------
PRG01_1104000     YLHCIKYKHVYISTFLQRYKTLLHITQQEKIK-------KTSNINN------------------------------------------------------
PBILCG01_1104000  FLHCIKYKHVYISTFLQKYKTLLHRTQQEKIK-------KI----------------------------------------NSNNNNNIR----------
PBLACG01_1104300  FLHCIKYKHVYISTFLEKYKTLLHITQQEKIKK------KINNNNNNNNNNNN-----------NNN------------NNNNNNNNNKS----------
PADL01_1105000    YLHCIKYKHVYISTFFQKYKTLLYNTQQEEIK-------KINT-----------------------------------------NK--KK----------
PGABG01_1103900   YLHCIKYKHVYISTFFQKYKTLLYNTQQEEIK-------KINKKKN--KKNKKN----------KKN------------KKNKKNKKNKK----------
PGSY75_1106800    YLHCIKYKHVYISTFFQKYKTLLYNTQQEEIK-------KINKKKN------------------KKN------------KKNKKNKKNKK----------
PGAL8A_00334100   YLHCIKYKHIYISKFFKKYKMNYNLDLDKKI----------TDYKSEN-ESKII----------YPN------------VRF-SND--------------
PRELSG_0904000    YLHCIKYKHVYISNFFKKYKMNYNLDLDKTL----------TDYKSRN-EHKNF----------YSN------------IKSSNNN--------------
PocGH01_09013500  YLHCIKHKHIYISKYIQKKKTINNFNEHKMIL--------CNDYMSSSIDSQCM----------YKH------------QKLYKKDT--KIYDDLYN---
PYYM_0941700      YLHCIKYKHIYVSKLLKYYQ--------KKL----------NSQNSQQ-KNNTI----------FSD------------RKYLSNDD-------------
PY02790           YLHCIKYKHIYVSKLLKYYQ--------KKL----------NSQNSQQ-KNNTI----------FSD------------RKYLSNDD-------------
PY02791           ----------------------------------------------------------------------------------------------------
PBANKA_0940100    YLHCVKYKHIYVSKLLKYYQ--------KKF----------INQNFQQ-QNNTM----------SSD------------RKYISNDDNEKINFDSRNI-L
PCHAS_0904200     YLHCVKYKHIYFSKLLKYYQ--------KKF----------PNQNGQQ----------------------------------------------------
PCHAS_090420      YLHCVKYKHIYFSKLLKYYQ--------KKF----------PNQNGQQ----------------------------------------------------
YYE_02751         YLHCIKYKHIYFSKLLKYYQ--------KKY----------PNQNDQQ----------------------------------------------------
YYG_01693         YLHCIKYKHIYFSKLLKYYQ--------KKF----------PNQNDQQ----------------------------------------------------
PmUG01_09016400   YLHCIKNEQVYISKFFKKYKRSCISGVHKGEST------KGSNNNGSSGSNGSR----------GSS------------RSSGSSGS----SGSSGS---
C922_04039        YLHCAGG---YVKDVSVRQHGGGVIAAQA-------------------------------------N------------ASSISRGGGIS----------
PcyM_0908600      YLHCAGG---YVKDVSVRQHGGGVRTPLGSASSI----SRGDGISAHS-ISRGG----------GIS------------AHSISRGGGIS----------
PKNOH_S120122800  YLHCTGS---YVKDLSVRQNVGTVRADLG-------------------------------------S------------VSTMSREGDIS----------
PKNH_0904400      YLHCTGS---YVKDLSVRQNVGTVRADLG-------------------------------------S------------VSTMSREGDIS----------
PVP01_0907600     YLHCAGG---YAKDLSGRQREGGVRTPLG-------------------------------------S------------ASSISRGGGIS----------
PVX_091015        YLHCAGG---YAKDLSGRQREGGVRTPLG-------------------------------------S------------ASSISRGGGIS----------
THRCLA_22740      VL--------------------------------------------------------------------------------------------------
THRCLA_04632      IL--------------------------------------------------------------------------------------------------
BESB_019990       FFG------TPLTRP--RQPAGAREAAH-------------VGRRQRESKREE-----------EATIG---------GDERARDGADAR----------
BN1204_042460     FFD------SPLHRP--RQLCRPGLYAGDRGVPRE-----------------------------ELK------------SSSADLPAKTGR---------
TGGT1_290225      FFD------APLSRP--RHLPSLVLHATDFVVAGDG---ESSASLSKNG---------------EPT------------DRSTATGAGTCRTQTKPEGDA
TGDOM2_290225     FFD------APLSRP--RHLPRLVLHATDFVVAGDG---ESSASLSKNG---------------EPT------------DRSTATGAGTCRTQTKPEGDA
BN1205_083850     FFD------APLSRP--RHLPRLVLHATDFVVAGDG---ESSASLSKNG---------------EPT------------DRSTATGAGTCRTQTKPEGDA
HHA_290225        FFD------APLCRP--RHLARPVLPLTNFAAAEDG---EVSGNPSKNAARPCMCATAPEPSDEEPT------------ERSAATGAGTWLTQTKSEGDA
ETH_00000110      HLSPGA---PPLGLPP-RHPMGAFSLSHREAAS-------------------------------SAGF------PHCEASGSPNSGDCSSRCGSV-----
ENH_0020460       HLSPGA---PHLGLPP-RHPLGAFSLSHREVP--------------------------------ASGF------PHCEASGSPNSGDCSSRCGSV-----
EMWEY_00023200    HLSPSP---PSWVPPPRRHPGGGPLQRQQQQQQQQQQLQHLGTPLQHSLRHDGDTGD----SSISPSI------SHTHSSSRSSSSSSSSRGGSSP----
Hs_RAF1           HLHVQ-----------------------------------------------------------------------------------------------
```

```
Pa_RAF1             HLHVQ-----------------------------------------------------------------------------------------------
Mm_RAF1             HLHVQ-----------------------------------------------------------------------------------------------
Rn_RAF1             HLHVQ-----------------------------------------------------------------------------------------------
Bt_RAF1             HLHVQ-----------------------------------------------------------------------------------------------
Gg_RAF1             HLHVQ-----------------------------------------------------------------------------------------------
Xl_RAF1             HLHVL-----------------------------------------------------------------------------------------------
Ce_RAF1             HIHVQE----------------------------------------------------------------------------------------------
GNI_162980          LFRQPQSRDMPWGPG--RNNECIIFWQRDHPDRG------------------------------SPRLL----------DREGGSGA-------------

                          2110      2120      2130      2140      2150      2160      2170      2180      2190      2200
                    ....|....|....|....|....|....|....|....|....|....|....|....|....|....|....|....|....|....|....|....|
PF3D7_1106800       YNKDY-------------NNKKKKE----------DEQHNIEHQDTF-IDLPEKSNISSDDNNSTD----ISQIQKENFH--------------------
PFIT_1107600        YNKDY-------------NNKKKKE----------DEQHNIEHQDTF-IDLPEKSNISSDDNNSTD----ISQIQKENFH--------------------
PPRFG01_1107500     YNKDY-------------NNKKKKE----------DEQHNIEHQDTF-IDLPEKSNISSDDNNSTD----ISQIQKENFH--------------------
PRCDC_1105300       -DKDY-------------ND-KKKE----------DEQHNIDHQDTF-VDLPEKSNISSDDNNSTD----ISQIQKENFH--------------------
PRG01_1104000       -DKDY-------------ND-KKKE----------DEQHNIDHQDTF-VDLPEKSNTSSDDNNSTD----ISQIQKENFH--------------------
PBILCG01_1104000    -NKDY-------------YN-KKKE----------DEHHNIDQQDTF-VDLPEKSNISSDDNNSTD----ISQIQKENFN--------------------
PBLACG01_1104300    -NKDY----------NNNNNNQKEEE--EDDNDDDEEEHNIDHQDTF-VDIPEKCSISSDDNNSTD----ISQMQKDNFH--------------------
PADL01_1105000      -KKDY-------------NNSNTEE---EVPN---EDQNNIDNQDTF-LDLSEKSNISSDDNNSTD----ISQIQKENFH--------------------
PGABG01_1103900     -NKDY-------------NNSNKEE---EDTN---EDQNNIDNQDTF-LDLSQESNISSDDDNSTD----ISQIQKENFN--------------------
PGSY75_1106800      -NKDY-------------NNSNKEE---EDTN---EDQNNIDNQDTF-LDLSQESNISSDDDNSTD----ISQIQKENFN--------------------
PGAL8A_00334100     --KVL-------------NDKK-------------------------------SIKNCNNDKNNSK----FIQIESENFN--------------------
PRELSG_0904000      -SKVL-------------NDKN-------------------------------NIKNSDSDKNNLK----FPEVGSKFFN--------------------
PocGH01_09013500    -NKHIGGKSKGGYVENCENGKNCES------SKNCESSKNCESSKN-----CESSKNCESSKNSEN----GKNSENEKFL--------------------
PYYM_0941700        ------------------NGKN-------------------------------KITKKNNLKDEQI----YSPSTSIKSL--------------------
PY02790             ------------------NGKN-------------------------------KITKKNNLKDEQI----YSPSTSIKSL--------------------
PY02791             ----------------------------------------------------------------------------------------------------
PBANKA_0940100      RNKLL-------EIKCKNNAKN-------------------------------KITEKNNLKDEQI----YSSSTSIKSL--------------------
PCHAS_0904200       ---------------------------------------------------------KHDTNNELA----YSSSASAKSL--------------------
PCHAS_090420        ---------------------------------------------------------KHDTNNELA----YSSSASAKSL--------------------
YYE_02751           ---------------------------------------------------------KHDTQNEQT----YSSSTSTKSF--------------------
YYG_01693           ---------------------------------------------------------KHDTKNEQT----YSSSTSNKSF--------------------
PmUG01_09016400     -SRSSGSSGSSGSSRSSGNSGNSGSS--GSSGRSGRSGSNASNAAN-----AANPNNVRADRNVYD----YSSPPNTASHKYDTSSDKGRKKMISTDGRE
C922_04039          ---------------------------------------------------AQSPADNSSTCDSSP----GYTYPSENPT--------------------
PcyM_0908600        ---------------------------------------------------AHSSTDDSSTCDSSP----GNTSPSENPT--------------------
PKNOH_S120122800    ---------------------------------------------------AKNSTDNSSSCDSCT----WNTSHSENPA--------------------
PKNH_0904400        ---------------------------------------------------AKNSTDNSSSCDSCT----WNTSHSENPA--------------------
PVP01_0907600       ---------------------------------------------------AHGSPGNSSTCDSSP----GGTSPSDNPT--------------------
PVX_091015          ---------------------------------------------------AHGSPGNSSTCDSSP----GGTSPSDNPT--------------------
THRCLA_22740        ----------------------------------------------------------------------------------------------------
THRCLA_04632        ----------------------------------------------------------------------------------------------------
BESB_019990         -RRRA----------------------AEETRRPLRRGGSAEDAGE-----REEASLASSGFTSIRELL--ETVEDERRRP-----MKGKRQL-------
BN1204_042460       -RRER--------------------------------GRETRGDVAE----REEITRSGSGRNTDED---------------------GRLTL-------
TGGT1_290225        CRRQTPDQGGSKTLQRQESEKDGRE--EDREREGKREGREARNRADEEARKNVEQGRERRGGKAEREEDEKRNVETERREG-----QWRRSCL-------
TGDOM2_290225       CRRQTPDQGGSETLQRQESEKDGRE--EDREREGKREGREARNRADEEARKNVEQGRERRGGKAEREEDEKRNVETERREG-----QWRRSCL-------
BN1205_083850       CRRQTLDQGGSETLQRQESEKNGRE--EHREREGKREGREGRDRADEEARKNVEQGRERRGGKAEREEDEKRNVETERREG-----QWRRSCL-------
HHA_290225          CRRQALDKGGSETLQRQDSDTREGE--EDREREEKREGREVRDHADEEAGTTVEQGRERRGGKAEREEDEKRTVETERREG-----DWGRSCL-------
ETH_00000110        --ERL---------------------HCLAPGAPESSGQSAGCPTA-----ASAPVESGSSQDAVG-----ASTERRWKQD-----EWGQK---------
ENH_0020460         --EQL---------------------HCLAPGAPESSGQSAGCATA-----ASAPVESGSSQDAVG-----TSTERRWKHD-----EWGQK---------
EMWEY_00023200      -FRPL---------------------MGASEGLPVPGGPSTGAPSS-----RGAPSVGPSSGGP------------------------------------
Hs_RAF1             ----------------------------------------------------------------------------------------------------
Pa_RAF1             ----------------------------------------------------------------------------------------------------
Mm_RAF1             ----------------------------------------------------------------------------------------------------
Rn_RAF1             ----------------------------------------------------------------------------------------------------
Bt_RAF1             ----------------------------------------------------------------------------------------------------
Gg_RAF1             ----------------------------------------------------------------------------------------------------
Xl_RAF1             ----------------------------------------------------------------------------------------------------
Ce_RAF1             ----------------------------------------------------------------------------------------------------
GNI_162980          ----------------------------------EPGGGTAPSEGA-----GSSPGVVGNGVAAGSGVGSRIGTGRRRRSGYLPSLFWGPR---------

                          2210      2220      2230      2240      2250      2260      2270      2280      2290      2300
                    ....|....|....|....|....|....|....|....|....|....|....|....|....|....|....|....|....|....|....|....|
PF3D7_1106800       ------------------FLNK-KIEENKNIIYDDHTSTLSDHS----IHNINKSYDNVYKNKMN-IFHYQH---------------NVLC---------
PFIT_1107600        ------------------FLNK-KIEENKNIIYDDHTSTLSDHS----IHNINKSYDNVYKNKMN-IFHYQH---------------NVLC---------
PPRFG01_1107500     ------------------FLNK-KIEENKNIIYDDHTSTLSDHS----IHNINKSYDNVYKNKMN-IFHYQH---------------NVLC---------
PRCDC_1105300       ------------------FINK-KIEEKKGIIYDDHTSTLSDHS----IHNINKSYYNVYKKKMN-IFHYEH---------------NVLC---------
PRG01_1104000       ------------------FINK-KIEEKKGIIYDDHTSTLSDHS----IHNINKSYYNVYKKKMN-IFHYEH---------------NVLC---------
PBILCG01_1104000    ------------------FLNNKKMEQNKDIIFDDHTSTLSDYS----IDNINKSYYNLYKKKMN-IFNCEH---------------NVLC---------
PBLACG01_1104300    ------------------LLNN-KIEENKDINCDDLTSTLSDQS----IDNINKSFYNVYKKKMN-IFNYEH---------------NVLC---------
PADL01_1105000      ------------------YLNN-KIEENKDFNYDDNTSTLSDHS----INNMKQSYYDVYKKKIN-IFNYEH---------------NVLC---------
PGABG01_1103900     ------------------YLNN-KIEENKNFNYHDNTSTLSDHS----INNMKQSYYNVYKKKIN-IFNYEH---------------NVLC---------
PGSY75_1106800      ------------------YLNN-KIEENKNFNYHDNTSTLSDHS----INNMKQSYYNVYKKKIN-IFNYEH---------------NVLC---------
PGAL8A_00334100     ---------------------------------NESESSLSDNTDFE-KNKRDNKYFKI--NKFN-LFNYEN---------------NVLC---------
PRELSG_0904000      ---------------------------------NESESSLSNNTYFE-KNNRDNKYYKK--NKFN-LFNYEN---------------NVLC---------
PocGH01_09013500    ------------------------RSYSLDSFINSTDSSLSDDFSFE-LQNKHCNRLTT--DNFN-LYNCEN---------------NVLC---------
PYYM_0941700        ---------------------------------DTNSSNM-NNKKL--KNKNSNEKRYI--NKIDSMFRNKN---------------NILC---------
PY02790             ---------------------------------DTNSSNM-NNKKL--KNKNSNEKRYI--NKIDSMLRNKN---------------NILC---------
PY02791             ----------------------------------------------------------------------------------------------------
PBANKA_0940100      ---------------------------------DTSSSNM-NNTKL--KNINFNKNRYI--NKIHSMFRNKN---------------NILC---------
PCHAS_0904200       ---------------------------------DTNSSNM-NNTEL--KNRNNSKYRYI--NKINSMFRNKN---------------NILC---------
PCHAS_090420        ---------------------------------DTNSSNM-NNTEL--KNRNNSKYRYI--NKINSMFRNKN---------------NILC---------
YYE_02751           ---------------------------------DTSSSNM-NNIKV--KNKNNSKYRYI--NKINSTFRNKN---------------NILC---------
```

```
YYG_01693            ---------------------------------DTSSSNM-NNTEL--KNRNNNKYRYI--NKINSTFRNKN---------------NILC---------
PmUG01_09016400      KNCVMNQTHLTCRVNRIKRTNRKSSKKNGDNYSSATENSLSEELRFEPASNRTSEGLRIQNNKFN-IFNYEH---------------NVLC---------
C922_04039           --------------------------------FESYSGRITTRMR----------------NKYN-SFKSEN---------------DLFC---------
PcyM_0908600         --------------------------------FESHGGRITTRMR----------------NKNN-SFKSEN---------------DLFC---------
PKNOH_S120122800     --------------------------------SESHSGRITIGVR----------------NKYN-PFKGDN---------------DLFC---------
PKNH_0904400         --------------------------------SESHSGRITIGVR----------------NKYN-PFKGDN---------------DLFC---------
PVP01_0907600        --------------------------------FESHSGRIATRMR----------------NKCN-SFVLEN---------------DLFC---------
PVX_091015           --------------------------------FESHSGRISTRMR----------------NKCN-SFVLEN---------------DLFC---------
THRCLA_22740         -------------------------------------------------------------AHSN-----------------------------------
THRCLA_04632         -------------------------------------------------------------SSTN-----------------------------------
BESB_019990          ---------------SCEAVHAQSTGAEPPDTGARHKQGFSPHLQPAHSHALSSPSSGAGGEADA-RLSSASSASLASLSGSAFPALSPLCARPLALR--
BN1204_042460        -----------------AFARANECSCETRPPCHPCETPPRPGESVASLPATLAP------EDIS-RLSASG--------FSPFSP-SPLCVLPLSLRYA
TGGT1_290225         ---------------HNDLAHANRSACD------TAASSLTPARSCTDIPAVSLPLQ---PRETP-QCST------------VFPPCSPLCVLPLSLRST
TGDOM2_290225        ---------------HNDLAHANRSACD------TAASSLTPARSCTDIPAVSLPLQ---PRETP-QCST------------VFPPCSPLCVLPLSLRST
BN1205_083850        ---------------HTDLAHANRSACD------TAASSLTPARSCTDIPAVSLPLQ---PRETP-QCST------------VFPPCSPLCVLPLSLRST
HHA_290225           ---------------HNGLAHAHRSACD------TAASSLPPALTCTTLPAVSLPLP---PRATP-QRSA------------VFPPCSPLCVLPLSLRST
ETH_00000110         --------------RHFRAPNA----------DPSGPQVLRP-QN----------------EEDP-----------------FLLP-PACCVVPLVLAGA
ENH_0020460          --------------RHSRAPNG----------GPSGVPVLGPSQN----------------EEDP-----------------FLLP-PACCVVPLVLAGA
EMWEY_00023200       ---------------------------------PSPNNSLRPGGTATG-----------GPQEDN-----------------LLMG-PPNC---------
Hs_RAF1              ----------------------------------------------------------------------------------------------------
Pa_RAF1              ----------------------------------------------------------------------------------------------------
Mm_RAF1              ----------------------------------------------------------------------------------------------------
Rn_RAF1              ----------------------------------------------------------------------------------------------------
Bt_RAF1              ----------------------------------------------------------------------------------------------------
Gg_RAF1              ----------------------------------------------------------------------------------------------------
Xl_RAF1              ----------------------------------------------------------------------------------------------------
Ce_RAF1              ----------------------------------------------------------------------------------------------------
GNI_162980           ---------------------------------TSATSRTVPIQNI------------CSGASMP----------------------LTFC---------

                              2310      2320      2330      2340      2350      2360      2370      2380      2390      2400
                     ....|....|....|....|....|....|....|....|....|....|....|....|....|....|....|....|....|....|....|....|
PF3D7_1106800        ---GAYDNNDNNINDNDIYCNNIYDNN--------------------INDNHIYCNNIN-----------DNHIYCNNIYD--------HHKNTSLNSKE
PFIT_1107600         ---GAYDNNDNNINDNDIYCNNIYDNN--------------------INDNHIYCNNIN-----------DNHIYCNNIYD--------HHKNTSLNSKE
PPRFG01_1107500      ---GAYDNNDNN-----------------------------------INDNDIYCNNIN-----------DNDIHCNNIYD--------HHKNTSLNSKE
PRCDC_1105300        ---GAYDNNDDNINDDNINDDNINDDNINDDNINDNHINDNHINDNHINDNHINDNHIN-----------DNHIYSNNIYD--------NHKNTSLNSKE
PRG01_1104000        ---GAYDNNDDNINDDNINDDNINDDNINDDNINDDNINDDNINDDNINDNHINDNNIN-----------DNHINDNHIND--------NHKNTSLNSKE
PBILCG01_1104000     ---GAYDNNDNNMYDNNMYDNNMYDNNMYDNNMYENNIYENNKYENNIYENNIYENNIY-ENNIYENNIYENNIYENNIYD--------HHKNTSLNSKE
PBLACG01_1104300     ---GAYDNNDNNIYDKNIYDNNIYDNNIYDNNIYDNNIYDNNIYDNNIYDNNIYDNNICNNNNINDKNIYDNNIYENNIYD--------NHKNTSLNSKE
PADL01_1105000       ---GAYDNNDNNM----------YDNN--------------------MYDNNMYDNNMN-----------HDNMYDNNFYD--------HHKNTSINSKE
PGABG01_1103900      ---GAYDNNDNNMNHD----------N--------------------MYDNNMYDNNMN-----------NDNMYDNNIYD--------HHKNTSINSKQ
PGSY75_1106800       ---GAYDNNDNNMNHDNMYDNNMYDNN--------------------MYDNNMYDNNMH-----------DNNMYDNNIYD--------HHKNTSINSKQ
PGAL8A_00334100      ---G------------------------------------------------IYADELK-----------NNNEIKSSSSS--------DTDITIFNASI
PRELSG_0904000       ---G------------------------------------------------VYADELE-----------GNNETQSFSSN--------DSDITVFNANS
PocGH01_09013500     ---G------------------------------------------------AYVDQLG-----------KASRTPVTPSA--------TSNSITTRGNA
PYYM_0941700         ---G----------------------------------------------NYYYLFRKK-----------KNNMSISNEHK--------DSDRTSFTNES
PY02790              ---G----------------------------------------------NYYYLFRKK-----------KNNMSISNEHK--------DSDRTSFTNES
PY02791              ----------------------------------------------------------------------------------------------------
PBANKA_0940100       ---G----------------------------------------------NYYYLFRKK-----------KNNISISHEHK--------NSDRTIFTNES
PCHAS_0904200        ---G----------------------------------------------NYHYLFRKK-----------SGNMSISQEHK--------NSDRISFTNES
PCHAS_090420         ---G----------------------------------------------NYHYLFRKK-----------SGNMSISQEHK--------NSDRISFTNES
YYE_02751            ---G----------------------------------------------NYHYLFQKK-----------KNNIPISHEHK--------NSDRISFTNES
YYG_01693            ---G----------------------------------------------NYHYLFRKK-----------KNNMSISHEHK--------NSDRISFTNES
PmUG01_09016400      ---G------------------------------------------------AYADQIK-----------YRKEIKPRAAN--------DTDMTEHNSSS
C922_04039           ---G------------------------------------------------VFAQA--------------------------------NGGPTSGRSRS
PcyM_0908600         ---G------------------------------------------------VYA---------------------EEAEE--------HSGPTSGENPP
PKNOH_S120122800     ---G------------------------------------------------AYA---------------------EEETE--------HVVPTPRKKLQ
PKNH_0904400         ---G------------------------------------------------AYA---------------------EEETE--------HVVPTPRKKLQ
PVP01_0907600        ---G------------------------------------------------VYAEE------------GEEGKEAEEAEQ--------HGGATSGESPP
PVX_091015           ---G------------------------------------------------VCAEE------------GEEGKEAEEAEQ--------HGGATSGESPP
THRCLA_22740         ----------------------------------------------------------------------------------------------------
THRCLA_04632         ----------------------------------------------------------------------------------------------------
BESB_019990          ---G----SSGRVASSPSGAPPLVSSA-------------AQERLARPVPEVFVLSDDE-----------AESANAHHEEG--------RTEDFLFRHLS
BN1204_042460        R------VTPSLSGSAPSRCQSVASASPLVPSSEAERLGRSQAPHASHVIQATHDRPVK-----------SRAACESSASPG--DSTKTADARAAERDKP
TGGT1_290225         SRIT---LSSSSSSSSSATAPPVSSELPRSSEGGKATFARLQEPQ-GNAAEALGGRSVE-----------SRSACALSSTT----------ARGVCRDTE
TGDOM2_290225        SRIT---PSSSSSSSSSATAPPVSSELPRSSEGGKATFARLQEPQ-GNAAEALGGRSVE-----------SRSACALSSTT----------ARGVCRDTD
BN1205_083850        SRMT---LSSSSSSSSSATAPPVSSELPRSSEGGKATFARLQEPQ-GNAAEALGGRSVE-----------SRSACALSSTT----------ARGVCRDTE
HHA_290225           SRTTSPSSSSASSSSSSARVPSVSSERPRSAEAGKATFARLQEPH-ENAAEALGGRSAE-----------SRSACVPPSTT----------ARGVCRDTE
ETH_00000110         L-EGPQYTDKSPTVSWSSPRGGVVEGSPRE--GAHAGGCYKSAPK-RVLHRTRHSAGVS-----------QGSASVGSPQCL-------HKSSGELNYRE
ENH_0020460          L-EGPQYTDKSPTVSWSSPRGGAVEGSPRE--GAHAGGGYKSAPK-RDLHRARHSAGVS-----------QDSASVGSPQCA-------HKGSGELNYRE
EMWEY_00023200       ---------PSLSVDKESQEGGLWSSSPW-----------GAPPPIKPLWGALHIED------------NRGAPSVPPPSCLVPIALAEAIERLHARGRS
Hs_RAF1              ----------------------------------------------------------------------------------------------------
Pa_RAF1              ----------------------------------------------------------------------------------------------------
Mm_RAF1              ----------------------------------------------------------------------------------------------------
Rn_RAF1              ----------------------------------------------------------------------------------------------------
Bt_RAF1              ----------------------------------------------------------------------------------------------------
Gg_RAF1              ----------------------------------------------------------------------------------------------------
Xl_RAF1              ----------------------------------------------------------------------------------------------------
Ce_RAF1              ----------------------------------------------------------------------------------------------------
GNI_162980           ------------------------EADPW-----------------REDLSVLAAGGLS-----------TANACCHRDYHL------DLLIRLLLKKKT

                              2410      2420      2430      2440      2450      2460      2470      2480      2490      2500
                     ....|....|....|....|....|....|....|....|....|....|....|....|....|....|....|....|....|....|....|....|
```

```
PF3D7_1106800        ---------------QNTDHNIEQI----------------------NECNKYASETKYNIKKSNLKNNI----------ISHKNFQKCNQIQMNQPYTF
PFIT_1107600         ---------------QNTDHNIEQI----------------------NECNKYASETKYNIKKSNLKNNI----------ISHKNFQKCNQIQMNQPYTF
PPRFG01_1107500      ---------------QNTDHNIEQI----------------------NECNKYASETKYNIKKSNLKNNI----------ISHKNFQKCNQIQMNQPYTF
PRCDC_1105300        ---------------QNIDHNIEQI----------------------NECNKYASETKYTIKKSNLKNNI----------ISHKNFQTCNQIQMNQPYTF
PRG01_1104000        ---------------QNIDHNIEQI----------------------NECNKYASETKYTIKKSNLKNNI----------ISHKNFQTCNQIQMNQPYTF
PBILCG01_1104000     ---------------QNTDPNNEQI----------------------NESTNYSSQTKYNIKKSNLKNNI----------ISHKNIQKCNQIQMNQPYTF
PBLACG01_1104300     ---------------QNTHQSNEQI----------------------NECISYVSQTKYNIKKSNLKKNI----------ISHKKLQKCNQIQMNQPYTF
PADL01_1105000       ----QH-------INQNLQQDTEQN----------------------NECTSYASQIKYNIKKSNFKNNI----------ISHKKIQKCNEIQINQPYTF
PGABG01_1103900      QNIHQN-------IHQNIQQDTEQN----------------------KECSSYASQIKYNIKKSNFKNNI----------ISHKNIKKCNEIQINQPYTF
PGSY75_1106800       QNIHQN-------IHQNIQQDTEQN----------------------NECSSYASQIKYNIKKSNFKNNI----------ISHKNIQKCNEIQINQPYTF
PGAL8A_00334100      ---------------DKNYSLYE-------------------------NNNNFKKVNQKDINKNN---------------KKLGKLENKKKIQLNDPYSF
PRELSG_0904000       ---------------EKEYPLSE-------------------------INNNFKKINQRDINKNN---------------KKFYKLQNKKKIQLNDPYSF
PocGH01_09013500     ---------------TTLVIPPSPV----------------------VQFSAHAKASNGNIQHMDMHVGSSGW-------RARNKQKLEKEIHFNHPYAF
PYYM_0941700         ---------------QKLLT---------------------------------NKIYKKKINK---------------------KLNFKAKIKINRPYSF
PY02790              ---------------QKLLT---------------------------------NKIYKKKINK---------------------KLNFKAKIKINRPYSF
PY02791              ----------------------------------------------------------------------------------------------------
PBANKA_0940100       ---------------QNLLK---------------------------------NKISQKKINK---------------------KLNFKAKIKINRPYAF
PCHAS_0904200        ---------------QNLLT---------------------------------NKKYKKKINK---------------------KLGFKEKIKINRPYAF
PCHAS_090420         ---------------QNLLT---------------------------------NKKYKKKINK---------------------KLGFKEKIKINRPYAF
YYE_02751            ---------------QNLLT---------------------------------NKTYKKKINK---------------------KLDFKVKIKINRPYVF
YYG_01693            ---------------QNLLT---------------------------------NKTYKKKINK---------------------KLDFKAKIKINRPYAF
PmUG01_09016400      ---------------EYDEASIEKKASNIATNRDSC----NRKSNRRYKRDKPKKTNPNDISKTSKKVGS--------------KLEMKKSIHLDHPCSF
C922_04039           --IAA----------NNLNSSVKSPS---------------------KERNINQSKTKAHIEEINKKNGNKK-------KKLHTKLFLQDQIGLHEPYAF
PcyM_0908600         --IPE----------NNSNVLVKSSS---------------------KEWNINESKTKAHIEEVNKKSGKKK-------KKLHTKLFLQNQVRLHEPYAF
PKNOH_S120122800     --IYT----------NNYNASGRSPS---------------------NEWSICQKKTNSHIEEINKKGGKKK-------KKLHTKLFLQDQIQLHEPYAF
PKNH_0904400         --IYT----------NNYNASGRSPS---------------------NEWSICQKKTNSHIEEINKKGGKKK-------KKLHTKLFLQDQIQLHEPYAF
PVP01_0907600        --LPA----------NKLNASVKSPP---------------------KEWNTHQGKSKAHIEKINKKRGKKK-------KKLHTKLFLQDQIQLHEPYAF
PVX_091015           --LPA----------NKLNASVKSPP---------------------KEWNTHQGKSKAHIEKINKKRGKKK-------KKLHTKLFLQDQIQLHEPYAF
THRCLA_22740         ------------------------------------------------------------------------------------------NVKLPWNHHF
THRCLA_04632         ------------------------------------------------------------------------------------------SVQLTWNHHF
BESB_019990          -V-------PRVSDAASEAGDGESCTGSHSALR------------------THREATTAEGEDPWRREGDS-----------------CEPLLFSLPRPP
BN1204_042460        RGLAARDELVRA-SSRSLASSGRWLTPRRGSHRLSALP---RREGWIREPAHPSSQHKRETDEREPRERTE-------ERGESGGSTCVQDGLQDGSASF
TGGT1_290225         DALGAAQGEIS----FSSSRPARWLLPGRVSRRFPAWPGSEERRSRIARRSPNEGETTREQGRELEREGERGTTEQGRQHGREQGAMHAEDRRETTPASF
TGDOM2_290225        DALGAAQGEIS--SAFSSSRPARWLLPGRVSRRFLAWPGSEERRSRIARRSPNEGETTREQGRELEREGERGTTEQGRQHGREQGAMHAEDRRETTPASF
BN1205_083850        DALGAAQGEIS--SAFSSSRPARWLLPGRVSRRFPAWPGSEERRSRIARRSPNEGETTREQGRELEREGERGTTEQGRQHGREQGAMHAEDRRETTPASF
HHA_290225           DASGVVEGEPF--SAFSSARHARWLLPRRVSRRFPAWPGREERRSRTARRSPNERETAREEEGEVEREGERAATEL----GREQGAMRAEDRRETTPGSF
ETH_00000110         --------GPP--ASSGLTASSRSGALSRGPSW-------------------------EELGGSQDMGGIQGP-----------------SSTQSAPAGI
ENH_0020460          --------GPR--APSGLPSSSRSRALSRGPSW-------------------------EELGGSQDMGGIQGH-----------------SSSQSAPAGI
EMWEY_00023200       --LPSFLGGPR--RAGGAHRNRRGAPSSRASS-----------------------IKKSEEDGGAPLSGSWGP---------------ASPLNNSTSQGP
Hs_RAF1              ----------------------------------------------------------------------------------------------------
Pa_RAF1              ----------------------------------------------------------------------------------------------------
Mm_RAF1              ----------------------------------------------------------------------------------------------------
Rn_RAF1              ----------------------------------------------------------------------------------------------------
Bt_RAF1              ----------------------------------------------------------------------------------------------------
Gg_RAF1              ----------------------------------------------------------------------------------------------------
Xl_RAF1              ----------------------------------------------------------------------------------------------------
Ce_RAF1              ----------------------------------------------------------------------------------------------------
GNI_162980           -PLPP----PTVPLVPSVKPSMGFCV------------------------SPVPILTKPFLGPRQTAPGHG-------------------QAAPPGPSGP

                              2510      2520      2530      2540      2550      2560      2570      2580      2590      2600
                     ....|....|....|....|....|....|....|....|....|....|....|....|....|....|....|....|....|....|....|....|
PF3D7_1106800        PPYQKELSSYLK-NEKIKRKRKVLFSYLKTHIHFNSQQ---INDQ---------HNRLSVQKIMKIIT-DVTLACTYLEKEK---------MSPINLKPT
PFIT_1107600         PPYQKELSSYLK-NEKIKRKRKVLFSYLKTHIHFNSQQ---INDQ---------HNRLSVQKIMKIIT-DVTLACTYLEKEK---------MSPINLKPT
PPRFG01_1107500      PPYQKELSSYLK-NEKTKRKRKVLFSYLKTHIHFNSQQ---INDQ---------HNRLSVQKIMKIIT-DVTLACTYLEKEK---------MSPINLKPT
PRCDC_1105300        PPYQKELSAYLK-NEKTKMKRKVLFSYLKTHIHFNSQQ---INDQ---------HNRLSVQKIMKIIT-DVTLACTYLEKEK---------MSPINLKPT
PRG01_1104000        PPYQKELSAYLK-NEKTKMKRKVLFSYLKTHIHFNSQQ---INDQ---------HNRLSVQKIMKIIT-DVTLACTYLEKEK---------MSPINLKPT
PBILCG01_1104000     PPYQKELSSYLK-NQKAKMKRKVLFSYLKTHTHFNSQQ---INDQ---------HNILSVQKIMKIII-DVTLACTYLEKEK---------ISPINLKPT
PBLACG01_1104300     PPYQKELSSYLK-NEKAKRKRKVLFSYIKTHIHFNSQQ---INDQ---------HNRLSVQKIMKIIT-DVTLACTYLEKEK---------MSPINLKPT
PADL01_1105000       PPYQKELSSYLK-NEKEKMKRKVLFSYLKTHVHFNSQQ---VNDQ---------NDRLSVQKIMKIIT-DVTLACTYLEKEK---------MSPINLKPT
PGABG01_1103900      PPYQKELSSYLK-NEKAKMKRKVLFSYLKTHAHFNSQQ---VNDQ---------NDRLSVQKIMKIIT-DVTLACTYLEKEK---------MSPINLKPT
PGSY75_1106800       PPYQKELSSYLK-NEKAKMKRKVLFSYLKTHAHFNSQQ---VNDQ---------NDRLSVQKIMKIIT-DVTLACTYLEKEK---------MSPINLKPT
PGAL8A_00334100      PPYQRELFSYLK-NERKKKKKKFLFSYLKTQIYFHSQK---SNFR---------NNRLSVQRIMKIIT-DVTLACSYLEKHK---------LYPLNLKPT
PRELSG_0904000       PPYQRELLSYLK-KEKKKKKKKVLFSYLKTQIYFHSQK---SNFR---------NNRLSVQRIMKIIT-DVTLACSYLEKQK---------LYPLNLKPT
PocGH01_09013500     PPFHRELLLYVK-RKKAKQTKKVLFSYLKRKSCLLSRKRYLMSSRQCTPSYANVQNRLSIQRIMKIIT-DITLACSYLEKQK---------LGPLNLKPT
PYYM_0941700         PPLQEDFNFYL---EKKKKKKKILFSYLKTHSYFKSKK---CDTR---------KNKLSDHQMMKIII-DITLACSYLEKQK---------IRWLNLKPT
PY02790              PPLQEDFNFYL---EKKKKKKKILFSYLKTHSYFKSKK---CDTR---------KNKLSDHQMMKII---------------------------------
PY02791              ----------------------------------------------------------------------ITLACSYLEKQK---------IRWLNLKPT
PBANKA_0940100       PPLQEDFNFYL---EKKKKKKKILFSYLKTHSYFKSKK---CDSR---------KNKLSDHQIMKIIM-DITLACSYLEKQK---------VRWINLKPT
PCHAS_0904200        PPLQEDLNFYF---EKKKQKNKILFSYLKTHSYFKSKK---CDDR---------KSKLSDQQIMKIII-GITLGCSYLEKQK---------VQWINLKPT
PCHAS_090420         PPLQEDLNFYF---EKKKQKNKILFSYLKTHSYFKSKK---CDDR---------KSKLSDQQIMKIII-GITLGCSYLEKQK---------VQWINLKPT
YYE_02751            PPLQEDLNFYF---EKKKQKNKILFSYLKTHSYYKSKK---CDDR---------KNKLSDQQIMKIII-GITLGCSYLEKQK---------IQCLNLKPT
YYG_01693            PPLQEDLNFYF---EKKKQKNKILFSYLKTHSYFKSKK---CDDR---------KNKLSDQQIMKIII-GITLGCSYLEKQK---------VQWLNLKPT
PmUG01_09016400      PEFQKELFSYLK--RKKKKKKKVLFSYLKTQIYINSRK---NTFR---------NNRLSVQRIMKIIT-DITLACSYLEKQK---------LYPLNLKPT
C922_04039           PPFEKELCLYIK-RQKKKKKKKILFSYPQAKLHFNLPQ---SALKA--------DRRLSIQRILKITT-DVTLACSYLEKHL---------SHHLNLKPT
PcyM_0908600         PPFEKELSLYVK-KQKKKKKKKILFTYPQTKLHFNLPE---SPLKA--------DRRLSVQRILKITT-DVTLACSYLEKHL---------SHPLNLKPT
PKNOH_S120122800     PPFDKELSFYVK-KKKKKKKKYILFTYPQPKLHFNLPG---NALKK--------DRRLSVQRILKITTRDVTLACSYLEKHL---------SHPLNLKPT
PKNH_0904400         PPFDKELSFYVK-KKKKKKKKYILFTYPQPKLHFNLPG---NALKK--------DRRLSVQRILKITT-DVTLACSYLEKHL---------SHPLNLKPT
PVP01_0907600        PPFEKELSLYVK-KQKKKKKKKILFSYPQTQLHLNLPE---SPLKE--------DRRLSVQRILKITT-DVTLACSYLEKHL---------SHPLNLKPT
PVX_091015           PPFEKELSLYVK-KQKKKKKKKILFSYPQTQLHLNLPE---SPLKE--------DRRLSVQRILKITT-DVTLACSYLEKHL---------SHPMNLKPT
THRCLA_22740         ---------------------------------------------------------------LDMAI-SICRAMSYLHGRN---------HIHKDLSSS
THRCLA_04632         ---------------------------------------------------------------LDMAI-SICRAMSYLHGRN---------HIHKDLSSS
BESB_019990          PKITREETQAEPPDRTRLRAKSRDFERRRLREDWKKRA---DAAA---------AFRMDIHSALRLAR-GIALGCAYLQRKG---------LYHHRLHPG
```

```
BN1204_042460      P--DGALRPTPGPARTALQAKSAEFERLRLQHDWRRRS---ASST---------RFRLSIHSALRLAR-GIALGCAYLQRKG---------ISRLELRPG
TGGT1_290225       P--NAELRSAALP-RTGLKAKSAEFERRRLEHDWRRRT---AAAA---------RFRLDIHSSLRLAR-GIALGCAYLQRKG---------ISYLQLRPG
TGDOM2_290225      P--NAELRSAALP-RTGLKAKSAEFERRRLEHDWRRRT---ATAA---------RFRLDIHSSLRLAR-GIALGCAYLQRKG---------ISYLQLRPG
BN1205_083850      P--NAELRSAALR-RTGLKAKSAEFERRRLEHDWRRRT---AAAA---------RFRLDIHSSLRLAR-GIALGCAYLQRKG---------ISYLQLRPG
HHA_290225         P--NAELRSAALP-RTGLKAKSAEFERRRLEHDWRRRT---AAAA---------RFRLDIHSSLRLAR-GIALGCAYLQRKG---------ISYLQLRPG
ETH_00000110       PLGALAKMCGVPVAVAGLRAPCVGHKDHRKGAGGPHSP-----------TR--GPCLPHLLDALNLAR-GIALGCAYLQRQQ---------VYHLQLSPS
ENH_0020460        PLGALAKMCGVPFAVAGLRAPCVGHTDHRKGAGGLHSP-----------TR--GPCLPHLLDALNLAR-GIALGCAYLQRQQ---------VYHLQLSPS
EMWEY_00023200     PLDENAPSGGPLPPAAGFQQQQQQQQQQQQQQQQQQQQ-------------------------------QQQQQQQQQQQQQQQ------QQQRPEVSPS
Hs_RAF1            ------------------------------------------------------ETKFQMFQLIDIAR-QTAQGMDYLHAKN---------IIHRDMKSN
Pa_RAF1            ------------------------------------------------------ETKFQMFQLIDIAR-QTAQGMDYLHAKN---------IIHRDMKSN
Mm_RAF1            ------------------------------------------------------ETKFQMFQLIDIAR-QTAQGMDYLHAKN---------IIHRDMKSN
Rn_RAF1            ------------------------------------------------------ETKFQMFQLIDIAR-QTAQGMDYLHAKN---------IIHRDMKSN
Bt_RAF1            ------------------------------------------------------ETKFQMFQLIDIAR-QTAQGMDYLHAKN---------IIHRDMKSN
Gg_RAF1            ------------------------------------------------------ETKFQMFQLIDIAR-QTAQGMDYLHAKN---------IIHRDMKSN
Xl_RAF1            ------------------------------------------------------DTKFQMFQLIDIAR-QTAQGMDYLHAKN---------IIHRDMKSN
Ce_RAF1            -----------------------------------------------------PRVEFEMGAIIDILK-QVSLGMNYLHSKN---------IIHRDLKTN
GNI_162980         PRQAAPLGQAP--GRLNTGSQQVLNEHRRIKSLPPAAQ---APAK----TSV-ASLNLDTCDIMLILK-YTLQGLIYLQRTAGRDDPQAGVSCHGNIKPS

                            2610      2620      2630      2640      2650      2660      2670      2680      2690      2700
                   ....|....|....|....|....|....|....|....|....|....|....|....|....|....|....|....|....|....|....|....|
PF3D7_1106800      NILLDESL-NAKISDFGISKIENCLDMNIDY---------------------------------------------------------------------
PFIT_1107600       NILLDESL-NAKISDFGISKIENCLDMNIDY---------------------------------------------------------------------
PPRFG01_1107500    NILLDESL-NAKISDFGISKIENCLDMNIDY---------------------------------------------------------------------
PRCDC_1105300      NILLDESL-NAKISDFGISKIEKCLDMNIDY---------------------------------------------------------------------
PRG01_1104000      NILLDESL-NAKISDFGISKIEECLDMNIDY---------------------------------------------------------------------
PBILCG01_1104000   NILLDESL-NAKISDFGISQIEECLDMNIDY---------------------------------------------------------------------
PBLACG01_1104300   NVLLDESL-NAKISDFGISQIEECLDMNIDY---------------------------------------------------------------------
PADL01_1105000     NILLDDSL-NAKISDFGISQIEECLDMNIDY---------------------------------------------------------------------
PGABG01_1103900    NILLDESL-NAKISDFGISQIEECLDMNIDY---------------------------------------------------------------------
PGSY75_1106800     NILLDESL-NAKISDFGISQIEECLDMNIDY---------------------------------------------------------------------
PGAL8A_00334100    NILLDESL-NAKISDFGIHQIEKCLDTNIDY---------------------------------------------------------------------
PRELSG_0904000     NILLDESL-NAKIADFGIQQIEKCLDTNIDY---------------------------------------------------------------------
PocGH01_09013500   NVLLDESL-NAKICDFGIGEIEKCLDTNVDY---------------------------------------------------------------------
PYYM_0941700       NILLDESL-NAKISDFGIKEIEECLDINIDY---------------------------------------------------------------------
PY02790            ----------------------------------------------------------------------------------------------------
PY02791            NILLDESL-NAKISDFGIKEIEECLDINIDY---------------------------------------------------------------------
PBANKA_0940100     NILLDGSL-NAKISDFGIKEIEQCLDINIDY---------------------------------------------------------------------
PCHAS_0904200      NILLDESL-NAKISDFGIKEIEQCLDTNIDY---------------------------------------------------------------------
PCHAS_090420       NILLDESL-NAKISDFGIKEIEQCLDTNIDY---------------------------------------------------------------------
YYE_02751          NILLDESL-NAKISDFGIKEIEQCLDTNINY---------------------------------------------------------------------
YYG_01693          NILLDESL-NAKISDFGIKEIEQCLDTNIDY---------------------------------------------------------------------
PmUG01_09016400    NVLLDEFL-NAKVSDFGISKIEKCLDTNIDY---------------------------------------------------------------------
C922_04039         NILLDEAL-NAKITDFGICEIEKCLDTNVDH---------------------------------------------------------------------
PcyM_0908600       NILLDEAL-NAKITDFGICEIEKCLDTNIDH---------------------------------------------------------------------
PKNOH_S120122800   NILLDEAL-NAKITDFGICEIEKCLDTNIDH---------------------------------------------------------------------
PKNH_0904400       NILLDEAL-NAKITDFGICEIEKCLDTNIDH---------------------------------------------------------------------
PVP01_0907600      NILLDEAL-NAKITDFGICEIEKCLDTHVDH---------------------------------------------------------------------
PVX_091015         NILLDEAL-NAKITDFGICEIEKCLDTHVDH---------------------------------------------------------------------
THRCLA_22740       SIFV--GL-NYRSTKVGW----------------------------------------------------------------------------------
THRCLA_04632       SIFV--GL-NYRSTKVGW----------------------------------------------------------------------------------
BESB_019990        KILVDEAL-NAKLIGSDSETLRQWCVAFAAASLRGPPPACASGGDPVTAAAVGSYGGCPPLPLSSSPLPLVARPTALSAPLPWASSPQSLARLPG-PIPA
BN1204_042460      NILVDDAM-NAKLTGLAVKSVHQWCCALMHAC---------AAGDA--AALSGSFA---------SVFPPFLDCLRLGPGASTPSVRKSQLSLPC-PSSP
TGGT1_290225       KVLVDESM-NAQLTGLAATTVHEWCRAWLQA----------DAGDG----VSGIFA--------SSTVPLFCESLRFERGGAT-------LSLPS-PSSP
TGDOM2_290225      KVLVDESM-NAQLTGLAATTVHEWCRAWLQA----------DAGDG----VSGIFA--------SSTVPLFCESLRFERGGAT-------LSLPS-PSSP
BN1205_083850      KVLVDESM-NAQLTGLAATTVHEWCRVWLQA----------DAGDG----VSGIFA--------SSTVPLFCESLRFERGGAT-------LSLPS-PSSP
HHA_290225         KVLVDESM-NAQLTGLAVTAVHEWSRAWLQA----------DAGDG----VSGTFT--------SSTGPPLCDSLRFELGDPT-------LSLPS-PSSP
ETH_00000110       KVLLDGAL-APKLAGFGIAEIESLLAQNVRSCI--------------------------------------------------------QQALEG-PLMG
ENH_0020460        KVLLDGAL-APKLAGFGIAEIESLLAQNVRSCI--------------------------------------------------------QQALEG-PLMG
EMWEY_00023200     IHFSLESL--ARLC--GIAEIGRRLALNVTECL--------------------------------------------------------QRCLQGAPRGA
Hs_RAF1            NIFLHEGL-TVKIGDFGLATVKSRWS--------------------------------------------------------------------------
Pa_RAF1            NIFLHEGL-TVKIGDFGLATVKSRWS--------------------------------------------------------------------------
Mm_RAF1            NIFLHEGL-TVKIGDFGLATVKSRWS--------------------------------------------------------------------------
Rn_RAF1            NIFLHEGL-TVKIGDFGLATVKSRWS--------------------------------------------------------------------------
Bt_RAF1            NIFLHEGL-TVKIGDFGLATVKSRWS--------------------------------------------------------------------------
Gg_RAF1            NIFLHEGL-TVKIGDFGLATVKSRWS--------------------------------------------------------------------------
Xl_RAF1            NIFLHEGL-TVKIGDFGLATVKTRWS--------------------------------------------------------------------------
Ce_RAF1            NIFLMDDMSTVKIGDFGLATVKTKWTVN------------------------------------------------------------------------
GNI_162980         NILLTDQH-EAKLGDYCEYMIERAFQPVVLDLD-------------------------------------------------------------------

                            2710      2720      2730      2740      2750      2760      2770      2780      2790      2800
                   ....|....|....|....|....|....|....|....|....|....|....|....|....|....|....|....|....|....|....|....|
PF3D7_1106800      ---------------------------------SYKISSN----------SVIKINKKEYEQ------KKAKKI------KIV-NK---------NNNDL
PFIT_1107600       ---------------------------------SYKISSN----------SVIKINKKEYEQ------KKAKKI------KIV-NK---------NNNDL
PPRFG01_1107500    ---------------------------------SYKISSN----------SVIKINKKEYEQ------KKAKKI------KIV-NK---------NNNDL
PRCDC_1105300      ---------------------------------SYKISSN----------SVIKINKKEYEQ------KKAKKI------KIV-NK---------NNNDL
PRG01_1104000      ---------------------------------SYKISSN----------SVIKINKKEYEQ------KKAKKI------KIV-NK---------NNNDL
PBILCG01_1104000   ---------------------------------SYTISSN----------SVIKINKKEYEQ------KKAKKI------KIV-KK---------KNNDL
PBLACG01_1104300   ---------------------------------SYKVSSN----------NVIKINKKEYEQ------MTAKKI------KIV-KK---------KNNDL
PADL01_1105000     ---------------------------------SYIVSSN----------SVIKINKKEYEE------KNAKKM------KIV-KK---------KNNDL
PGABG01_1103900    ---------------------------------SYIVSAN----------SVIKINKKEYEQ------KNAKKM------KIV-KK---------KNNDL
PGSY75_1106800     ---------------------------------SYIVSAN----------SVIKINKKEYEQ------KNAKKM------KIV-KK---------KNNDL
PGAL8A_00334100    ---------------------------------SYVVLSN----------KLIKFNKKHLN-------KRVKEM------KIV-NK---------NSNDL
```

```
PRELSG_0904000      ---------------------------------SYIVLSN----------KLIKFNKNHLDK------KRVKEM------KIV-NK---------NSNDL
PocGH01_09013500    ---------------------------------AYVIFSN----------NVVKFNKKYAE-------RNVKKM------KMVSNK---------DFNNL
PYYM_0941700        ---------------------------------SYIVFPN----------NVIKFNNNHFK-------NKIKKI------KIV-NK---------GSEDM
PY02790             ----------------------------------------------------------------------------------------------------
PY02791             ---------------------------------SYIVFPN----------NVIKFNNNHFK-------NKIKKI------KIV-NK---------GSEDM
PBANKA_0940100      ---------------------------------SYIVFPN----------NVIKFNNKHFK-------NKIKKI------KIV-NK---------GSEDM
PCHAS_0904200       ---------------------------------SYIVFPN----------NVIKFNNNHFK-------NKIKKI------KIV-NK---------DSEDM
PCHAS_090420        ---------------------------------SYIVFPN----------NVIKFNNNHFK-------NKIKKI------KIV-NK---------DSEDM
YYE_02751           ---------------------------------SYIVFPN----------NLIKFNNQHFK-------NKIKKI------KIV-NK---------DSEDM
YYG_01693           ---------------------------------SYIVFPN----------NVIKFNNKHFK-------NKIKKI------KIV-NK---------DSEDM
PmUG01_09016400     ---------------------------------SYVVFPN----------NLIKFNRKYSQ-------KKVKKM------QIV-NK---------KSNDL
C922_04039          ---------------------------------SYVVHPN----------GVTTFDAVLAD-------RKVQRM------EFSRN----------DLSDV
PcyM_0908600        ---------------------------------SYVVHPN----------GSTSFDAVLAD-------RKVQRM------EFSRN----------DFNDV
PKNOH_S120122800    ---------------------------------SYVVYPN----------GLTTFDVVLAD-------RNVQKM------EFSRN----------DLSDV
PKNH_0904400        ---------------------------------SYVVYPN----------GLTTFDVVLAD-------RNVQKM------EFSRN----------DLSDV
PVP01_0907600       ---------------------------------SYVVYAN----------GVTTFDAALAD-------RKVHRM------EFSRNAFNDVPLSDVPLSDV
PVX_091015          ---------------------------------SYVVYAN----------GVTTFDAALAD-------RKVHRM------EFSRNAFNDVPLSDVPLSDV
THRCLA_22740        ---------------------------------------N----------DVFGFERAHGK-----------------------------P----RPCDV
THRCLA_04632        ---------------------------------------N----------DVFGFERAHGK-----------------------------P----RPCDV
BESB_019990         PLLSPALPCPPALSPRSPSLQLCLASEPVSRHSSGAPTPS--SRRSRRRATAAAQTRGAGDP------QRESCVA--PLSRLSRLSS--------FEERP
BN1204_042460       SV-SP----PCSALSRSPTLSFACPSERFA---SGVASPVGDKKREGLRHQLGRFVTAPSSG----------------EGSTQRRIAGR------GGKDE
TGGT1_290225        P--SPFL---SARAAASPALSFVCLEET-----LGLSSPA--VKQQTPRRQLTLFSALSGVDSAAHTTRKAEGRS--TRGEEGRREEGR------SSRAA
TGDOM2_290225       P--SPFL---SARAAASPALSFVCLEET-----LGLSSPA--VKQQTPRRQLTLFSASSGVDSAAHTTRKAEGRS--TRGEEGRREEGR------SSRAA
BN1205_083850       P--SPFL---SARAAASPALSFVCLEET-----LGLSSPA--VKQQTPRRQLTLFSASSGVDSAAHTTRKAEGRS--TRGEEGRREEGR------SSRAA
HHA_290225          P--SPLL---SARAAASPALSFFCLAET-----LGLSSPA--AKQQTPRRHLPLFSAPSGADGAAHTARKAEGRS--TRGDEGRREEGS------STRAE
ETH_00000110        PAGA----------SQGPTQ------QGL----SAEATPR-------RRRGSDEGLNCPAD-------NRAEGM------SLQQDQQ--------QQQQQ
ENH_0020460         PAGA----------SQGPTQ------QGL----SAEAAPY-------RRRGSDEGPNCPAD-------NRAEGI------SLQQDQQ--------QQQQK
EMWEY_00023200      PLGAP--------PPWGPLV-----GSPWG---SPWGSPS-------AAGQAAGGPQGSAD-------RDNEGQEQEQQQEEQQQQE--------QQQQQ
Hs_RAF1             ----------------------------------------------------------------------------------------------------
Pa_RAF1             ----------------------------------------------------------------------------------------------------
Mm_RAF1             ----------------------------------------------------------------------------------------------------
Rn_RAF1             ----------------------------------------------------------------------------------------------------
Bt_RAF1             ----------------------------------------------------------------------------------------------------
Gg_RAF1             ----------------------------------------------------------------------------------------------------
Xl_RAF1             ----------------------------------------------------------------------------------------------------
Ce_RAF1             ----------------------------------------------------------------------------------------------------
GNI_162980          ---------------------------------ESVPCPK------------------------------------------------------------

                          2810      2820      2830      2840      2850      2860      2870      2880      2890      2900
                    ....|....|....|....|....|....|....|....|....|....|....|....|....|....|....|....|....|....|....|....|
PF3D7_1106800       LYLYDHNNNVYKYNTQYIDVTYNNSYPSI-------FYWTPPEILRG---KKNKKFYSDIYAFGIILWEMLSNDIP------------YNYPFASHIMAV
PFIT_1107600        LYLYDHNNNVYKYNTQYIDVTYNNSYPSI-------FYWTPPEILRG---KKNKKFYSDIYAFGIILWEMLSNDIP------------YNYPFASHIMAV
PPRFG01_1107500     LYLYDHNNNVYKYNTQYIDVTYNNSYPSI-------FYWTPPEILRG---KKNKKFYSDIYAFGIILWEMLSNDIP------------YNYPFASHIMAV
PRCDC_1105300       LYLYDHNNNVYKYNTQYIDVTYNSSYPSI-------FYWTPPEILRG---KKNKKFYSDIYAFGIILWEMLSNDIP------------YNYPFASHIMAA
PRG01_1104000       LYLYDHNNNVYKYNTQYIDVTYNSSYPSI-------FYWTPPEILRG---KKNKKFYSDIYAFGIILWEMLSNDIP------------YNYPFASHIMAA
PBILCG01_1104000    LYLYDHNNNVYKYNTQYIDVSSNSSYPSI-------FYWTPPEILRG---KKNKKFYSDIYAFGIILWEMLSNDIP------------YNYPFASHIMAA
PBLACG01_1104300    LYLYDHNNNVYKYNTQYIDVSSNSSYPSV-------FFWTPPEILRG---KKNKKFYSDIYAFGIILWEMLTNDIP------------FNYPFASHIMAA
PADL01_1105000      LYLYDHNNNVYKYNTQYIDVSTDSSYPSI-------FYWTPPEILRG---KKNKKFYSDIYAFGIILWQMLSNDIP------------FNYPFASHIMAA
PGABG01_1103900     LYLYDHNNNVYKYNTQYIDVSTDSSYPSI-------FYWTPPEILRG---KKNKKFYSDIYAFGIILWEMLSNDIP------------FNYPFASHIMAA
PGSY75_1106800      LYLYDHNNNVYKYNTQYIDVSPDSSYPSI-------FYWTPPEILRG---KKNKKFYSDIYAFGIILWEMLSNDIP------------FNYPFASHIMAA
PGAL8A_00334100     LYIYDDKNNVNEYNTKLIYGSPSNHYSYV-------SYWTPPEVLRG---KASKYLYSDVYAFGIILWEMLSNNIP------------FNYPFKSHLVAS
PRELSG_0904000      LYIYDDKNNVHKYNTRQLYVSPSSHYSYI-------SFWTPPEILRG---KKSRHLYSDVYAFGIILWEMLSNNIP------------FNYPFKSHLMAS
PocGH01_09013500    LYVHTDKNYVHKYHTKEIYTSSTNLYNSI-------SFWTAPEILRG---KKNTRLYSDVYAFGIILWEMLANSIP------------FNYPFKSHLVAS
PYYM_0941700        LHVFSSKNHIYKYNTRKIDVSSNNHDSSV-------SFWTAPEILKR---KQNPSLYSDVYAYGVILWELMTNSVP------------FNYRFKSHLLAS
PY02790             ----------------------------------------------------------------------------------------------------
PY02791             LHVFSSKNHIYKYNTRKIDVSSNNHDSSV-------SFWTAPEILKR---KQNPSLYSDVYAYGVILWELMTNSVP------------FNYRFKSHLLAS
PBANKA_0940100      LHVFSSKNHIYKYNTREINVSSNTHNSSV-------FFWTSPEILKG---KQSPSLYSDVYAYGIILWELMTNSVP------------FNYRFKSHLLAS
PCHAS_0904200       LHVFSTQNNVYKYNTREIDVSSNSH-NSV-------FFWTAPEILKG---GKNPSLYSDVYAYGIILWELMTSSIP------------FNYRFKSHLVSS
PCHAS_090420        LHVFSTQNNVYKYNTREIDVSSNSH-NSV-------FFWTAPEILKG---GKNPSLYSDVYAYGIILWELMTSSIP------------FNYRFKSHLVSS
YYE_02751           LHVFSAQNNIYKYNTREIDIPSNIH-DSV-------FFWTAPEILKG---KKNTRLYSDVYAYGIILWELMTNSIP------------FNYRFKSHLVAS
YYG_01693           LHVFNTKNNIYKYNTREIDIPSNSH-DSV-------FFWTAPEILKG---KKNPSRYSDIYAYGIILWELMTSSIP------------FNYRFKSHLVAS
PmUG01_09016400     LYIYDDKNNAHKYNTKQIYSSSAGQCAPI-------SFWTPPEVLRG---KRNKLAYADVYAFGIILWEMLSNSIP------------FNYPFKSHLVAA
C922_04039          LRVYDDEDQLHLYSIERIVASPSSAYPSV-------SFWTPPEILRG---QRGRPFYTDVYALGIVLWEMLTHSVP------------FNYPFKSHLVAS
PcyM_0908600        LRVYDDEDKMHLYSIERIVASPSSAYPSV-------SFWTPPEILRG---QRGKSFYADVYALGIVLWEMLTHSVP------------FNYPFKSHLVAS
PKNOH_S120122800    LRVHDYEDKLHLYSVQRIVASPSSAYPSV-------SFWTPPEILRG---QRGKPFYADVYALGIVLWEMLTRSVP------------FNYPFKSHLVAS
PKNH_0904400        LRVHDYEDKLHLYSVQRIVASPSSAYPSV-------SFWTPPEILRG---QRGKPFYADVYALGIVLWEMLTRSVP------------FNYPFKSHLVAS
PVP01_0907600       LRVYDDEDKLHLYSTERIVASPSSAYPSV-------SFWTPPEILRG---QREKPFYADVYALGIVLWEMLTHSVP------------FNYPFKSHLVAS
PVX_091015          LRVYDDEDKLHLYSTERIVASPSSAYPSV-------SFWTPPEILRG---QREKPFYADVYALGIVLWEMLTHSVP------------FNYPFKSHLVAS
THRCLA_22740        --------------------------------------YLAPEILSG---NSNYDTRSDIYSFGIVLWEMITKESPEQIYSEYQEKMRRNYSFTNNEFV-
THRCLA_04632        --------------------------------------YLAPEILSG---NSNYDTRSDVYSFGIVLWEMITKESPEQIYSEYQEKMRRNYSFTNNEFV-
BESB_019990         LRSGSEVALDVAEETLELRDGGGKEEFVAWLQR---LNWASPEELLGY-FPPHARGACDIHAFGVILWQMLSGLVP------------FQFMSPAQIIGA
BN1204_042460       RREPRSQNRPNSQSSKEKTDFTADEEDTAWLKR---VNWASPEELLGY-IPPQTSGSADIHAFGVILWQMLSGLVP------------HQFMSPAQIIGA
TGGT1_290225        RRESSKSQKKRSQRARESARGTGEDSSDLWLKH---ANWASPEELLCQ-IHPQTRGASDIHAFGVILWQMVTGLVP------------HQFMSTAQIIGA
TGDOM2_290225       RRESSKSQKKRSQRARESARGTGEDSSDLWLKH---ANWASPEELLCQ-IHPQTRGASDIHAFGVILWQMVTGLVP------------HQFMSTAQIIGA
BN1205_083850       RRESSKSQKKRSQRARESARGTGEDSSDLWLKH---ANWASPEELLCQ-IHPQTRGASDIHAFGVILWQMVTGLVP------------HQFMSTAQIIGA
HHA_290225          RRESSKSQKKRSQRARELASGTGEDSSDLWLKR---ANWASPEELLGQ-IRPQTRGSSDIHAFGVIMWQMLTGLVP------------HQFMSTAQIIGA
ETH_00000110        LQ--RQRQEDLQQQISAVRAISAALRQFSWYERMLQLPWSPPAVCLGL-SEPDEKKYIDVYSFGVVFWQILTGLQP------------FAELSSAQIVGC
ENH_0020460         LQ--QQRQEDLQQQISAARAISAALRQFSWYERMLQLPWSPPAVCLGL-SEPDEKKYIDVYSFGVVFWQILTGLQP------------FAELSSAQIVGC
EMWEY_00023200      LQQYEQQQYEQQQQGAAARALLDALRQSDWCERMLQLPWNPPSVSLGL-SHPEEKKYIDVYSFGAVFWQILTGRPP------------FAELSSAQIVGC
Hs_RAF1             ----------GSQQVE--QPTGS-------------VLWMAPEVIRMQ-DNNPFSFQSDVYSYGIVLYELMTGELP------Y-----SHINNRDQIIFM
Pa_RAF1             ----------GSQQVE--QPTGS-------------VLWMAPEVIRMQ-DNNPFSFQSDVYSYGIVLYELMTGELP------Y-----SHINNRDQIIFM
Mm_RAF1             ----------GSQQVE--QPTGS-------------VLWMAPEVIRMQ-DDNPFSFQSDVYSYGIVLYELMAGELP------Y-----AHINNRDQIIFM
```

```
Rn_RAF1              ----------GSQQVE--QPTGS-------------VLWMAPEVIRMQ-DNNPFSFQSDVYSYGIVLYELMTGELP------Y-----SHINNRDQIIFM
Bt_RAF1              ----------GSQQVE--QPTGS-------------ILWMAPEVIRMQ-DNNPFSFQSDVYSYGIVLYELMTGELP------Y-----SHINNRDQIIFM
Gg_RAF1              ----------GSQQVE--QPTGS-------------ILWMAPEVIRMQ-DSNPFSFQSDVYSYGIVLYELMTGELP------Y-----SHINNRDQIIFM
Xl_RAF1              ----------GSQQVE--QLTGS-------------ILWMAPEVIRMQ-DNNPFSFQSDVYSYGIVLYELMTGELP------Y-----SHIRDRDQIIFL
Ce_RAF1              ----------GGQQQQ--QPTGS-------------ILWMAPEVIRMQ-DDNPYTPQSDVYSFGICMYEILSSHLP------Y-----SNINNRDQILFM
GNI_162980           ----------CALQTRTLQLVPTVQKRES-------IHFLAPEFLRKPIICKSNTVEADIYSFGLTTAAVLLKKIP------------YPQLSPTQIRVV

                             2910      2920      2930      2940      2950      2960      2970      2980      2990      3000
                     ....|....|....|....|....|....|....|....|....|....|....|....|....|....|....|....|....|....|....|....|
PF3D7_1106800        VG---YANEELS--FNNIPVSIQSLIKACVNRNKYKRPTFEHILK-------TISTLYQKANTKV---EDALISFMDGT---------------------
PFIT_1107600         VG---YANEELS--FNNIPVSIQSLIKACVNRNKYKRPTFEHILK-------TISTLYQKANTKV---EDALISFMDGT---------------------
PPRFG01_1107500      VG---YANEELS--FNNIPVSIQSLIKACVNRNKYKRPTFEHILK-------TISTLYQKANTKV---EDALISFMDGT---------------------
PRCDC_1105300        VG---YANEELS--FNNIPVSIQSLIKACVNRNKYKRPTFEHILK-------TISTLYQKANTKV---EDALIS-MDGT---------------------
PRG01_1104000        VG---YANEELS--FNNIPVSIQSLIKACVNRNKYKRPTFEHILK-------TISTLYQKANTKV---EDALISFMDGT---------------------
PBILCG01_1104000     VG---YANEELS--FNNIPVSIQSLIKACVNRNKYKRPTFEHILK-------TISTLYQKANTKV---EDALISFMDGT---------------------
PBLACG01_1104300     VG---YANEELS--FNNIPVSIQSLIKACVNRNKYKRPTFEHILK-------TISTLYQKANTKV---EDALISFMDGT---------------------
PADL01_1105000       VG---YANEELS--FNNIPVSIQSLIKACVNRNRYKRPTFEHILK-------TISTLYQKANTKV---EDALISFMDGT---------------------
PGABG01_1103900      VG---YANEELS--FNNIPVSIQSLIKACVNRNRYKRPTFEHILK-------TISTLYQKANTKV---EDALISFMDGT---------------------
PGSY75_1106800       VG---YANEELS--FNNIPVSIQSLIKACVNRNRYKRPTFEHILK-------TISTLYQKANTKV---EDALISFMDGT---------------------
PGAL8A_00334100      VG---YAKEELS--FNNIPIPIQSLIKSCINRDKRKRPSFQKILN-------ILSKLYDQANTKA---EDALISFMDGT---------------------
PRELSG_0904000       VG---YAKEELS--FNNIPMPIQSLIKSCINRDKSKRPSFEQILN-------TLSKLYDQANTKA---EDALISFMDGT---------------------
PocGH01_09013500     VG---YAKEELP--FNNIPTPIQSLIRSCVNRDKYKRPTFDHILY-------TLSKLYEKANTKA---EDALISFMDGM---------------------
PYYM_0941700         VG---YAKETLP--FQNIPPFIKNLIKSCVNRNKYKRPTFDRILI-------ELSMIYEKINPKE---EDALMSFMDG----------------------
PY02790              ----------------------------------------------------------------------------------------------------
PY02791              VG---YAKETLP--FQNIPPFIKNLIKSCVNRNKYKRPTFDRILI-------ELSMIYEKINPKE---EDALMSFMDG----------------------
PBANKA_0940100       VG---YAKESLP--FQNIPPFIKNLIKSCINRNKYKRPTFDRILI-------EISMIYEQINPKE---EDALMSFMDG----------------------
PCHAS_0904200        VG---YAKEKLS--FQNIPPYIKNLIKSCINRDKYKRPTFDKILI-------ELSMIYEKINSKE---EDALMSFMDG----------------------
PCHAS_090420         VG---YAKEKLS--FQNIPPYIKNLIKSCINRDKYKRPTFDKILI-------ELSMIYEKINSKE---EDALMSFMDG----------------------
YYE_02751            VG---YAKEKLS--FQNIPPYIKNLIKSCINRDIYKRPTFDEILI-------ELSMIYEKINPKE---EDALMSFMDG----------------------
YYG_01693            VG---YAKEKLS--FQNIPPYIKNLIKSCINRDKYKRPTFDKILI-------ELSMIYEKINPKE---EDALMSFMDG----------------------
PmUG01_09016400      VG---FAKEQLS--FSNIPMPIQSLIKSCTNRDKYKRPTFGQILI-------VLSKLYEKANTKA---EDALISFMDGT---------------------
C922_04039           VG---YAKEELT--YDNIPEPIQGLIKSCVHRNMYRRPTFGHILA-------ELSRLYEKANTKA---EDALMSFMDGA---------------------
PcyM_0908600         VG---YAKEELT--YNNIPEPIQGLIKSCVHRNMYKRPTFGHILA-------ELSRLYEKANTKA---EDALMSFMDGA---------------------
PKNOH_S120122800     VG---YAKEELN--YNNIPDPIQGLIKSCVHRNMYKRPNFEQILA-------ELSRLYEKANTKA---EDALMSFMDGT---------------------
PKNH_0904400         VG---YAKEELN--YNNIPDPIQGLIKSCVHRNMYKRPNFEQILA-------ELSRLYEKANTKA---EDALMSFMDGT---------------------
PVP01_0907600        VG---YANEELT--YDDIPEPIQGLIKSCVHRNMYKRPTFGHILA-------VLSRLYEKANTKC---EDALMSFMDGT---------------------
PVX_091015           VG---YANEELT--YDDIPEPIQGLIKSCVHRNMYKRPTFGHILA-------VLSRLYEKANTKC---EDALMSFMDGT---------------------
THRCLA_22740         LE---FPSEDLS-----IPEYVEDLFEDCTDGNPYNRPTFEEILL-------TLESEKSKLMAKF-----------------------------------
THRCLA_04632         LE---FPSEDSS-----IPEYVEDLFEDCTDGNPYKRPSFEDILR-------RLEKEKSKLIPKE---S-------------------------------
BESB_019990          VG---LGRDSLPDLGPQVPLSLRVLIRCCTHPDPRRRPSFHHIVS-------HLVSAQEEANTPA---EEALIDFMNGT---------------------
BN1204_042460        VA---LARDTLPELDPHVPSSLRVLIRCCTHSDPRQRPSFHQIVS-------LIACAYEEVNSPA---EEALIDFMNGT---------------------
TGGT1_290225         VA---VGRDMLPEVDPHVPFSLRVLIRCCTHPDPRLRPSFHQIVS-------LIARAYEEVNSPE---EDALIDFMNGT---------------------
TGDOM2_290225        VA---VGRDMLPEVDPHVPFSLRVLIRCCTHPDPRLRPSFHQIVS-------LIARAYEEVNSPE---EDALIDFMNGT---------------------
BN1205_083850        VA---VGRDMLPEVDPHVPFSLRVLIRCCTHPDPRLRPSFHQIVS-------LIARAYEEVNSPE---EDALIDFMNGT---------------------
HHA_290225           VA---VGRDMLPEVDFHVPSSLRVLIRCCTHPDPRLRPSFNQIVS-------LIARAYEEVNSPE---EDALIDFMNGT---------------------
ETH_00000110         VE---FGKGPLGNPYDYIPHQLKTLIDKCCQRNGHRRPTFLTIVH-------AIAALHARTRDSA---EDALEEFMGAL---------------------
ENH_0020460          VE---FWKEPLGNPYDYIPYQLKTLIDKCCQRNGHRRPTFLTIVH-------AIAALHARTRDSA---EDALEEFMGAL---------------------
EMWEY_00023200       VE---LWDDPLGNPCSGIPHQLMVLLKKCCQRKANRRPTFLALLH-------AIAALYAKTHESA---EDALEEFMGAIY--------------------
Hs_RAF1              VG-RGYASPDLSKLYKNCPKAMKRLVADCVKKVKEERPLFPQILSSIELLQHSLPKINRSASEPS-LHRAAHTEDINACTLT----------TSPRLP--
Pa_RAF1              VG-RGYASPDLSKLYKNCPKAMKRLVADCVKKVKEERPLFPQILSSIELLQHSLPKINRSASEPS-LHRAAHTEDINACTLT----------TSPRLP--
Mm_RAF1              VG-RGYASPDLSRLYKNCPKAMKRLVADCVKKVKEERPLFPQILSSIELLQHSLPKINRSASEPS-LHRAAHTEDINACTLT----------TSPRLP--
Rn_RAF1              VG-RGYASPDLSRLYKNCPKAMKRLVADCVKKVKEERPLFPQILSSIELLQHSLPKINRSASEPS-LHRAAHTEDINACTLT----------TSPRLP--
Bt_RAF1              VG-RGYASPDLSKLYKNCPKAMKRLVADCVKKVKEERPLFPQILSSIELLQHSLPKINRSASEPS-LHRAAHTEDINACTLT----------TSPRLP--
Gg_RAF1              VG-RGYASPDLSKLYKNCPKAMKRLVADCLKKVREERPLFPQILSSIELLQHSLPKINRSASEPS-LHRASHTEDINSCTL-----------TSTRLP--
Xl_RAF1              VG-RGGVVPDLSKLYKNCPKAMKRLVADSIKKLRDERPLFPQILSSIELLQHSLPKINRSALEPS-LHRAAHTEDISSCAL-----------TSTRLP--
Ce_RAF1              VG-RGYLRPDRSKIRHDTPKSMLKLYDNCIMFDRNERPVFGEVLE--RLRDIILPKLTRSQSAPNVLHLDSQYSVMDAVMRSQMLSWSYIPPATAKTPQS
GNI_162980           RGFSDVAPEIIQD-LDNGWEPIKSTIIQCIQQDPSRRCSWKVICN-------LLLHYKHTASAIA---ENALISFLTGNE--------------------

                             3010
                     ....|....|....|....
PF3D7_1106800        -------------------
PFIT_1107600         -------------------
PPRFG01_1107500      -------------------
PRCDC_1105300        -------------------
PRG01_1104000        -------------------
PBILCG01_1104000     -------------------
PBLACG01_1104300     -------------------
PADL01_1105000       -------------------
PGABG01_1103900      -------------------
PGSY75_1106800       -------------------
PGAL8A_00334100      -------------------
PRELSG_0904000       -------------------
PocGH01_09013500     -------------------
PYYM_0941700         -------------------
PY02790              -------------------
PY02791              -------------------
PBANKA_0940100       -------------------
PCHAS_0904200        -------------------
PCHAS_090420         -------------------
YYE_02751            -------------------
```

```
YYG_01693           -------------------
PmUG01_09016400     -------------------
C922_04039          -------------------
PcyM_0908600        -------------------
PKNOH_S120122800    -------------------
PKNH_0904400        -------------------
PVP01_0907600       -------------------
PVX_091015          -------------------
THRCLA_22740        -------------------
THRCLA_04632        -------------------
BESB_019990         -------------------
BN1204_042460       -------------------
TGGT1_290225        -------------------
TGDOM2_290225       -------------------
BN1205_083850       -------------------
HHA_290225          -------------------
ETH_00000110        -------------------
ENH_0020460         -------------------
EMWEY_00023200      -------------------
Hs_RAF1             --------------VF---
Pa_RAF1             --------------VF---
Mm_RAF1             --------------VF---
Rn_RAF1             --------------VF---
Bt_RAF1             --------------VF---
Gg_RAF1             --------------VF---
Xl_RAF1             --------------VF---
Ce_RAF1             AAAAAAANKKAYYNVYGLI
GNI_162980          -------------------
```

Supporting Information 6 – MAFFT Alignment SAM and SAM-like domains

```
                              10        20        30        40        50        60        70        80        90       100
                     ....|....|....|....|....|....|....|....|....|....|....|....|....|....|....|....|....|....|....|....|
PWI_SRM160_Hs        QLKFAECLEKKVDMSKVNLEV-----IKPWITKRVTEI-------------LGF-EDDVVI---------------------------------EFIFNQ
PWI_PF3D7_0311100    -------------VNKIKFPL-----IETWINKRLIEI-------------LGF-EDDILY---------------------------------EYCISQ
PWI_RBM25_Hs         PELFAYPLDWSI-VDSILMER----RIRPWINKKIIEY-------------IGE-EEATLV---------------------------------DFVCSK
PWI_PF3D7_0610200    --------------------T----KLKPWIYKKITEY-------------IGA-DEKDIIE-----------------------------EISNYFVKQ
SAM2_SARM1_Hs        ---------------------------------NLADW-LGSL----D---PRF--RQYTY---------------------------------GLVSCG
SAM2_Sarm1_Mm        ---------------------------------NLADW-LGSL----D---PRF--RQYTY---------------------------------GLVSCG
BAF_Hs               ------------------MTTS--QKHRDFVAEPMGEKPVGSL--------AGI--GEVLG---K------------------------KLEERGFDKAY
BAF_Dm               ------------------MSGT-SQKHRNFVAEPMGNKSVTEL--------AGI--GETLG---G------------------------RLKDAGFDMAY
BAF_Ce               ------------------MSTS--VKHREFVGEPMGDKEVTCI--------AGI--GPTYG---T------------------------KLTDAGFDKAY
PWI_USP107_Sp        -------------ESLWALPIDWSKVTEDLLKEEMQAFVTKKIIEY-----IGI-QEDSLI---------------------------------TFTIDH
Pnt_ETS1_Hs          ----ATFSGFTKEQQRLGIPK----DPRQWTETHVRDW-VMWAVNE-----FSL-KGVDFQ---------------------------------KF-CM-
Pnt_ETS1_Mm          ----ATFSGFTKEQQRLGIPK----DPRQWTETHVRDW-VMWAVNE-----FSL-KGVDFQ---------------------------------KF-CM-
Pnt_ETS2_Hs          ----ATFSGFKKEQRRLGIPK----NPWLWSEQQVCQW-LLWATNE-----FSL-VNVNLQ---------------------------------RF-GM-
Pnt_ETS2_Mm          ----ATFSGFQKEQRRLGIPK----NPWLWSEQQVCQW-LLWATNE-----FSL-VNVNLH---------------------------------QF-GM-
Pnt_PNT2_Dm          ----ASFASWEKEVQKCNITK----DPREWTEEHVIYW-LNWAKNE-----FSL-VSMNLD---------------------------------PFYKM-
Pnt_GABPalpha_Mm     ----AALEGYRKEQERLGIPY----DPIHWSTDQVLHW-VVWVMKE-----FSM-TDIDLT------------------------------------TLN
Pnt_ERG_Hs           ----MPPPNMTTNERRVIVPA----DPTLWSTDHVRQW-LEWAVKE-----YGL-PDVNIL---------------------------------LFQNI-
Pnt_Etv6_Hs          --------ALRMEEDSIRLPAHLRLQPIYWSRDDVAQW-LKWAENE-----FSL---------------------------------------RPIDSNT
Pnt_Etv6_Mm          --------ALRMEEDSIHLPTHLRLQPIYWSRDDVAQW-LKWAENE-----FSL---------------------------------------RPIESNK
Pnt_Yan_Dm           --------SSQLAELKTQLPPSLPSDPRLWSREDVLVF-LRFCVREFD--LPKL----------------------------------------DFDLFQ
Pnt_Mae_Dm           ----DGTTSLLHPLGSDGLPL----DPRDWTRADVWKWLINMAVSE------GL--EVTAE---------------------------------LPQKFP
SAM_smg_Dm           ---------------------------------GIGLW-LKSL---------RL--HKYIE---------------------------------LFKNMT
SAM_smg_Xl           ---------------------------EGSGMKDVPAW-LKSL---------RL--HKYAA---------------------------------LFSHIT
SAM_smg_Rn           -----------------------------SGMKDVPAW-LKSL---------RL--HKYAA---------------------------------LFSQMT
SAM_smg1_Mm          -----------------------------SGMKDVPAW-LKSL---------RL--HKYAA---------------------------------LFSQMT
SAM_smg1_Hs          -----------------------------SGMKDVPAW-LKSL---------RL--HKYAA---------------------------------LFSQMT
SAM_smg2_Hs          -----------------------------SGMKDVPSW-LKSL---------RL--HKYAA---------------------------------LFSQMS
SAM_smg2_Mm          -----------------------------SGMKDVPSW-LKSL---------RL--HKYAA---------------------------------LFSQMS
SAM_VTS1_Sc          -----------------------------KLLKNIPMW-LKSL---------RL--HKYSD---------------------------------ALSGTP
SAM_EphA4_Hs         -----------------------------SAVVSVGDW-LQAI---------KM--DRYKD---------------------------------NFTAAG
SAM_EPhA7_Hs         -----------------------------TTFCSVGEW-LQAI---------KM--ERYKD---------------------------------NFTAAG
SAM_EphA6_Hs         -----------------------------PLFVTVGDW-LDSI---------KM--GQYKN---------------------------------NFVAAG
SAM_EphB1_Hs         -----------------------------TAFTTVDDW-LSAI---------KM--VQYRD---------------------------------SFLTAG
SAM_EphB2_Hs         -----------------------------TSFNTVDEW-LEAI---------KM--GQYKE---------------------------------SFANAG
SAM_EphB3_Hs         -----------------------------TTFTTVGDW-LDAI---------KM--GRYKE---------------------------------SFVSAG
SAM_EphB5_Gg         -----------------------------PSLSNAHEW-LDAI---------KM--GRYKE---------------------------------NFDQAG
SAM_EphA2_Hs         -----------------------------VPFRTVSEW-LESI---------KM--QQYTE---------------------------------HFMAAG
SAM_EphA3Hs          -----------------------------TTFRTTGDW-LNGV---------WT--AHCKE---------------------------------IFTGVE
SAM1_Caskin1_Hs      -----------------------------KSSEAVSQW-LTAF---------QL--QLYAP---------------------------------NFISAG
SAM_SHIP2_Hs         -----------------------------LGEAGMSAW-LRAI---------GL--ERYEE---------------------------------GLVHNG
SAM_INPPL1_Hs        -----------------------------LGEAGMSAW-LRAI---------GL--ERYEE---------------------------------GLVHNG
SAM2_Caskin1_Hs      -----------------------------HKPANLAVW-LSMI---------GL--AQYYK---------------------------------VLVDNG
SAM1_ANKS1A_Hs       -----------------------------TLEQSVGEW-LESI---------GL--QQYES---------------------------------KLLLNG
SAM2_ANKS1A_Hs       -----------------------------NSPPSVPSW-LDSL---------GL--QDYVH---------------------------------SFLSSG
SAM_Sqp53_Lf         -----------------------------PTENTIAQW-LTKL---------GL--QAYID---------------------------------NFQQKG
SAM_p63_Es           -----------------------------PNENTIAQW-LTKL---------GL--QAYID---------------------------------NFQQKG
SAM_p63alpha_Hs      --------------------------PPYPTDCSIVSF-LARL---------GC--SSCLD---------------------------------YFTTQG
SAM_p73alpha_Hs      --------------------------PPYHADPSLVSF-LTGL---------GC--PNCIE---------------------------------YFTSQG
SAM_p73_Mm           --------------------------PPYHADPSLVSF-LTGL---------GC--PNCIE---------------------------------CFTSQG
SAM_ARAP1_Hs         -----------------------------DAALSVAEW-LRAL---------HL--EQYTG---------------------------------LFEQHG
SAM_Neurabin1_Hs     -----------------------------WSVQQVSHW-LMSL---------NL--EQYVS---------------------------------EFSAQN
SAM_Neurabin_Pa      -----------------------------WSVQQVSHW-LMSL---------NL--EQYVS---------------------------------EFSAQN
SAM_Byr2_Sp          -----------------------------YTSKEVAEW-LKSI---------GL--EKYIE---------------------------------QFSQNN
SAM_BOI2_Sc          -----------------------------WSPEEITAY-FIME---------GY-DVQSAS---------------------------------RFQKHK
SAM_BOI1_Sc          -----------------------------WSPEEVTDY-FSLV---------GF-DQSTCN---------------------------------KFKEHQ
SAM_pob1_Sp          -----------------------------WSTEEVVEW-LMNA---------GL--GSVAP---------------------------------NFAENE
SAM_SDRG_01147_Sd    -----------------------------WTARQVAEW-VTRS---------GF--AEYAQ---------------------------------TFEKND
SAM_DPYK1_Dd         -----------------------------WAPNDVAIW-LESF---------NY--GQYRK---------------------------------NFRDNN
SAM_SPLA_Dd          -----------------------------WAPNDVAIW-LESF---------NY--GQYRK---------------------------------NFRDNN
SAM1_SARM1_Hs        -----------------------------WKEAEVQTW-LQQI---------GF--SKYCE---------------------------------SFREQQ
SAM1_Sarm1_Mm        -----------------------------WKEAEVQTW-LQQI---------GF--SQYCE---------------------------------NFREQQ
SAM1_tir1_Ce         -----------------------------WTCADVQYW-VKKI---------GF--EEYVE---------------------------------KFAKQM
SAM_C33B4.3_Ce       -----------------------------WSVDDVIGW-LSSL---------HL--SEYTP---------------------------------AFRSQR
SAM_DGKD_Hs          -----------------------------WGTEEVAAW-LEHL---------SL--CEYKD---------------------------------IFTRHD
SAM_SHANK1_Rn        -----------------------------WTKFDVADW-LEWL---------GL--SEHRA---------------------------------QFLDHE
SAM_SHANK3_Hs        -----------------------------WSKFDVGDW-LESI---------HL--GEHRD---------------------------------RFEDHE
SAM_ZAK_Hs           -----------------------------WTEDDVYCW-VQQLVRKGDSS-AEM--SVYAS---------------------------------LFKENN
SAM_Ste4_Sp          -----------------------------WNNEAVCNW-IEQL---------GF---PHKE---------------------------------AFEDYH
SAM_Samhd1_Mm        -----------------------------WEPEDVCSF-LENR---------GFREKKVLD---------------------------------IFRDNK
SAM_SAMHD1_Hs        -----------------------------WGPEQVCSF-LRRG---------GFEEPVLLK---------------------------------NIRENE
SAM_SPKA1_Dd         -----------------------------KKYKDLFDW-LQKH---------GF--EQYKD---------------------------------AFLKEE
SAM_DDB_G0278535_Dd  -----------------------------KNVQDIFNW-LKSI---------DL--EQYWL---------------------------------NFVKEE
SAM_DDB_G0272092_Dd  -----------------------------EKVIEISDW-LNEL---------QL--ETLIP---------------------------------KFIKNE
SAM_TNKS_Hs          ---------------------------------NISQF-LKSL---------GL--EHLRD---------------------------------IFETEQ
SAM_LRSAM1_Hs        -----------------------------GMERQLVAL-LEEL---------SA--EHYLP---------------------------------IFAHHR
SAM_SAMD3_Hs         -----------------------------WSVEQVCSW-LVEK---------NL--GELVH---------------------------------RFQEEE
SAM_PF3D7_1106800    ----------------NLIIE----NYQTWSLREVIQW-LMLC---------NV-PVKWLI---------------------------------SFYKNN
SAM_Kif6_Dd          -----------------------------FENDQLYNW-LQLA---------NL--ESFYP---------------------------------NFIKKN
```

```
SAM_KIF24_Hs          ----------------------------------MASW-LYECLC--EA---EL--AQYYS---------------------------------HFTALG
SAM_CNKSR1_Hs         -----------------------------WTPGKVATW-LRGL----DD---SL--QDY-----------------------------------PFEDWQ
SAM_Ste11_Sc          -----------------------------NDLPFVQLF-LEEI---------GC--TQYLD---------------------------------SFIQCN
SAM_PHC1_Mm           -----------------------------WSVEEVYEF-IASL--------QGC--QEIAE---------------------------------EFRSQE
SAM_RAE28_Mm          -----------------------------WSVEEVYEF-IASL--------QGC--QEIAE---------------------------------EFRSQE
SAM_PHC2_Hs           -----------------------------WNVEDVYEF-IRSL--------PGC--QEIAE---------------------------------EFRAQE
SAM_php_Dm            -----------------------------WSVDDVSNF-IREL--------PGC--QDYVD---------------------------------DFIQQE
SAM_L3MBTL3_Hs        -----------------------------WSTDEVSEF-IQSL--------PGC--EEHGK---------------------------------VFKDEQ
SAM_Scmh1_Mm          -----------------------------WTVEDVMQF-VREA-------DPQL--GSHAD---------------------------------LFRKHE
SAM_Scm_Dm            -----------------------------WTIEEVIQY-IESN-------DNSL--AVHGD---------------------------------LFRKHE
SAM_SFMBT1_Hs         -----------------------------WSVADVVRF-IRST---------DC--APLAR---------------------------------IFLDQE
SAM_Sfmbt_Dm          -----------------------------WNVYDVSQF-LRVN---------DC--TAHCD---------------------------------TFSRNK
SAM_SEC23IP_Hs        -----------------------------KEVLTLQET-LEAL---------SL--SEYFS---------------------------------TFEKEK
SAM_Ush1g_Mm          ---------------------------LEPETSPLETF-LASL---------HM--EDFAS---------------------------------LLRHEK
SAM1_LiprinA_Ce       -----------------------------WRSEQLADW-IAEI---------GY--PQYMN---------------------------------EVSRHV
SAM2_Liprin_Hs        -----------------------------NHEWIGNEW-LPSL---------GL--PQYRS---------------------------------YFMECL
SAM2_Kazrin_Hs        -----------------------------DHHWVAKAW-LNDI---------GL--SQYSQ---------------------------------AFQNHL
SAM_STIM1_Hs          -----------------------------WTVDEVVQWLITYV---------EL--PQYEE---------------------------------TFRKLQ
SAM_ave_Dm            -----------------------------WTVSDVLKWYRRHC--------GEY--TQYEQ---------------------------------LFAQHD
SAM1_Liprin_Hs        -----------------------------WDGPTVVSW-LELW--------VGM-PAWYVA---------------------------------ACRANV
SAM1_Kazrin_Hs        -----------------------------WKAGTVQAW-LEVV--------MAM--PMYVK---------------------------------ACTENV
SAM2_tir1_Ce          -----------------------------VDESNLDNF-LMGL----SP---EL--SVYTY---------------------------------QMLTNG
SAM_D52_Dm            -----------------------------NHAANVEQI-LMHM---------GL--ENYVT---------------------------------NFEEAH
SAM_SAMKD_Dd          -----------------------------WDNETVCKWLLNNI---------KTIQKVSIE---------------------------------IFQANE
SAM_SAMKC_Dd          -----------------------------WDNEMVCKW-LHDT---------KRIQKVSIE---------------------------------IFKANE
SAM_SAMKA_Dd          -----------------------------WNNEKIIKW-LSDT---------KKIQKVI-----------------------------------VFKIYE
SAM_SAMKB_Dd          -----------------------------WNNEAVCEW-LSEN---------NF-KMSDIE---------------------------------KFQENK
SAM_LCP2_Hs           -----------------------------WDPDSLADY-FKKL---------NY--KDCEK---------------------------------AVKKYH
SAM_LCP2_Mm           ------------------VLA--------WNSDNLADY-FRKL---------NY--RDCEK---------------------------------AVKKYH
SAM_GARE1_Hs          -----------------------------LSIEEVSKS-LRFI---------GL-SEDVIS---------------------------------FFVTEK
SAM_PF3D7_0211700     -------------------------NYIYWNIFELNLF-FFLV---------GI-PKEILE---------------------------------IFIYHR
SAM3_Liprin_Hs        -----------------------------WTNDQVVHW-VQSI---------GL--RDYAG---------------------------------NLHESG
SAM3_Kazrin_Hs        -----------------------------WTNQRVLKW-VRDI---------DL--KEYAD---------------------------------NLTNSG
SAM_PF3D7_0926000     ------------------------ENIYQWERHHILIL-LYLF---------KL--NRYIE---------------------------------SFSSNK
SAM_Dlc_Rn            --------------------------LTQIEAKEACDW-LRVT---------GF--PQYAQ---------------------------------LYEDLL
SAM_STA13_Hs          --------------------------QQEIEAKEACDW-LRAA---------GF--PQYAQ---------------------------------LYEDSQ
SAM_STE50_Sc          ---------------TILMNN---EDFSQWSVDDVITWCISTL---------EV--EETDPLCQ------------------------------RLREND
rpoA_Cter_Bs          -------------------------------KEKVLEMTIEEL---------DL-SVRSYN---------------------------------CLKRAG
rpoA_Cter_Ec          -------------------------------FDPILLRPVDDL---------EL-TVRSAN---------------------------------CLKAEA
rpoA_Cter_Tt          ------------------------------EQEEELDLPLEEL---------GL-STRVLH---------------------------------SLKEEG
FEN_Cter_Mja          -----------------------------ISLDDLIDIAIFMGTDYNPGGVKGIGFKRAYELVRS-GVAKDVL------KKEVEY---YDEIKRIFKEPK
FEN_Cter_Pyf          ----------------------------KLTREKLIELAILVGTDYNPGGIKGIGLKKALEIVRH---SKDPLAKFQK-QSDVD----LYAIKEFFLNPP
FEN_Cter_Pyh          -----------------------------ITREKLIELAILVGTDYNPGGVKGIGPKKALEIVRY---SRDPLAKFQR-QSDVD----LYAIKEFFLNPP
FEN_Cter_Arf          -----------------------------LTREQLIDIAILVGTDYNE-GVKGVGVKKALNYIKT---YGDIFRALKALKVNIDH---VEEIRNFFLNPP
FEN_Cter_Hs           -----------------------------LNQEQFVDLCILLGSDYCE-SIRGIGPKRAVDLIQKHKSIEEIVRRLDPNKYPVPENWLHKEAHQLFLEPE
SAM1_PF3D7_1302100    ----------------------------DIEFHACSDY-IYLL--------MTL--SKDPE---------------------------------KFNYAL
SAM2_PF3D7_1302100    ----------------DYIKK---IDDYLWVEQRVIEE-------------VSINVDHARE---------------------------------VKEKKR
PWI_PRPF3_Hs          ------------------------MALSKRELDELKPW-IEKTVK------RVL----------------------------------------GFSEPT
PWI_SWM_Dm            --------------------------------DKLKDW-LSVVLE-------PL-CDADSS---------------------------------ALARYV
SAM_PF3D7_1349300     ------------------LRA---SDYNKWSVNMLYNF-LKMI---------GL--KKEAY---------------------------------LFKINK

                              110       120       130       140       150       160       170       180
                      ....|....|....|....|....|....|....|....|....|....|....|....|....|....|....|....|....|...
PWI_SRM160_Hs         L----------EV----KNPDSKMMQIN----L-TGFLNGKNAREFMG--ELWP--LLLSAQENIAGIPSAFL-------------EL
PWI_PF3D7_0311100     LKQSKEKKDGEED----KYLNAKKLKIN----L-TGFIGNKKSDIFIE--ELLE--LLINEEKK------------------------
PWI_RBM25_Hs          V-----------M----AHSSPQSILDD----V-AMVL-DEEAEVFIV--KMWR--LLIYETEAKKIGLVK-----------------
PWI_PF3D7_0610200     I-----------L----NETSPKNMLVE----A-EKFL-DSDGKIFIL--NMYK--LIIFEQLKVQ----------------------
SAM2_SARM1_Hs         L-------DRSLL----HRVSEQQLLED----C-GIHLGVHRARIL----TAAR--EMLHS---------------------------
SAM2_Sarm1_Mm         L-------DRSLL----HRVSEQQLLED----C-GIRLGVHRTRIL----SAAR--EMLHS---------------------------
BAF_Hs                V------VLGQFL----VLKKDEDLFRE--W-L-KDTCGANAKQSR----DCFG---CLREWCDAFL---------------------
BAF_Dm                T------VLGQYL----VLKKDEELFKD--W-M-KEVCHASSKQAS----DCYN---CLNDWCEEFL---------------------
BAF_Ce                V------LFGQYL----LLKKDEDLFIE--W-L-KETAGVTANHAK----TAFN---CLNEWADQFM---------------------
PWI_USP107_Sp         IRQ----HKGAE-----QLVSELDLA------L-AEDAPEFVSKVY----RYLHVLLILRSEA-------------------------
Pnt_ETS1_Hs           --------NGAAL----CALGKDCFLE-----L----APDFVGDILW---EHLE---ILQKED-------------------------
Pnt_ETS1_Mm           --------SGAAL----CALGKECFLE-----L----APDFVGDILW---EHLE---ILQKED-------------------------
Pnt_ETS2_Hs           --------NGQML----CNLGKERFLE-----L----APDFVGDILW---EHLE---QMIKEN-------------------------
Pnt_ETS2_Mm           --------NGQML----CNLGKERFLE-----L----APDFVGDILW---EHLE---QMIKEN-------------------------
Pnt_PNT2_Dm           --------KGRAM----VDLGKEKFLA-----I----TPPFTGDILW---EHLD---ILQKDC-------------------------
Pnt_GABPalpha_Mm      I-------SGREL----CSLNQEDFFQR----V-------PRGEILW---SHLE---LLRKYV-------------------------
Pnt_ERG_Hs            --------DGKEL----CKMTKDDFQR-----L----TPSYNADILL---SHLH---YLRETP-------------------------
Pnt_Etv6_Hs           F-----EMNGKAL----LLLTKEDFRY----------RSPHSGDVLY---ELLQ--HILKQR--------------------------
Pnt_Etv6_Mm           F-----EMNGKAL----LLLTKEDFRY----------RSPHSGDVLY---ELLQ--HILKQR--------------------------
Pnt_Yan_Dm            M-------NGKAL----CLLTRADFGHR----C------PGAGDVLH---NVLQ--MLIIES--------------------------
Pnt_Mae_Dm            M-------NGKAL----CLMSLD---------M-YLCRVPVGGKMLY---RDFR--VRLARA--------------------------
SAM_smg_Dm            Y---------EEM----LLITEDFLQS-----V-GVTK-GASHKLA----LCID---KLK----------------------------
SAM_smg_Xl            Y---------DEM----MSLNECHLEA-----Q-NVTK-GARHKIV----ISIQ---KLK----------------------------
SAM_smg_Rn            Y---------EEM----MALTECQLEA-----Q-NVTK-GARHKIV----ISIQ---KLKERQNLLKSLERDI-------------IE
SAM_smg1_Mm           Y---------EEM----MALTECQLEA-----Q-NVTK-GARHKIV----ISIQ---KLKERQNLLKSLERDI-------------IE
SAM_smg1_Hs           Y---------EEM----MALTECQLEA-----Q-NVTK-GARHKIV----ISIQ---KLKERQNLLKSLE------------------
SAM_smg2_Hs           Y---------EEM----MTLTEQHLES-----Q-NVTK-GARHKIA----LSIQ---KLRERQSVLKSLEKDV-------------LE
SAM_smg2_Mm           Y---------EEM----MTLTEQHLES-----Q-NVTK-GARHKIA----LSIQ---KLRERQSVLKSLEKDV-------------LE
SAM_VTS1_Sc           W---------IEL----IYLDDETLEK-----K-GVLALGARRKLLKAFGIVID---Y------------------------------
```

```
SAM_EphA4_Hs        Y------TTLEAV----VHVNQEDLAR-----I-GITAITHQNKIL----SSVQ---AMRTQ------------------------
SAM_EPhA7_Hs        Y------NSLESV----ARMTIEDVMS-----L-GITLVGHQKKIM----SSIQ---TMRAQ------------------------
SAM_EphA6_Hs        F------TTFDLI----SRMSIDDIRR-----I-GVILIGHQRRIV----SSIQ---TLRLH------------------------
SAM_EphB1_Hs        F------TSLQLV----TQMTSEDLLR-----I-GITLAGHQKKIL----NSIH---SMRVQ------------------------
SAM_EphB2_Hs        F------TSFDVV----SQMMMEDILR-----V-GVTLAGHQKKIL----NSIQ---VMRAQ------------------------
SAM_EphB3_Hs        F------ASFDLV----AQMTAEDLLR-----I-GVTLAGHQKKIL----SSIQ---DMRLQ------------------------
SAM_EphB5_Gg        L------ITFDVI----SRMTLEDLQR-----I-GITLVGHQKKIL----NSIQ---LMKVH------------------------
SAM_EphA2_Hs        Y------TAIEKV----VQMTNDDIKR-----I-GVRLPGHQKRIA----YSLL---GLKDQ------------------------
SAM_EphA3Hs         Y------SSCDTI----AKISTDDMKK-----V-GVTVVGPQKKII----SSIK---ALETQ------------------------
SAM1_Caskin1_Hs     Y-------DLPTI----SRMTPEDLTA-----I-GVTKPGHRKKIA----AEIS---GLSIP------------------------
SAM_SHIP2_Hs        W------DDLEFL----SDITEEDLEE-----A-GVQDPAH-KRLL----LDTL---QLSK-------------------------
SAM_INPPL1_Hs       W------DDLEFL----SDITEEDLEE-----A-GVQDPAH-KRLL----LDTL---QLSK-------------------------
SAM2_Caskin1_Hs     Y------ENIDFI----TDITWEDLQE-----I-GITKLGHQKKLM----LAVR---KLAEL------------------------
SAM1_ANKS1A_Hs      F------DDVHFLG--SNVMEEQDLRD-----I-GISDPQHRRKLL----QAAR---SLPKV------------------------
SAM2_ANKS1A_Hs      Y------SSIDTV----KNLWELELVNV----L-KVQLLGHRKRII----ASLAD-RPYEEP------------------------
SAM_Sqp53_Lf        L------HNMFQL----DEFTLEDLQS-----M-RIG-------------------------------------------------
SAM_p63_Es          L------HNMFQL----DEFTLEDLQS-----M-RIG-------------------------------------------------
SAM_p63alpha_Hs     L------TTIYQI----EHYSMDDLAS-----L-KIPEQ-FRHAIW----KGIL---DHRQL------------------------
SAM_p73alpha_Hs     L------QSIYHL----QNLTIEDLGA-----L-KIPEQ-YRMTIW----RGLQ---DLKQG------------------------
SAM_p73_Mm          L------QSIYHL----QNLTIEDLGA-----L-KVPDQ-YRMTIW----RGLQ---DLKQS------------------------
SAM_ARAP1_Hs        L------VWATEC----QGLSDTRLMD-----M-GMLLPGHRRRIL----AGLL---RAHTS------------------------
SAM_Neurabin1_Hs    I-------TGEQL----LQLDGNKLKA-----L-GMTASQDRAVVK----KKLK---EMKMS------------------------
SAM_Neurabin_Pa     I-------TGEQL----LQLDGNKLKA-----L-GMTASQDRAVVK----KKLK---EMKMS------------------------
SAM_Byr2_Sp         I-------EGRHL----NHLTLPLLKD-----L-GIENTAKGKQFL----KQRD---YLREF------------------------
SAM_BOI2_Sc         I-------SGKIL----LELELVHLKE-----L-DINSFGTRFEIF----KEIE---KIKEA------------------------
SAM_BOI1_Sc         V-------SGKIL----LELELEHLKE-----L-EINSFGIRFQIF----KEIR---NIKSA------------------------
SAM_pob1_Sp         I-------TGEIL----LGLDSNVLKE-----L-NITSFGKRFEVL----RKIQ---QLKDS------------------------
SAM_SDRG_01147_Sd   I-------TGDVL----PTITNDVLRED----L-KVSAYGHRIKLV----NAIQ---KLIEA------------------------
SAM_DPYK1_Dd        I-------SGRHL----EGITHAMLKND----L-GIEPYGHREDII----NRLN---RMIQI------------------------
SAM_SPLA_Dd         I-------SGRHL----EGITHAMLKND----L-GIEPYGHREDII----NRLN---RMIQI------------------------
SAM1_SARM1_Hs       V-------DGDLL----LRLTEEELQTD----L-GMKSGITRKRFF----RELT---ELKTF------------------------
SAM1_Sarm1_Mm       V-------DGDLL----LRLTDEELQTD----L-GMKSSITRKRFF----RELT---ELKTF------------------------
SAM1_tir1_Ce        V-------DGDLL----LQLTENDLKHD----V-GMISGLHRKRFL----RELQ---TLKVA------------------------
SAM_C33B4.3_Ce      I-------NGRCL----RQCDRSRFTQ-----L-GVTRIAHRQIIE----SALR---GLLQ-------------------------
SAM_DGKD_Hs         I-------RGSEL----LHLERRDLKD-----L-GVTKVGHMKRIL----CGIK---ELSRS------------------------
SAM_SHANK1_Rn       I-------DGSHL----PALTKEDYVD-----L-GVTRVGHRMNID----RALK---FFLER------------------------
SAM_SHANK3_Hs       I-------EGAHL----PALTKDDFVE-----L-GVTRVGHRMNIE----RALR---QLDGS------------------------
SAM_ZAK_Hs          I-------TGKRL----LLLEEEDLKD-----M-GIVSKGHIIHFK----SAIE---KLTHD------------------------
SAM_Ste4_Sp         I-------LGKDI----DLLSSNDLRD-----M-GIESVGHRIDIL----SAIQ---SMKKQ------------------------
SAM_Samhd1_Mm       I-------AGSFL----PFLDEDRLED-----L-GVSSLEERKKMI----ECIQ---QLSQS------------------------
SAM_SAMHD1_Hs       I-------TGALL----PCLDESRFEN-----L-GVSSLGERKKLL----SYIQ---RLVQI------------------------
SAM_SPKA1_Dd        M-------FLDEL----GEMSEDILNK-----M-GITSTGTRLRIL----KETS---NLANE------------------------
SAM_DDB_G0278535_Dd I-------FMDLL----LDIDERTLDS-----L-GITYSGHRLKII----RNCR---ILRDQ------------------------
SAM_DDB_G0272092_Dd I-------YMDVI----TDINEGTLDL-----L-NISVSGQRTKLL----RAVR---KIKDP------------------------
SAM_TNKS_Hs         I-------TLDVL----ADMGHEELKE-----I-GINAYGHRHKLI----KGVE---RLLGG------------------------
SAM_LRSAM1_Hs       L-------SLDLL----SQMSPGDLAK-----V-GVSEAGLQHEIL----RRVQ---ELLDA------------------------
SAM_SAMD3_Hs        V-------SGAAL----LALNDRMVQQ-----L--VKKIGHQAVLM----DLIK---KYKQNTQGLK-------------------
SAM_PF3D7_1106800   I-------TGDKL----KYININTIRNE----L-GIIAYGHAIKIL----QLIK---NLQVMAYN---------------------
SAM_Kif6_Dd         V-------SCDSF----LSFTMQDYGN-----V-GITSLQDRKKLF----HLLQ---QLKKQ------------------------
SAM_KIF24_Hs        L------QKIDEL----AKITMKDYSK-----L-GVHDMNDRKRLF----QLIK---IIKIM------------------------
SAM_CNKSR1_Hs       L-------PGKNL----LQLCPQSLEA-----L-AVRSLGHQELIL----GGVE---QLQAL------------------------
SAM_Ste11_Sc        L------VTEEEI----KYLDKDILIA-----L-GVNKIGDRLKIL----RKSK---SFQRD------------------------
SAM_PHC1_Mm         I-------DGQAL----LLLKEEHLMSA----M-NI-KLGPALKIC----AKIN---VLKET------------------------
SAM_RAE28_Mm        I-------DGQAL----LLLKEEHLMSA----M-NI-KLGPALKIC----AKIN---VLKET------------------------
SAM_PHC2_Hs         I-------DGQAL----LLLKEDHLMSA----M-NI-KLGPALKIY----ARIS---MLKDS------------------------
SAM_php_Dm          I-------DGQAL----LLLKEKHLVNA----M-GM-KLGPALKIV----AKVE---SIKEV------------------------
SAM_L3MBTL3_Hs      I-------DGEAF----LLMTQTDIVKI----M-SI-KLGPALKIF----NSIL---MFKAA------------------------
SAM_Scmh1_Mm        I-------DGKAL----LLLRSDMMMKY----M-GL-KLGPALKLS----FHID---RLKQV------------------------
SAM_Scm_Dm          I-------DGKAL----LLLNSEMMMKY----M-GL-KLGPALKIC----NLVN---KVNGRRNNLA-------------------
SAM_SFMBT1_Hs       I-------DGQAL----LLLTLPTVQEC----M-DL-KLGPAIKLC----HHIE---RIKFAFYEQF-------------------
SAM_Sfmbt_Dm        I-------DGKRL----LQLTKDDIMPL----L-GM-KVGPALKIS----DLIA---QLKCK------------------------
SAM_SEC23IP_Hs      I-------DMESL----LMCTVDDLKE-----M-GI-PLGPRKKIA----NFVE--HKAAKL------------------------
SAM_Ush1g_Mm        I-------DLEAL----MLCSDLDLRS-----I-SVP-LGPRKKIL----GAVR---RRR--------------------------
SAM1_LiprinA_Ce     R-------SGRHF----LNMSMNEYEGV----L-NIKNPVHRKRVA----ILLR---RIEED------------------------
SAM2_Liprin_Hs      V-------DARML----DHLTKKDLRVH----L-KMVDSFHRTSLQ----YGIM---CLKRL------------------------
SAM2_Kazrin_Hs      V-------DGRML----NSLMKRDLEKH----L-NVSKKFHQVSIL----LGIE---LLYQV------------------------
SAM_STIM1_Hs        L-------SGHAMP--RLAVTNTTMTGT---VL-KMTDRSHRQKLQ----LKAL---DTVLF------------------------
SAM_ave_Dm          I-------TGRAL----LRITDSSLQR-----M-GVTDNRDREAIW----REIV---KQRLK------------------------
SAM1_Liprin_Hs      K-------SGAIM----SALSDTEIQRE----I-GISNALHRLKLR----LAIQ---EMVSL------------------------
SAM1_Kazrin_Hs      K-------SGKVL----LSLSDEDLQLG----L-GVCSSLHRRKLR----LAIE---DYRDA------------------------
SAM2_tir1_Ce        V-------NRSLL----SSLTDEMMQNA----C-GITNPIHRLKLT----QAFE---TAKHP------------------------
SAM_D52_Dm          I-------DLVEL----ASLERADLVK-----I-GLNTDEDCNRIM----DVLH---TL---------------------------
SAM_SAMKD_Dd        I-------IGKDL----EFLTDKILLK-----M-GV-GIRDILNFK----FEYQ---ILKNCY-----------------------
SAM_SAMKC_Dd        I-------TGNYL----ESLTDKMLLK-----M-GL-TIRDLLSFR----SEFD---DLKNIF-----------------------
SAM_SAMKA_Dd        I-------TGRDL----EFLSDKILFK-----M-GV-GIRDLLSFK----SEFE---ILKNNY-----------------------
SAM_SAMKB_Dd        I-------KGDHL----EFIGDKILIQ-----M-GL-SIYERLSFK----SIFK---KLKNNN-----------------------
SAM_LCP2_Hs         I-------DGARF----LNLTENDIQK-----F-PKLRVPILSKLS----QEIN---KNEERRSI---------------------
SAM_LCP2_Mm         I-------DGARF----LNLTENDIQK-----F-PKLRMPLLSKLS----QDIN---KNEER------------------------
SAM_GARE1_Hs        I-------DGNLL----VQLTEEILSED----F-KLSKL-QVKKIM----QFIN---GWRPKI-----------------------
SAM_PF3D7_0211700   L-------DGYCL----KYIDKKILKE-----M-KIKNRMMRKYI-----------------------------------------
SAM3_Liprin_Hs      V-------HGALLA-LDENFDHNTLALI----L-QIPTQNTQARQVME--REFN---NLLAL------------------------
SAM3_Kazrin_Hs      V-------HGAVLV-LEPTFNAEAMATA----L-GIPSGKHILR------RHLA--EEMSAVF-----------------------
```

```
SAM_PF3D7_0926000    I-------KGIHFF---FILNSKVLKE-----L-GINNKEHIFFLM----NIIN---TFNSI-------------------------
SAM_Dlc_Rn           F-------PIDIA---LVKREHDFLDRD------AIEALCRRLNTL----NKCA---VMKLE-------------------------
SAM_STA13_Hs         F-------PINIV---AVKNDHDFLEKD------LVEPLCRRLNTL----NKCA---SMKLD-------------------------
SAM_STE50_Sc         I-------VGDLL----PELCLQDCQDL----CDGDLNKAIKFKILI---NKMRD-SKLEWKD------------------------
rpoA_Cter_Bs         I------NTVQEL----ANKTEEDMMKV--RNL-GRKSLEEVKAKL----EELG--LGLRKDD------------------------
rpoA_Cter_Ec         I------HYIGDL----VQRTEVELLKT--PNL-GKKSLTEIKDVLAS--RGLS--LGMRLENWPPASIADE---------------
rpoA_Cter_Tt         I------ESVRAL----LALNLKDLKNI--PGI-GERSLEEIKEAL----EKKG--FTLKE--------------------------
FEN_Cter_Mja         V------TDNYSL--SLKLPDKEGIIKF----L-VDENDFNYDRVK----KHVD-----------------------------------
FEN_Cter_Pyf         V------TDNYNL--VWRDPDEEGILKF----L-CDEHDFSEERVK----NGLE---RLKKAIKSGKQSTLESWFK-----------
FEN_Cter_Pyh         V------TNEYSL--SWKEPDEEGILKF----L-CDEHNFSEERVK----NGIE---RLKKAIKAGRQS------------------
FEN_Cter_Arf         V------TDDYRI--EFREPDFEKAIEF----L-CEEHDFSRERVE----KALE---KLK---------------------------
FEN_Cter_Hs          VL----DPESVEL--KWSEPNEEELIKF----M-CGEKQFSEERIR----SGVK---RLSKSRQGSTQGRLDDFFKVTGSLSSAKRKE
SAM1_PF3D7_1302100   K-------DRVSI---RRYVRKNQNRYN----Y-FLIEERVQDNIV----NRIS--DRLISYCTDKE--------------------
SAM2_PF3D7_1302100   I------MNDKKL--IRMLFDTYEYVKD----V-KFTDDQYKDAAA----RISQ---FLIDV-------------------------
PWI_PRPF3_Hs         V-------VTAALNCVGKGMDKKKAADH----L-KPFLDDSTLRFVD---KLFE---AVEEGRSSRHSKSSS------------DRSR
PWI_SWM_Dm           I-A---------L--LKKDKSDKDLKRIMIEQL-DVFLSEETTRFVE---RLFD---AIASE-------------------------
SAM_PF3D7_1349300    I-------RGYHI----LKLTDKELKKL---NI------------------------------------------------N-----
```

Supporting Information 7 – EMBOSS Needle alignment *Pf*pTKL vs *Pb*pTKL

```
PF3D7_1106800       1 MGNTLDSNKPKNFVTYADYKYIGKLNNKNEHHGIGIILYNSGESFYGSFI     50
                      ||| ::|.:.||::.:..|.|.|.||..|..||.|::||.:|.||:|.||
PBANKA_094010       1 MGN-INSIQTKNYIKFEKYYYAGDLNVDNLPHGRGLMLYENGNSFFGHFI     49

PF3D7_1106800      51 NGKKEGKGIYIDKNLTRYINTWVDNKVFGKVKVVPYNSNRVYYFYYKNYM    100
                      ||||.|||||||||||:||:.|..:.:..||||..::|:.||.|||||.:
PBANKA_094010      50 NGKKHGKGIYIDKNLTKYISKWKYDHISNKVKVKQFDSDIVYLFYYKNGL     99

PF3D7_1106800     101 IEKCIYFDNNINNKESHHKNNIYNNYDNNSYNNNSCDDEEKRKYPIGVTK    150
                      |:.|..:: .|:||:...|..          |:...:|.|.:...|...|
PBANKA_094010     100 IDHCKVYE-YISNKKKKKKKK----------NDGILEDSEIQNRSINFEK    138

PF3D7_1106800     151 FKEDLSNYIHSTHIMKKNNKLFNKNDNEYNIFSSSLSYSSDSENINLLDI    200
                      ....:........|:.|..::.:..:|:||          ::::.:.:|.
PBANKA_094010     139 DNIMIIQNCQEKSIVNKKEEIIDIPNNKYN----------ENDDTDKVDN    178

PF3D7_1106800     201 LKKKKNKKNKKNKKKKNKKTKNTQILSCTQHKMYEHNMNESNFTKKDNVN    250
                      ..||.|.:..:|...|.||.|       |..:|  :::|:|.|..    :
PBANKA_094010     179 FSKKINLEQSQNNMLKYKKKK-------TFDEM--NSLNDSIFCS----S    215

PF3D7_1106800     251 CEHTDKM----NISLHEKNDKK-NEKKNEKKNKKKKLFKY---------F    286
                      ||.|.|.    .||..||.:|: |::::|.|..|:....:         |
PBANKA_094010     216 CESTSKSIGSDYISHFEKKEKQINKQEDELKKSKENYMNHSEYSNSSTNF    265

PF3D7_1106800     287 SNN-------------IENLI-------IENYQTWSLREVIQWLMLCNVP    316
                      .||             ::|:|       ||||:.|:..:|.|||.|||||
PBANKA_094010     266 ENNKIKDISDESIMLRLKNIINNNTDLKIENYELWNKEQVAQWLSLCNVP    315

PF3D7_1106800     317 VKWLISFYKNNITGDKLKYININTIRNELGIIAYGHAIKILQLIKNLQVM    366
                      :||.:|.|||||.|.:|..:|:..|||:|||::||.|||.|||||||:|.
PBANKA_094010     316 MKWALSVYKNNINGQRLNNLNLYFIRNKLGILSYGQAIKFLQLIKNLRVT    365

PF3D7_1106800     367 AYNKKFNNLIQIEEYKNYIRQKENTNKNIKKGKNIKKEKKKKKEKNIKKE    416
                      |||.:|:|.:.:|||:.|::               ||.||||..:|...|
PBANKA_094010     366 AYNTRFSNTLNLEEYEIYLK---------------KKMKKKKMRENKDGE    400

PF3D7_1106800     417 ---------KKKKKKETKKFNNMDKKYIDLAIHKNVKNIQNDTFYNKHEN    457
```

```
                                   :..:|...|...|..|..||      .||..:.:|.:|.:|
PBANKA_094010     401 YTATTSSQAEDFQKNNQKIIMNQIKSMID------QKNDDDKSFSDKIKN    444

PF3D7_1106800     458 IY-----NCKNQTNFIYQNDSEIKKIMNKKKVSFEY---DNN-----EEK    494
                      .:     |.:|..|.:      |..|.||.|..|:.   :||     |:.
PBANKA_094010     445 GFFKGNTNIRNLQNLV------INSIWNKSKDEFKIPIDENNVPMNIEKG    488

PF3D7_1106800     495 KKKNIIKFI--KNNKSLQNSNGEYYLINHLSKGICSDSIFYKSSQSKSSS    542
                      |:.|....|  |..|.|:|.     .|||:.|.|   |:..|.:.:.:.|
PBANKA_094010     489 KELNTNDQITGKKKKKLKNK-----YINHIQKNI---SMLKKLNNASNES    530

PF3D7_1106800     543 QLSSPLSSPLSSPSPSSSPSSSPSSSPSSSPSSSPSPSSSPSPSSSPSSS    592
                      |.|:...:.:.|.|                     |.||..:..|..||.
PBANKA_094010     531 QSSNESVTQILSTS---------------------SESSFANIHSGLSSK    559

PF3D7_1106800     593 PSSSPSSPPSPLSYKDNFPISSSCSSLERLPSYEKKLLSSSQSNIEHIKN    642
                      ........|.|:.                 |:.||:
PBANKA_094010     560 MLFHDKLEPEPIK-----------------PNKEKE--------------    578

PF3D7_1106800     643 LPLDVLSNNNSSANIKIKKSKSKYNNDK---KEQKKLPLILNKSSSEFSP    689
                            .||.:.:.|...|:::...||.   |.|.:||    ||......
PBANKA_094010     579 ------ENNPNLSPIINSKNETNLLNDSNPTKLQDELP----KSPCSIDS    618

PF3D7_1106800     690 SHSYTSKSYHYNIKPSLQSSSNNSSDSSYSISSTCSSSSSYVSSLYSNRS    739
                                       :|||..||::|...||.||..|.:.
PBANKA_094010     619 -----------------ESSSEKSSETSSETSSFCSEHSEFA--------    643

PF3D7_1106800     740 NDILNFY-RNKIIKYCNNIYMNTKLAYSYMNGFIIPHEDLIFIHPIENYY    788
                          ||: .|||:||.||||:|:.||:||:..||||.|:|.|::.|.|||
PBANKA_094010     644 ----NFHNNNKIVKYSNNIYINSSLAFSYIYSFIIPPENLTFLYQIRNYY    689

PF3D7_1106800     789 MDNTNEKNNINNPYTKEKIMNHNFSFNTKNNTSFID---INTNI------    829
                      :.:.....|.||.      ::...|||...|...|.   ||..|
PBANKA_094010     690 VRDVENDLNPNNE------LDFCDSFNFYKNCEIIKNRIINPGIRNCSYA    733

PF3D7_1106800     830 -----FSSNKQQNINNFG---------KYKKMKSRMFKGKYMGKEVAIKI    865
                           |..||.:...|:.         |.:|||||:|:|:||||:||||:
PBANKA_094010     734 SNHSKFQQNKNRKYFNYSDCKNERKKTKPQKMKSRVFRGRYMGKDVAIKV    783

PF3D7_1106800     866 LVGKIKNFKKLHQILYNLYNLRHSNLVLIMGVSIHYPFVFIIYEYMKNKC    915
                      |||.||||.|.|::||.||.|||:|:.|||||||.||||||||||:||.|
PBANKA_094010     784 LVGNIKNFTKFHKVLYKLYILRHTNIALIMGVSISYPFVFIIYEYIKNLC    833

PF3D7_1106800     916 LFSYLHCIKYKHVYISTFLQRYKTLLHITQQEKIKKTNNINNNNNINHNN    965
                      |||||||:||||:|:|..|:.|        |:|....|....||.::.:.
PBANKA_094010     834 LFSYLHCVKYKHIYVSKLLKYY--------QKKFINQNFQQQNNTMSSDR    875

PF3D7_1106800     966 INNNNINHNNINHNNINNNNINNNNINYNKDYNNKKKKEDEQHNIEHQDT   1015
                      ...:|.::..|   |.::.||..|.:...|..||.|.|..|::|::.:..
PBANKA_094010     876 KYISNDDNEKI---NFDSRNILRNKLLEIKCKNNAKNKITEKNNLKDEQI    922
```

```
PF3D7_1106800      1016 FIDLPEKSNISSDDNNSTDISQIQKENFHFLNKKIEENKNIIYDDHTSTL   1065
                        :             ::||.|..:...:.:..|.|:   |||
PBANKA_094010       923 Y-------------SSSTSIKSLDTSSSNMNNTKL---KNI---------    947

PF3D7_1106800      1066 SDHSIHNINKSYDNVYKNKM-NIFHYQHNVLCGAYDNNDNNINDNDIYCN   1114
                              |.||   |.|.||: ::|..::|:|||.|           .|..
PBANKA_094010       948 ------NFNK---NRYINKIHSMFRNKNNILCGNY-----------YYLF    977

PF3D7_1106800      1115 NIYDNNINDNHIYCNNINDNHIYCNNINDNHIYCNNIYDHHKN---TSLN   1161
                        ....|||:.:|                            .|||   |...
PBANKA_094010       978 RKKKNNISISH----------------------------EHKNSDRTIFT    999

PF3D7_1106800      1162 SKEQNTDHNIEQINECNKYASETKYNIKKSNLKNNIISHKNFQKCN---Q   1208
                        ::.||.                         |||.|...|..:|.|   :
PBANKA_094010      1000 NESQNL-------------------------LKNKISQKKINKKLNFKAK   1024

PF3D7_1106800      1209 IQMNQPYTFPPYQKELSSYLKNEKIKRKRKVLFSYLKTHIHFNSQQINDQ   1258
                        |::|:||.|||.|::.:.||  ||.|:|:|:||||||||.:|.|::.:.:
PBANKA_094010      1025 IKINRPYAFPPLQEDFNFYL--EKKKKKKKILFSYLKTHSYFKSKKCDSR   1072

PF3D7_1106800      1259 HNRLSVQKIMKIITDVTLACTYLEKEKMSPINLKPTNILLDESLNAKISD   1308
                        .|:||..:|||||.|:||||:||||:|:..|||||||||||.||||||||
PBANKA_094010      1073 KNKLSDHQIMKIIMDITLACSYLEKQKVRWINLKPTNILLDGSLNAKISD   1122

PF3D7_1106800      1309 FGISKIENCLDMNIDYSYKISSNSVIKINKKEYEQKKAKKIKIVNKNNND   1358
                        |||.:||.|||:||||||.:..|:|||.|.|.: :.|.||||||||.:.|
PBANKA_094010      1123 FGIKEIEQCLDINIDYSYIVFPNNVIKFNNKHF-KNKIKKIKIVNKGSED   1171

PF3D7_1106800      1359 LLYLYDHNNNVYKYNTQYIDVTYNNSYPSIFYWTPPEILRGKKNKKFYSD   1408
                        :|:::...|::|||||:.|:|:.|....|:|:||.||||:||::...|||
PBANKA_094010      1172 MLHVFSSKNHIYKYNTREINVSSNTHNSSVFFWTSPEILKGKQSPSLYSD   1221

PF3D7_1106800      1409 IYAFGIILWEMLSNDIPYNYPFASHIMAVVGYANEELSFNNIPVSIQSLI   1458
                        :||:||||||:::|.:|:||.|.||::|.||||.|.|.|.|||..|::||
PBANKA_094010      1222 VYAYGIILWELMTNSVPFNYRFKSHLLASVGYAKESLPFQNIPPFIKNLI   1271

PF3D7_1106800      1459 KACVNRNKYKRPTFEHILKTISTLYQKANTKVEDALISFMDGT   1501
                        |:|:|||||||||||:.||..||.:|::.|.|.||||:|||||
PBANKA_094010      1272 KSCINRNKYKRPTFDRILIEISMIYEQINPKEEDALMSFMDG-   1313
```

Length: 1593

Identity: 577/1593 (36.2%)

Similarity: 814/1593 (51.1%)

Gaps: 372/1593 (23.4%)

Score: 2367.5

Supporting Information 8 – Sequencing data – pTKL_RVxF1

RAW DNA sequencing data

>PF3D7_1106800_SAM&RVxF1

AATTTAATTATTGAAAATTATCAGACATGGTCTTTAAGAGA---AGTAATTCAATGGCTTAT-----------------G----------
--TTAT--GCAATG-
--TACCTGTTAAATGGTTAATAAGTTTTTATAAAA----
-----------ATAAT--------ATAACAGGTGATAAATT---AAAATATATTAATATAAAT-
ACTATAAGAAATGAATTGGGGATTATTGCTTATGGACATGCAATCAAAATATTACAATTA---------------------
ATCAAAAATCTACAAGTT-ATGGCAT--ATAATAAAAAATTCAATAACTTAATACAAATAGAAGAATAT-
AAAAATTATATAAGACAAAAGGAAAACACAAACAAAAATATAAAAAAAGGGAAAAATATAAAAAAAGAAAAAAA
GAAAAAAAAAGAAAAAAATATAAAAAAAGAAAAAAAGAAAAAAAAGAAAGAAACAAAAAAATTCAACAACATGG
ATAAAAAATATATAGATTTAGCTATTCATAAAAATGTTAAGAATATACAAAATGATACTTTTTATAATAAGCATGAA
AATATTTACAATTGTAAGAATCAAACAAATTTTATTTATCAAAATGATTCTGAAATAAAAAAAATCATGAATAAAAA
GAAAGTATCATTTGAATATGATAATAATGAAGAAAAAAAAAAAAAAAAATATTATTAAATTTATAAAAAATAATAAA
TCATTACAAAATTCAAATGGGGAATATTATTTAATAAATCACTTATCAAAAGGTATATGTAGTGATAGCATATT------
----

----------TTATA---AA-------------------------

>RVxF1_pETduET_clone4_petDown

GACTTGGATAACGTTAACTGTTACACATCATCACTG-
GAGCTGACTATTTTCCGAGGCGTATTCATGCCATACGCGAAAAGTTTTGCGCATTCGATGGGTCCGGGGATCTGGA
CGGGTTGCCAGTATTCGGACTGCTGCATTAAGAAGCAGACCAGTAGTAGGGTTGAGAGCCGATGAGCACGCCGCC
GCAAGGAAATGTGCATGCAAGGAGATGGCGCACAACAGTCCCCCGGCCTCGGGGCCTGCCACCATACCCACGCCG
AAACAAGCGCTCATGAGCCCGAAGTGGCGAAGCCCGATCTTCCCCATCGGTG-----------
ATGTCGGCGATATAGGCGCCAGCAACCGCACCTGTGGCGCCGGTGATGCCGGCCACGATGCGTCCGGCGTAGAG
GATAGAGATCGATCTCGATCCCGCGAAATTAATACGACTCACTATAGGGGAATTGTGAGCGGATAACAATTCCCCT
CTAGAAATAATTTTGTTTAACTTTAAGAAGGAGATATACCATGGGCAGCAGCCATCACCATCATCACCACAGCCAG
GATCCGAATTCCAATAACTTAATACAAATAGAAGAATAT-
AAAAATTATATAAGACAAAAGGAAAACACAAACAAAAACATAAAAAAAGGGAAAAATATAAAAAAAGAAAAAAA
TATAAAAAAAGAAAAAAATATAAAAAAAGAAAAAAAGAAAAAAAAGAAAGAAACAAAAAAATTCAACAACATGG
ATAAAAAATATATAGATTTAGCTATTCATAAAAATGTTAAGAATATACAAAATGATACTTTTTATAATAAGCATGAA
AATATTTACAATTGTAAGAATCAAACAAATTTTATTTATCAAAATGATTTTGAAATAAAAAAAATCATGAATAAAAA
GAAAGTATCATTTGAATATGATAATAATGAAGAAAAAAAAAAAAAAAAATATTATTAAATTTATAAAAAATAATAAA
TCATTACAAAATTCAAATGGGGAATATTATTTAATAAATCACGTA-CAAAAAATAAT------------------------------------
----

>RVxF1_pETduET_clone4_petUp

-----------------------CCTC--

-----------------TTT--------------
TTATAAAAACTTATATAAGACAAAAGGAAAACACAAACAAAAACATAAAAAAAGGGAAAAATATAAAAAAAGAAA
AAAATATAAAAAAAGAAAAAAATATAAAAAAAGAAAAAAAGAAAAAAAAGAAAGAAACAAAAAAATTCAACAAC
ATGGATAAAAAATATATAGATTTAGCTATTCATAAAAATGTTAAGAATATACAAAATGATACTTTTTATAATAAGCA
TGAAAATATTTACAATTGTAAGAATCAAACAAATTTTATTTATCAAAATGATTCTGAAATAAAAAAAATCATGAATA
AAAAGAAAGTATCATTTGAATATGATAATAATGAAGAAAAAAAAAAAAAAAAATATTATTAAATTTATAAAAAATAA
TAAATCATTACAAAATTCAAATGGGGAATATTATTTAATAAATCACTTATCAAAAGGTATATGTAGTGATAGCATAG
CGGCCGCATAATGCTTAA--GTCGAACAGAAAGTAATCGTATTGTACACGGCCGCATAATCGA--
AATTAATACGACTCACTATAGGGG---------AATTGTGAGCGGATAACAA--------
TTCCCCATCTTAGTATATTAGTTAAGTATAAGAAGGAGATATACATATGGCAGATCTCAATTGGATATCGGCCGGCC
ACGCGATCGCTGACGTCGGTACCCTCGAGTCTGGTAAAGAAACCGCTGCTGCGAAAT--TTGAACGCCAG--C---
ACATGGACTCGTCTACTAGCGCAGCTTAAT----------------TAACCTA---
--------------GGCTGCTGCCACCGCTGAG-CAATAACTAGCATAACCCCTTGGGGCCTCTAAACCGGGT--
CTTGAGGGTTTTTTGCTGAAAG--
GAGGAACTATATCCGGATTGGCGAATGGGACGCGCCCTGTAGCGGCGCATTTAGCGCGGCGGGTGT-GGTG------
GTTACGCG---------CAGCGTGACGCTACACCTGGCTAGCGTCCTAAGCG-----------------
CCCGCTTCGTTCGCTTTCTGCGTACATCCGGCCACGTTCG----------CCGGCTTTCCCGTT----------------------
CAAGCTCTAAATCGGGGGGGGA-CTCCCCT---------ATTTATGGGG

>RVxF1_pGEX_clone4_F

------------------TCGGACGTT----TT--GGGT---GGTG---
--GCGACC-------------------------------------
---ATCCTCCAAATCGGAT-----------CTGGAA----------------
GTTCTGTTCCAGGGGCCCCTGGGAT--
---CC----------------------
CCGAATTCCAATAACTTAATACAAATAGAAGAATAT-
AAAAATTATATAAGACAAAAGGAAAACACAAACAAAAATATAAAAAAAGGGAAAAATATAAAAAAAGAAAAAAA
TATAAAAAAAGAAAAAAATATAAAAAAAGAAAAAAAGAAAAAAAAGAAAGAAACAAAAAAATTCAACAACATGG
ATAAAAAATATATAGATTTAGCTATTCATAAAAATGTTAAGAATATACAAAATGATACTTTTTATAATAAGCATGAA
AATATTTACAATTGTAAGAATCAAACAAATTTTATTTATCAAAATGATTCTGAAATAAAAAAAATCATGAATAAAAA
GAAAGTATCATTTGAATATGATAATAATGAAGAAAAAAAAA-
AAAAAAATATTATTAAATTTATAAAAAATAATAAATCATTACAAAATTCAAATGGGGAATATTATTTAATAAATCACT
TATCAAAAGGTATATGTAGTGATAGCATAGCGGCCGCATCGTGACTGACTGACGATCTGCCT-

CGCGCGTTTCGGTGATGACGGTGAAAACCTCTGACACATGCAGCTCCCGGAGACGGTCACAGCTTGTCTGTAAGC
GGATGCCGGGAGCAGACAAGCCCGTCAGGGCGCGTCAGC---------------------------------------
GGGTGTTGGCGGGTGTCGGG-GCGCAGCCAT---GACCC---AGTCACGTAGCGATAGCGGAGT-
GTATAATTCTTGAAGACGAGAGGGCCTCGT-
GATACGCCTATTTTTATAGGTTAATGTCATGATAATAATGGTCTCTTAGACGTCAGGTGGCACTTTTCGGAGAAATG
TGCGCGGAACCCCTATTTGTTTATTTTTCTAAATACATTCAAATATGTATCCGCTCATGAGACAATACCCCTGATAAA
TGCTTCATTATATTGAAAAAGGAAGAGTATGAGTATTCAACATTTCCGTGTCGCCCTTATTCCTTTTTTGCGGCTTTT
TGCCTTCCTGTTTTTGCTCACCAAGAACG-
CTGGTGAAAGTAAAGATGCTGAAGATCGGTGGGGGGGCCGAGTGGGTTCATCCGAATGG-
ATCCAAAGCGGTAAATCCTTGAAGTTTCCCCC--
CAGAAACTTTCAAGGAAGAACCTTTTAAGTTCGGTATGGGGGGCGGGTTATCCCGGTGGCCGGGAAAGAATTCG
GCCCCAACCATTTCAAAGGTTGGTGATCCCCCCCCCAAAAAACCTTTGGGGG

Deduced protein sequences

>PF3D7_1106800_RVXF1

YKNYIRQKENTNKNIKKGKNIKKEKKKKKEKNIKKEKKKKKKETKKFNNMDKKYIDLAIHKNVKNIQNDTFYNKHENIYNC
KNQTNFIYQNDSEIKKIMNKKKVSFEYDNNEEKKKKNIIKFIKNNKSLQNSNGEYYLINHLSKGICSDSIFYK

>pTKL_RVxF1_6His

YKNYIRQKENTNKNIKKGKNIKKEKNIKKEKNIKKEKKKKKKETKKFNNMDKKYIDLAIHKNVKNIQNDTFYNKHENIYNC
KNQTNFIYQNDFEIKKIMNKKKVSFEYDNNEEKKKKNIIKFIKNNKSLQNSNGEYYLINHLSK----------

>pTKL_RVxF1_GST

YKNYIRQKENTNKNIKKGKNIKKEKNIKKEKNIKKEKKKKKKETKKFNNMDKKYIDLAIHKNVKNIQNDTFYNKHENIYNC
KNQTNFIYQNDSEIKKIMNKKKVSFEYDNNEEKKKKILLNL-------------------------------

Supporting Information 9 – Sequencing data pTKL_SAM&RVxF1

Raw DNA sequencing data

>PF3D7_1106800_SAM&RVxF1

AA---

---T--------

TTAATTATTGAAAATTATCAGACATGGTCTTTAAGAGAAGTAATTCAATGGCTTATGTTATGCAATGTACCTGTTAA
ATGGTTAATAAGTTTTTATAAAAATAATATAACAGGTGATAAATTAAAATATATTAATATAAATACTATAAGAAATG
AATTGGGGATTATTGCTTATGGACATGCAATCAAAATATTACAATTAATCAAAAATCTACAAGTTATGGCATATAAT
AAAAAATTCAATAACTTAATACAAATAGAAGAATATAAAAATTATATAAGACAAAAGGAAAACACAAACAAAAATA
TAAAAAAAGGGAAAAATATAAAAAAAGAAAAAAAGAAAAAAAAAGAAAAAAATATAAAAAAAGAAAAAAAGAA
AAAAAAGAAAGAAACAAAAAAATTCAACAACATGGATAAAAAATATATAGATTTAGCTATTCATAAAAATGTTAAG
AATATACAAAATGATACTTTTTATAATAAGCATGAAAATATTTACAATTGTAAGAATCAAACAAATTTTATTTATCAA
AATGATTCTGAAATAAAAAAAATCATGAATAAAAAGAAAGTATCATTTGAATATGATAATAATGAAGAAAAAAAAA
AAAAAAATATTATTAAATTTATAAAAAATAATAAATCATTACAAAATTCAAATGGGGAATATTATTTAATAAATCACT
TATCAAAAGGTATATGTAGTGATAGCATA--
-------------------------T------------------T-T----------------TAT--
----
--A---AA

>SAM&RVxF1_STOP_revT7

AGGGG-----CCG----AAGC---

TGATTTCAGAGGAGGACCTGCATATGGCCATGGAGGCCGAATTTGACAAAATGAATATTTCCCTTCATGAAAAAAA
TGATAAAAAAAAATGGGAA-
AAAAATGGGAAGAAAAATAAGAAAAAAAAACTTTTTAAATATTTTTCAAATAATATAGAAAATTTAATTATTGAAAA
TTATCAGACATGGTCTTTAAGAGAAGTAATTCAATGGCTTATGTTATGCAATGTACCTGTTAAATGGTTAATAAGTT
TTTATAAAAATAATATAACAGGTGATAAATTAAAATATATTAATATAAATACTATAAGAAATGAATTGGGGATTATT
GCTTATGGACATGCAATCAAAATATTGCAATTAATCAAAAATCTACAAGTTATGGCATATAATAAATAATTCAATAA
CTTAATACAAATAGAAGAATATAAAAATTATATAAGACAAAAGGAAAACACAAACAAAAATATAAAAAAAGGGAA
AAATATAAAAAAAGAAAAAAATATAAAAAAAGAAAAAAATATAAAAAAAGAAAAAAAGAAAAAAAAGAAAGAAA
CAAAAAAATTCAACAACATGGATAAAAAGTATATAGATTTAGCTATCCATAAAAATGTTAAGAATATACAAAATGA
TACTTTTTATAATAAGCATGAAAATATTTACAATTGTAAGAATCAAACAAATTTTATTTATCAAAATGATTCTGAAAT
AAAAAAAATCATGAATAAAAAGAAAGTATCATTTGAATATGATAATAATGAAGAAAAAAA-
AAAAAAAATATTATTAAATTTATAAAAAATAATAAATCATTACAAAATTCAAATGGGGAATATTATTTAATAAATCA
CTTATCAAAAGGTATATGTAGTGATAGCATAGGATCCGTCGACCTGCAGCGGCCGCATAACTAGCATAACCCCTTG
GGGCCTCTAAACGGGTCTTG-
AGGGGTTTTTTGCGCGCTTGCAGCCAAGCTAATTCCGGGCGAATTTCTTATGATTTATGATTTTTATTATTAAATAAG
TTATAAAAAAAAATAAGG------------------------------TTT-ACAAAT--

---TAA---AT

>SAM&RvXF1_STOP_FT7
GGACATTATGCAGGAGAGAGC---

TGATCTCAGAGGAGGACCTGCATATGGCCATGGAGGCCGAATTCGACAAAATGAATATATCCCTTCATGAAAAAAA
TGATAAAAAAAATGAGAAAAAAAAATGAGAAGAAAAATAAGAAAAAAAAAACTTTTTAAATATTTTTCAAATAATATA
GAAAATTTAATTATTGAAAATTATCAGACATGGTCTTTAAGAGAAGTAATTCAATGGCTTATGTTATGCAATGTACC
TGTTAAATGGTTAATAAGTTTTTATAAAAATAATATAACAGGTGATAAATTAAAATATATTAATATAAATACTATAA
GAAATGAATTGGGGATTATTGCTTATGGACATGCAATCAAAATATTGCAATTAATCAAAAATCTACAAGTTATGGC
ATATAATAAATAATTCAATAACTTAATACAAATAGAAGAATATAAAAATTATATAAGACAAAAGGAAAACACAAAC
AAAAATATAAAAAAAGGGAAAAATATAAAAAAAGAAAAAAATATAAAAAAAGAAAAAAATATAAAAAAAGAAAA
AAAGAAAAAAAAAGAAAGAAACAAAAAAATTCAACAACATGGATAAAAAGTATATAGATTTAGCTATCCATAAAAA
TGTTAAGAATATACAAAATGATACTTTTTATAATAAGCATGAAAATATTTACAATTGTAAGAATCAAACAAATTTTAT
TTATCAAAATGATTCTGAAATAAAAAAAATCATGAATAAAAAGAAAGTATCATTTGAATATGATAATAATGAAGAA
AAAAA-
AAAAAAAATATTATTAAATTTATAAAAAATAATAAATCATTACAAAATTCAAATGGGGAATATTATTTAATAAATCA
CTTATCAAAAGGTATATGTAGTGATAGCATAGGATCCGTCGACCTGCAGCGGCCGCATAACTAGCATAACCCCTTG
GGGCCTCTAAACGGGTCTTGGAGGGGTTTTTTGCGCGCTTGCAGCCAAGCTAATTCCGGGCGAATTTCTTATGATTT
ATGATTTTTATTATTAAATAAGTTATAAAAAAAATAAGTGGAATCAAATTTTAAAGGGACTCTTAGGTTTTA-
AAACG---
--AAAATCC
>SAM&RvXF1_cloneJ3_R
CCC-----------------
CAACCCCAAAGTTTCCGGTGATTAGGGCAATTTGCCAGGATGGGATCCAAGCTTGAAAGACTGGAACAGGTATTTT
CCGGATTTTCCTCGGAGAACCTTGCAATGATTTGAAAATGGATTCTTCCAGGGATTTAAAGCATTGTTACCAGGATT
ATTGTCCAAGATAATGTGAATAAAGATGCCGTCACAGATAGATTGGCTTCAGTGGAGACTGATATGCTTTTAACATT
GAGACAGCATAGAATAAGTGGGACATCATCATCGGAAGGGAGTAGTACCAAAGGTCAAAGACAGTTGACTGTATC
GCCGGAATTTGTAATACGACTCACTATAGGGCGAGCCGCCATCATGGAGGAGCAGAAGCTGATTTCAGAGGAGGA
CCTGCATATGGCCATGGAGGCCGAATTCGACAAAATGAATATATCCCTTCATGAAAAAAATGATAAAAAAAATGAG
AAAAAAAATGAGAAGAAAAATAAGAAAAAAAAAACTTTTTAAATATTTTTCAAATAATATAGAAAATTTAATTATTGA
AAATTATCAGACATGGTCTTTAAGAGAAGTAATTCAATGGCTTATGTTATGCAATGTACCTGTTAAATGGTTAATAA
GTTTTTATAAAAATAATATAACAGGTGATAAATTAAAATATATTAATATAAATACTATAAGAAATGAATTGGGGATT
ATTGCTTATGGACATGCAATCAAAATATTGCAATTAATCAAAAATCTACAAGTTATGGCATATAATAAAAAATTCAA
TAACTTAATACAAATAGAAGAATATAAAAATTATATAAGACAAAAGGAAAACACAAACAAAAATATAAAAAAAGG
GAAAAATATAAAAAAAGAAAAAAATATAAAAAAAGAAAAAAATATAAAAAAAGAAAAAAAGAAAAAAAAAGAAAG
AAACAAAAAAATTCAACAACATGGATAAAAAGTATATAGATTTAGCTATCCATAAAAATGTTAAGAATATACAAAA
TGATACTTTTTATAATAAGCATGAAAATATTTACAATTGTAAGAATCAAACAAATTTTATTTATCAAAATGATTCTGA
AATAAAAAAAATCATGAATAAAAAGAAAGTATCATTTGAATATGATAATAATGAAGAAAAAAAAAAAAAAAAATATT
ATTAAATTTATAAAAAATAATAAATCATTACAAAATTCAAATGGGGAATATTAT-TAATAAATCACT-
ATCAAAAGGGG---

--GG---GG
>SAM&RxF1_cloneJ3_F
CCC-----------------C---

--CG-TTT-------------
TATATAGT--------------ATGATAAAA--------GCCAATCGTGA----------------------AA-
AAAAATGAGAAGAAAAATAAGAAAAAAAAACTTTTTAAATATTTTTCAAATAATATAGAAAATTTAATTATTGAAAA
TTATCAGACATGGTCTTTAAGAGAAGTAATTCAATGGCTTATGTTATGCAATGTACCTGTTAAATGGTTAATAAGTT
TTTATAAAAATAATATAACAGGTGATAAATTAAAATATATTAATATAAATACTATAAGAAATGAATTGGGGATTATT
GCTTATGGACATGCAATCAAAATATTGCAATTAATCAAAAATCTACAAGTTATGGCATATAATAAAAAATTCAATAA
CTTAATACAAATAGAAGAATATAAAAATTATATAAGACAAAAGGAAAACACAAACAAAAATATAAAAAAAGGGAA
AAATATAAAAAAAGAAAAAAATATAAAAAAAGAAAAAAATATAAAAAAAGAAAAAAAGAAAAAAAAGAAAGAAA
CAAAAAAATTCAACAACATGGATAAAAAGTATATAGATTTAGCTATCCATAAAAATGTTAAGAATATACAAAATGA
TACTTTTTATAATAAGCATGAAAATATTTACAATTGTAAGAATCAAACAAATTTTATTTATCAAAATGATTCTGAAAT
AAAAAAAATCATGAATAAAAAGAAAGTATCATTTGAATATGATAATAATGAAGAAAAAAA-
AAAAAAAATATTATTAAATTTATAAAAAATAATAAATCATTACAAAATTCAAATGGGGAATATTATTTAATAAATCA
CTTATCAAAAGGTATATGTAGTGATAGCATAGGATCCGTCGACCTGCAGCGGCCGCATAACTAGCATAACCCCTTG
GGGCCTCTAAACGGGTCTTG-
AGGGGTTTTTTGCGCGCTTGCAGCCAAGCTAATTCCGGGCGAATTTCTTATGATTTATGATTTTTATTATTAAATAAG
TTATAAAAAAAATAAGTGTATACAAATTTTAAAGTGACTCTTAAGTTTTA-
AAACGAAAATTCTTATTCTTGAGTAACTCTTTCCTGGAGGTCAGGTTGCTTTCTCAGGTATAGCATGAGGTCCCTCTT
ATTGACCAACCCTCTACCGGCATGCAAGCTTGGCGTAATCATGGGCCTAAGCTGTTCCCTGGTGGAAATTGTAACC
CGCTCCAATTCCCACCACCTACCAGCCCGGAACCTAAAGTGAAAGCCGGGGTGCCCAAAGAGGAGCCATCCCATTA
TTTGGCTCGCCTCATGGCCCTTCA---AA

Proteins

>PF3D7_1106800_SAM&RVxF1
LIIENYQTWSLREVIQWLMLCNVPVKWLISFYKNNITGDKLKYININTIRNELGIIAYGHAIKILQLIKNLQVMAYNKKFNNL
IQIEEYKNYIRQKENTNKNIKKGKNIKKEKKKKKEKNIKKEKKKKKKETKKFNNMDKKYIDLAIHKNVKNIQNDTFYNKHEN
IYNCKNQTNFIYQNDSEIKKIMNKKKVSFEYDNNEEKKKKNIIKFIKNNKSLQNSNGEYYLINHLSK
>pTKL_SAM&RVxF1_pGBTK7
LIIENYQTWSLREVIQWLMLCNVPVKWLISFYKNNITGDKLKYININTIRNELGIIAYGHAIKILQLIKNLQVMAYNKKFNNL
IQIEEYKNYIRQKENTNKNIKKGKNIKKEKNIKKEKNIKKEKKKKKKETKKFNNMDKKYIDLAIHKNVKNIQNDTFYNKHEN
IYNCKNQTNFIYQNDSEIKKIMNKKKVSFEYDNNEEKKKKNIIKFIKNNKSLQNSNGEYY-------
>pTKL_SAM_STOP_pGBKT7
LIIENYQTWSLREVIQWLMLCNVPVKWLISFYKNNITGDKLKYININTIRNELGIIAYGHAIKILQLIKNLQVMAYNK--------
----

Supporting Information 10 – Sequencing data pTKL_KD_WT and mutants

Raw DNA sequencing data

>Synthetic_pTKL_KD_WT

GAATTCGACAATCACATTTACTGCAATAACATTTACGATCACCACAAAAACACGTCATTGAACTCGAAAGAACAAAA
TACGGACCATAACATCGAACAGATTAATGAGTGTAACAAATATGCCAGTGAAACCAAATACAACATTAAGAAATCG
AACCTGAAGAATAACATTATCTCGCATAAGAACTTCCAGAAATGCAATCAGATTCAGATGAACCAACCCTACACGTT
TCCACCATATCAGAAAGAACTGAGCTCTTATCTTAAAAACGAAAAGATCAAACGGAAACGCAAAGTGCTGTTTTCC
TACCTGAAAACCCATATTCACTTCAACAGTCAGCAGATTAACGATCAACACAATCGCTTATCTGTGCAGAAAATTAT
GAAAATCATTACTGATGTCACCCTTGCTTGCACATATCTGGAAAAGGAGAAAATGAGCCCCATTAATCTGAAACCTA
CCAATATCCTGTTGGACGAATCTCTGAATGCCAAAATTAGCGATTTCGGTATCAGCAAAATTGAGAATTGTCTGGAT
ATGAACATTGACTATTCATACAAAATTAGCTCCAACTCAGTCATCAAAATTAATAAAAAAGAATATGAGCAAAAGA
AAGCGAAGAAAATCAAAATTGTGAATAAAAACAATAATGACTTACTGTACCTGTACGATCACAATAACAACGTCTA
TAAGTACAATACCCAATATATCGACGTAACCTATAACAACAGTTATCCGAGCATCTTCTATTGGACTCCTCCGGAAA
TCTTACGTGGCAAAAAAAACAAGAAATTTTACAGCGATATTTACGCATTTGGGATCATCCTCTGGGAAATGCTGAG
TAATGATATTCCGTACAACTATCCGTTTGCATCCCATATTATGGCCGTTGTAGGCTATGCGAATGAAGAACTGTCAT
TCAACAACATTCCGGTTTCCATTCAGTCGTTGATCAAAGCGTGTGTTAATCGCAACAAATATAAGCGTCCGACGTTT
GAGCATATTCTGAAAACGATCTCTACACTCTATCAGAAAGCGAATACCAAAGTGGAAGATGCTCTTATCAGCTTTAT
GGATGGTACTCTCGAGCATCATCACCATCATCATTAAGGATCC--

>pTKL_KD_ND_clone3_R

--ATC----------------------------CGGAAGCAAAATCGGCCTTAACTTGGA--CGGTTAA--GGGGTACCAA---TTCCCGTG-
-ACCCAATCCACCTT----AGAATTGACCTGAAGAATACCTTT-TCTGCCATAAG-ACTTCCAGAAA-
GCAATCAGATTCAGATGAACCAACCTACCACGTTTCCACCATTTCAGAAGGAACTGAGTTCTTATCTT-
AAAACGAAAAGATCAAACGGAAACGCAAAGTGCTGT-TTCCTACCTGAAAACCCATA-
TCACTTCAACAGTCAGCAGATTAACGATCAACACAATCGCTTATCTGTGCAGAAAATTATGAAAATCATTACTGATG
TCACCCTTGCTTGCACATATCTGGAAAAGGAGAAAATGAGCCCCATTGATCTGAAACCTACCAATATCCTGTTGGAC
GAATCTCTGAATGCCAAAATTAGCGATTTCGGTATCAGCAAAATTGAGAATTGTCTGGATATGAACATTGACTATTC
ATACAAAATTAGCTCCAACTCAGTCATCAAAATTAATAAAAAAGAATATGAGCAAAAGAAAGCGAAGAAAATCAA
AATTGTGAATAAAAACAATAATGACTTACTGTACCTGTACGATCACAATAACAACGTCTATAAGTACAATACCCAAT
ATATCGACGTAACCTATAACAACAGTTATCCGAGCATCTTCTATTGGACTCCTCCGGAAATCTTACGTGGCAAAAAA
AACAAGAAATTTTACAGCGATATTTACGCATTTGGGATCATCCTCTGGGAAATGCTGAGTAATGATATTCCGTACAA
CTATCCGTTTGCATCCCATATTATGGCCGTTGTAGGCTATGCGAATGAAGAACTGTCATTCAACAACATTCCGGTTT
CCATTCAGTCGTTGATCAAAGCGTGTGTTAATCGCAACAAATATAAGCGTCCGACGTTTGAGCATATTCTGAAAACG
ATCTCTACACTCTATCAGAAAGCGAATACCAAAGTGGAAGATGCTCTTATCAGCTTTATGGATGGTACTCTCGAGCA
TCATCACCATCATCATTAAGGATCCTCTAGAGTCGACCTGCAGGCAAGCTTGGCACTGGCCGTCGTTTTACAACGTC

GTGACTGGGAAAACCCTGGCGTTACCCAACTTAATCGCCTTGCAGCACATCCCCCTTTCGCCAGCTGGCGTAATAGC
GAAGAGGCCCGCACCGATCGCCCTTCCCAACAGTGCGCAGCAGTAAGTGGAAGTCC

>pTKL_KD_DG_clone9_R

--CGGTG--ACCCA-------------------------------
---CCCTCCCGTTTCCCCCATTC--AGAAGGACTGAGTT-TTATTCT-
AAAACGAAAAGATCAAACGGAAACGCAAAGTGCTGT-
TTCCTACCTGAAAACCCATATTCACTTCAACAGTCAGCAGATTAACGATCAACACAATCGCTTATCT-
TGCAGAAAATTATGAAAATCATTACTGATGTCACCCTTGCTTGCACATATCTGGAAAAGGAGAAAATGAGCCCCAT
TAATCTGAAACCTACCAATATCCTGTTGGACGAATCTCTGAATGCCAAAATTAGCGGTTTCGAAATCAGCAAAATTG
AGAATTGTCTGGATATGAACATTGACTATTCATACAAAATTAGCTCCAACTCAGTCATCAAAATTAATAAAAAAGAA
TATGAGCAAAAGAAAGCGAAGAAAATCAAAATTGTGAATAAAAACAATAATGACTTACTGTACCTGTACGATCACA
ATAACAACGTCTATAAGTACAATACCCAATATATCGACGTAACCTATAACAACAGTTATCCGAGCATCTTCTATTGG
ACTCCTCCGGAAATCTTACGTGGCAAAAAAAACAAGAAATTTTACAGCGATATTTACGCATTTGGGATCATCCTCTG
GGAAATGCTGAGTAATGATATTCCGTACAACTATCCGTTTGCATCCCATATTATGGCCGTTGTAGGCTATGCGAATG
AAGAACTGTCATTCAACAACATTCCGGTTTCCATTCAGTCGTTGATCAAAGCGTGTGTTAATCGCAACAAATATAAG
CGTCCGACGTTTGAGCATATTCTGAAAACGATCTCTACACTCTATCAGAAAGCGAATACCAAAGTGGAAGATGCTC
TTATCAGCTTTATGGATGGTACTCTCGAGCATCATCACCATCATCATTAAGGATCCTCTAGAGTCGACCTGCAGGCA
AGCTTGGCACTGGCCGTCGTTTTACAACGTCGTGACTGGGAAAACCCTGGCGTTACCCAACTTAATCGCCTTGCAG
CACATCCCCCTTTCGCCAGCTGGCGTAATAGCGAAGAGGCCCGCACCGATCGCCC-TCCCAACAGTGCGCAGCGA-
AGTCGAAGTTCC

>pTKL_KD_KRIMAS_cloneB_R

--------------------------------AGAAGAACAAATCGGGCCCAAACTTGGACCGGTTAA-GGGGGTAACAAAT-
TGCCCGTG-AACCAAATCCACCTT---AAGATTGGACCTGAAGAATACCATT-TCTCGCATAAG-
ACTTCCAGAAATGCAATCAGATTCAGATGAACCAACCCTACACGTTTCCACCATATCAGAAAGAACTGAGCTCTTAT
CTT-
AAAACGAAAAGATCAAACGGAAACGCATAATGGCGTCTTCCTACCTGAAAACCCATATTCACTTCAACAGTCAGCA
GATTAACGATCAACACAATCGCTTATCTGTGCAGAAAATTATGAAAATCATTACTGATGTCACCCTTGCTTGCACAT
ATCTGGAAAAGGAGAAAATGAGCCCCATTAATCTGAAACCTACCAATATCCTGTTGGACGAATCTCTGAATGCCAA
AATTAGCGATTTCGGTATCAGCAAAATTGAGAATTGTCTGGATATGAACATTGACTATTCATACAAAATTAGCTCCA
ACTCAGTCATCAAAATTAATAAAAAAGAATATGAGCAAAAGAAAGCGAAGAAAATCAAAATTGTGAATAAAAACA
ATAATGACTTACTGTACCTGTACGATCACAATAACAACGTCTATAAGTACAATACCCAATATATCGACGTAACCTAT
AACAACAGTTATCCGAGCATCTTCTATTGGACTCCTCCGGAAATCTTACGTGGCAAAAAAAACAAGAAATTTTACAG
CGATATTTACGCATTTGGGATCATCCTCTGGGAAATGCTGAGTAATGATATTCCGTACAACTATCCGTTTGCATCCC
ATATTATGGCCGTTGTAGGCTATGCGAATGAAGAACTGTCATTCAACAACATTCCGGTTTCCATTCAGTCGTTGATC
AAAGCGTGTGTTAATCGCAACAAATATAAGCGTCCGACGTTTGAGCATATTCTGAAAACGATCTCTACACTCTATCA
GAAAGCGAATACCAAAGTGGAAGATGCTCTTATCAGCTTTATGGATGGTACTCTCGAGCATCATCACCATCATCATT

AAGGATCCGT---------------GGCAGCTCTAGAGCTAGC-------------------------GAATTCTTTGGTGAAAT-----------------
-----TGTTATCCGCTCACAATCCA-------------------CACATCAATGG-----------------------------------

>pTKL_KD_KRIMAS_cloneB_F

------------------------------CCACCGCT---
CGCGGTTGGCGGGTGTCGGG--GCTGGCTTAACTATGCGGCAT---
CAGAGCAGATTGTACTGAGAGTTTGGCAATTG----------------
GTCGACCTCGAGGGCGCGCCCGTAGAATTCGACAATCACATTTACTGCAATAACATTTACGATCACCACAAAAACA
CGTCATTGAACTCGAAAGAACAAAATACGGACCATAACATCGAACAGATTAATGAGTGTAACAAATATGCCAGTGA
AACCAAATACAACATTAAGAAATCGAACCTGAAGAATAACATTATCTCGCATAAGAACTTCCAGAAATGCAATCAG
ATTCAGATGAACCAACCCTACACGTTTCCACCATATCAGAAAGAACTGAGCTCTTATCTTAAAAACGAAAAGATCAA
ACGGAAACGCATAATGGCGTCTTCCTACCTGAAAACCCATATTCACTTCAACAGTCAGCAGATTAACGATCAACACA
ATCGCTTATCTGTGCAGAAAATTATGAAAATCATTACTGATGTCACCCTTGCTTGCACATATCTGGAAAAGGAGAAA
ATGAGCCCCATTAATCTGAAACCTACCAATATCCTGTTGGACGAATCTCTGAATGCCAAAATTAGCGATTTCGGTAT
CAGCAAAATTGAGAATTGTCTGGATATGAACATTGACTATTCATACAAAATTAGCTCCAACTCAGTCATCAAAATTA
ATAAAAAAGAATATGAGCAAAAGAAAGCGAAGAAAATCAAAATTGTGAATAAAAACAATAATGACTTACTGTACC
TGTACGATCACAATAACAACGTCTATAAGTACAATACCCAATATATCGACGTAACCTATAACAACAGTTATCCGAGC
ATCTTCTATTGGACTCCTCCGGAAATCTTACGTGGC-
AAAAAAACAAGAAATTTTACAGCGATATTTACGCATTTGGGATCATCCTCTGGGAAATGCTGAGTAATGATATTCC
GTACAACTATCCGTTTGCAT-CCATATTATGGCCGTTGTAGGCTATGCGAATGAAG-ACTGTCATTC-
ACAACATTCCGGTTTCCTTTC-GTCGTTGATCAAAGCGTG-GGTAATCGCA--
CAAAATAAACGTCCGAAGTTTGAGCA-ATTCTGAAAA-GATCTCTACTTTTTC---AAAAGCGAA-ACCAAGG-----------
----

>pTKL_KD_ND_clone3_F

ATTTGAGATTGGGCGAAAGATCCACGTATTGCCGCCACTATGGAAAACGCCCAGAAAGGTGAAATCATGCCGAAC
ATCCCGCAGATGTCCGCTTTCTGGTATGCCGTGCGTACTGCGGTGATCAACGCCGCCAGCGGTCGTCAGACTGTCG
ATGAAGCCCTGAAAGACGCGCAGACTAATTCGAGCTCGAACAACAACAACAATAACAATAACAACAACCTCGGGA
TCGAGGGAAGGATTTCAGAATTCGACAATCACATTTACTGCAATAACATTTACGATCACCACAAAAACACGTCATTG
AACTCGAAAGAACAAAATACGGACCATAACATCGAACAGATTAATGAGTGTAACAAATATGCCAGTGAAACCAAA
TACAACATTAAGAAATCGAACCTGAAGAATAACATTATCTCGCATAAGAACTTCCAGAAATGCAATCAGATTCAGAT
GAACCAACCCTACACGTTTCCACCATATCAGAAAGAACTGAGCTCTTATCTTAAAAACGAAAAGATCAAACGGAAA
CGCAAAGTGCTGTTTTCCTACCTGAAAACCCATATTCACTTCAACAGTCAGCAGATTAACGATCAACACAATCGCTT
ATCTGTGCAGAAAATTATGAAAATCATTACTGATGTCACCCTTGCTTGCACATATCTGGAAAAGGAGAAAATGAGC
CCCATTGATCTGAAACCTACCAATATCCTGTTGGACGAATCTCTGAATGCCAAAATTAGCGATTTCGGTATCAGCAA
AATTGAGAATTGTCTGGATATGAACATTGACTATTCATACAAAATTAGCTCCAACTCAGTCATCAAAATTAATAAAA
AAGAATATGAGCAAAAGAAAGCGAAGAAAATCAAAATTGTGAATAAAAACAATAATGACTTACTGTACCTGTACG
ATCACAATAACAACGTCTATAAGTACAATACCCAATATATCGACGTAACCTATAACAACAGTTATCCGAGCATCTTC
TATTGGACTCCTCCGGAAATCTTACGTGGC-AAAAAAACAAGGAATTTTACAGGGA-ATTTACGCATTTGGGATC-
TCCTTCTGGGAATGGTGAGTAAGGA-ATTCCGGAC-ACTATCCGTTT-CAT-
CCCCATTTAGGGCGGTGGAAGCTAAGC-AATGAAAAATGGCTTCCA---AACATTCCGGTTCC-------

TTACCGCTTGAACAACGTGGGAATCCCA--AAAATAAGGCGCC---AGTTGGAA---TTTCCGAAAG-------
TCTCCTCCTTCAAAAGGAAAC-----AAGGGGAAGTTCTCCCTTTTGG--
GGGGTCCGG--CACCCCCCTTT------

>pTKL_KD_DG_clone9_F

CCGGGGTGTGGGCGAGATCCACGTATTGCCGCCACTATGGAAAACGCCCAGAAAGGTGAAATCATGCCGAACATC
CCGCAGATGTCCGCTTTCTGGTATGCCGTGCGTACTGCGGTGATCAACGCCGCCAGCGGTCGTCAGACTGTCGATG
AAGCCCTGAAAGACGCGCAGACTAATTCGAGCTCGAACAACAACAACAATAACAATAACAACAACCTCGGGATCG
AGGGAAGGATTTCAGAATTCGACAATCACATTTACTGCAATAACATTTACGATCACCACAAAAACACGTCATTGAAC
TCGAAAGAACAAAATACGGACCATAACATCGAACAGATTAATGAGTGTAACAAATATGCCAGTGAAACCAAATAC
AACATTAAGAAATCGAACCTGAAGAGTAACATTATCTCGCATAAGAACTTCCAGAAATGCAATCAGATTCAGATGA
ACCAACCCTACACGTTTCCACCATATCAGAAAGAACTGAGCTCTTATCTTAAAAACGAAAAGATCAAACGGAAACG
CAAAGTGCTGTTTTCCTACCTGAAAACCCATATTCACTTCAACAGTCAGCAGATTAACGATCAACACAATCGCTTATC
TGTGCAGAAAATTATGAAAATCATTACTGATGTCACCCTTGCTTGCACATATCTGGAAAAGGAGAAAATGAGCCCC
ATTAATCTGAAACCTACCAATATCCTGTTGGACGAATCTCTGAATGCCAAAATTAGCGGTTTCGAAATCAGCAAAAT
TGAGAATTGTCTGGATATGAACATTGACTATTCATACAAAATTAGCTCCAACTCAGTCATCAAAATTAATAAAAAAG
AATATGAGCAAAAGAAAGCGAAGAAAATCAAAATTGTGAATAAAAACAATAATGACTTACTGTACCTGTACGATCA
CAATAACAACGTCTATAAGTACAATACCCAATATATCGACGTAACCTATAAC-
ACAGTTATCCGAGCATCTTCTATTGGACTCCTCCGGAAATCTTACGTGGC-AAAAAAACA--GAAATTTTCAGCGA-
TATTACGC-TTTGGGATC-TCCCCTGGGAAA--

Proteins

>PF3D7_1106800_KD

DNHIYCNNIYDHHKNTSLNSKEQNTDHNIEQINECNKYASETKYNIKKSNLKNNIISHKNFQKCNQIQMNQPYTFPPYQK
ELSSYLKNEKIKRKRKVLFSYLKTHIHFNSQQINDQHNRLSVQKIMKIITDVTLACTYLEKEKMSPINLKPTNILLDESLNAKIS
DFGISKIENCLDMNIDYSYKISSNSVIKINKKEYEQKKAKKIKIVNKNNNDLLYLYDHNNNVYKYNTQYIDVTYNNSYPSIFY
WTPPEILRGKKNKKFYSDIYAFGIILWEMLSNDIPYNYPFASHIMAVVGYANEELSFNNIPVSIQSLIKACVNRNKYKRPTF
EHILKTISTLYQKANTKVEDALISFMDGT--------

>pTKL_KD_DG

EFDNHIYCNNIYDHHKNTSLNSKEQNTDHNIEQINECNKYASETKYNIKKSNLKSNIISHKNFQKCNQIQMNQPYTFPPY
QKELSSYLKNEKIKRKRKVLFSYLKTHIHFNSQQINDQHNRLSVQKIMKIITDVTLACTYLEKEKMSPINLKPTNILLDESLNA
KISGFEISKIENCLDMNIDYSYKISSNSVIKINKKEYEQKKAKKIKIVNKNNNDLLYLYDHNNNVYKYNTQYIDVTYNNSYPSI
FYWTPPEILRGKKNKKFYSDIYAFGIILWEMLSNDIPYNYPFASHIMAVVGYANEELSFNNIPVSIQSLIKACVNRNKYKRP
TFEHILKTISTLYQKANTKVEDALISFMDGTLEHHHHHH

>pTKL_KD_KRIMAS

EFDNHIYCNNIYDHHKNTSLNSKEQNTDHNIEQINECNKYASETKYNIKKSNLKNNIISHKNFQKCNQIQMNQPYTFPPY
QKELSSYLKNEKIKRKRIMASSYLKTHIHFNSQQINDQHNRLSVQKIMKIITDVTLACTYLEKEKMSPINLKPTNILLDESLN
AKISDFGISKIENCLDMNIDYSYKISSNSVIKINKKEYEQKKAKKIKIVNKNNNDLLYLYDHNNNVYKYNTQYIDVTYNNSYP
SIFYWTPPEILRGKKNKKFYSDIYAFGIILWEMLSNDIPYNYPFASHIMAVVGYANEELSFNNIPVSIQSLIKACVNRNKYKR
PTFEHILKTISTLYQKANTKVEDALISFMDGTLEHHHHHH

>pTKL_KD_ND

EFDNHIYCNNIYDHHKNTSLNSKEQNTDHNIEQINECNKYASETKYNIKKSNLKNNIISHKNFQKCNQIQMNQPYTFPPY
QKELSSYLKNEKIKRKRKVLFSYLKTHIHFNSQQINDQHNRLSVQKIMKIITDVTLACTYLEKEKMSPIDLKPTNILLDESLNA
KISDFGISKIENCLDMNIDYSYKISSNSVIKINKKEYEQKKAKKIKIVNKNNNDLLYLYDHNNNVYKYNTQYIDVTYNNSYPSI
FYWTPPEILRGKKNKKFYSDIYAFGIILWEMLSNDIPYNYPFASHIMAVVGYANEELSFNNIPVSIQSLIKACVNRNKYKRP
TFEHILKTISTLYQKANTKVEDALISFMDGTLEHHHHHH

>pTKL_KD_WT

EFDNHIYCNNIYDHHKNTSLNSKEQNTDHNIEQINECNKYASETKYNIKKSNLKNNIISHKNFQKCNQIQMNQPYTFPPY
QKELSSYLKNEKIKRKRKVLFSYLKTHIHFNSQQINDQHNRLSVQKIMKIITDVTLACTYLEKEKMSPINLKPTNILLDESLNA
KISDFGISKIENCLDMNIDYSYKISSNSVIKINKKEYEQKKAKKIKIVNKNNNDLLYLYDHNNNVYKYNTQYIDVTYNNSYPSI
FYWTPPEILRGKKNKKFYSDIYAFGIILWEMLSNDIPYNYPFASHIMAVVGYANEELSFNNIPVSIQSLIKACVNRNKYKRP
TFEHILKTISTLYQKANTKVEDALISFMDGTLEHHHHHH

Supporting Information 11 – Sequencing data *Pf*SERA5 recombinant protein

Raw DNA sequencing data

>PF3D7_0207600_SERA5

ATGAAGTCATATATTTCCTTGTTTTTCATATTGTGTGTTATATTTAACAAAAATGTTATAAAATGTACAGGAGAAAGT
CAAACAGGTAATACAGGAGGAGGTCAAGCAGGTAATACAGGAGGAGATCAAGCAGGTAGTACAGGAGGAAGTC
CACAAGGTAGTACGGGAGCAAGTCCACAAGGTAGTACGGGAGCAAG---
TCCACAAGGTAGTACGGGAGCAAGTCAACCCGGAAGTTCCGAACCAAGCAATCCTGTAAGTTCCGGACATTCTGTA
AGTACTGTATCAGTATCACAAACTTCAACTTCTTCAGAAAAACAGGATACAATTCAAGTAAAATCAGCTTTATTAAA
AGATTA-------------TA-
TGGGTTTAAAAGTTACTGGTCCATGTAACGAAAATTTCATAATGTTCTTAGTTCCTCATATATATATTGATGTTGATA
CAGAAGATACTAATATCGAATTAAGAACAACATTGAAAAAAACAAATAATGCAATATCATTTGAATCAAACAGTGG
TTCATTAGAAAAAAAAAAATATGTAAAACTACCATCAAATGGTACAACTGGTGAACAAGGTTCAAGTACGGGAACA
GTTAGAGGAGATACAGAACCAATTTCAGATTCAAGCTCAAGTTCAAGTTCAAGCTCTAGTTCAAGTTCAAGTTCAA
GTTCAAGTTCTAGTTCAAGTTCTAGTTCAAGTTCAGAAAGTCTTCCTGCTAATGGACCTGATTCCCCTACTGTTAAAC
CGCCAAGAAATTTACAAAATATATGTGAAACTGGAAAAAACTTCAAGTTGGTAGTATATATTAAGGAGAATACATT
AATACTTAAATGGAAAGTATACGGAGAAACAAAAGATACTACTGAAAATAACAAAGTTGATGTAAGAAAGTATTT
GATAAATGAAAAGGAAACCCCATTTACTAATATACTAATACATGCGTATAAAGAACATAATGGAACAAACTTAATA
GAAAGTAAAAACTACGCAATAGGATCAGACATTCCAGAAAAATGTGATACCTTAGCTTCCAATTGCTTTTTAAGTG
GTAATTTTAACATTGAAAAATGCTTTCAATGTGCTCTTTTAGTAGAAAAAGAAAATAAAAATGACGTATGTTACAAA
TACCTATCTGAAGATATTGTAAGTAAATTCAAAGAAATAAAAGC---TGAGACAGAAGATGAT-
GATGAAGATGATTATACTGAATATAAATTAACAGAA----
TCTATTGATAATATATTAGTAAAAATGTTTAAAACAAATGAAAATAATGATAAATCAGAATTAATAAAATTAGAAGA
AGTAGATGATAGTTTGAAATTAGAATTAATGAATTACTGTAGTTTACTTAAAGACGTAGATACAACAGGTACCTTAG
ATAATTATGGGATGGGAAAT-
GAAATGGATATATTTAATAACTTAAAGAGATTATTAATTTATCATTCAGAAGAAAATATTAATACTTTAAAAAATAA
ATTCCGTAATGCAGCTGTATGTCTTAAAAATGTTGATGATTGGATTGTAAATAAGAGAGGTTTAGTATTACCTGAAT
TAAATTATGATTTAGAATATTTCAATGAACATTTATATAATGATAAAAATTCTCCAGAAGATAAAGATAATAAAGGA
AAAGGTGTCGTACATGTTGATACAACTTTAGAAAAAGAAGATACTTTATCATATGATAACTCAGATAATATGTTTTG
TAATAAAGAATATTGTA--
ACAGATTAAAAGATGAAAATAATTGTATATCTAATCTTCAAGTTGAAGATCAAGGTAATTGTGATACTTCATGGATT
TTTGCTTCAAAATATCATTTAGAAACTATTAGATGTATGAAAGGATATGAACCTACCAAAATTTCTGCTCTTTATGTA
GCTAATTGTTATAAAGGTGAACATAAAGATAGATGTGATGAAGGTTCTAGTCCAATGGAATTCTTACAAATTATTG
AAGATTATGGATTCTTACCAGCAGAATCAAATTATCCATATAACTATGTGAAAGTTGGAGAACAATGTCCAAAGGT
AGAAGATCACTGGATGAATCTATGGGAT------------A---ATGGAAAAAT-------
CTTACATAACAAAAATGAACCTAATAGTTTAGATGGTAAGGGATATACTGCATATGAAAGTGAAAGATTTCATGAT
AATATGGATGCATTTGTTAAAATTATTAAAACTGAAGTAATGAATAAAGGTTCAGTTATTGCATATATTAAAGCTGA
AAATGTTATGGGATATGAATTTAGTGGAAAGAAAGTACAGAACTTATGTGGTGATGATACAGCTGATCATGCAGTT
AATATTGTTGGTTATGGTAATTATGTGAATAGCGAAGGAGAAAAAAAATCCTATTGGATTGTAAGAAACAGTTGGG
GTCCATATTGGGGAGATGAAGGTTATTTTAAAGTAGATATGTATGGACCAACTCATTGTCATTTTAACTTTATTCAC
AGTGTTGTTATATTCAATGTTGATTTACCTATGAATAATAAAACAACTAAAAAAGAATCAAAAATATATG-
ATTATTATTTAAAGGCCTCTCCAGAATTTTATCATAACCTTTACTTTAAGAATTTTAATGTTGGTAAGAAAAATTTATT

CTCTGAAAAGGAAGATAATGAAAACAACAAAAAATTAGGTAACAACTATATTATATTCGGTCAAGATACGGCAGG
ATCAGGACAAAGTGGAAAGGAAAGCAATACTGCATTAGAATCTGCAGGAACTTCAAATGAAGTCTCAGAACGTGT
TCATGTTTATCACATATTAAAACATATAAAGGATGGCAAAATAAGAATGGGTATGCGTAAATATATAGATACACAA
GATGTAAATAAGAAACATTCTTGTACAAGATCCTATGCATTTAATCCAGAGAATTATGAAAAATGTGTAAATTTATG
TAATGTGAACTGGAAAACATGCGAGGAAAAAACATCACCAGGACTTTGTTTATCCAAATTGGATACAAATAACGAA
TGTTATTTCTGTTATGTATAA

>SERA5-cloneG-pETdown

CAACAG-------TTCCCC-GGG---CA--AGGGGGCTTGC------C------CCCATA---CCCAC--GCGGAA-ACAAGC-GCT----
CATG---AGCCCGAAGTGGCGA-GC-----CCGATCTTCCC-----CATCG-GTGATGTCGGCGATATAG-
GCGCCAGCAACCGCACCT-GTGGCGCCGGTG-ATGCCGGCCACGATGC-GTCCG-GCGT----------
AGAGGATCGAGATCGATCTCGATCCCGCGAAATTAATACGACTCACTATAGGGGAATTGTGAGCGGATA------------
-----ACA--ATTCCCCTCTAG---AAATAATTTTGTTTAACTTTAAGAAGGAGATATACCA-TGGGC-----AG----CAG-
CCAT--CAC--------CATCATCA--------CC--ACAGCC---
AGGATCCGGATACAGAAGATACTAATATCGAATTAAGAACAACATTGAAAAAAACAAATAATGCAATATCATTTGA
ATCAAACAGTGGTTCATTAGAAAAAAAAAAAATATGTAAAACTACCATCAAATGGTACAACTGGTGAACAAGGTTCA
AGTACGGGAACAGTTAGAGGAGATACAGAACCAATTTCAGATTCAAGCTCAAGTTCAAGTTCAAGCTCTAGTTCAA
GTTCAAGTTCAAGTTCAAGTTCTAGTTCAAGTTCTAGTTCAAGTTCAGAAAGTCTTCCTGCTAATGGACCTGATTCCC
CTACTGTTAAACCGCCAAGAAATTTACAAAATATATGTGAAACTGGAAAAAACTTCAAGTTGGTAGTATATATTAAG
GAGAATACATTAATACTTAAATGGAAAGTATACGGAGAAACAAAAGATACTACTGAAAATAACAAAGTTGATGTA
AGAAAGTATTTGATAAATGAAAAGGAAACCCCATTTACTAATATACTAATACATGCGTATAAAGAACATAATGGAA
CAAACTTAATAGAAAGTAAAAACTACGCAATAGGATCAGACATTCCAGAAAAATGTGATACCTTAGCTTCCAATTG
CTTTTTAAGTGGTAATTTTAACATTGAAAAATGCTTTCAATGTGCTCTTTTAGTAGAAT------------------------TC-------
-----G-------------------------------AGC---TC-----------GGC-G---------------------------------CG----CCT-----------------------
---GCA--------------------------G----------------
---G-------TCCGACAAG--C--------------------TTGCGGCCGCAT--------
AA-GCT---TAAG-----------------TC--

--------A-------------AACG----------------------------------G--
------------------------------AA---A------------------------------GA----A----------------
----------TG

>SERA5-cloneG-pETup

GCT-----------TTCCC-AA--------TTCCGGCCGAAA-----T------TTAATA--
----

------------ACGACTCACTATAGGGGAATTGTGAGCGGATA-----------------ACA--ATTCCCCTCTAG---
AAATAATTTTGTTTAACTTTAAGAAGGAGATATACCA-TGGGC-----AG----CAG-CCAT--CAC--------CATCATCA------
--CC--ACAGCC---
AGGATCCGGATACAGAAGATACTAATATCGAATTAAGAACAACATTGAAAAAAACAAATAATGCAATATCATTTGA
ATCAAACAGTGGTTCATTAGAAAAAAAAAAATATGTAAAACTACCATCAAATGGTACAACTGGTGAACAAGGTTCA
AGTACGGGAACAGTTAGAGGAGATACAGAACCAATTTCAGATTCAAGCTCAAGTTCAAGTTCAAGCTCTAGTTCAA
GTTCAAGTTCAAGTTCAAGTTCTAGTTCAAGTTCTAGTTCAAGTTCAGAAAGTCTTCCTGCTAATGGACCTGATTCCC
CTACTGTTAAACCGCCAAGAAATTTACAAAATATATGTGAAACTGGAAAAAACTTCAAGTTGGTAGTATATATTAAG
GAGAATACATTAATACTTAAATGGAAAGTATACGGAGAAACAAAAGATACTACTGAAAATAACAAAGTTGATGTA
AGAAAGTATTTGATAAATGAAAAGGAAACCCCATTTACTAATATACTAATACATGCGTATAAAGAACATAATGGAA
CAAACTTAATAGAAAGTAAAAACTACGCAATAGGATCAGACATTCCAGAAAAAATGTGATACCTTAGCTTCCAATTG
CTTTTTAAGTGGTAATTTTAACATTGAAAAATGCTTTCAATGTGCTCTTTTAGTAGAAT-----------------------TC-------
-----G------------------------------AGC---TC-----------GGC-G--------------------------------CG----CCT----------------------
---GCA-------------------------G----------------
---G-------TC-GACAAG--C-------------------TTGCGGCCGCAT--------
AATGCT--TAAG-----------------TC---GAACA------------------------GAA-----AGT-
AA---TC--------------------GT---------------ATTGTACACGGCCGCA--------
-----TA----------ATC--------GAA----------AT--TAATA---CG-ACTCA------
-------------------CTA-------TA--GG---------------------------------------GGA---AT-----T--
------------GTGAG---CGGATAACAATTCCCC---------------------ATC---TTA-----------
--------------------------GTA---TATTAGTT-AAG-------------TAT------------
----------AAAA--AGGAGA-
---------TATA------CATA-------TGG------------GC---------AGATCTC-AA------T----TGGATA---TC-------------------------
-----------C--GGCC-----------GGCCC--CCCGA--------------------------------
---------------T-----CGCTG------------------------ACGTC--GGTAC--
----------CC---------TCCA------G------TCTG---------------------------------GG---
---AA---AAAAACC-----GCTGCC-TG-----
CCAAATTTGA-----A---C----G----------------CAA

>SERA5_Y2H_3457

AGA-----------T-----------------G------AGA--------------CATA-------C-------------------------G---ACG-----------TAC-----
CAGAT-----------------------------------TACG--------CTCA--

TATGAAGCAGTGGTATCAACGCAG-----AG----TGG-CCAT----------------T----------------ACGGCC--------
GGGGATACAGAAGATACTAATATCGAATTAAGAACAACATTGAAAAAAACAAATAATGCAATATCATTTGAATCAA
ACAGTGGTTCATTAGAAAAAAAAAAATATGTAAAACTACCATCAAATGGTACAACTGGTGAACAAGGTTCAAGTAC
GGGAACAGTTAGAGGAGATACAGAACCAATTTCAGATTCAAGCTCAAGTTCAAGTTCAAGCTCTAGTTCAAGTTCA
AGTTCAAGTTCAAGTTCTAGTTCAAGTTCTAGTTCAAGTTCAGAAAGTCTTCCTGCTAATGGACCTGATTCCCCTACT
GTTAAACCGCCAAGAAATTTACAAAATATATGTGAAACTGGAAAAAACTTCAAGTTGGTAGTATATATTAAGGAGA
ATACATTAATACTTAAATGGAAAGTATACGGAGAAACAAAAGATACTACTGAAAATAACAAAGTTGATGTAAGAAA
GTATTTGATAAATGAAAAGGAAACCCCATTTACTAATATACTAATACATGCGTATAAAGAACATAATGGAACAAACT
TAATAGAAAGTAAAAACTACGCAATAGGATCAGACATTCCAGAAAAAATGTGATACCTTAGCTTCCAATTGCTTTTTA

AGTGGTAATTTTAACATTGAAAAATGCTTTCAATGTGCTCTTTTAGTAAAAAAAAAAAAA--------------------------------
-------------AAAAAAAAAAAAAAAAAAAACATGTC-----------GGCCG---------------------------------CCTCGGCCT--------
--CTC-----------GA------T--------
------------CTAGAGGGTGGCATC-GAT------A---------------CGGGAT-------CCATC------------------GAGCTC-----------
----------GAGCTGCAG---------------ATGA----ATCGT--
AGATACTGAA--AAA--CCCC-GCA-----AGT---------------------------T-----------------------CACTT----------CAACT--------
GTGC---------------ATCGTGCACC--------------------------------ATCT-CAATTT---
CTTTCATTTATACAT----------------CGTTTTG--C----C-----TTCT----TTTATGTAACTA-------TA-----------------------------
-------CTCC-----------TCTA---------AGT-------TTC----------AATC---TTGGCCATG--------T-----------AAC------------------
--CTCTG-----ATCTATAGAATTTTTTAAATGACTAGAAT-----TAATGCCCATCTTT--------------------------------------
TTT---TTGGAACTAAAT--------------TC-----TT-CA-----------TGAAA------
---AT-------------------------------TATT------
TACGA-------GG------------GC---------TTATT-CAAAGC-------TTTGGACT---TCTTCG---------------CCA----------------
AAGGTTG-----------GG---CAAGT--CCC-------AATCAAGG---------TT-----------GCCGC---------TT---------GCTACTT----
------------GCC-------------------------A---------AAATTACAAAAAT--GGGAA---------------------A-----GGCA-AATC-----
---GTG----GGAAACC--------TTTTGA-CC---------TCCAA------------AACC-----------------------------------G-----------------
-------------------ATTCCTTAGT------TTA-----------------G---------------TTTA------TATTAA--------AAATTTA--------------
---------------AAAAATA-----GGGACTTTATTAGGCC---TTGGATACAAA--------------------------AC

Proteins

>PF3D7_0207600_SERA5

MKSYISLFFILCVIFNKNVIKCTGESQTGNTGGGQAGNTGGDQAGSTGGSPQGSTGASPQGSTGASPQGSTGASQPGS
SEPSNPVSSGHSVSTVSVSQTSTSSEKQDTIQVKSALLKDYMGLKVTGPCNENFIMFLVPHIYIDVDTEDTNIELRTTLKKT
NNAISFESNSGSLEKKKYVKLPSNGTTGEQGSSTGTVRGDTEPISDSSSSSSSSSSSSSSSSSSSSSSSSSSSSSSSSESLPANGPDSPT
VKPPRNLQNICETGKNFKLVVYIKENTLILKWKVYGETKDTTENNKVDVRKYLINEKETPFTNILIHAYKEHNGTNLIESKNY
AIGSDIPEKCDTLASNCFLSGNFNIEKCFQCALLVEKENKNDVCYKYLSEDIVSKFKEIKAETEDDDEDDYTEYKLTESIDNIL
VKMFKTNENNDKSELIKLEEVDDSLKLELMNYCSLLKDVDTTGTLDNYGMGNEMDIFNNLKRLLIYHSEENINTLKNKFR
NAAVCLKNVDDWIVNKRGLVLPELNYDLEYFNEHLYNDKNSPEDKDNKGKGVVHVDTTLEKEDTLSYDNSDNMFCNK
EYCNRLKDENNCISNLQVEDQGNCDTSWIFASKYHLETIRCMKGYEPTKISALYVANCYKGEHKDRCDEGSSPMEFLQII
EDYGFLPAESNYPYNYVKVGEQCPKVEDHWMNLWDNGKILHNKNEPNSLDGKGYTAYESERFHDNMDAFVKIIKTEV
MNKGSVIAYIKAENVMGYEFSGKKVQNLCGDDTADHAVNIVGYGNYVNSEGEKKSYWIVRNSWGPYWGDEGYFKVD
MYGPTHCHFNFIHSVVIFNVDLPMNNKTTKKESKIYDYYLKASPEFYHNLYFKNFNVGKKNLFSEKEDNENNKKLGNNYII
FGQDTAGSGQSGKESNTALESAGTSNEVSERVHVYHILKHIKDGKIRMGMRKYIDTQDVNKKHSCTRSYAFNPENYEKC
VNLCNVNWKTCEEKTSPGLCLSKLDTNNECYFCYV

>Recomb_prot_SERA5

MGSSHH--

HHHHSQDPDTEDTNIELRTTLKKTNNAISFESNSGSLEKKKYVKLPSNGTTGEQGSSTGTVRGDTEPISDSSSSSSSSSSSSS
SSSSSSSSSSSSSSSESLPANGPDSPTVKPPRNLQNICETGKNFKLVVYIKENTLILKWKVYGETKDTTENNKVDVRKYLINEK
ETPFTNILIHAYKEHNGTNLIESKNYAIGSDIPEKCDTLASNCFLSGNFNIEKCFQCALLV--

----

>Y2H_clone3457

DTEDTNIELRTTLKKTNNAISFESNSGSLEKKKYVKLPSNGTTGEQGSSTGTVRGDTEPISDSSSSSSSSSSSSSSSSSSSSSS
SSSSESLPANGPDSPTVKPPRNLQNICETGKNFKLVVYIKENTLILKWKVYGETKDTTENNKVDVRKYLINEKETPFTNILIH
AYKEHNGTNLIESKNYAIGSDIPEKCDTLASNCFLSGNFNIEKCFQCALLV---

References

1 Letunic, I. & Bork, P. 20 years of the SMART protein domain annotation resource. *Nucleic Acids Res.* **46**, D493-D496, doi:10.1093/nar/gkx922 (2018).

2 Finn, R. D. *et al.* InterPro in 2017-beyond protein family and domain annotations. *Nucleic Acids Res.* **45**, D190-D199, doi:10.1093/nar/gkw1107 (2017).

3 Dawson, N. L. *et al.* CATH: an expanded resource to predict protein function through structure and sequence. *Nucleic Acids Res.* **45**, D289-D295, doi:10.1093/nar/gkw1098 (2017).

4 de Castro, E. *et al.* ScanProsite: detection of PROSITE signature matches and ProRule-associated functional and structural residues in proteins. *Nucleic Acids Res.* **34**, W362-365, doi:10.1093/nar/gkl124 (2006).

5 Kelley, L. A., Mezulis, S., Yates, C. M., Wass, M. N. & Sternberg, M. J. The Phyre2 web portal for protein modeling, prediction and analysis. *Nat. Protoc.* **10**, 845-858, doi:10.1038/nprot.2015.053 (2015).

6 Kallberg, M. *et al.* Template-based protein structure modeling using the RaptorX web server. *Nat. Protoc.* **7**, 1511-1522, doi:10.1038/nprot.2012.085 (2012).

7 The UniProt, C. UniProt: the universal protein knowledgebase. *Nucleic Acids Res.* **45**, D158-D169, doi:10.1093/nar/gkw1099 (2017).

8 Katoh, K. & Standley, D. M. MAFFT multiple sequence alignment software version 7: improvements in performance and usability. *Mol. Biol. Evol.* **30**, 772-780, doi:10.1093/molbev/mst010 (2013).

9 Felsenstein, J. Confidence Limits on Phylogenies: An Approach Using the Bootstrap. *Evolution* **39**, 783-791, doi:10.1111/j.1558-5646.1985.tb00420.x (1985).

10 Jones, D. T., Taylor, W. R. & Thornton, J. M. The rapid generation of mutation data matrices from protein sequences. *Comput. Appl. Biosci.* **8**, 275-282 (1992).

11 Tamura, K., Stecher, G., Peterson, D., Filipski, A. & Kumar, S. MEGA6: Molecular Evolutionary Genetics Analysis version 6.0. *Mol. Biol. Evol.* **30**, 2725-2729, doi:10.1093/molbev/mst197 (2013).

12 Pettersen, E. F. *et al.* UCSF Chimera--a visualization system for exploratory research and analysis. *J. Comput. Chem.* **25**, 1605-1612, doi:10.1002/jcc.20084 (2004).

13 Crooks, G. E., Hon, G., Chandonia, J. M. & Brenner, S. E. WebLogo: a sequence logo generator. *Genome Res.* **14**, 1188-1190, doi:10.1101/gr.849004 (2004).

14 Aurrecoechea, C. *et al.* PlasmoDB: a functional genomic database for malaria parasites. *Nucleic Acids Res.* **37**, D539-543, doi:10.1093/nar/gkn814 (2009).

15 Philip, N. & Waters, A. P. Conditional degradation of *Plasmodium* calcineurin reveals functions in parasite colonization of both host and vector. *Cell Host Microbe* **18**, 122-131, doi:10.1016/j.chom.2015.05.018 (2015).

16 Bushell, E. *et al.* Functional profiling of a *Plasmodium* genome reveals an abundance of essential genes. *Cell* **170**, 260-272 e268, doi:10.1016/j.cell.2017.06.030 (2017).

17 Anderluh, G., Pungercar, J., Strukelj, B., Macek, P. & Gubensek, F. Cloning, sequencing, and expression of equinatoxin II. *Biochem. Biophys. Res. Commun.* **220**, 437-442, doi:10.1006/bbrc.1996.0391 (1996).
